# Supplementary material for: Covalent modification of a glutamic acid inspired by HaloTag technology
Source: Nat Commun. 2026 Jan 30;17:1257. doi: 10.1038/s41467-026-68999-9 (PMC12864978; doi:10.1038/s41467-026-68999-9)
Supplement: Supplementary file 1 — Supplementary Information [file 41467_2026_68999_MOESM1_ESM.pdf]

Supplementary Information

Covalent Modification of a Glutamic Acid Inspired by HaloTag Technology

Ruirui Zhang[1§], Jie Liu[1§], Raphael Gasper[2], Anke Unger[3], Farnusch Kaschani[4], Markus Kaiser[4], Petra Janning[1], Herbert Waldmann[1,5*]

[1] Max Planck Institute of Molecular Physiology, Department of Chemical Biology, Otto-Hahn-Street 11, 44227 Dortmund, Germany

[2] Max Planck Institute of Molecular Physiology, Crystallography and Biophysics Facility, Otto-Hahn-Street 11, 44227 Dortmund, Germany

[3] Lead Discovery Center GmbH, Otto-Hahn-Street 15, 44227 Dortmund, Germany

[4] Analytics Core Facility Essen (ACE), Chemical Biology, Faculty of Biology, University of Duisburg-Essen, ZMB, 45141 Essen, Germany

[5] Technical University Dortmund, Faculty of Chemistry and Chemical Biology, Otto-Hahn-Street 6, 44221 Dortmund, Germany

[§] The authors contributed equally to this work.

* Corresponding author, email: Herbert.waldmann@mpi-dortmund.mpg.de

Table of Contents

1. Supplementary Figures and Tables **2**

Supplementary Fig. S1. Structural development of PDEδ inhibitors. 2

Supplementary Fig. S2. Stability of **5e** and **6a** in aqueous buffers and in presence of GSH. 4

Supplementary Fig. S3. Fluorescence polarisation assays. 5

Supplementary Fig. S4. Phosphoproteomic profiling upon treatment of **6a.** 6

Supplementary Fig. S5. Cellular profiling of DeltaTag (**6a**) for on-target and off-target effects. 7

Supplementary Fig. S6. Mouse plasma and liver microsomal stability of DeltaTag (**6a**). 9

Supplementary Fig. S7. Examples of proteins with buried Glu/Asp for covalent targeting. 10

Supplementary Fig. S8. Applicability of alkyl bromide warhead to targeting UNC119. 12

Supplementary Fig. S9. Omit maps for 9HMC and 9HMD. 13

Supplementary Table 1. Mass spectrometry analysis of PDEδ adducts after Glu-C digestion. 14

Supplementary Table 2. In-lysate thermal proteome profiling (TPP) of **6a.** 15

Supplementary Table 3. Physiochemical properties of **6a** predicted by SwissADME. 16

Supplementary Table 4. Mass spectrometry analysis of UNC119B adducts after Lys-C digestion. 17

Supplementary Table 5. X-ray crystallography data collection and refinement statistics. 21

2. Methods **22**

Mass spectrometry analysis of UNC119B adducts after Lys-C digestion 22

In-lysate cellular thermal shift assay (CETSA) and thermal proteome profiling (TPP) 25

Phosphoproteomics 30

3. Chemical Synthesis **34**

General information 34

Compound characterisation 38

4. Supplementary Figures for Chemical Synthesis: Compound NMR Spectra **57**

5. Supplementary References **101**

1. Supplementary Figures and Tables

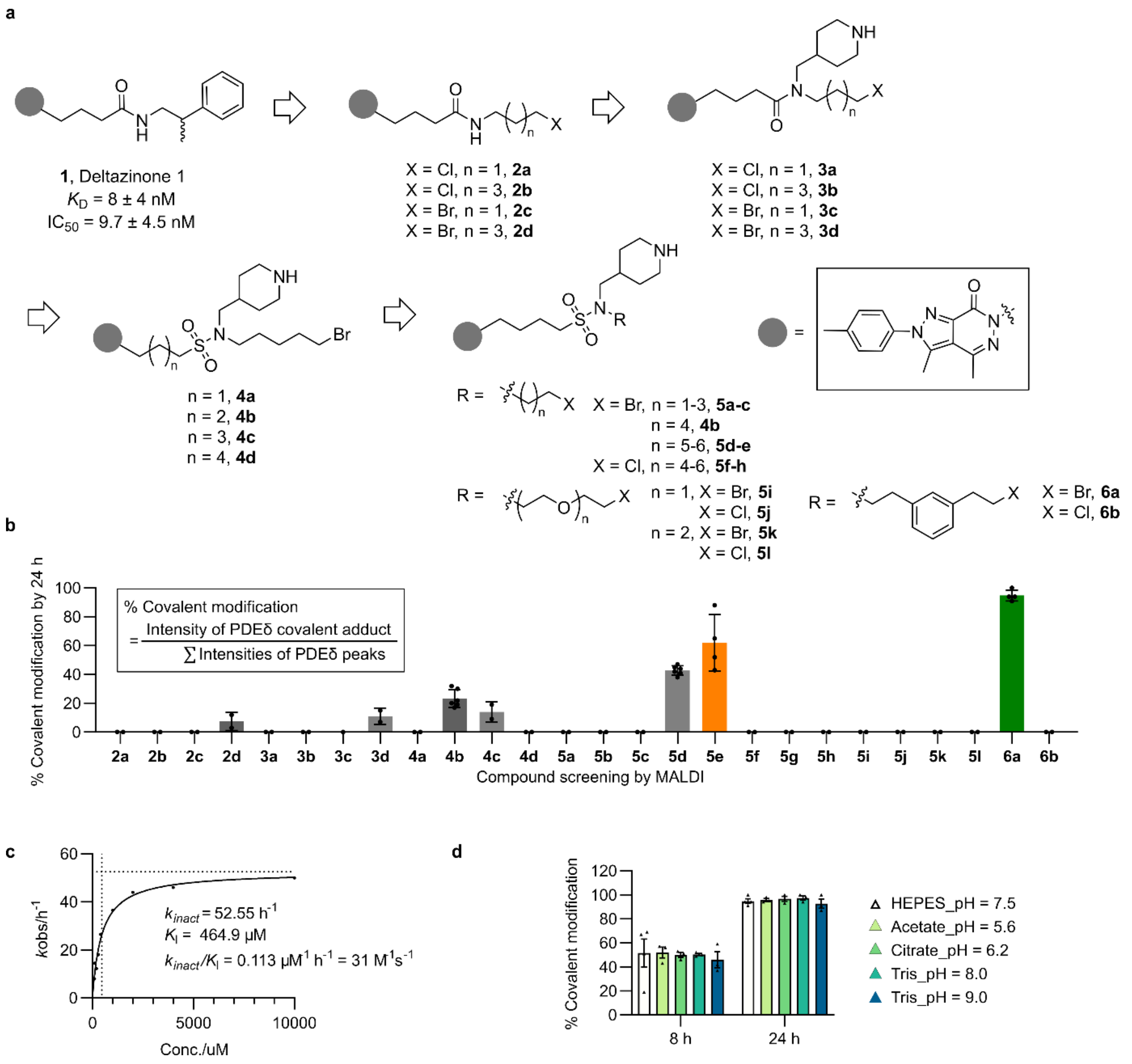

Supplementary Fig. S1. Structural development of PDEδ inhibitors.

a, Structures of Deltazinone-based compounds **1-6**. **b**, Percent (%) covalent modification at 24 hours by MALDI mass spectrometry. PDEδ (20 µM) was incubated with compounds (60 µM) in HEPES buffer (20 mM HEPES, 150 mM NaCl, pH = 7.5) at 37 °C for 24 hours before analysis by MALDI. Percentages of covalent adduct formation were estimated by the relative intensity of the respective peaks in MALDI spectra. Data are presented as mean ± s.e.m. (n = 6 for **4b** and **5d**, n = 4 for **5e** and **6a**, n = 2 for the rest). **c**, Second-order PDEδ labelling kinetics by compound **6a**. PDEδ (20 µM) was incubated with compound **6a** (60 µM – 10 mM) in HEPES buffer (20 mM HEPES, 150 mM NaCl, pH = 7.5) at 37 °C. Apparent pseudo-first order k_{obs} was estimated by percentages of covalent adduct formation at 2 hours by MALDI. Plot of k_{obs} (h^{-1}) against concentration (µM) was fitted by GraphPad

Prism Michaelis-Menten model and separate values for k_{inact} and K_I were obtained to calculate apparent second-order kinetic k_{inact}/K_I. **d**, Covalent modification of PDEδ by compound **6a** in different solvents with various pH values at 8 h and 24 h by MALDI analysis. PDEδ (20 µM) was incubated with compounds (60 µM) in respective buffers at 37 °C. Data are presented as mean ± s.e.m. (n = 4 for **6a** in HEPES buffer, n = 3 for the rest). One-way ANOVA with matched time-points, with Geisser-Greenhouse correction, p-value = 0.2852, ns. Source data are provided as a Source Data file.

a

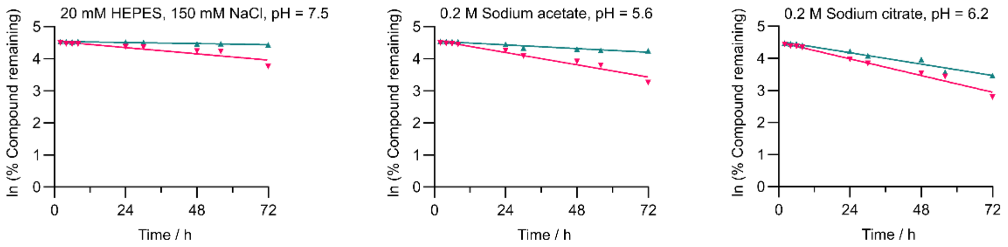

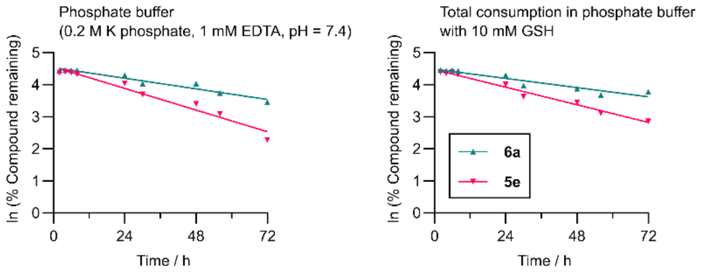

	5e	6a
k (HEPES buffer, pH = 7.5)/×10^{-3} h^{-1}	8.13	1.44
$t_{1/2}$ (HEPES buffer, pH = 7.5)/ h	85	480
k (acetate buffer, pH = 5.6) /×10^{-3} h^{-1}	15.97	4.79
$t_{1/2}$ (acetate buffer, , pH = 5.6)/ h	43	145
k (citrate buffer, pH = 6.2)/×10^{-3} h^{-1}	21.68	14.79
$t_{1/2}$ (citrate buffer, pH = 6.2)/ h	32	47
k (GSH in phosphate buffer, pH = 7.4)/×10^{-3} h^{-1}	22.87	14.47
$t_{1/2}$ (GSH in phosphate buffer, pH = 7.4)/ h	30	48
k (phosphate buffer, pH = 7.4)/×10^{-3} h^{-1}	22.71	13.80
$t_{1/2}$ (phosphate buffer, pH = 7.4)/ h	31	50
k(GSH)/×10^{-3} h^{-1}	0.16	0.67
$t_{1/2}$ (GSH)/ h	4332	1035

b

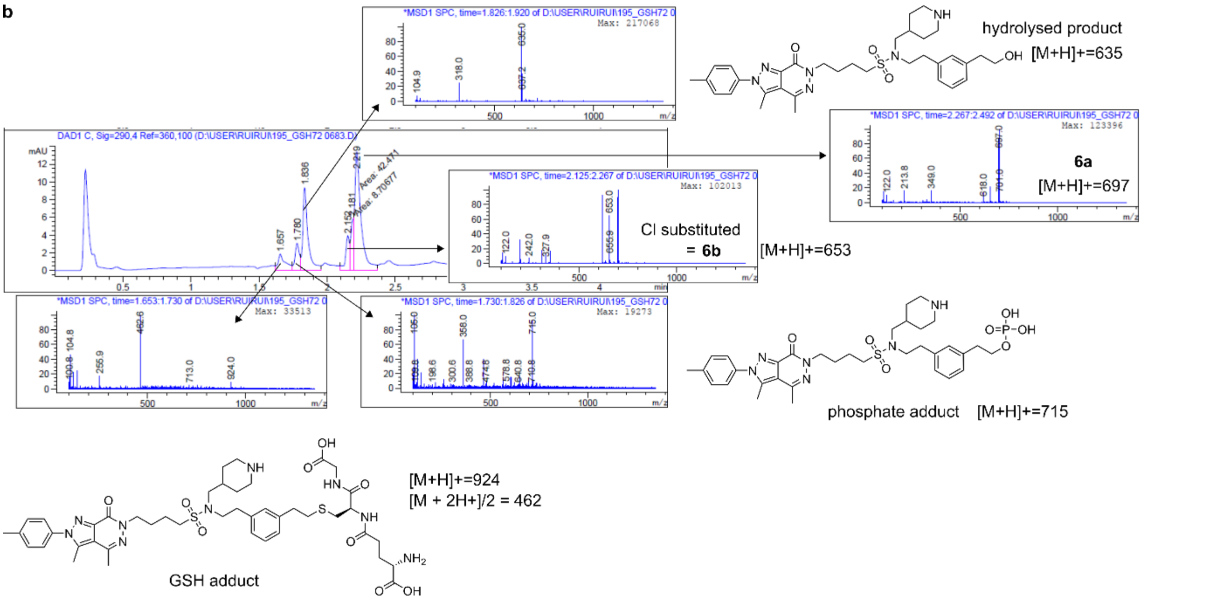

Supplementary Fig. S2. Stability of 5e and 6a in aqueous buffers and in presence of GSH.

a, Percentage (%) of compound remaining was monitored by HPLC-MS and data was fitted to pseudo-first kinetics in calculation of rate constants (k) and half-lives ($t_{1/2}$) in respective buffers, where k is the negative slope of a linear regression of In (% compound remaining) over time and $t_{1/2}$ = In2/k. Initial compound concentration = 1 mM, 37 °C, 600 rpm, protected from light. Initial GSH concentration = 10 mM in phosphate buffer. **b**, Representative LCMS spectra of **6a** incubated in phosphate buffer with GSH after 72 hours at 37 °C, with MS corresponding to GSH adduct, phosphate adduct, hydrolysed product and chloro-substituted product of **6a**. Source data are provided as a Source Data file.

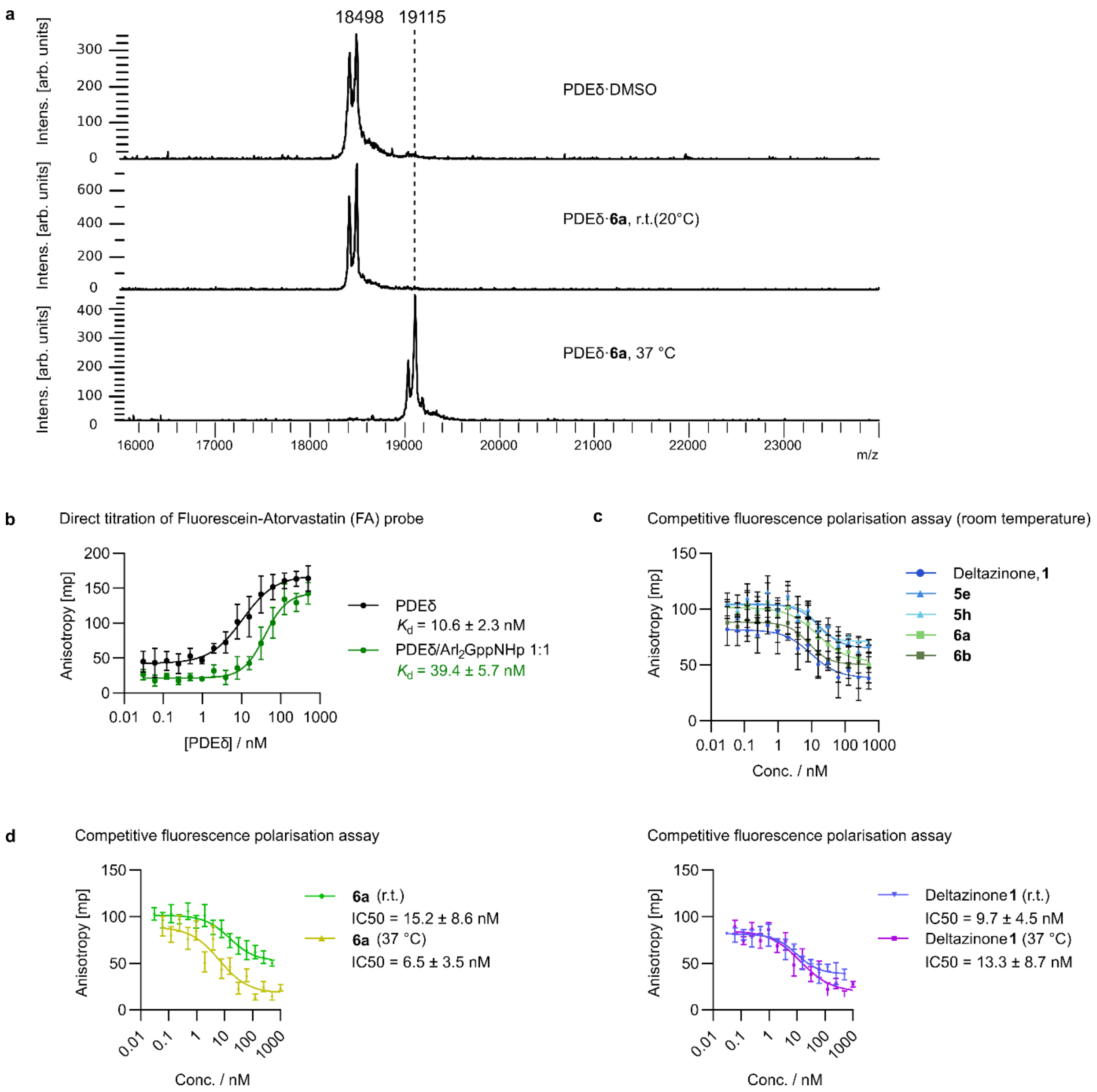

Supplementary Fig. S3. Fluorescence polarisation assays.

a, Model validation suggesting that covalent inhibitor **6a** was bound to PDEδ in a temperature dependent manner by incubating 125 µM of compounds (12.5-fold) with PDEδ (10 µM) in PBS buffer with 0.05% CHAPS and 1% of DMSO overnight at room temperature (approximately 20 °C) and 37 °C. **b**, Direct titration of FA probe (24 nM) against PDEδ at room temperature. Data are presented as mean ± s.d. (n = 3). **c**, Competitive fluorescence polarisation assay for IC_{50} values of inhibitors by incubating the compounds with PDEδ (40 nM) and FA probe (24 nM) at room temperature overnight. Data are presented as mean ± s.e.m. (n = 3 for **1, 5e-h** and n = 4 for **6a-6b**). **d**, Competitive fluorescence polarisation assay for IC_{50} values of inhibitors by incubating the compounds with PDEδ (40 nM) and FA probe (24 nM) at room temperature or 37 °C overnight. Data are presented as mean ± s.e.m. (n = 4 for **6a** at r.t., n = 3 for the rest). Source data are provided as a Source Data file.

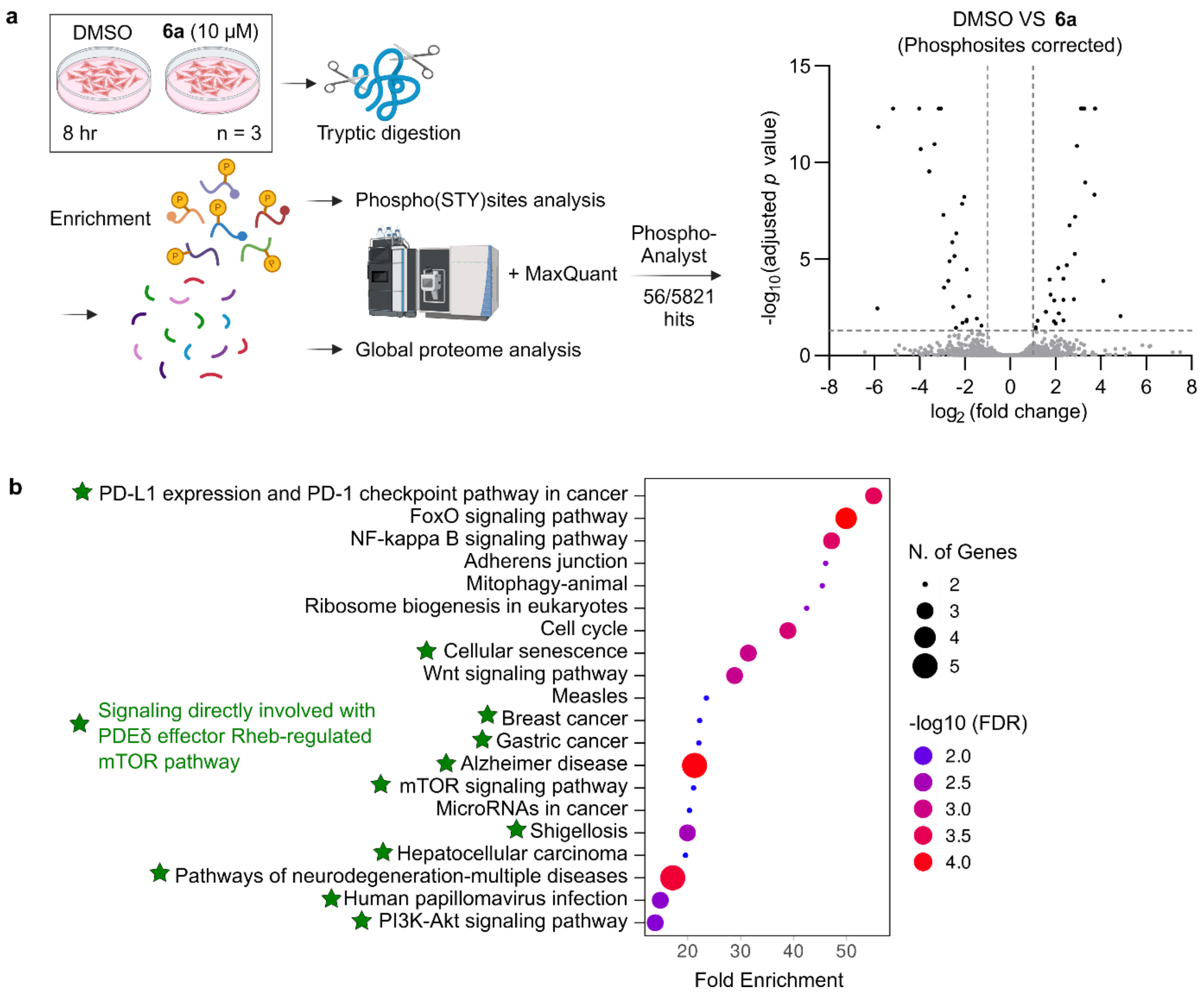

Supplementary Fig. S4. Phosphoproteomic profiling upon treatment of 6a.

a, Workflow of phosphoproteomic profiling upon treatment of **6a** in NCI-H358 cells. Output files from MaxQuant were analysed by Phospho-Analyst web application[1] with volcano plot for changes in phosphosites corrected to the underlying protein abundance (adjust two-tailed p-value ≤ 0.05, $\log_2$ fold change ≥ 1 or ≤ -1). Illustrative image was created in BioRender. Zhang, R. (2026) https://BioRender.com/q3k1jqg. **b**, KEGG pathway enrichment of downregulated kinase genes by ShinyGO 0.80 (FDR cutoff = 0.05, 20 pathways of top fold enrichment were shown).[2-4] Pathways directly involved with Rheb-regulated mTOR pathways were labelled with green stars. Source data are provided as a Source Data file.

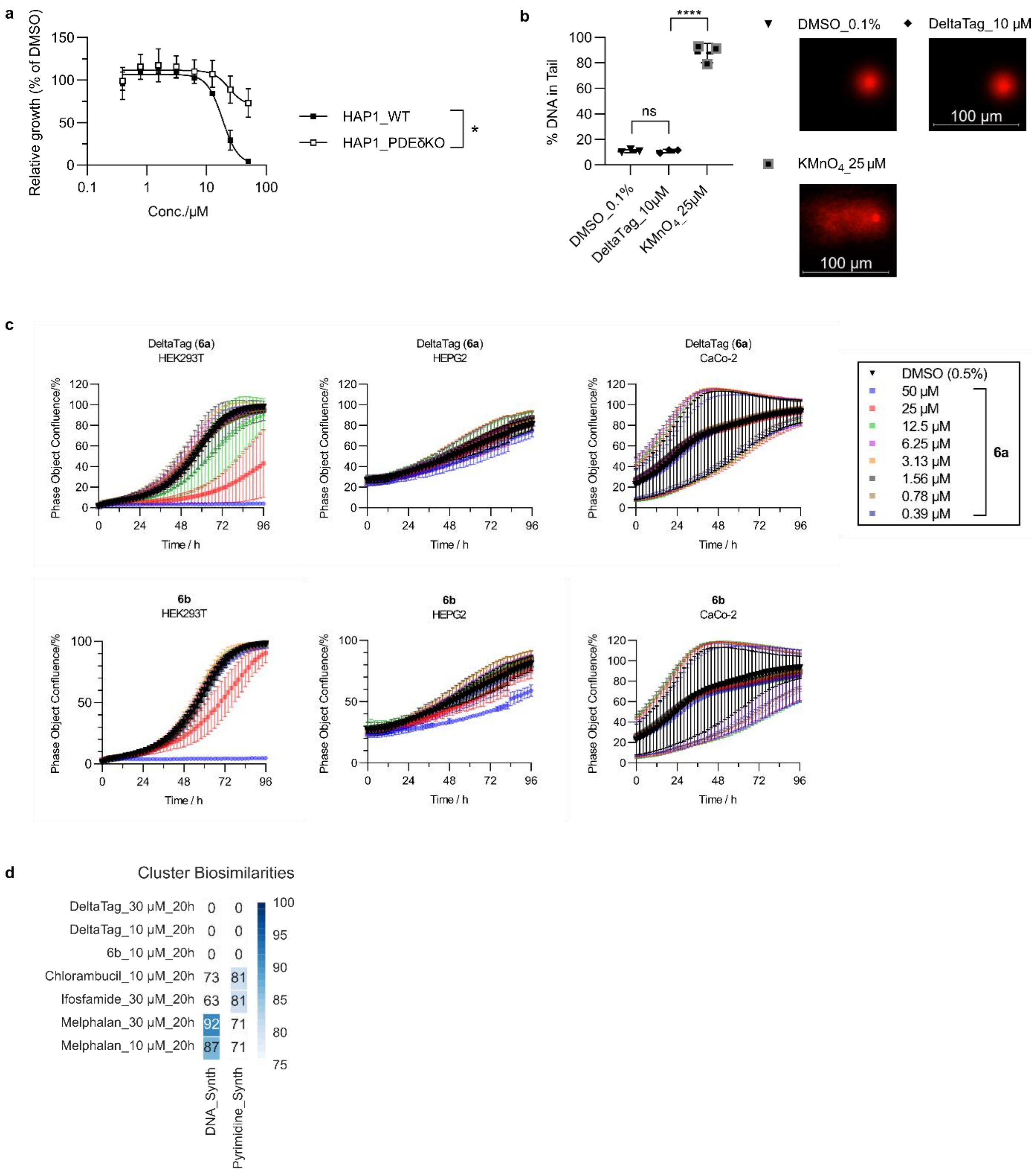

Supplementary Fig. S5. Cellular profiling of DeltaTag (6a) for on-target and off-target effects.

a, Relative growth of respective cell lines after 72 hours of DeltaTag treatment normalised to DMSO control. Data were plotted as mean ± s.d. (representative of biological replicates n = 3 in HAP1 wild type cells, n = 5 in HAP1 PDEδ knockout cells). Paired *t*-test, two-tailed *p*-value = 0.049. **b**, Comet assay.[5] % DNA in tail was estimated OpenComet (v1.3) plugin in Image J. Jurkat cells were treated with 10 µM DeltaTag or vehicle DMSO 0.1% at 37 °C for 2 hours, or with 25 µM $KMnO_4$ at 4 °C for 30 min before subjecting to single-cell gel electrophoresis. Data were plotted as mean ± s.d.,

representative of biological replicates n = 3. DeltaTag vs DMSO: unpaired *t*-test, two-tailed *p*-value = 0.997, ns; DeltaTag vs $KMnO_4$: unpaired *t*-test, two-tailed *p*-value < 0.0001 (****). Representative comet images for each condition were shown. Scale bar = 100 µm. **c**, Cytotoxicity screening of **6a** and **6b** in human kidney HEK293T cells, human liver HEPG2 cells and human intestine epithelial CaCo-2 cells. Cell proliferation with dose-dependent treatment of DeltaTag (**6a**) and **6b** monitored with real-time live-cell imaging by Incucyte. Data of percent (%) phase object confluence were plotted as mean ± s.d. (representative of biological replicates n = 3). **d**, Morphological cellular profiling[6,7] of DeltaTag (**6a**) and **6b** in U2OS cells at non-toxic doses in comparison to DNA alkylating agents, with no clustering similarities to DNA synthesis and pyrimidine synthesis clusters. Source data are provided as a Source Data file.

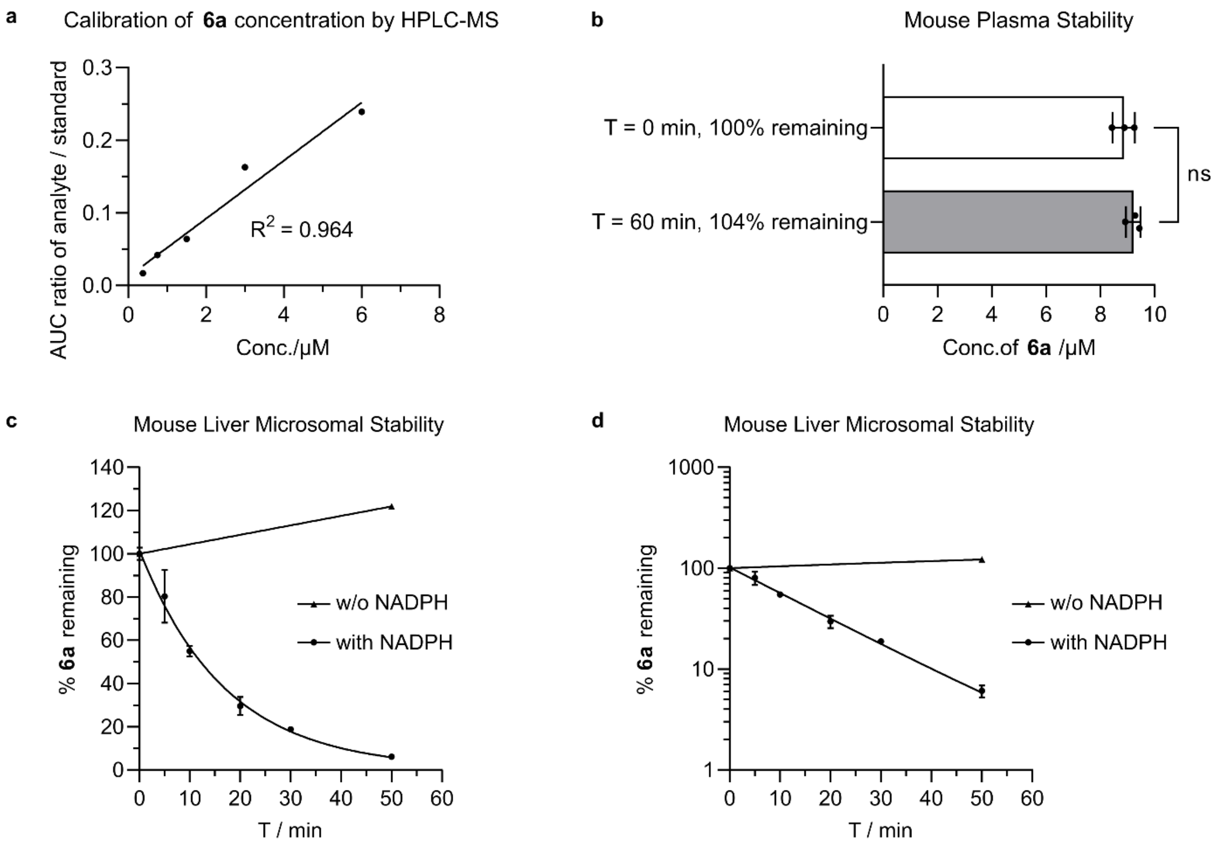

**Elimination constant K**	0.059
**$T_{1/2}$ [min]**	11.8
**CLint [μL/min/mg]**	117.5

Supplementary Fig. S6. Mouse plasma and liver microsomal stability of DeltaTag (6a).

a, Calibration curve of **6a** concentration assessed by HPLC-MS. Simple linear regression of area under the curve (AUC) of analyte **6a**/internal standard over **6a** concentration ($R^2 = 0.964$). Data were plotted as individual points (n = 1). **b**, Stability of **6a** in the presence of 50% mouse plasma in PBS. Data were plotted as mean ± standard deviation, representative of 3 technical replicates ($N = 3$). Unpaired, two-tailed *t*-test between T = 0 min and 60 min, p-value = 0.276, ns. **c** and **d**, Stability of **6a** in the presence of mouse liver microsomes (0.5 mg/ml protein), in the presence and in the absence of co-factor NADPH. Data were plotted as mean ± standard deviation, representative of 3 technical replicates in the presence of NADPH ($N = 3$). Curve of metabolism of **6a** in the presence of NADPH is fitted to a one-phase decay model in **c** ($R^2 = 0.978$) and shows a linear decline with the semi-log plot in **d**. Source data are provided as a Source Data file.

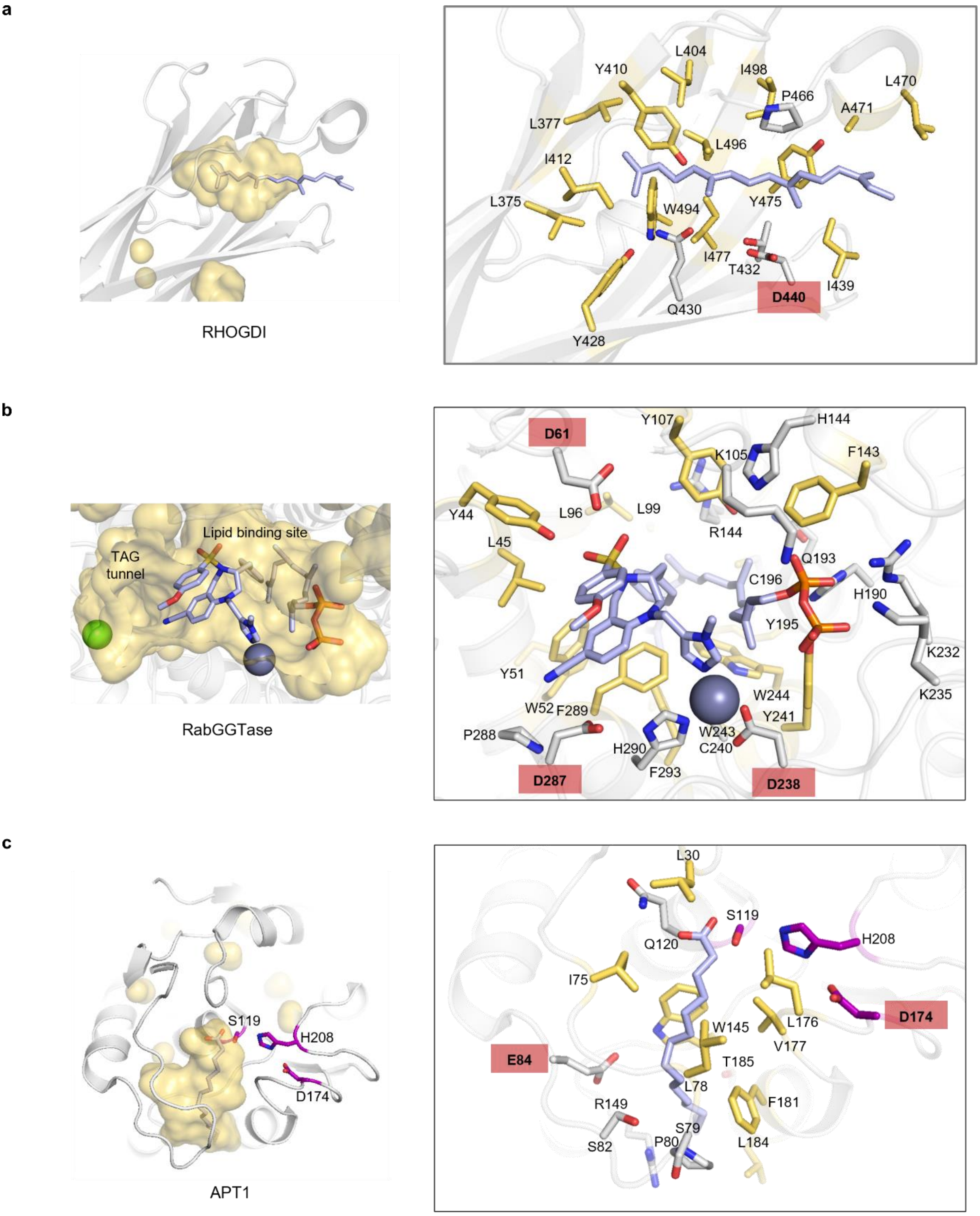

Supplementary Fig. S7. Examples of proteins with buried Glu/Asp for covalent targeting.

Cavities and protein binding sites are shown with yellow surfaces in the left panel and binding sites amino acids are labelled in the right panel, with co-crystallised small molecules in light blue, hydrophobic residues shown as yellow sticks and potential Asp/Glu residues for covalent modifications highlighted in red boxes. **a**, RHO GDP-Dissociation Inhibitor 1 (RHOGDI) co-crystallised with geran-

8-yl geran (GER) ligand (PDB code: 1HH4).[8] **b**, A closed-up view of the active site of Rab geranylgeranyl transferase (RabGGTase) co-crystallised with BMS3 and GGPP (PDB code: 3PZ2),[9] with RabGGTase-specific tunnel adjacent to the GGPP binding site (TAG tunnel) and hydrophobic lipid binding site accommodating GGPP. The green sphere represents a chlorine atom and the blue sphere represents a zinc ion. **c**, Acyl Protein Thioesterase 1 (APT1) co-crystallised with palmitic acid (PDB code: 6QGS),[10] with the catalytic triad amino acids - S119, H208 and D169 shown as purple sticks.

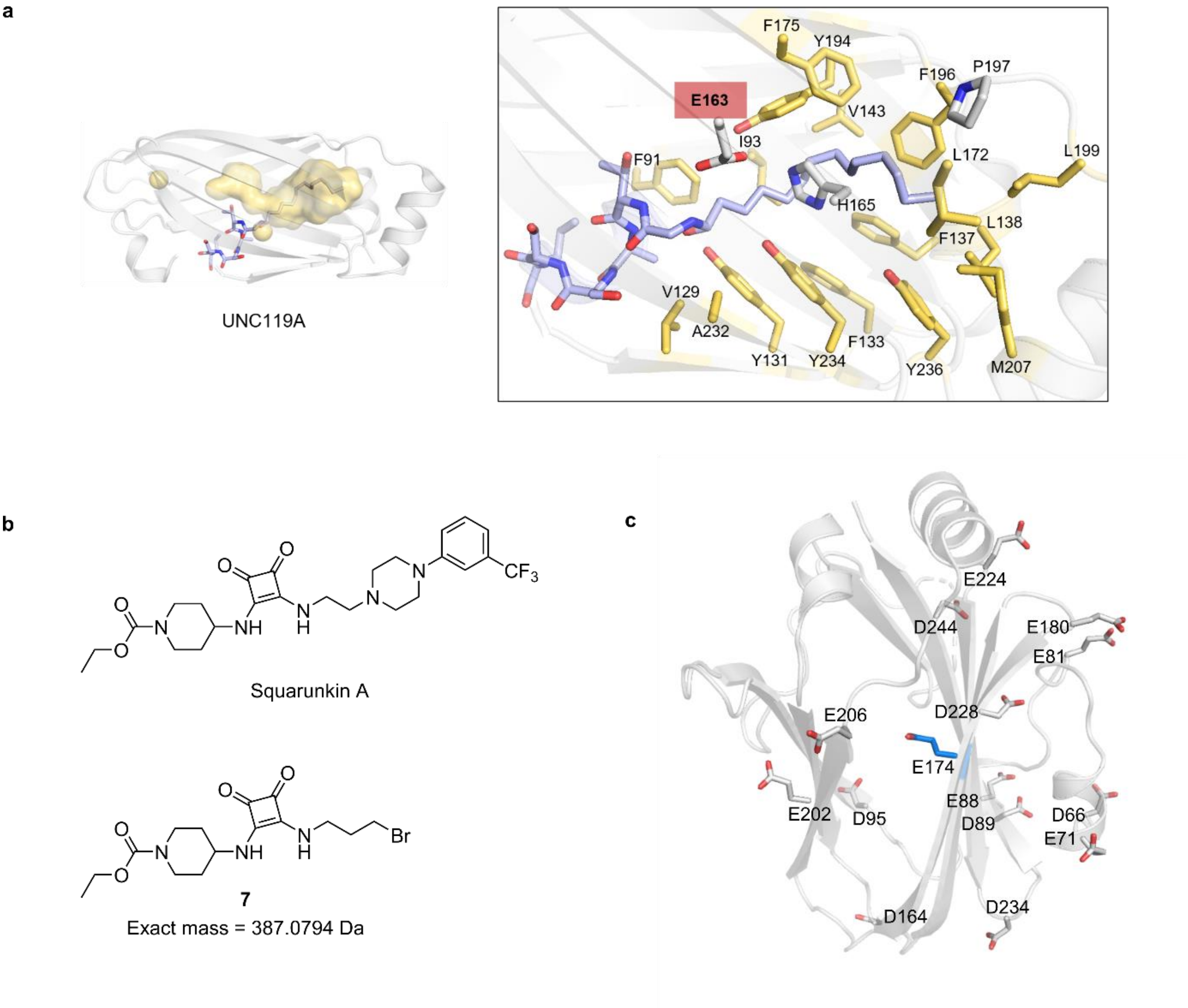

Supplementary Fig. S8. Applicability of alkyl bromide warhead to targeting UNC119.

a, Binding site analysis of UNC119A. Crystal structure of UNC119A with myristoylated NPHP3 peptide (PDB code: 5L7K).[11] Cavities and the hydrophobic pocket are shown with yellow surfaces in the left panel and binding sites amino acids are labelled in the right panel, with hydrophobic residues shown as yellow sticks and potential carboxylate residue p.E163 for covalent modifications highlighted in a red box. **b**, Structures of squarunkin A[12] and compound **7**. **c**, Acid sites in UNC119B (PDB code: 7OK7)[13] found to be covalently modified by compound **7** including binding pocket p.E174 (analogous to p.E163 in UNC119A, side chain highlighted in blue). Side chains of acid residues are shown with ball-and-stick representations.

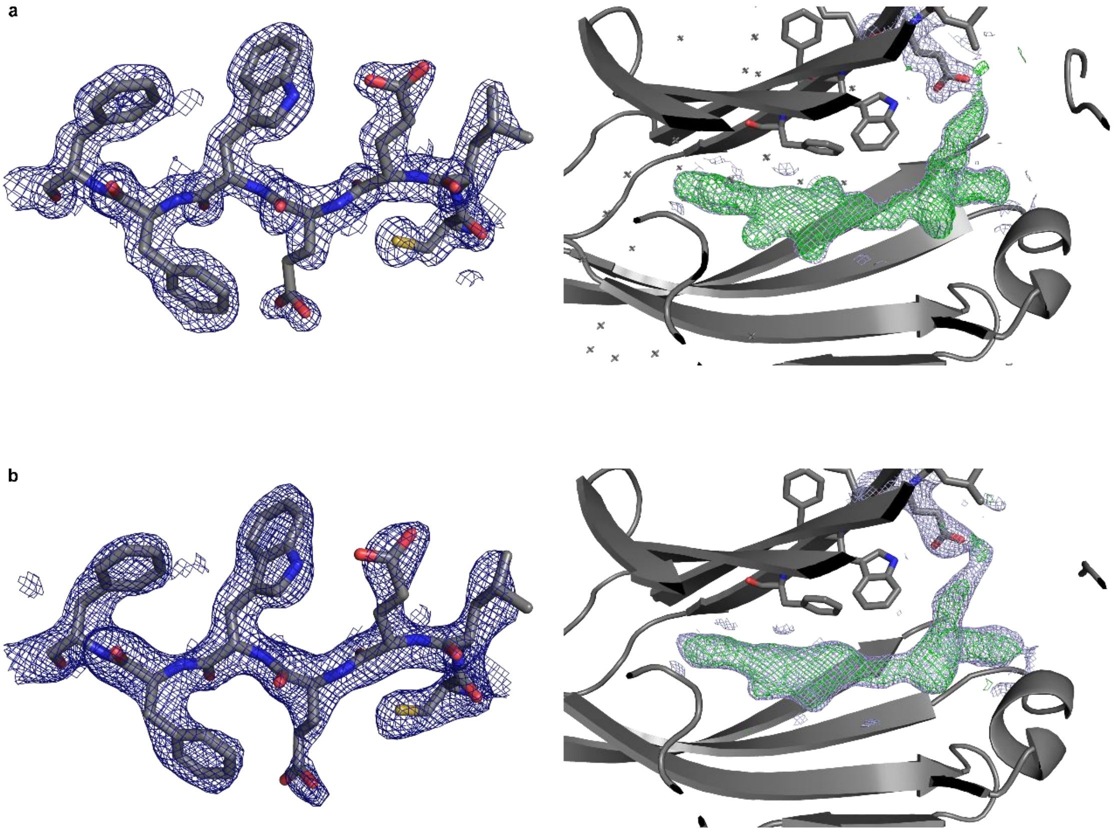

Supplementary Fig. S9. Omit maps for 9HMC and 9HMD.

The electron density maps for the crystal structures were calculated with ligand omitted, three cycles of Phenix refine, and then with Fast Fourier Transform (FFT). Electron density maps for a portion of the PDEδ molecule including p.E88 were shown in blue mesh on the left panel ($2F_o$-F_c map, contour level 1.3). Ligand maps were shown in the right panel (light blue: $2F_o$-F_c map, contour level 0.8; green: F_o-F_c map, contour level 2.3). **a,** images of electron density maps for crystal structure PDEδ•**5e** (PDB code: 9HMC). **b**, images of electron density maps for crystal structure PDEδ•**6a** (PDB code: 9HMD).

Supplementary Table 1. Mass spectrometry analysis of PDEδ adducts after Glu-C digestion.

Site modified		Peptides identified with localisation probability[a]	Score[b]	PEP[c]	Intensity[d]	Ratio modified /base[e]
PDEδ·**5e**						
R1	C86	QKVY(0.006)FK(0.359)GQC(0.628) LE(0.007)	44.425	0.003818	7.2×10^{5}	0.001308
	E88	QKVYFKGQCLE(0.941)E(0.059)	90.685	1.83×10^{-11}	1.3×10^{8}	0.24218
	E88/ E89	QKVYFKGQCLE(0.420)E(0.560)	132.76	3.15×10^{-27}	3.8×10^{7}	0.19986
R2	E88	QKVYFKGQCLE(0.814)E(0.186)	114.36	5.93×10^{-20}	9.9×10^{8}	1.8473
	E89	QKVYFKGQCLE(0.225)E(0.775)	142.97	3.21×10^{-37}	4.4×10^{8}	2.9095
R3	E88/ E89	QKVYFKGQCLE(0.453)E(0.547)	78.908	3.60×10^{-9}	2.3×10^{7}	N[f]
**Sequence coverage**						
R1	MSAKDERAREILRGFKLNWMNLRDAETGKILWQGTEDLSVPGVEHEARVPKKILKCKA VSRELNFSSTEQMEKFRLEQKVYFKGQCLEEWFFEFGFVIPNSTNTWQSLIEAAPESQMM PASVLTGNVIIETKFFDDDLLVSTSRVRLFYV					
R2	MSAKDERAREILRGFKLNWMNLRDAETGKILWQGTEDLSVPGVEHEARVPKKILKCKA VSRELNFSSTEQMEKFRLEQKVYFKGQCLEEWFFEFGFVIPNSTNTWQSLIEAAPESQMM PASVLTGNVIIETKFFDDDLLVSTSRVRLFYV					
R3	MSAKDERAREILRGFKLNWMNLRDAETGKILWQGTEDLSVPGVEHEARVPKKILKCKA VSRELNFSSTEQMEKFRLEQKVYFKGQCLEEWFFEFGFVIPNSTNTWQSLIEAAPESQMM PASVLTGNVIIETKFFDDDLLVSTSRVRLFYV					
PDEδ·**6a**						
R1	-	-	-	-	-	-
R2	E88	QKVYFKGQCLE(0.733)E(0.267)	124.12	7.65×10^{-23}	1.12×10^{8}	0.71287
	E88/ E89	QKVYFKGQCLE(0.5)E(0.5)	65.305	1.02×10^{-6}	2.20×10^{6}	N[f]
R3	E88	QKVYFKGQCLE(0.839)E(0.161)	106.01	2.18×10^{-16}	1.45×10^{7}	0.2393
	E88/ E89	QKVYFKGQCLE(0.421)E(0.579)	109.24	1.57×10^{-16}	2.25×10^{8}	3.7043
**Sequence coverage**						
R1	MSAKDERAREILRGFKLNWMNLRDAETGKILWQGTEDLSVPGVEHEARVPKKILKCKA VSRELNFSSTEQMEKFRLEQKVYFKGQCLEEWFFEFGFVIPNSTNTWQSLIEAAPESQMM PASVLTGNVIIETKFFDDDLLVSTSRVRLFYV					
R2	MSAKDERAREILRGFKLNWMNLRDAETGKILWQGTEDLSVPGVEHEARVPKKILKCKA VSRELNFSSTEQMEKFRLEQKVYFKGQCLEEWFFEFGFVIPNSTNTWQSLIEAAPESQMM PASVLTGNVIIETKFFDDDLLVSTSRVRLFYV					
R3	MSAKDERAREILRGFKLNWMNLRDAETGKILWQGTEDLSVPGVEHEARVPKKILKCKA VSRELNFSSTEQMEKFRLEQKVYFKGQCLEEWFFEFGFVIPNSTNTWQSLIEAAPESQMM PASVLTGNVIIETKFFDDDLLVSTSRVRLFYV					

a. Peptide sequences identified by searching against sequence of PDEδ (Uniprot ID, O43924) and a contaminant database with a false discovery rate of 1%, with localisation probability value (0 to 1) indicating the probability for a correct localisation of the modification from a MS/MS spectrum;
b. The Andromeda score of the best identified modified peptide containing this site;
c. The posterior error probability (PEP) of the best identified modified peptide containing this site;
d. Summed up intensities of the modified peptides;
e. Ratio of the intensities of the modified peptides to the unmodified peptides, not quantitative due to possibility of unequal ionisation efficiency;
f. No unmodified peptide of the specified sequence was identified in this sample.

Supplementary Table 2. In-lysate thermal proteome profiling (TPP) of 6a.

Proteins were considered as stabilised or destabilised when, in all three replicates,

1) they had a significant change of area under the fitted melting curves between DMSO control and DMSO treated conditions, ΔAUC of at least ± 1.5 with *p*-value ≤ 0.05 (-log *p* ≥ 1.3), where two-tailed *p*-values were determined by applying paired *t*-test comparing AUC of melting curves between DMSO-treated and compound-treated conditions, OR
2) they had a shift in melting points (ΔT_m) of at least ± 2 °C (same direction), OR
3) they showed a difference in the relative peak intensities for the highest temperature ($\Delta I_{67°C}$) of at least ± 10 % (same direction) compared to the DMSO control.

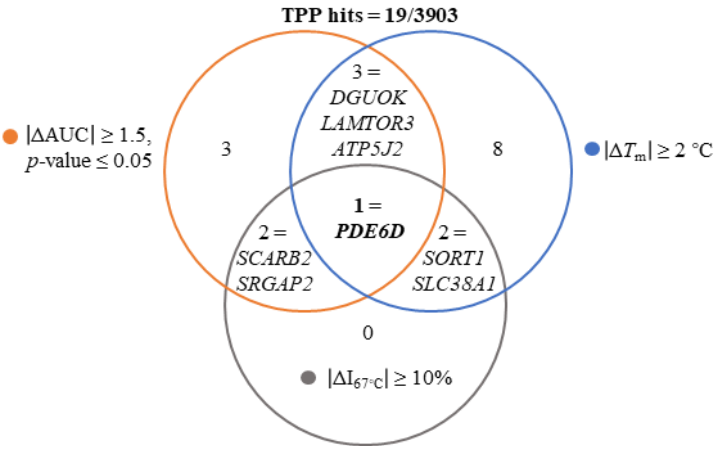

Gene names	Protein names	ΔAUC	-log *p*	ΔT_m ± s.d. / °C	$\Delta I_{67°C}$ ± s.d. / %
PDE6D	Retinal rod rhodopsin-sensitive cGMP 3,5-cyclic phosphodiesterase subunit δ	3.5	2.1	11.8 ± 0.2	21 ± 7
SCARB2	Lysosome membrane protein 2	-2.6	3.1	0.7 ± 2.1	-14 ± 4
SRGAP2	SLIT-ROBO Rho GTPase-activating protein 2	-2.5	1.3	-1.3 ± 1.1	-10 ± 7
DGUOK	Deoxyguanosine kinase, mitochondrial	-2.3	1.3	-2.9 ± 1.9	0 ± 2
LAMTOR3	Ragulator complex protein LAMTOR3	-2.1	1.6	-2.6 ± 1.4	-3 ± 2
SNRPD3	Small nuclear ribonucleoprotein Sm D3	-2	1.5	0.8 ± 0.7	-9 ± 11
ATP5J2	ATP synthase subunit f, mitochondrial	-1.8	1.8	-2.0 ± 1.0	1 ± 1
FBL	rRNA 2-O-methyltransferase fibrillarin	-1.8	1.4	-1.8 ± 1.1	-1 ± 2
RHEB	GTP-binding protein Rheb	-1.5	1.4	-0.9 ± 0.8	-6 ± 8
HINT1	Histidine triad nucleotide-binding protein 1	3.6	1.1	4.0 ± 1.4	3 ± 6
TAGLN2	Transgelin-2	1.4	0.4	2.8 ± 0.7	-5 ± 9
ACP1	Low molecular weight phosphotyrosine protein phosphatase	2.1	0.6	2.3 ± 0.3	0 ± 4
RPL31	60S ribosomal protein L31	-2.5	1.1	-2.1 ± 0.6	-6 ± 5
SORT1	Sortilin	-3.6	0.8	-2.2 ± 2.4	-16 ± 7
CNOT4	CCR4-NOT transcription complex subunit 4	-1.7	1.1	-2.7 ± 1.0	0 ± 3
HEATR1	HEAT repeat-containing protein 1	-2.9	0.7	-2.8 ± 1.1	-8 ± 6
LDHB	L-lactate dehydrogenase B chain; L-lactate dehydrogenase	-1.8	0.5	-2.9 ± 0.7	-1 ± 4
SLC38A1	Sodium-coupled neutral amino acid transporter 1	-3.3	0.4	-3.1 ± 0.5	-13 ± 5
RPLP0	60S acidic ribosomal protein P0	-2.9	0.7	-5.2 ± 2.7	-4 ± 5

Supplementary Table 3. Physiochemical properties of DeltaTag (6a) predicted by SwissADME[a].

**Physicochemical Properties**	
Formula	C34H45BrN6O3S
Molecular weight	697.73 g/mol
Num. heavy atoms	45
Num. arom. heavy atoms	21
Fraction Csp3	0.50
Num. rotatable bonds	14
Num. H-bond acceptors	7
Num. H-bond donors	1
Molar Refractivity	190.75
TPSA	110.50 Å^2

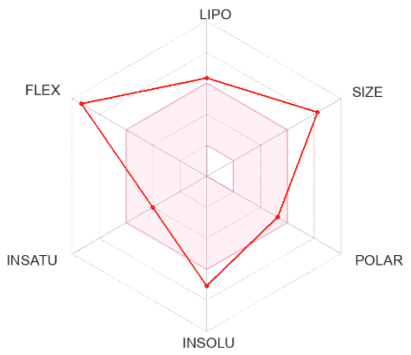

**Lipophilicity**		**Water Solubility**	
Log $P_{o/w}$ (iLOGP)	5.28	Log S (ESOL)	-7.09
Log $P_{o/w}$ (XLOGP3)	5.56	Solubility	5.67e-05 mg/ml; 8.13e-08 mol/l
Log $P_{o/w}$ (WLOGP)	5.80	Class	Poorly soluble
Log $P_{o/w}$ (MLOGP)	4.01	Log S (Ali)	-7.64
Log $P_{o/w}$ (SILICOS-IT)	5.99	Solubility	1.59e-05 mg/ml; 2.28e-08 mol/l
Consensus Log $P_{o/w}$	5.33	Class	Poorly soluble
		Log S (SILICOS-IT)	-11.14
**Pharmacokinetic Properties**		Solubility	5.01e-09 mg/ml; 7.18e-12 mol/l
GI absorption	Low	Class	Insoluble
BBB permeant	No		
PGP substrate	Yes	**Druglikeness**	
CYP1A2 inhibitor	Yes		
CYP2C19 inhibitor	No	Lipinski	Yes; 1 violation: MW>500
CYP2C9 inhibitor	No	Ghose	No; 4 violations: MW>480, WLOGP>5.6, MR>130, #atoms>70
CYP2D6 inhibitor	Yes	Veber	No; 1 violation: Rotors>10
CYP3A4 inhibitor	Yes	Egan	Yes
Log K_p (skin permeation)	-6.61 cm/s	Muegge	No; 2 violations: MW>600, XLOGP3>5
		Bioavailability Score	0.55

**Medicinal Chemistry**			
PAINS	0 alert	Leadlikeness	No; 3 violations: MW>350, Rotors>7, XLOGP3>3.5
Brenk	1 alert: alkyl_halide	Synthetic accessibility	5.02

a. SwissADME online predicator (https://www.swissadme.ch/).[14]

Supplementary Table 4. Mass spectrometry analysis of UNC119B adducts after Lys-C digestion.

	Charge	Intensity	Hyper Score	Prot. Start	Prot. End	Delta Mass	Probability	Modified Peptide Sequence
E	3	4102460	23.352	12	27	0.0006	0.9928	A.ASAAGPGGLVAGKE[436]EK.K
E	3	13300000	32.362	12	27	0.0008	0.9994	A.ASAAGPGGLVAGKE[436]EK.K
E	3	119000000	39.579	11	27	0.0006	0.999	A.AASAAGPGGLVAGKE[436]EK.K
E	3	4860540	27.525	11	28	-0.0047	0.9896	A.AASAAGPGGLVAGKE[436]EK[171]K.K
E	3	1902160	30.9	9	27	-0.0012	0.9988	A.AAAASAAGPGGLVAGKE[436]EK.K
E	3	333000000	57.153	8	27	0.0003	1	K.AAAAASAAGPGGLVAGKE[436]EK.K
E	2	9033910	43.984	8	27	0.0063	0.9999	K.AAAAASAAGPGGLVAGKE[436]EK.K
E	3	13500000	46.806	8	28	-0.0002	1	K.AAAAASAAGPGGLVAGKE[436]EK[171]K.K
E	2	2553210	34.469	8	25	0.0008	0.999	K.AAAAASAAGPGGLVAGKE[436].E
E	2	6472800	37.402	11	27	0.002	0.9999	A.A[114]ASAAGPGGLVAGKEE[436]K.K
E	3	7423920	44.901	8	28	0.0007	1	K.A[114]AAAASAAGPGGLVAGKE[436]EKK.K
E	2	2650410	25.791	13	27	0	0.9829	A.S[130]AAGPGGLVAGKE[436]EK.K
E	2	4699280	22.444	12	27	0.0009	0.9585	A.A[114]SAAGPGGLVAGKE[436]EK.K
E	2	13500000	52.112	8	27	0.0012	1	K.A[114]AAAASAAGPGGLVAGKEE[436]K.K
E	3	24800000	57.86	8	27	-0.0004	1	K.A[114]AAAASAAGPGGLVAGKEE[436]K.K
E	2	34300000	48.974	11	27	-0.0002	1	A.A[114]ASAAGPGGLVAGKE[436]EK.K
E	3	4400550	39.278	8	28	0.0012	0.9991	K.A[114]AAAASAAGPGGLVAGK[171]E[436]EK K.K
E	3	107000000	58.183	8	27	-0.0002	1	K.A[114]AAAASAAGPGGLVAGKE[436]EK.K
E	2	40000000	51.952	8	27	0.0007	1	K.A[114]AAAASAAGPGGLVAGKE[436]EK.K
E	3	9160880	44.547	8	28	0.0022	0.9997	K.A[114]AAAASAAGPGGLVAGKE[436]EK[171] K.K
E	5	8384940	46.606	76	100	0.0002	1	R.LSRVTENYLCKPE[436]DNIYSIDFTRFK.I
E	4	21800000	56.693	79	100	0.0007	1	R.VTENYLCKPE[436]DNIYSIDFTRFK.I
E	3	1587770	28.202	79	100	0	0.9999	R.VTENYLCKPE[436]DNIYSIDFTRFK.I
E	7	54300000	17.388	144	184	-0.039	0.9606	Q.FTPAFLRLRTVGATVEFTVGDKPVSNFRMIE[436]RHYFREHLLK.N
E	5	8557630	18.673	40	69	-0.0231	0.9679	K.ARRQAPHHAADDGVGAAVTE[436]QELLAL DTIR.P
E	3	1503800	40.123	79	100	1.0046	0.9999	R.V[142]TENYLCKPE[436]DNIYSIDFTRFK.I
E	3	18000000	18.679	8	27	-0.0059	0.9685	K.AAAAASAAGPGGLVAGKE[436]EK.K
E	4	10100000	25.717	8	28	-0.0004	0.9998	K.AAAAASAAGPGGLVAGKE[436]EKK.K
E	3	4197680	19.087	14	27	0.0009	0.9223	S.AAGPGGLVAGKE[436]EK.K
E	3	61100000	32.159	13	27	0.0012	0.9997	A.SAAGPGGLVAGKE[436]EK.K
E	3	28600000	32.409	12	27	0.0031	0.9997	A.ASAAGPGGLVAGKE[436]EK.K
E	2	38000000	27.087	11	27	2.0283	0.985	A.AASAAGPGGLVAGKE[436]EK.K
E	3	203000000	36.929	11	27	0.0022	1	A.AASAAGPGGLVAGKE[436]EK.K
E	3	64800000	33.414	8	28	-0.0114	0.9999	K.AAAAASAAGPGGLVAGK[171]E[436]EKK.K
E	3	30300000	43.134	10	27	0.0028	1	A.AAASAAGPGGLVAGKE[436]EK.K
E	3	16000000	20.377	11	28	-0.0062	0.9837	A.A[114]ASAAGPGGLVAGKE[436]EKK.K
E	2	28300000	46.481	8	27	-0.0004	1	K.AAAAASAAGPGGLVAGKE[436]EK.K
E	2	6010570	27.867	13	27	-0.0026	0.9999	A.SAAGPGGLVAGKE[436]EK.K
E	3	28800000	52.42	8	28	-0.0014	1	K.AAAAASAAGPGGLVAGKE[436]EK[171]K.K
E	2	7842840	39.356	8	25	-0.0009	1	K.AAAAASAAGPGGLVAGKE[436].E
E	2	14700000	44.418	11	27	-0.0011	1	A.A[114]ASAAGPGGLVAGKEE[436]K.K
E	3	15000000	49.498	8	28	0.0002	1	K.A[114]AAAASAAGPGGLVAGKE[436]EKK.K
E	2	7371090	19.674	13	27	-0.0008	0.9995	A.S[130]AAGPGGLVAGKE[436]EK.K
E	2	9662880	34.667	12	27	-0.0004	1	A.A[114]SAAGPGGLVAGKE[436]EK.K
E	2	30000000	54.412	8	27	-0.0009	1	K.A[114]AAAASAAGPGGLVAGKEE[436]K.K
E	3	48400000	58.053	8	27	-0.0014	1	K.A[114]AAAASAAGPGGLVAGKEE[436]K.K
E	2	71400000	51.304	11	27	-0.0014	1	A.A[114]ASAAGPGGLVAGKE[436]EK.K
E	2	5238120	15.618	10	27	0.0025	0.9004	A.A[114]AASAAGPGGLVAGKE[436]EK.K
E	3	8413290	42.035	8	28	0.0004	1	K.A[114]AAAASAAGPGGLVAGK[171]E[436]EK K.K
E	5	7827930	29.519	76	100	0.0329	1	R.LSRVTENYLCKPE[436]DNIYSIDFTRFK.I
E	2	109000000	54.602	8	27	-0.0004	1	K.A[114]AAAASAAGPGGLVAGKE[436]EK.K
E	3	238000000	58.116	8	27	-0.0009	1	K.A[114]AAAASAAGPGGLVAGKE[436]EK.K
E	2	9610290	25.22	16	27	-0.0033	0.9369	A.GPGGLVAGKE[436]EK.K
E	4	21800000	18.955	81	100	-0.0158	0.9891	T.ENYLCK[171]PE[436]DNIYSIDFTRFK.I
E	5	14100000	12.569	76	100	0.0002	0.9948	R.LSRVTENYLCKPE[436]DNIYSIDFTRFK.I
E	4	38800000	57.167	79	100	1.0029	1	R.VTENYLCKPE[436]DNIYSIDFTRFK.I

E	4	38800000	56.698	79	100	-0.0019	1	R.VTENYLCKPE[436]DNIYSIDFTRFK.I
E	7	110000000	19.663	144	184	-0.0356	0.9597	Q.FTPAFLRLRTVGATVEFTVGDKPVSNFRMIE[436]RHYFREHLLK.N
E	3	4582200	47.523	79	100	0.0041	1	R.V[142]TENYLCKPE[436]DNIYSIDFTRFK.I
E	2	7942380	15.201	8	27	-0.0054	0.9874	K.AAAAASAAGPGGLVAGKEE[436]K.K
E	3	22100000	34.999	12	27	0.0017	0.9999	A.ASAAGPGGLVAGKE[436]EK.K
E	2	7107760	9.398	12	27	0.0035	0.9136	A.ASAAGPGGLVAGKE[436]EK.K
E	3	198000000	39.668	11	27	0	0.9999	A.AASAAGPGGLVAGKE[436]EK.K
E	3	4450270	37.323	9	27	0.0002	0.9999	A.AAAASAAGPGGLVAGKE[436]EK.K
E	3	666000000	57.298	8	27	-0.0001	1	K.AAAAASAAGPGGLVAGKE[436]EK.K
E	2	28700000	51.187	8	27	0.004	1	K.AAAAASAAGPGGLVAGKE[436]EK.K
E	2	5720350	27.143	13	27	0.0012	0.9995	A.SAAGPGGLVAGKE[436]EK.K
E	3	27300000	54.909	8	28	0.0007	1	K.AAAAASAAGPGGLVAGKE[436]EK[171]K.K
E	2	8075370	23.747	8	25	0.0008	0.9924	K.AAAAASAAGPGGLVAGKE[436].E
E	2	13900000	41.971	11	27	0.0015	1	A.A[114]ASAAGPGGLVAGKEE[436]K.K
E	3	16100000	50.192	8	28	0.0017	1	K.A[114]AAAASAAGPGGLVAGKE[436]EKK.K
E	2	6243500	19.968	13	27	-0.0007	0.9962	A.S[130]AAGPGGLVAGKE[436]EK.K
E	2	8925710	34.961	12	27	0.0002	0.9997	A.A[114]SAAGPGGLVAGKE[436]EK.K
E	2	28300000	54.557	8	27	0.0007	1	K.A[114]AAAASAAGPGGLVAGKEE[436]K.K
E	3	42900000	47.477	8	27	-0.0007	1	K.A[114]AAAASAAGPGGLVAGKEE[436]K.K
E	2	59200000	51.26	11	27	0.0002	1	A.A[114]ASAAGPGGLVAGKE[436]EK.K
E	3	7102680	28.502	8	28	0.0039	0.9953	K.A[114]AAAASAAGPGGLVAGK[171]E[436]EKK.K
E	5	21700000	18.037	40	67	0.0202	0.9662	K.ARRQAPHHAADDGVGAAVTE[436]QE[436]LLALDT.I
E	3	215000000	58.168	8	27	-0.0004	1	K.A[114]AAAASAAGPGGLVAGKE[436]EK.K
E	2	103000000	51.846	8	27	0.0002	1	K.A[114]AAAASAAGPGGLVAGKE[436]EK.K
E	2	5313920	29.176	16	27	-0.0014	0.9934	A.GPGGLVAGKE[436]EK.K
E	4	6480320	14.428	81	100	-0.0102	0.981	T.ENYLCK[171]PE[436]DNIYSIDFTRFK.I
E	5	12700000	64.909	76	100	0.0007	1	R.LSRVTENYLCKPE[436]DNIYSIDFTRFK.I
E	4	4739720	12.484	79	100	-0.0014	0.9931	R.VTE[436]NYLCKPEDNIYSIDFTRFK.I
E	4	52500000	40.186	79	100	2.0041	1	R.VTENYLCKPE[436]DNIYSIDFTRFK.I
E	4	52500000	56.872	79	100	0.0009	1	R.VTENYLCKPE[436]DNIYSIDFTRFK.I
E	7	88200000	21.093	144	184	-0.0351	0.9913	Q.FTPAFLRLRTVGATVEFTVGDKPVSNFRMIE[436]RHYFREHLLK.N
E	3	7415840	26.494	13	27	-0.0006	0.9888	A.SAAGPGGLVAGKE[436]EK.K
E	3	5938450	44.698	8	27	-0.0011	0.9984	K.AAAAASAAGPGGLVAGKEE[436]K.K
E	3	4830100	30.077	10	27	0.0011	0.9977	A.AAASAAGPGGLVAGKE[436]EK.K
E	3	115000000	24.533	8	27	0.0007	0.9762	K.AAAAASAAGPGGLVAGKE[436]EK.K
E	3	12800000	38.127	11	28	0.0014	0.9893	A.AASAAGPGGLVAGK[171]E[436]EKK.K
E	3	9753000	46.046	8	27	0.9953	1	K.AAAAASAAGPGGLVAGKEE[436]K.K
E	3	36000000	32.788	13	27	-0.0017	0.999	A.SAAGPGGLVAGKE[436]EK.K
E	3	31400000	34.982	12	27	0.0011	0.9999	A.ASAAGPGGLVAGKE[436]EK.K
E	3	316000000	32.889	11	27	0.0003	0.9998	A.AASAAGPGGLVAGKE[436]EK.K
E	3	21300000	28.351	10	27	0.0012	0.9914	A.AAASAAGPGGLVAGKE[436]EK.K
E	3	6093630	42.001	9	27	0.0002	0.9999	A.AAAASAAGPGGLVAGKE[436]EK.K
E	3	795000000	52.928	8	27	0.0014	1	K.AAAAASAAGPGGLVAGKE[436]EK.K
E	3	35400000	49.739	8	28	0.0017	1	K.AAAAASAAGPGGLVAGKE[436]EK[171]K.K
E	3	9124580	20.004	8	27	-0.0004	0.9836	K.A[114]AAAASAAGPGGLVAGKE[436]EK.K
E	3	17600000	39.599	8	28	0.0061	0.9999	K.A[114]AAAASAAGPGGLVAGKE[436]EKK.K
E	3	1129230	17.418	8	28	-0.0114	0.96	K.A[114]AAAASAAGPGGLVAGKEE[436]K[171]K.K
E	3	7953540	38.501	8	27	-0.0039	0.9999	K.AAAAASAAGPGGLVAGK[171]EE[436]K.K
E	3	49200000	58.086	8	27	-0.0017	0.9999	K.A[114]AAAASAAGPGGLVAGKEE[436]K.K
E	3	8795400	49.583	8	28	0.0051	0.999	K.A[114]AAAASAAGPGGLVAGK[171]E[436]EKK.K
E	3	241000000	58.296	8	27	0.0012	1	K.A[114]AAAASAAGPGGLVAGKE[436]EK.K
E	3	13600000	49.784	8	28	0.0002	0.9968	K.A[114]AAAASAAGPGGLVAGKE[436]EK[171]K.K
E	4	20700000	26.131	81	100	0.9907	0.9997	T.E[436]NYLCK[171]PEDNIYSIDFTRFK.I
E	4	20000000	29.873	81	100	-0.0119	0.9999	T.ENYLCK[171]PE[436]DNIYSIDFTRFK.I
E	4	3971570	13.909	81	100	-0.0144	0.9878	T.E[436]NYLCK[171]PEDNIYSIDFTRFK.I
E	3	3538460	42.279	8	27	0.0048	0.9999	K.A[114]AAAASAAGPGGLVAGKE[436]EK[171].K
E	4	11300000	55.953	76	100	1.0014	1	R.LSRVTENYLCKPE[436]DNIYSIDFTRFK.I
E	4	11300000	50.533	76	100	0.0009	1	R.LSRVTENYLCKPE[436]DNIYSIDFTRFK.I
E	5	13800000	62.116	76	100	0.0022	1	R.LSRVTENYLCKPE[436]DNIYSIDFTRFK.I
E	3	14000000	21.218	81	100	-0.0043	0.9992	T.E[436]NYLCKPE[436]DNIYSIDFTRFK.I
E	4	64500000	56.6	79	100	0.0007	1	R.VTENYLCKPE[436]DNIYSIDFTRFK.I

E	3	5824100	57.438	79	100	-0.0002	1	R.VTENYLCKPE[436]DNIYSIDFTRFK.I
E	6	51800000	19.382	144	184	0.9643	0.9682	Q.FTPAFLRLRTVGATVEFTVGDKPVSNFRMIE[436]RHYFREHLLK.N
E	7	188000000	19.057	144	184	-0.0312	0.9942	Q.FTPAFLRLRTVGATVEFTVGDKPVSNFRMIE[436]RHYFREHLLK.N
E	4	5464960	41.351	76	100	-0.0004	1	R.L[156]SRVTENYLCKPE[436]DNIYSIDFTRFK.I
E	3	3202980	59.299	79	100	1.0002	1	R.V[142]TENYLCKPE[436]DNIYSIDFTRFK.I
E	3	3087240	61.366	79	100	-0.0022	1	R.V[142]TENYLCKPE[436]DNIYSIDFTRFK.I
E	5	1375690	26.9	198	236	-0.0483	0.9968	S.RNTCE[436]HIYE[436]FPQLSEDVIRLMIENPYETRSDSFYFVDNK.L
E	3	1349410	12.023	177	184	1.0033	0.925	H.YFRE[436]HLLK.N
E	3	8678420	26.327	13	27	-0.002	0.9846	A.SAAGPGGLVAGKEE[436]K.K
E	3	42000000	38.684	11	27	-0.0006	0.9999	A.AASAAGPGGLVAGKE[436]EK.K
E	3	5771660	44.867	8	27	-0.0057	0.9999	K.AAAAASAAGPGGLVAGKEE[436]K.K
E	3	5173500	34.341	10	27	0.0008	0.988	A.AAASAAGPGGLVAGKEE[436]K.K
E	3	106000000	30.474	8	27	-0.0006	0.9964	K.AAAAASAAGPGGLVAGKE[436]EK.K
E	3	3528000	29.142	14	27	1.0035	0.9978	S.AAGPGGLVAGKE[436]EK.K
E	3	28900000	29.537	13	27	-0.0025	0.9937	A.SAAGPGGLVAGKE[436]EK.K
E	3	32300000	34.759	12	27	0.0009	0.9989	A.ASAAGPGGLVAGKE[436]EK.K
E	3	332000000	34.967	11	27	0.0006	0.9999	A.AASAAGPGGLVAGKE[436]EK.K
E	3	21100000	29.833	10	27	0.0001	0.9888	A.AAASAAGPGGLVAGKE[436]EK.K
E	3	6799700	37.303	9	27	0.0009	0.9976	A.AAAASAAGPGGLVAGKE[436]EK.K
E	3	867000000	50.186	8	27	0.0001	1	K.AAAAASAAGPGGLVAGKE[436]EK.K
E	3	857000000	55.352	8	27	1.0013	1	K.AAAAASAAGPGGLVAGKE[436]EK.K
E	3	34100000	49.793	8	28	0.0004	1	K.AAAAASAAGPGGLVAGKE[436]EK[171]K.K
E	3	5343640	37.29	8	27	-0.0029	0.9987	K.AAAAASAAGPGGLVAGK[171]EE[436]K.K
E	3	18300000	42	8	28	0.0041	0.9995	K.A[114]AAAASAAGPGGLVAGKE[436]EKK.K
E	3	4949250	32.56	8	27	-0.0056	0.9989	K.AAAAASAAGPGGLVAGK[171]EE[436]K.K
E	3	34000000	58.136	8	27	-0.0056	1	K.A[114]AAAASAAGPGGLVAGKEE[436]K.K
E	3	9594750	49.69	8	28	0.0002	0.9999	K.A[114]AAAASAAGPGGLVAGK[171]EE[436]KK.K
E	5	11400000	38.45	76	100	0.0322	1	R.LSRVTENYLCKPE[436]DNIYSIDFTRFK.I
E	3	262000000	58.283	8	27	-0.0009	1	K.A[114]AAAASAAGPGGLVAGKE[436]EK.K
E	3	13900000	47.187	8	28	-0.0017	0.9993	K.A[114]AAAASAAGPGGLVAGKE[436]EK[171]K.K
E	5	8240860	17.901	78	100	-0.0163	0.9592	S.RVTE[436]NYLCK[171]PEDNIYSIDFTRFK.I
E	4	23800000	26.362	81	100	-0.0146	0.9924	T.ENYLCK[171]PE[436]DNIYSIDFTRFK.I
E	3	3349260	16.316	81	100	-0.0151	0.9719	T.E[436]NYLCK[171]PEDNIYSIDFTRFK.I
E	4	5758640	13.732	81	100	-0.018	0.9711	T.ENYLCK[171]PE[436]DNIYSIDFTRFK.I
E	5	13200000	61.861	76	100	-0.0004	1	R.LSRVTENYLCKPE[436]DNIYSIDFTRFK.I
E	4	13200000	66.659	76	100	-0.0041	1	R.LSRVTENYLCKPE[436]DNIYSIDFTRFK.I
E	4	3587590	29.818	79	100	0.0324	0.9998	R.V[142]TENYLCKPE[436]DNIYSIDFTRFK.I
E	3	20300000	16.901	81	100	-0.0102	0.9788	T.E[436]NYLCKPE[436]DNIYSIDFTRFK.I
E	4	57400000	55.21	79	100	-0.0031	1	R.VTENYLCKPE[436]DNIYSIDFTRFK.I
E	3	5380120	52.455	79	100	-0.0056	1	R.VTENYLCKPE[436]DNIYSIDFTRFK.I
E	6	17000000	23.164	144	184	-0.0444	0.9979	Q.FTPAFLRLRTVGATVEFTVGDKPVSNFRMIE[436]RHYFREHLLK.N
E	3	1791900	14.312	81	100	0.9865	0.912	T.E[172]NYLCK[171]PE[436]DNIYSIDFTRFK.I
E	3	2941080	15.601	81	100	2.0146	0.9843	T.E[436]NYLCK[171]PE[436]DNIYSIDFTRFK.I
E	3	2941080	15.828	81	100	1.0127	0.9918	T.E[436]NYLCK[171]PE[436]DNIYSIDFTRFK.I
E	3	2941080	15.976	81	100	-0.0058	0.9944	T.E[436]NYLCK[171]PE[436]DNIYSIDFTRFK.I
E	4	4904650	44.737	76	100	-0.0024	1	R.L[156]SRVTENYLCKPE[436]DNIYSIDFTRFK.I
E	3	2071060	63.569	79	100	0.9995	1	R.V[142]TENYLCKPE[436]DNIYSIDFTRFK.I
E	5	3397070	27.004	198	236	-0.0498	0.9996	S.RNTCE[436]HIYE[436]FPQLSEDVIRLMIENPYETRSDSFYFVDNK.L
E	4	21200000	23.373	173	184	-0.0006	0.953	M.IERHYFRE[436]HLLK.N
E	3	9213080	44.593	8	27	-0.0052	0.9999	K.AAAAASAAGPGGLVAGKEE[436]K.K
E	3	4114760	38.138	10	27	-0.0001	0.9997	A.AAASAAGPGGLVAGKEE[436]K.K
E	3	99600000	28.484	8	27	-0.002	0.9919	K.AAAAASAAGPGGLVAGKE[436]EK.K
E	3	14900000	24.91	11	28	2.0028	0.9473	A.AASAAGPGGLVAGK[171]E[436]EKK.K
E	3	15000000	29.881	11	28	-0.0012	0.9973	A.AASAAGPGGLVAGK[171]EE[436]KK.K
E	3	21700000	29.001	13	27	-0.0025	0.9941	A.SAAGPGGLVAGKE[436]EK.K
E	3	30700000	34.9	12	27	-0.0004	0.9997	A.ASAAGPGGLVAGKE[436]EK.K
E	3	15900000	32.068	10	27	-0.0008	0.9958	A.AAASAAGPGGLVAGKE[436]EK.K
E	3	5144700	32.321	9	27	-0.0004	0.9928	A.AAAASAAGPGGLVAGKE[436]EK.K
E	3	677000000	52.25	8	27	-0.0018	0.9998	K.AAAAASAAGPGGLVAGKE[436]EK.K
E	3	27900000	55.406	8	28	-0.0034	0.9998	K.AAAAASAAGPGGLVAGKE[436]EK[171]K.K

E	3	4452410	33.794	8	27	-0.007	0.9996	K.AAAAASAAGPGGLVAGK[171]EE[436]K.K
E	3	25200000	60.875	8	27	-0.0095	0.9999	K.A[114]AAAASAAGPGGLVAGKEE[436]K.K
E	3	1650240	27.949	11	28	0.0097	0.966	A.A[114]ASAAGPGGLVAGKE[436]EK[171]K.K
E	3	8199530	47.123	8	28	0.0004	0.9999	K.A[114]AAAASAAGPGGLVAGK[171]EE[436]K K.K
E	5	7779120	25.749	76	100	0.028	0.9995	R.LSRVTENYLCKPE[436]DNIYSIDFTRFK.I
E	3	214000000	58.307	8	27	-0.0017	1	K.A[114]AAAASAAGPGGLVAGKE[436]EK.K
E	3	17600000	44.503	8	28	-0.0022	0.9995	K.A[114]AAAASAAGPGGLVAGKE[436]EK[171] K.K
E	4	4611550	16.728	81	100	0.0449	0.9254	T.E[436]NYLCK[171]PE[436]DNIYSIDFTRFK.I
E	4	3906070	15.796	81	100	-0.0166	0.9561	T.E[436]NYLCK[171]PEDNIYSIDFTRFK.I
E	3	2947260	37.409	8	27	0.0036	0.999	K.A[114]AAAASAAGPGGLVAGKE[436]EK[171] .K
E	5	11800000	59.07	76	100	0.0004	1	R.LSRVTENYLCKPE[436]DNIYSIDFTRFK.I
E	4	9839600	53.668	76	100	0.0004	1	R.LSRVTENYLCKPE[436]DNIYSIDFTRFK.I
E	4	4312660	26.078	79	100	1.0002	0.9997	R.VTE[436]NYLCKPEDNIYSIDFTRFK.I
E	3	5164820	21.476	81	100	-0.0068	0.9941	T.E[436]NYLCKPE[436]DNIYSIDFTRFK.I
E	4	47000000	35.725	79	100	0.0004	1	R.VTENYLCKPE[436]DNIYSIDFTRFK.I
E	3	3834960	39.818	79	100	0.0026	1	R.VTENYLCKPE[436]DNIYSIDFTRFK.I
E	3	3834960	52.546	79	100	1.0058	1	R.VTENYLCKPE[436]DNIYSIDFTRFK.I
E	7	62300000	16.795	144	184	-0.0341	0.9827	Q.FTPAFLRLRTVGATVEFTVGDKPVSNFRMIE[436]RHYFREHLLK.N
E	3	1118210	13.315	81	100	2.0151	0.9351	T.E[436]NYLCK[171]PE[436]DNIYSIDFTRFK.I
E	3	4119590	17.95	81	100	0.0131	0.9865	T.E[436]NYLCK[171]PE[436]DNIYSIDFTRFK.I
E	4	4080490	46.506	76	100	0.0012	1	R.L[156]SRVTENYLCKPE[436]DNIYSIDFTRFK. I
D	3	32600000	30.772	237	251	0.0004	0.9982	K.LIMHNKAD[422]YAYNGGQ.L
D	2	8423590	29.974	237	251	-0.0079	0.9986	K.L[156]IMHNKAD[422]YAYNGGQ.L
D	3	60700000	34.913	237	251	0.0012	0.9997	K.LIMHNKAD[422]YAYNGGQ.L
D	2	17300000	32.024	237	251	-0.0063	0.9988	K.L[156]IMHNKAD[422]YAYNGGQ.L
D	3	59100000	36.611	237	251	-0.0003	0.9982	K.LIMHNKAD[422]YAYNGGQ.L
D	4	10800000	25.807	78	100	-0.0014	1	S.RVTENYLCKPEDNIYSID[422]FTRFK.I
D	2	19000000	30.039	237	251	-0.0078	0.9985	K.L[156]IMHNKAD[422]YAYNGGQ.L
D	3	4015900	26.432	237	251	0.0024	0.9442	K.LIM[147]HNKAD[422]YAYNGGQ.L
D	3	150000000	30.843	237	251	-0.0014	0.9488	K.LIMHNKAD[422]YAYNGGQ.L
D	7	12200000	13.63	148	184	0.9765	0.9198	A.FLRLRTVGATVEFTVGD[422]KPVSNFRMIER HYFREHLLK.N
D	7	2740460	24.114	40	78	-0.0043	1	K.ARRQAPHHAAD[422]DGVGAAVTEQELLAL DTIRPEHVLRLSR.V
D	6	3579110	19.097	153	184	1.0065	0.9752	R.TVGATVEFTVGD[422]KPVSNFRMIERHYFR EHLLK.N
D	6	56100000	17.683	144	184	-0.0385	0.9729	Q.FTPAFLRLRTVGATVEFTVGD[422]KPVSNFR MIERHYFREHLLK.N
D	4	1268680	33.633	79	100	1.0024	0.9998	R.VTENYLCKPEDNIYSID[422]FTRFK.I
D	4	1268680	29.644	79	100	-0.0029	0.9999	R.VTENYLCKPEDNIYSID[422]FTRFK.I
D	3	174000000	33.168	237	251	-0.0031	0.9954	K.LIMHNKAD[422]YAYNGGQ.L
D	4	1135630	24.94	79	100	-0.0031	0.9868	R.VTENYLCKPEDNIYSID[422]FTRFK.I
D	3	2020670	23.46	237	251	0.0004	0.9013	K.LIM[147]HNKAD[422]YAYNGGQ.L
D	3	148000000	32.706	237	251	-0.0034	0.9663	K.LIMHNKAD[422]YAYNGGQ.L
D	3	1332140	15.07	232	242	-0.0007	0.9184	Y.FVD[422]NKLIMHNK.A
D	7	1745970	20.023	40	78	-0.0087	0.9995	K.ARRQAPHHAAD[422]DGVGAAVTEQELLAL DTIRPEHVLRLSR.V
D	3	4136130	29.084	237	251	-0.0031	0.9901	K.L[156]IMHNKAD[422]YAYNGGQ.L
D	6	2399460	16.94	153	184	-0.0041	0.9343	R.TVGATVEFTVGD[422]KPVSNFRMIERHYFR EHLLK.N

PSM’s identified by MSFragger search of compound **7** covalently modified UNC119B peptides. Purified UNC119B protein was incubated overnight at 37°C with compound **7** (10 eq.) in triplicate. After digestion with LysC and sample workup samples were analyzed by two different LC/MS methods. Recorded RAW spectra were searched in MSFragger. The identified PSM data was combined in one file and then filtered for compound 7 modified UNC119B peptides with intensity above zero and peptide probability bigger 0.9. (See method section for details about LC/MS).

Supplementary Table 5. X-ray crystallography data collection and refinement statistics.

Statistics for the highest resolution shell are shown in parentheses.

**PDB ID**	**9HMC**	**9HMD**
Crystal and ligand	**PDEδ•5e**	**PDEδ•6a**
**Data collection**		
Space group	P 21 21 21	P 21 21 21
Unit cell dimensions		
a, *b*, *c* (Å)	*a* = 56.74, *b* = 75.65, *c* = 82.31	*a* = 56.47, *b* = 75.22, *c* = 81.6
α, β, γ (°)	α =90, β =90, γ = 90	α =90, β =90, γ = 90
Wavelength	1.00	1.542
Resolution range (Å)	46.72 - 1.65 (1.69 - 1.65)	19.98 - 2.5 (2.75 - 2.5)
Total reflections	579371 (38449)	86719 (21274)
Unique reflections	82349 (5485)	12453 (3026)
R-meas	0.08715 (2.333)	0.445 (1.813)
R-pim	0.03273 (0.8782)	0.1667 (0.6783)
R-merge	0.08073 (2.16)	0.4119 (1.679)
$CC_{1/2}$	0.999 (0.213)	0.967 (0.466)
Mean I/σ (I)	12.91 (0.89)	4.83 (1.17)
Completeness (%)	99.98 (99.89)	99.54 (99.44)
Redundancy	7.0 (7.0)	7.0 (7.0)
**Refinement**		
Resolution (Å)	1.65	2.5
Reflections used in refinement	43329 (2841)	12451 (3025)
Reflections used for *R*-free	2167 (142)	623 (151)
R-work	0.1880 (0.3474)	0.2394 (0.3198)
R-free	0.2234 (0.3464)	0.2829 (0.3931)
Protein residues	297	292
Number of non-H atoms	2704	2491
macromolecules	2456	2395
ligands	101	88
solvent	147	8
Wilson B-factor	25.74	28.12
Average B-factor	33.17	31.46
macromolecules	32.85	31.47
ligands	38.39	32.34
solvent	34.86	20.38
Root mean square deviation		
RMS (bonds) (Å)	0.004	0.002
RMS (angles) (°)	0.77	0.55
Ramachandran favoured (%)	97.94	97.55
Ramachandran allowed (%)	2.06	2.45
Ramachandran outliers (%)	0.00	0.00
Rotamer outliers (%)	1.84	0.00
Clashscore	2.14	2.62

2. Methods

Mass spectrometry analysis of UNC119B adducts after Lys-C digestion

Samples of UNC119B (200 µg protein per sample), either treated with compound **7** or vehicle DMSO, were processed by denaturation, reduction, alkylation, and digestion with rLys-C (Promega V1671), followed by desalting prior to mass spectrometry analysis. The experiment was conducted with three biological replicates. Mass spectrometry experiments were carried out using an Orbitrap Fusion LUMOS instrument (Thermo) paired with an Vanquish Neo ultra-performance liquid chromatography (UPLC) system (Thermo). The UPLC system was configured in one-column mode, with the analytical column consisted of a fused silica capillary (75 µm × 28 cm) equipped with an integrated fritted emitter (CoAnn Technologies), packed in-house with 1.7 µm Kinetex core-shell beads (Phenomenex). The analytical column was housed within a column oven (Sonation PRSO-V2), which maintained at 50 °C throughout both sample loading and data acquisition, and connected to a nanospray flex ion source (Thermo). The LC system utilised two mobile phases: solvent A (0.2% FA, 2% ACN, 97.8% H_2O) and solvent B (0.2% FA, 80% ACN, 19.8% H_2O), all solvents of UPLC grade from Honeywell. Peptide samples were loaded directly onto the analytical column at a maximum flow rate, typically ranging between 0.4 and 0.6 µL/min, so that the pressure would not surpass the set limit of 980 bar. Following loading, samples were separated on the analytical column using a 60- or 67-minute gradient of solvent A and B (refer to the LC settings table for specifics) at a flow rate of 250 nl/min. The mass spectrometer operates with a Orbitrap Fusion Lumos Tune Application (version 4.1.4244) and Xcalibur software (version 4.7.69.37). The modified peptides were analysed at two different concentrations (5000 ng or 4000 ng/2000 ng for the first replicate) and using two distinct separation methods (60-minute or 67-minute solvent gradients, respectively).

LC Settings

MS device	Orbitrap Fusion Lumos
LC device	Thermo Vanquish Neo
ion source	Thermo Nanospray Flex
**Analytical column**	Self-packed fused silica capillary with an integrated sintered frit; CoAnn Technologies ICT36007515F-50-5
column diameter	Length (L_C) = 28 cm; ID = 75µm; OD = 360 µm; emitter 15 µm
stationary phase	Phenomenex Kinetex C18-XB core shell
particle diameter (d_p)	1.7 µm (core shell)
Pore size	100 Å
Column ID	AC167
Column oven	Sonation column oven PRSO-V2
Column oven temp.	50°C
**solvents**	A: 0.2% FA, 2% ACN, 98% H_2O;<br>B: 0.2% FA, 80% ACN, 20 % H_2O
gradient	67min: 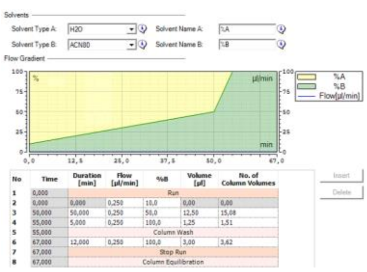 60min: 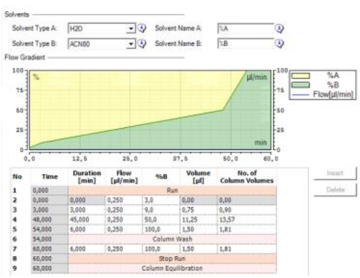

MS Settings

MS	general	MS1	MS2	Comments; special settings
Lumos	Tune v4.1.4244<br>Xcalibur v4.7.69.37<br>SII: 1.7.0.468<br>Gradient: 60 min	Analyzer: FT<br>Res.: 120000<br>SR: 375 - 1500<br>AGC: Standard<br>AGC abs.: 400000<br>AcT: 50 ms<br>RF: 30<br>SF: --<br>DDM: CT/3sec	Analyzer: FT<br>Res./ScR: 30000/-<br>SR: Auto<br>AGC: 300%<br>AGC abs.: 150000<br>AcT: auto<br>CS: +2 to +7<br>IsM: Q<br>IsW: 1.6<br>Frag.: sHCD<br>NCE: 25, 32, 45	classic orbitrap experiment: MS1 in Orbitrap at high resolution and data dependent MS2 also in Orbitrap high resolution. Dynamic exclusion enabled (exclude after n times=1; Exclusion duration (s)= 30; mass tolerance ± 10ppm)<br><br>Intensity Threshold: 25000<br>Ion transfer Tube Temp: 270 °C<br>Ion Source Voltage: 2300 V
Lumos	Tune v4.1.4244<br>Xcalibur v4.7.69.37<br>SII: 1.7.0.468<br>Gradient: 67 min	Analyzer: FT<br>Res.: 120000<br>SR: 380 - 1400<br>AGC: Standard<br>AGC abs.: 400000<br>AcT: auto<br>RF: 30<br>SF: --<br>DDM: CT/3sec	Analyzer: FT<br>Res./ScR: 30000/-<br>SR: Auto<br>AGC: 300%<br>AGC abs.: 150000<br>AcT: 70 ms<br>CS: +3 to +8<br>IsM: Q<br>IsW: 1.6<br>Frag.: sHCD<br>NCE: 25, 30, 40	classic orbitrap experiment: MS1 in Orbitrap at high resolution and data dependent MS2 also in Orbitrap high resolution. Dynamic exclusion enabled (exclude after n times=1; Exclusion duration (s)= 30; mass tolerance ± 10ppm)<br><br>Intensity Threshold: 50000<br>Ion transfer Tube Temp: 270 °C<br>Ion Source Voltage: 2300 V

Note: **FT**= Fourier Transform (Orbitrap); **IT**= Iontrap; **Q**= Quadrupol; **Res.**= max. Resolution at 200 m/z (Lumos) or 400 m/z (Elite) [FWHM (full width at half maximum)]; **ScR**= scan rate for measurements in the IT; **SR**= scan range [m/z]; **AGC**= automatic gain control, max number of acquired ions per measurement; **AcT**= max. Ion acquisition time [ms]; **CS**= charge states used for fragmentation; **IsM**= Isolation mode (Q or IT), MS2 isolation and further is only done in IT; **IsW**= Isolation window [m/z], value followed by scan mode the isolation is based on (MS1, MS2 …) **Frag.**= Fragmentation method; **HCD**= Higher-energy collisional dissociation; **CID**= Collision-induced dissociation; **ETD**= Electron-transfer dissociation**; EThcD=** Electron-Transfer/Higher-Energy Collision Dissociation; **sHCD**= stepped HCD**; NCE**= normalized collision energy; **cycles**: number of MSn recorded or max cycle time; RF= RF Lens [%]; **SF**= Source Fragmentation [V]; **DDM**: Data dependent Mode (cycle time in seconds, CT/[s] or number of scans, NS); **NS**= Number of data dependent scans.

RAW spectra were submitted to a closed MSFragger (version 4.1.)[15] search in Fragpipe (version 22)[16] using the "LFQ-MBR" workflow (label-free quantification and match-between-runs; default settings were used unless otherwise stated). RAW files were listed in the "Input LC-MS Files" section and experiment set "by file name". As "Data Type" we kept the default "DDA" (data dependent acquisition). The MS/MS spectra were searched against a custom database generated in Fragpipe (2025-08-20-decoys-contam-UP000000625_83333_plus_SOI_v01.fasta.fas (9040 entries)) containing the Uniprot *E. coli* reference proteome (UP000000625_83333.fasta; 4402 entries), the sequence of interest (UNC119B-6xHis), contaminants and decoys (the last two appended and generated by Fragpipe in the

Database section). MSFragger searches allowed oxidation of methionine residues (16 Da; 3) and acetylation of the protein N-terminus (42 Da; 1), Carbamylation at Lysin (43 Da; 3) and peptide N-terminus (43 Da; 1) and modification of glutamic acids and aspartic acids by compound **7** (307 Da; 2) as variable modification (first value in brackets refers to the molecular weight of the modification, second value to the maximum number of occurrences per peptide). A maximum of three variable modifications and a maximum of 5 combinations were allowed globally. Carbamidomethylation on Cysteine (57 Da) was selected as static modification. Enzyme specificity was set to “LysC-p SEMI”. The initial precursor and fragment mass tolerance was kept at ± 20 ppm. Mass calibration and parameter optimization was selected. Validation of peptide spectrum matches was done using MSBooster[17] using DIA-NN[18] for RT and spectra prediction. Peptide spectra matches (PSM) were validated using percolator with a minimum probability setting of 0.5. Protein inference was performed using ProteinProphet (part of Philosopher version 5.1.1).[19] The final reported protein FDR was 0.01 (based on target-decoy approach). Protein quantification was performed with IonQuant (version 1.10.27). Add MaxLFQ (min ions 1), MBR (FDR 0.01) and normalization of intensity across runs was selected. Unique and razor peptides were allowed. Advanced options were kept at default. Further analysis and filtering of the results was done in Perseus v1.6.10.0.[20] Comparison of protein group quantities (relative quantification) between different MS runs is based solely on the LFQs as calculated by IonQuant, MaxLFQ algorithm.

In-lysate cellular thermal shift assay (CETSA) and thermal proteome profiling (TPP)

<u>*General information*</u>

Three biological replicates employing lysates from Jurkat cells were measured in which compound **6a** was used at a concentration of 10 μM and alternatively with the vehicle DMSO (0.1% v/v) treated samples as controls. The samples were split into 9 aliquots each and incubated at 9 different temperatures between 37 and 67 °C. The precipitated fraction of the proteins was centrifuged off and the soluble fraction quantified relatively to the lowest temperature using a mass spectrometry-based approach with TMTpro 18-plex label (Thermo Fisher Scientific, A52047), i.e., thermal protein profiling (TPP).

In total, we identified 3903 proteins, of which 3470 were identified in all three replicates and 3048 were identified with at least two unique peptides in all replicates (compound treated and DMSO controls). 2265 of these proteins showed a normal melting curve in the DMSO control, i.e., they had relative intensities of the labels of the highest temperature smaller or equal to 35% of the labels of the lowest temperatures.

Proteins were considered as stabilised or destabilised when, in all three replicates, 1) they had a significant change of area under the fitted melting curves between DMSO control and DMSO treated conditions, ΔAUC of at least ± 1.5 with p-value ≤ 0.05 (-$\log_{10}$ p-value ≥ 1.3), or 2) they had a shift in melting points (ΔT_m) of at least ± 2 °C (same direction), or 3) they showed a difference in the relative peak intensities ($\Delta I_{67°C}$) of at least 10 % for the highest temperature compared to the DMSO control.

Out of the 3903 proteins, we identified 19 potential hits including PDEδ according to the definition above (Supplementary Information, Supplementary Table 3). PDEδ showed a typical melting behaviour only in the DMSO controls. In compound treated samples, the percentage of soluble protein at high temperatures (T > 57 °C) was much higher than in DMSO controls (see Fig. 5a), i.e., the stabilisation of PDEδ by **6a** leads to an incomplete precipitation of the protein at higher temperatures, which was only observed with PDEδ among the hits. The incomplete precipitation of compound stabilised PDEδ caused inaccuracy in the calculation of ΔT_m and western blot-based analysis of the same samples validated this melting behaviour changes in PDEδ and the ΔT_m (11.8 ± 0.2 °C) from the immunoblot readout was taken instead. PDEδ was identified as the only significant hit ($p \leq 0.05$) that showed target stabilisation with the largest change in area under the curves (ΔAUC) between DMSO control and compound treated conditions (ΔAUC = 3.5, -log (p-value) = 2.1, Fig. 5a) and highest degree of melting shift (ΔT_m =11.8 ± 0.2 °C).

Cultivation and harvesting of cells, cell lysis

Jurkat cells were grown to a density of 1.0 - 2.5 × 10^6 cells/ml. A 100 ml aliquot of the suspension was divided equally into two Falcon tubes and chilled on ice for 2 minutes. Cells were pelleted by centrifugation at 350 x g for 3 minutes at room temperature. After discarding the supernatant, the combined pellet was resuspended in 25 ml of ice-cold PBS buffer. The washing step—centrifugation at 350 x g for 2 minutes at room temperature followed by supernatant removal—was further repeated twice. Following the final wash, the cell pellet was resuspended in 1.5 ml of PBS containing 0.4% (v/v) NP40 alternative and flash-frozen in liquid nitrogen. Lysis was achieved through five to six iterative freeze-thaw cycles: frozen samples were thawed at 23 °C in a thermomixer (Thermomixer Comfort, Eppendorf) until 60-80% liquefied, then held on ice until fully thawed, before being rapidly refrozen in liquid nitrogen. The resulting lysate was separated by ultracentrifugation at 100,000 x g for 20 minutes at 4 °C (Beckman Coulter Optima MAX-XP, MLA-80 rotor). The supernatant was carefully collected, snap-frozen in liquid nitrogen, and stored at -80 °C for subsequent analysis.

In-lysate CETSA

Protein concentration was quantified using the Bradford protein assay. Samples were diluted with PBS to a final concentration of 2.5 mg/ml and split into two aliquots of equal volume. One aliquot was treated with 10 µM compound **6a** (achieved by adding 1.7 µl of a 10 mM DMSO stock to 1.7 ml sample, yielding 0.1% DMSO final concentration), while the control aliquot received an equivalent volume of DMSO alone. Both aliquots were incubated at 37 °C for either 15 minutes or 2 hours. Following incubation, each aliquot was further subdivided into nine 120 µl portions in PCR tubes. These portions underwent thermal denaturation for 5 minutes at precisely defined temperatures (37.0, 41.7, 45.8, 49.7, 53.5, 57.4, 61.3, 64.3, 67.0 °C) using a MasterCycler EpGradient S thermal cycler (Eppendorf SE, DE). Samples were then cooled to room temperature and centrifuged at 100,000 x g for 20 minutes at 4 °C (Beckman Coulter Optima MAX-XP, TLA-120.1 rotor). The supernatant from each sample was carefully collected into low protein-binding tubes, snap-frozen in liquid nitrogen and kept at -80 °C for storage. For analysis, 75 µl of each supernatant was allocated for mass spectrometry-based thermal protein profiling (TPP), while the remaining 25 µl was used for immunoblotting to assess PDEδ levels. Western blot band intensities were quantified using Image Lab software and normalised to the intensity of the first band thermal denatured at the 45.8 °C condition.

Mass spectrometry-based readout of CETSA

For the mass spectrometry-based readout samples were reduced, alkylated, precipitated with acetone and tryptic digested. After that, each aliquot was labelled using TMT reagents according to the temperature used during the thermal shift procedure. For reduction, to each 75 µl aliquot resulting from the thermal shift procedure 7.5 µl of 200 mM TCEP (tris(2-carboxyethyl) phosphine) solution (prepared from 140 µl of 0.5 M TCEP, 140 µl H_2O and 70 µl of 1M TEAB buffer - triethylammonium bicarbonate buffer, pH = 8.5, Sigma-Aldrich, #18587, cas 15715-58-9) were added, mixed by inversion, shortly centrifuged at 10,000 g and incubated at 55 °C for 1 h in a thermo block (Thermomixer Comfort, Eppendorf). To alkylate, 7.5 µl of freshly prepared 375 mM iodoacetamide solution in 200 mM TEAB buffer (26 mg iodoacetamide dissolved in 300 µl H_2O and 75 µl of 1M TEAB buffer) were added to each sample and incubated for 30 minutes in the dark at room temperature. For precipitation of proteins, six volumes (900 µl) of pre-chilled (-20 °C) acetone were added and incubated at -20 °C overnight. Afterwards, samples were centrifuged for 10 min at 8000 g at 4 °C and the supernatants were disposed while keeping the samples cold. The pellets were dried for about 30 to 45 min at room temperature. For tryptic digestion, the dried pellets were re-suspended in 107.5 µl of trypsin (proteomics grade, Sigma-Aldrich, #03708969001) solution (165 µl of 0.4 µg/ml trypsin solution (100 µg trypsin dissolved in 250 µl of 10 mM HCl) diluted in 2200 µl of freshly prepared 100 mM TEAB buffer). Samples were vigorously vortexed for about 20 s and shortly centrifuged to gather the suspension at the bottom to the

tube, followed by incubation at 37 °C for 2 - 3 hours. Vortexing and centrifugation were repeated and samples were incubated at 37 °C overnight.

For TMT labelling at the next day, samples were spinned down and 100 µl of the resulting supernatant from each sample was transferred into a new tube and labelled with TMTpro 18-plex label (Thermo Fisher Scientific, A52047). Briefly, 20 µl of anhydrous acetonitrile (Sigma-Aldrich, #271004) was added to each 0.5 mg of TMT label reagent, which was equilibrated to room temperature in a foil pouch immediately before use. 10 µl of the respective TMT reagent solution was added to each 100 µl sample. Directly after addition, samples were briefly vortexed. Samples were incubated for 2 h at room temperature, protected from light. Afterwards, 5 µl of 5% hydroxylamine solution (50 µl of the 50% hydroxylamine in 450 µl of 100 mM TEAB buffer) was added to each sample and samples were incubated for 15 min to quench the reaction. After that, equal volume of each labelled aliquot was combined into one sample. Solvent was evaporated in a SpeedVac at 30 °C until a dry white pellet was obtained.

Prior to nanoHPLC-MS/MS analysis, samples were fractionated into 8 fractions with a benchtop Pierce high pH reversed-phased peptide fractionation kit (Thermo Fisher Scientific, #84868) according to the manufacturer's protocol to reduce the complexity of the samples, and thereby increasing the number of quantified proteins. In brief, the spin column was conditioned by removing the pack solution and washing twice with 300 µl acetonitrile followed by washing twice with 300 µl of H_2O with 0.1% TFA, each time with centrifugation at 5000 g for 2 min to discard the liquid. Approximately 50 µg of the dried pellet resulting from TMT labelling was dissolved in 300 µl of 0.1% TFA solution and loaded onto one spin column for separation. The flow-through and two wash fractions (300 µl H_2O, followed by 300 µl of 5% acetonitrile, 0.1% triethylamine) were discarded before collection of 8 factions of 300 µl each in increasing percentage of acetonitrile (10.0, 12.5, 15.0, 17.5, 20.0, 22.5, 25.0, 50.0%) in 0.1% triethylamine solution, each time with centrifugation at 3000 g for 2 min to collect the liquid. Each fraction was dried in a SpeedVac at 30 °C until complete dryness and subsequently subjected to nanoHPLC-MS/MS analysis.

For nanoHPLC-MS/MS analysis, samples were dissolved in 10 µl of 0.1% TFA in water and 7 µl each were loaded onto a pre-column cartridge for desalting. Desalting was performed using 0.1% TFA as eluent for 5 min with flow to waste followed by back-flushing of the sample during the whole analysis from the pre-column to the PepMap100 RSLC C18 nano-HPLC column (2 µm, 100 Å, 75 µm ID × 50 cm, nanoViper, Dionex, Germany) using a linear gradient starting with 95% solvent A (H_2O with 0.1% formic acid)/5% solvent B (acetonitrile with 0.1% formic acid) and increasing to 28% solvent B

after 105 min using a flow rate of 300 nl/min. The nano-HPLC was online coupled to a Q-Exactive HFOrbitrap Mass Spectrometer using an uncoated SilicaTip (ID 20 µm, Tip-ID 10 µM). Mass range of m/z 375 to 1500 was acquired with a resolution of 120000 for full scan, followed by up to fifteen high energy collision dissociation (HCD) MS/MS scans of the most intense at least doubly charged ions using a resolution of 60000 and a NCE energy of 32%.

Data evaluation was performed using MaxQuant software[21] (v.2.4.14.0) including the Andromeda search algorithm and searching the human reference proteome of the Uniprot database. The search was performed for full enzymatic trypsin cleavages allowing two miscleavages. For protein modifications, carbamidomethylation was chosen as fixed and methionine oxidation and acetylation of the N-terminus were chosen as variable modifications. For relative quantification, the type "reporter ion MS2" was chosen. For lysine residues and peptide N-termini 18plex TMTpro labels were defined. The mass accuracy for full mass spectra was set to 20 ppm (first search) and 4.5 ppm (second search). The mass accuracy for MS/MS spectra was set to 20 ppm. False discovery rates for peptide and protein identification were set to 1 %. Only proteins for which at least two peptides were quantified were chosen for further validation. Relative quantification of proteins was carried out using the reporter ion MS2 algorithm implemented in MaxQuant. All experiments were performed in three biological triplicates.

Melting curves calculation

To determine the melting point shifts between **6a** and DMSO treated samples of each protein, an in-house developed Excel-Macro was used.[22] Briefly, denaturation changes at different temperatures were tracked by the reporter ion intensity and observed in relation to the lowest temperature for normalisation. The lowest temperature was set to value 1. The first and third replicates were normalised to the lowest temperature (37.0 °C) with intensities at 41.7 °C removed as outliers, and the second replicate was normalised to intensities at 41.7 °C with intensities at 37.0 °C removed as outliers for better curve fitting.

The quality of the protein identification is shown by the colour of the chart frames. A green frame indicates that the protein was identified by at least three razor and unique peptides and a reporter ion intensity of more than 1×10^5 for the first three temperatures. An orange frame is given to proteins identified by at least two razor and unique peptides and a reporter ion intensity of more than 1×10^4 for the first three temperatures. All other proteins get a red frame.

The relative fold changes were calculated as a function of temperature. The measuring points, which showed a sigmoidal trend, were fitted with the following equation using an iterative working macro for Microsoft Excel.

$$(1)\ y = bottom\, plateau + \frac{top\, plateau - bottom\, plateau}{1 + e^{-(\frac{a}{temp}) - b}}$$

The top plateau is set to one, temp is temperature, bottom plateau is a protein specific constant that defines the maximal denaturation and a and b are constants which describe the curve progression. The melting point of a protein is defined as the temperature at which half of the protein has been denatured. This point aligns with the inflection point of the curve. The inflection point shows the highest slope of the curve, which is defined as the value of the first derivation. Areas under the fitted melting curves (AUC) were calculated in GraphPad Prism 9.5.1 (GraphPad software, USA) by plotting the fitted values against respective thermal treatment temperatures and *p*-values were calculated by applying Student's paired *t*-test between DMSO treated and compound treated conditions. For hit identification, the requirements previously defined were followed.

Phosphoproteomics

NCI-H358 cells (5×10^6 cells/dish) were seeded in two 10 cm dishes and incubated in a humidified atmosphere at 37 °C and 5% CO_2 overnight. Cells were then treated with 10 µM of compound **6a** or DMSO, with 0.1% DMSO adjusted for both conditions in fresh medium for 8 h incubated in a humidified atmosphere at 37 °C and 5% CO_2. Cells were washed twice with warm PBS (phosphate buffered saline) and once with warm TBS (Tris buffered saline containing 50 mM Tris-HCl, pH = 7.5, 138 mM NaCl, 2.7 mM KCl) and lysed by incubating with boiling guanidine hydrochloride lysis buffer (1 ml/dish, freshly prepared, containing 6 M guanidine HCl, 100 mM Tris HCI, pH = 8.5, 10 mM TCEP, 40 mM CAA) at 96 °C for 5 min in a thermomixer with protection from light. The collected lysates were sonicated by four cycles of 30s sonication and 30s rest (Bandelin SONOPULS HD2070, 60% power, 70% duty cycle) and centrifuged at 16,000 g for 30 min at room temperature. The supernatants were transferred into new tubes and snap frozen by liquid nitrogen and kept at -80 °C until further usage. Upon thawing, samples were homogenised again with four cycles of sonication and protein concentration was determined by Pierce™ 660 nm protein assay according to the manufacturer's instruction (Thermo Scientific, #22662). For each sample, 1 mg of protein was digested on Sera-Mag beads (GE Healthcare, #45152105050250 and #65152105050250) with trypsin/Lys-C mix (Promega, V5072). A 20 µg/ul suspension of 1:1 mix of hydrophilic and hydrophobic Sera-Mag™ SpeedBead™ carboxylate modified magnetic particles was first prepared and added to prediluted protein samples with 1 M of guanidine HCl. Acetonitrile was added to a final 70% v/v to precipitate proteins on beads with a total of 20 min incubation and occasional mixing. Beads with aggregates of proteins were collected

with a magnetic rack, washed once with acetonitrile followed by an additional wash with 70% ethanol and dried for 5 min at room temperature. To each sample, 260 µl of digestion buffer (100 mM triethylammonium bicarbonate, cas 15715-58-9, Sigma Aldrich, T7408-100ml; 10% 2,2,2-trifluoroethanol TFE, cas 75-89-8, Fisher Scientific, #10051560) was added followed by 40 µl of trypsin/Lys-C mix (1 µg/µl in resuspension buffer containing 50 mM acetic acid, 1:25 w/w enzyme to protein ratio) and incubated at 37 °C in a thermomixer with 1000 rpm overnight. The reaction was quenched by adding 15 µl of 10% TFA to a final 0.5% v/v and the supernatant was collected in a fresh tube. Approximately 10 µg/sample was desalted by stage tip purification with C18 extraction disks (Empore^{TM} high performance extraction disks, 47 mm, 3M Bioanalytical Technologies #2215) and subsequently used for LC-MSMS analysis for global proteome profiling. The rest of the protein digest (approximately 990 µg/sample) was enriched for phosphopeptides with MagReSyn TiO_2 particles (ReSyn Biosciences, MR-TID005) with procedure adapted from the manufacturer's instruction and phosphoproteomics with the EasyPhos platform.[23] The samples were first dried in SpeedVac at 30 °C and resuspended in loading buffer (400 µl/sample, containing 1 M glycolic acid (cas 79-14-1, VWR, #1041060100), 80% acetonitrile, 5% TFA). Supernatants were collected by centrifugation at 10,000 g for 5 min at 4 °C and added to prepared TiO_2 particles in loading buffer (200 µl/sample, 2:1 w/w beads to peptides, prewashed with 200 µl of 70% EtOH and twice with 200 µl loading buffer), incubated for 20 min at room temperature with continuous mixing on a rolling shaker. The particles were then washed once with 200 µl loading buffer, twice with 200 µl wash buffer 1 (containing 1% TFA in 80% acetonitrile/H_2O) and twice with 200 µl wash buffer 2 (containing 0.2% TFA in 10% acetonitrile/H_2O), each time with 550 rpm gentle agitation for 2 min on a thermomixer before removal of wash buffers. Bound phosphopeptides were eluted from the microparticles by adding three times of 80 µl elution buffer (freshly prepared, 1% NH_4OH in water) and incubation for 10 min with constant gentle agitation at 550 rpm. The 80 µl elution buffer was transferred into a new tube containing 20 µl 15% TFA and three rounds of elution were pooled together for a total of 300 µl/sample. The samples were centrifuged at 10,000 g at room temperature for 10 min to remove particulate material and eluates were frozen by liquid nitrogen and kept at -80 °C for 30 min before vacuum drying (to approximately 15 µl/sample) in a SpeedVac at 30 °C. Enriched phosphopeptides were reconstituted in SDB-RPS loading buffer (1% TFA in isopropanol, to 200 µl/sample) and desalted by stage tip purification (2 stage tips/sample, 2 layers of SBD-RPS and 1 layer of C8 on top/stage tip) with SDB-RPS extraction disks (Sigma Aldrich, 66886-U) and C8 extraction disks (Sigma Aldrich, 66882-U). Each stage tip was equilibrated with 50 µl acetonitrile, 50 µl equilibration buffer (containing 30% methanol, 0.2% TFA in water) and 50 µl of wash buffer 1 (containing 0.2% TFA in water) prior to loading. Each loaded stage tip was washed with 100 µl SDB-RPS loading buffer, 100 µl of wash buffer 1 and 100 µl of wash buffer 2 (containing 0.2% TFA, 5% acetonitrile in water), each time by centrifuging the samples to near dryness with 500 g for

5 min at room temperature. Phophopeptides were eluted with freshly prepared elution buffer (containing 0.1% NH_4OH in 60% acetonitrile in water, 60 µl/stage tip, a total of 120 µl/sample) and immediately dried in a SpeedVac at 45 °C. After approximately 45 min, the samples were acidified by adding 10 µl of 0.3% TFA, 2% acetonitrile in water to each sample, mixed at 1400 rpm for 2 min, centrifuged at 2000 g for 1 min at room temperature and further dried in a SpeedVac at 45 °C.

For nanoHPLC-MS/MS analysis of non-enriched samples, samples were dissolved in 15 µl of 0.1% TFA in water and 4.5 µl were loaded onto a pre-column cartridge for desalting. Desalting was performed for 5 min using 0.1% TFA as eluent with flow to waste (30 µl/min) followed by back-flushing of the sample during the whole analysis from the pre-column to the PepMap100 RSLC C18 nano-HPLC column (2 µm, 100 Å, 75 µm ID × 50 cm, nanoViper, Dionex, Germany) using a linear gradient starting with 95% solvent A (H_2O with 0.1% formic acid)/5% solvent B (acetonitrile with 0.1% formic acid) and increasing to 20% solvent B after 115 min and further increasing to 32% solvent B after 135 min using a flow rate of 300 nl/min. The nano-HPLC was online coupled to a Q-Exactive HF Orbitrap Mass Spectrometer using an uncoated SilicaTip (ID 20 µm, Tip-ID 10 µM). Mass range of m/z 375 to 1500 was acquired with a resolution of 120,000 for full scan, followed by up to fifteen high energy collision dissociation (HCD) MS/MS scans of the most intense at least doubly charged ions using a resolution of 15,000 and a NCE energy of 27%.

For nanoHPLC-MS/MS analysis of phospho-enriched samples, samples were dissolved in 10 µl of 0.1% TFA in water and 5 µl were loaded onto a pre-column cartridge for desalting. Desalting was performed for 5 min using 0.1% TFA as eluent with flow to waste (30 µl/min) followed by back-flushing of the sample during the whole analysis from the pre-column to the PepMap100 RSLC C18 nano-HPLC column (2 µm, 100 Å, 75 µm ID × 50 cm, nanoViper, Dionex, Germany) using a linear gradient starting with 95% solvent A (H_2O with 0.1% formic acid)/5% solvent B (acetonitrile with 0.1% formic acid) and increasing to 20% solvent B after 115 min and further increasing to 32% solvent B after 135 min using a flow rate of 300 nl/min. The nano-HPLC was online coupled to a Q-Exactive HF Orbitrap Mass Spectrometer using an uncoated SilicaTip (ID 20 µm, Tip-ID 10 µM). Mass range of m/z 375 to 1500 was acquired with a resolution of 120,000 for full scan, followed by up to fifteen high energy collision dissociation (HCD) MS/MS scans of the most intense at least doubly charged ions using a resolution of 30,000 and a NCE energy of 27%.

Data evaluation was performed using MaxQuant software (v.2.4.14.0).[21] The spectra were queried against the human taxonomy of Uniprot and a contamination database with a 1% false discovery rate, utilising a decoy database to analyse the false discovery rate. For database search,

carbamidomethylation on cysteine residues was designated as a static modification. Additionally, phosphorylations on serine, threonine, and tyrosine, as well as acetylation on *N*-termini, and oxidation of methionine were set as variable modifications. Trypsin-specific cleavages allowing up to 2 missed cleavages were included in the analysis. Raw-data of non-enriched and phospho-enriched samples were searched together. For protein quantification just data of non-enriched samples were taken into account. All experiments were performed in biological triplicates. The output files from MaxQuant were further analysed by PhosphoAnalyst,[1] followed by kinase-substrate enrichment analysis using KSEA App with NetworKIN[24-26] and GO enrichment.[2-4] For phosphor-enriched samples a site probability threshold above 75 was employed for phosphosites and data were normalised to the total peptide content.

For validation of phosphoproteomics results by western blot analysis, PA-TU-8902 cells (1.5×10^5/ml) were seeded in 6-well plates (2 ml/well) in serum-starved medium (DMEM containing 2% FBS) and incubated overnight in a humidified atmosphere at 37 °C and 5% CO_2. Cells were treated with 10 µM of compound **6a** or 10 µM Deltazinone **1** or DMSO, with 0.1% DMSO adjusted for all conditions in fresh serum-starved medium for 3 h, 5 h and 8 h incubated in a humidified atmosphere at 37 °C and 5% CO_2. Prior to harvesting, cells were stimulated with 200 ng/ml of EGF (Cell Signalling, #72528) with incubation in a humidified atmosphere at 37 °C and 5% CO_2 for 5 min. Cells were then washed twice with cold PBS and lysed with RIPA buffer (50 mM Tris-HCl, pH 8.0, 150 mM NaCl, 1% NP40 alternative, 0.5% sodium deoxycholate, 0.1% SDS) with complete phosphatase inhibitor (Roche, # 4906845001) and protease inhibitor cocktail (Roche, #5892970001) for 30 min on ice. After centrifugation at 16,000 g at 4 °C, the supernatant was transferred into a new tube and the protein concentration was determined by DC protein assay (BIO-RAD, #5000116). Approximately 25 µg/sample was loaded onto SDS-PAGE gel and analysed by immunoblotting.

3. Chemical Synthesis

General information

Unless otherwise noted, all commercially available reagents were purchased from Sigma Aldrich or TCI Chemicals and were used as provided without further purifications. Solvents for chromatography were technical grade, purchased from VWR Chemicals. Analytical thin-layer chromatography (TLC) was performed on Merck silica gel aluminium plates with F-254 indicator. Compounds were visualised by irradiation with UV light, potassium permanganate staining with heating by a heat gun. Column chromatography was performed using silica gel Merck 60 (particle size 0.040-0.063 mm).

^{1}H-NMR and ^{13}C-NMR were recorded on a *Bruker DRX400* (400 MHz), *Bruker DRX500* (500 MHz), *INOVA500* (500 MHz) and *Bruker DRX700* (700 MHz) at room temperature using $CDCl_3$ or DMSO-*d6* as solvent. Data are reported in the following order: chemical shift (δ) values are reported in ppm with the solvent resonance as internal standard ($CDCl_3$: δ = 7.26 ppm for ^{1}H, δ = 77.16 ppm for ^{13}C; DMSO-*d6*: δ = 2.50 ppm for ^{1}H, δ = 39.52 ppm for ^{13}C); multiplicities are indicated br s (broadened singlet), s (singlet), d (doublet), t (triplet), q (quartet), m (multiplet); coupling constants (*J*) are given in Hertz (Hz).

HPLC-MS analyses were performed with an Agilent 1100 Series connected to a Thermo LCQ Advantage mass spectrometer using a C18 HPLC column 3 µm from Macherey Nagel. High resolution mass spectra were recorded on a *LTQ Orbitrap* mass spectrometer coupled to an *Accela HPLC*-System (HPLC column: *Hypersyl GOLD*, 50 mm x 1 mm, particle size 1.9 µm, ionisation method: electron spray ionisation).

General scheme for compound synthesis

a.

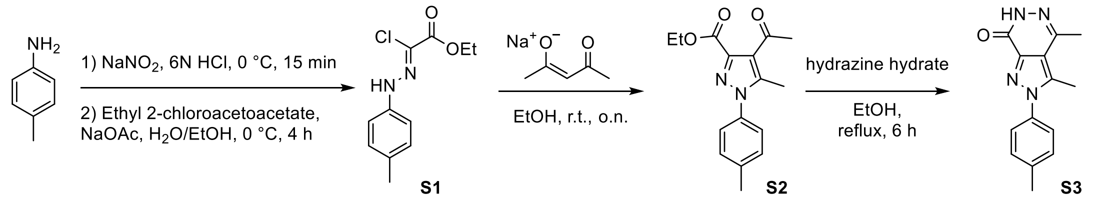

b.

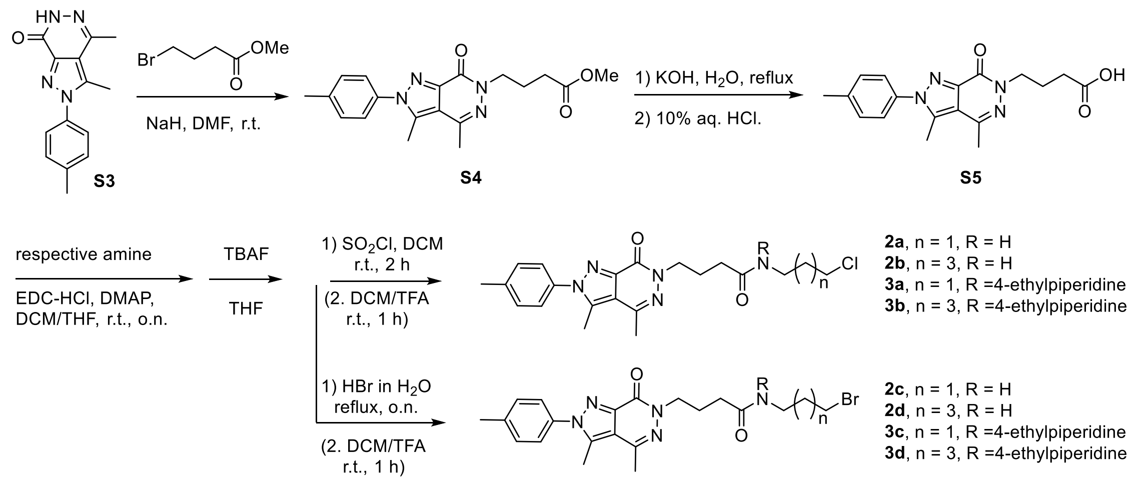

c.

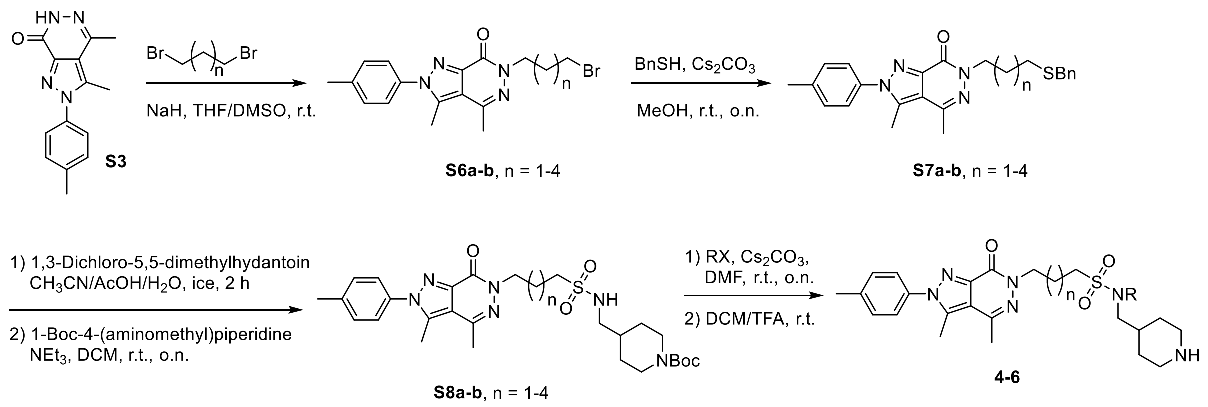

d.

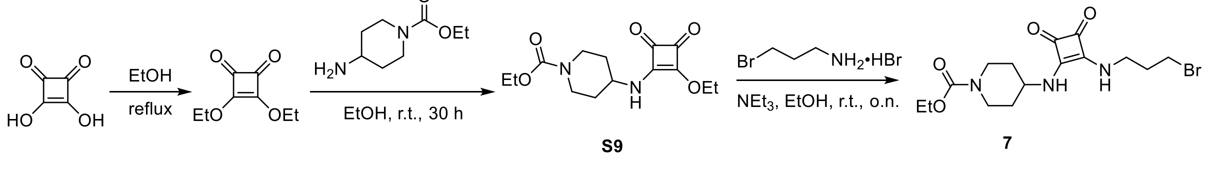

S1-5 were synthesised according to previously published methods; ^{1}H NMR, ^{13}C NMR and HRMS(ESI) of **S1-5** are identical with the reported ones.[27,28]

General procedure A for the synthesis of amides 2-3:

For synthesis of the respective amine derivatives:

To a cooled solution of the respective diol (1 equiv), TBSCl (1 equiv) and DMAP (0.2 equiv) in DCM at 0 °C was added triethylamine (1.5 equiv) and the mixture was stirred overnight at room temperature. The reaction was quenched by H_2O and extracted with DCM. The combined organic layers were dried over $MgSO_4$, filtered and concentrated *in vacuo*. The crude product was purified by flash column chromatography using 10% EtOAc/n-pentane an eluent to give the desired TBS monoprotected product. This product (1 equiv) in DCM was then added to a suspension of PCC (1.5 equiv) and celite (same weight as PCC) in DCM and the resultant mixture was stirred at room temperature. After completion, the mixture was filtered through a short pad of celite and silica gel, concentrated *in vacuo* and used for next step without any further purification.

To the respective aldehyde (1 equiv) in methanol (0.1 M) was added NH_4OAc (30 equiv) followed by $NaBH_3CN$ (2 equiv) portion wise and the resulting mixture was stirred at room temperature overnight. The reaction was quenched with addition of H_2O and methanol was removed under reduced pressure. The resulting aqueous layer was extracted with chloroform and the combined extracts were washed with brine, dried over $MgSO_4$, filtered and concentrated *in vacuo* to give the desired primary amines as yellow oils, which were used without further purification.

To the respective aldehyde (1 equiv) in DCM was added 1-Boc-4-(aminomethyl)piperidine (1.2 equiv) and the reaction was stirred at room temperature overnight. DCM was removed under reduced pressure and the resultant material was redissolved in dry methanol and cooled to 0 °C before addition of $NaBH_4$ portion wise. The reaction was then warmed to room temperature. After completion, the reaction was quenched with H_2O and extracted with EtOAc. The combined organic layer was sequentially washed with brine, dried over $MgSO_4$, filtered and concentrated *in vacuo* to give corresponding secondary amines, which were used without further purification.

For amide coupling:

To a solution of **S5** (1.0 equiv) and DMAP (1.3 equiv) in THF/DCM (1:1, 0.12 M) was added EDC-HCl (1.3 equiv), followed by the corresponding amine derivative (1.1 equiv) and the resulting solution was stirred overnight at room temperature. After completion, the reaction mixture was diluted with DCM and quenched by adding saturated $NaHCO_3$ solution. The aqueous layer was extracted with

EtOAc and combined organic layers were sequentially washed with saturated NH_4Cl solution, H_2O and brine, dried over $MgSO_4$, filtered and concentrated under vacuum. To the resulting amide in dry THF (0.03 M) was added TBAF (2.0 equiv) dropwise. The reaction was stirred at room temperature for 1 h until full deprotection and followed by the addition of H_2O. The mixture was extracted with EtOAc, washed with brine, dried over $MgSO_4$, filtered, concentrated *in vacuo* and used without further purification. To the resulting alcohol (1.0 equiv) in dry DCM (0.08 M) at 0 °C was added $SOCl_2$ (2.0 equiv) dropwise. The reaction was warmed to room temperature and stirred for 2 h. Then the mixture was added by TFA/DCM (1:1, 0.1 M). After completion, the reaction was concentrated and purified by column chromatography to give the desired chloro-substituted products, **2a-b** and **3a-b**. Alternatively, the TBS deprotected alcohol was dissolved in HBr in water (17 equiv) and the reaction was reflux overnight. After completion, the reaction was extracted with EtOAc and sequentially washed with H_2O, brine, dried over $MgSO_4$, filtered, concentrated *in vacuo* and further purified by column chromatography to give the desired bromo-substituted products, **2c-d** and **3c-d**.

General procedure B for the synthesis of S6-8:

To a suspension of **S3** (1.0 g, 3.93 mmol, 1.0 equiv) in THF/DMSO (20 ml/6 ml) was added portion wise NaH (0.24 g, 5.90 mmol, 1.5 equiv) and the mixture was stirred at room temperature for 20 min before addition of the respective dibromoalkane (15.7 mmol, 4.0 equiv). The resulting reaction mixture was further stirred at room temperature for approximately 30 min. After completion, the reaction was quenched with H_2O and the aqueous layer was extracted with EtOAc and the combined organic layers were sequentially washed with H_2O and brine, dried with $MgSO_4$ and concentrated *in vacuo*. The crude material was further purified by flash column chromatography using 100% EtOAc as an eluent to give the desired product **S6** as white solids.

To a solution of **S6** (2.66 mmol, 1.0 equiv) and benzyl mercaptan (0.3 ml, 3.93 mmol, 1.0 equiv) in methanol (10 ml) was added Cs_2CO_3 (1.7 g, 5.33 mmol, 2.0 equiv). The reaction was stirred overnight at room temperature. After completion, the reaction was quenched by adding H_2O and diluted with EtOAc. The aqueous layer was extracted with EtOAc and the combined organic layers were sequentially washed with H_2O, brine, dried over $MgSO_4$, filtered, and concentrated under vacuum. The crude material was further purified by flash column chromatography using 50% EtOAc/n-pentane as an eluent to give the desired product **S7**.

To an ice-cold solution of **S7** (1 g, 1.0 equiv) in $CH_3CN/AcOH/H_2O$ (35 ml/2.5 ml/1.5 ml) was added 1,3-dichloro-5,5-dimethylhydantoin (2.0 equiv) and the reaction mixture was stirred at 0 °C in ice bath for 2 h. After completion, the reaction was diluted with DCM, cooled to 0 °C and then 5% $NaHCO_3$

(10 ml) solution was added dropwise to quench the reaction. The resulting mixture was stirred slowly at 0 °C for additional 15 min before the organic layer was collected and washed with cold brine, dried over $MgSO_4$, filtered, concentrated *in vacuo* and used without further purification for the next step. To the resultant crude material in DCM was added 1-Boc-4-(aminomethyl)piperidine (1.1 equiv) and triethylamine (2.5 equiv) and the reaction mixture was stirred at room temperature overnight. After completion, the mixture was concentrated *in vacuo* and the crude material was further purified by flash column chromatography using 100% EtOAc an eluent to give the desired product **S8** as white solids.

General procedure C for the synthesis of sulphonamides 4-6:

To a solution of sulphonamide **S8** (1.0 equiv) in DMF (0.1 M) was added the respective alkyl halide (2.0 equiv) and Cs_2CO_3 (4.0 equiv). The reaction mixture was stirred overnight at room temperature. The reaction was quenched by addition of H_2O and extracted with DCM. The combined extracts were dried over $MgSO_4$, filtered and concentrated *in vacuo*. The residue was purified by flash column chromatography with 100% EtOAc as an eluent to give the desired product, which was then dissolved in TFA/DCM (1:1, 0.1 M) and stirred at room temperature under full Boc deprotection. The reaction mixture was further purified by prep-HPLC with gradient eluent 10% to 80% CH_3CN with 0.1% TFA/H_2O with 0.1% TFA to give the desired chloro- or bromo-substituted sulphonamides, **4-6**.

Synthesis of squaramide derivative 7

The advanced intermediate **S9** was synthesised according to the previously published method[12] and the intermediate was used without further characterisation. To a solution of **S9** (1 eqv.), 3-bromopropylamine hydrobromide (1.1 eqv.) in EtOH (0.5 M) was added dry triethylamine (10 eqv.) and the resulting mixture was stirred at room temperature overnight. The solvent was removed under reduced pressure and the desired product **7** was purified by column chromatography.

<u>Compound characterisation</u>

Ethyl (*Z*)-2-chloro-2-(2-(*p*-tolyl)hydrazineylidene)acetate (**S1**)

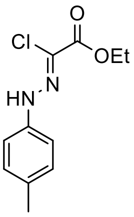

The title product compound **S1** was prepared according to previously published method from *p*-toluidine (20 mmol) as an off-white solid (4.45 g, 18.5 mmol, 92%). ^{1}H NMR, ^{13}C NMR and HRMS(ESI) are identical with the reported ones.[27,28] TLC (EtOAc/petrol 50%): Rf = 0.81. **^{1}H NMR (700 MHz, $CDCl_3$)** δ 8.32 (s, 1H), 7.13 (s, 4H), 4.38 (q, *J* = 7.2 Hz, 2H), 2.31 (s, 3H), 1.40 (t, *J* = 7.2 Hz, 3H). **^{13}C NMR (176 MHz, $CDCl_3$)** δ 159.9, 139.4, 132.8, 130.1, 115.5, 114.5, 62.8, 20.9, 14.4. **HRMS(ESI):** $[M+Na]^+$ calcd. $C_{11}H_{13}ClN_2O_2Na$ m/z 263.0558, found 263.0552.

Ethyl 4-acetyl-5-methyl-1-(*p*-tolyl)-1*H*-pyrazole-3-carboxylate (**S2**)

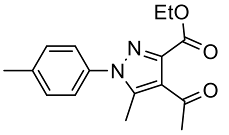

The title product compound **S2** was prepared according to previously published method from **S1** (13.0 mmol) as a yellow solid. TLC (EtOAc/petrol 50%): Rf = 0.67. **^{1}H NMR (700 MHz, DMSO-*d*$_6$)** δ 7.47 (d, *J* = 8.3 Hz, 2H), 7.42 (d, *J* = 8.3 Hz, 2H), 4.37 (q, *J* = 7.1 Hz, 2H), 2.50 (s, 3H), 2.44 (s, 3H), 2.38 (s, 3H), 1.34 (t, *J* = 7.1 Hz, 3H). **^{13}C NMR (176 MHz, DMSO-*d*$_6$)** δ 194.8, 162.5, 142.7, 142.4, 139.1, 135.4, 129.9, 125.6, 121.8, 61.3, 30.7, 20.7, 14.0, 13.8, 11.7. **HRMS(ESI):** [M+H]$^+$ calcd. $C_{16}H_{19}N_2O_3$ m/z 287.1390, found 287.1395.

3,4-Dimethyl-2-(*p*-tolyl)-2,6-dihydro-7*H*-pyrazolo[3,4-*d*]pyridazin-7-one (**S3**)

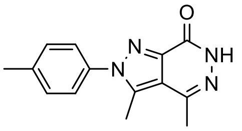

The title product compound **S3** was prepared according to previously published method from **S2** (13 mmol) as a white solid (2.31 g, 9.10 mmol, 70% over two step). TLC (100% EtOAc): Rf = 0.25. ^{1}H NMR, ^{13}C NMR and HRMS(ESI) are identical with the reported ones.[27,28] **^{1}H NMR (700 MHz, DMSO-*d*$_6$)** δ 12.00 (s, 1H), 7.49 (d, *J* = 8.2 Hz, 2H), 7.43 (d, *J* = 8.2 Hz, 2H), 2.59 (s, 3H), 2.49 (s, 3H), 2.42 (s, 3H). **^{13}C NMR (176 MHz, DMSO-*d*$_6$)** δ 156.5, 141.3, 141.1, 139.3, 137.2, 135.9, 129.9, 125.8, 117.5, 20.7, 19.3, 11.9. **HRMS(ESI):** [M+H]$^+$ calcd. $C_{14}H_{15}N_4O$ m/z 255.1240, found 255.1241.

Methyl 4-(3,4-dimethyl-7-oxo-2-(*p*-tolyl)-2,7-dihydro-6*H*-pyrazolo[3,4-*d*]pyridazin-6-yl) butanoate (**S4**)

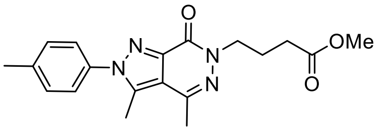

The title product compound **S4** was prepared according to previously published method from **S3** (3.69 mmol) as a white solid (1.3 g, 3.62 mmol, 98%). TLC (100% EtOAc): Rf = 0.55. ^{1}H NMR, ^{13}C NMR and HRMS(ESI) are identical with the reported ones.[27,28] **^{1}H NMR (700 MHz, CDCl$_3$)** δ 7.36 (d, *J* = 8.2 Hz, 2H), 7.33 (d, *J* = 8.2 Hz, 2H), 4.26 (t, *J* = 6.9 Hz, 2H), 3.66 (s, 3H), 2.62 (s, 3H), 2.58 (s, 3H), 2.45 (s, 3H), 2.43 (t, *J* = 8.0 Hz, 2H), 2.17 (p, *J* = 7.2 Hz, 2H). **^{13}C NMR (176 MHz, CDCl$_3$)** δ 173.7, 156.2, 142.1, 141.2, 140.0, 136.5, 136.1, 130.0, 125.9, 117.7, 51.7, 48.8, 31.4, 24.2, 21.4, 19.9, 12.5. **HRMS(ESI):** [M+H]$^+$ calcd. $C_{19}H_{23}N_4O_3$ m/z 355.1765, found 355.1773.

4-(3,4-Dimethyl-7-oxo-2-(*p*-tolyl)-2,7-dihydro-6*H*-pyrazolo[3,4-*d*]pyridazin-6-yl)butanoic acid (**S5**)

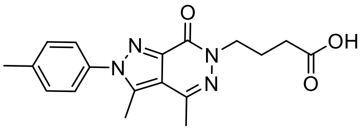

The title product compound **S5** was prepared according to previously published method from **S4** (3.39 mmol) as a white solid (1.2 g, 3.39 mmol, 100%). TLC (Acetone: petrol = 2:1): Rf = 0.35. ^{1}H NMR, ^{13}C NMR and HRMS(ESI) are identical with the reported ones.[27,28] **^{1}H NMR (700 MHz, DMSO-*d*$_6$)** δ 12.08 (br.s, 1H), 7.48 (d, *J* = 8.2 Hz, 2H), 7.43 (d, *J* = 8.2 Hz, 2H), 4.07 (t, *J* = 7.0 Hz, 2H), 2.59 (s,

3H), 2.52 (s, 3H), 2.43 (s, 3H), 2.26 (t, *J* = 7.4 Hz, 2H), 1.93 (p, *J* = 7.2 Hz, 2H).^{13}**C NMR (176 MHz, DMSO-*d*$_6$)** δ 174.0, 155.2, 141.1, 141.1, 139.4, 137.5, 135.9, 129.9, 125.7, 117.2, 48.2, 30.8, 23.8, 20.8, 19.4, 11.9. **HRMS(ESI):** [M+H]$^+$ calcd. $C_{18}H_{21}N_4O_3$ m/z 341.1608, found 341.1607.

6-(3-Bromopropyl)-3,4-dimethyl-2-(*p*-tolyl)-2,6-dihydro-7*H*-pyrazolo[3,4-*d*]pyridazin-7-one (**S6a**)

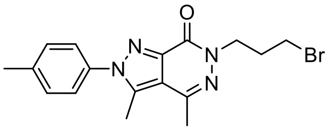

The title product compound **S6a** was prepared using general procedure B from **S3** (3.93 mmol) and 1,3-dibromopropane (1.6 ml, 15.7 mmol, 4.0 equiv) as a white solid (1.1 g, 3.04 mmol, 77%). TLC (100% EtOAc): Rf = 0.71. ^{1}**H NMR (700 MHz, DMSO-*d*$_6$)** δ 7.56 (d, *J* = 8.3 Hz, 2H), 7.48 (d, *J* = 8.1 Hz, 2H), 4.92 (t, *J* = 7.5 Hz, 2H), 4.38 (t, *J* = 7.2 Hz, 2H), 2.99 (s, 3H), 2.78 (s, 3H), 2.61 – 2.54 (m, 2H), 2.44 (s, 3H). ^{13}**C NMR (176 MHz, DMSO)** δ 151.4, 147.4, 144.9, 141.4, 140.4, 134.8, 130.2, 125.8, 116.0, 56.4, 48.0, 20.9, 20.8, 17.5, 13.0. **HRMS(APCI):** [M+H]$^+$ calcd. $C_{17}H_{20}BrN_4O$ m/z 375.0815, found 375.0785

6-(4-Bromobutyl)-3,4-dimethyl-2-(*p*-tolyl)-2,6-dihydro-7*H*-pyrazolo[3,4-*d*]pyridazin-7-one (**S6b**)

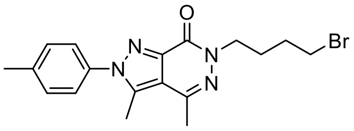

The title product compound **S6b** was prepared using general procedure B from **S3** (1.0 g, 3.93 mmol, 1.0 equiv) and 1,4-dibromobutane (2.0 ml, 15.7 mmol, 4.0 equiv) as an off-white solid (0.8 g, 3.93 mmol, 100%). TLC (100% EtOAc): Rf = 0.70. ^{1}**H NMR (500 MHz, DMSO-*d*$_6$)** δ 7.49 (d, *J* = 8.4 Hz, 2H), 7.43 (d, *J* = 8.2 Hz, 2H), 4.08 (t, *J* = 6.4 Hz, 2H), 3.58 (t, *J* = 6.2 Hz, 2H), 2.59 (s, 3H), 2.52 (s, 3H), 2.43 (s, 3H), 1.86 – 1.79 (m, 4H). ^{13}**C NMR (126 MHz, DMSO-*d*$_6$)** δ 155.2, 141.1, 141.1, 139.4, 137.5, 135.9, 129.9, 125.7, 117.1, 47.8, 34.7, 29.4, 27.0, 20.7, 19.4, 11.9. **HRMS(ESI):** [M+H]$^+$ calcd. $C_{18}H_{22}BrN_4O$ m/z 389.0972, found 389.0981.

6-(5-Bromopentyl)-3,4-dimethyl-2-(*p*-tolyl)-2,6-dihydro-7*H*-pyrazolo[3,4-*d*]pyridazin-7-one (**S6c**)

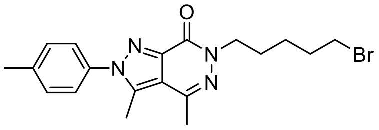

The title product compound **S6c** was prepared using general procedure B from **S3** (1.0 g, 3.93 mmol, 1.0 equiv) and 1,5-dibromopentane (2.1 ml, 15.7 mmol, 4.0 equiv) as a white solid (0.9 g, 2.99 mmol, 76%). TLC (100% EtOAc): Rf = 0.73. ^{1}**H NMR (500 MHz, DMSO-*d*$_6$)** δ 7.48 (d, *J* = 8.4 Hz, 2H), 7.42 (d, *J* = 8.2 Hz, 2H), 4.04 (t, *J* = 7.1 Hz, 2H), 3.52 (t, *J* = 6.7 Hz, 2H), 2.59 (s, 3H), 2.51 (s, 3H), 2.42 (s, 3H), 1.84 (dt, *J* = 14.4, 6.8 Hz, 2H), 1.73 (p, *J* = 7.4 Hz, 2H), 1.40 (p, *J* = 7.6 Hz, 2H). ^{13}**C NMR (126 MHz, DMSO-*d*$_6$)** δ 155.1, 141.1, 140.9, 139.3, 137.4, 135.9, 129.9, 125.7, 117.1, 48.6, 35.0, 31.9, 27.4, 24.7, 20.7, 19.4, 11.9. **HRMS(ESI):** [M+H]$^+$ calcd. $C_{19}H_{24}BrN_4O$ m/z 403.1128, found 403.1128.

6-(6-Bromohexyl)-3,4-dimethyl-2-(*p*-tolyl)-2,6-dihydro-7*H*-pyrazolo[3,4-*d*]pyridazin-7-one (**S6d**)

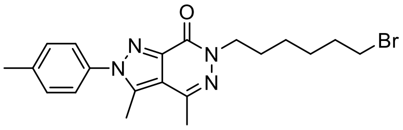

The title product compound **S6d** was prepared using general procedure B from **S3** (1.0 g, 3.93 mmol, 1.0 equiv) and 1,6-dibromohexane (2.4 ml, 15.7 mmol, 4.0 equiv) as a white solid (1.1 g, 3.30 mmol, 84%). TLC (100% EtOAc): Rf = 0.77. **^{1}H NMR (500 MHz, DMSO-*d*$_6$)** δ 7.48 (d, *J* = 8.4 Hz, 2H), 7.42 (d, *J* = 8.3 Hz, 2H), 4.03 (t, *J* = 7.2 Hz, 2H), 3.51 (t, *J* = 6.7 Hz, 2H), 2.58 (s, 3H), 2.51 (s, 3H), 2.42 (s, 3H), 1.79 (dt, *J* = 14.3, 6.8 Hz, 2H), 1.70 (p, *J* = 7.3 Hz, 2H), 1.42 (p, *J* = 7.2 Hz, 2H), 1.31 (p, *J* = 7.4, 7.0 Hz, 2H). **^{13}C NMR (126 MHz, DMSO-*d*$_6$)** δ 155.1, 141.1, 140.9, 139.3, 137.4, 135.9, 129.9, 125.7, 117.1, 48.7, 35.1, 32.2, 28.1, 27.2, 25.2, 20.7, 19.4, 11.9. **HRMS(ESI):** [M+H]$^+$ calcd. $C_{20}H_{26}BrN_4O$ m/z 417.1285, found 417.1285.

6-(3-(Benzylthio)propyl)-3,4-dimethyl-2-(*p*-tolyl)-2,6-dihydro-7*H*-pyrazolo[3,4-*d*]pyridazin-7-one (**S7a**)

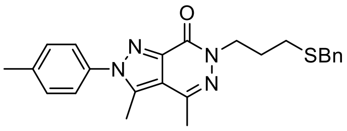

The title product compound **S7a** was prepared using general procedure B from **S6a** (1.0 g, 2.66 mmol) as a yellow oil (1.1 g, 2.61 mmol, 98%). TLC (EtOAc/petrol 40%): Rf = 0.36. **^{1}H NMR (500 MHz, CDCl$_3$)** δ 7.40 – 7.24 (m, 8H), 7.23 – 7.17 (m, 1H), 4.26 (t, *J* = 7.0 Hz, 2H), 3.73 (s, 2H), 2.63 (s, 3H), 2.56 (s, 3H), 2.51 (t, *J* = 7.8 Hz, 2H), 2.45 (s, 3H), 2.13 – 2.08 (m, 2H). **^{13}C NMR (126 MHz, CDCl$_3$)** δ 156.2, 142.1, 141.0, 139.9, 138.5, 136.3, 136.1, 130.0, 129.0, 128.5, 126.9, 125.9, 117.8, 49.0, 36.2, 28.6, 28.5, 21.4, 19.9, 12.4. **HRMS(ESI):** [M+H]$^+$ calcd. $C_{24}H_{27}N_4OS$ m/z 419.1900, found 419.1897.

6-(4-(Benzylthio)butyl)-3,4-dimethyl-2-(*p*-tolyl)-2,6-dihydro-7*H*-pyrazolo[3,4-*d*]pyridazin-7-one (**S7b**)

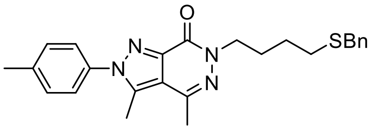

The title product compound **S7b** was prepared using general procedure B from **S6b** (1.0 g, 2.66 mmol) as a white solid (0.9 g, 2.18 mmol, 82%). TLC (EtOAc/petrol 50%): Rf = 0.30. **^{1}H NMR (600 MHz, DMSO-*d*$_6$)** δ 7.48 (d, *J* = 8.3 Hz, 2H), 7.43 (d, *J* = 8.2 Hz, 2H), 7.36 – 7.32 (m, 1H), 7.29 – 7.27 (m, 3H), 7.23 – 7.18 (m, 1H), 4.03 (t, *J* = 7.0 Hz, 2H), 3.70 (s, 2H), 2.59 (s, 3H), 2.51 (s, 3H), 2.46 – 2.40 (m, 5H), 1.75 (p, *J* = 7.2 Hz, 2H), 1.52 (p, *J* = 7.4 Hz, 2H). **^{13}C NMR (151 MHz, DMSO-*d*$_6$)** δ 155.1, 141.1, 140.9, 139.3, 138.7, 137.4, 137.3, 135.9, 129.9, 129.4, 128.7, 128.4, 128.3, 127.3, 126.7, 125.7, 117.1, 48.3, 35.0, 30.4, 27.5, 25.9, 20.7, 19.4, 11.9. **HRMS(ESI):** [M+Na]$^+$ calcd. $C_{25}H_{28}N_4OSNa$ m/z 455.1876, found 455.1878.

6-(5-(Benzylthio)pentyl)-3,4-dimethyl-2-(*p*-tolyl)-2,6-dihydro-7*H*-pyrazolo[3,4-*d*]pyridazin-7-one (**S7c**)

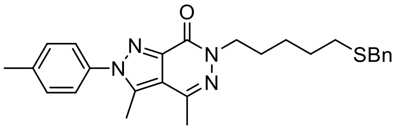

The title product compound **S7c** was prepared using general procedure B from **S6c** (1.1 g, 2.66 mmol) as a white solid (1.0 g, 2.18 mmol, 82%). TLC (EtOAc/petrol 50%): Rf = 0.34. **^{1}H NMR (600 MHz, DMSO-*d*$_6$)** δ 7.48 (d, *J* = 8.4 Hz, 2H), 7.43 (d, *J* = 8.3 Hz, 2H), 7.31 – 7.26 (m, 4H), 7.24 – 7.19 (m, 1H), 4.02 (t, *J* = 7.1 Hz, 2H), 3.70 (s, 2H), 2.59 (s, 3H), 2.51 (s, 3H), 2.43 (s, 3H), 2.37 (t, *J* = 7.3 Hz, 2H), 1.68 (p, *J* = 7.4 Hz, 2H), 1.59 – 1.51 (m, 2H), 1.32 (p, *J* = 7.6 Hz, 2H). **^{13}C NMR (151 MHz, DMSO-*d*$_6$)** δ 155.1, 141.1, 140.9, 139.3, 138.8, 137.4, 135.9, 129.9, 128.8, 128.3, 126.7, 125.7, 117.1, 48.6, 35.0, 30.5, 28.4, 27.8, 25.3, 20.7, 19.4, 11.9. **HRMS(ESI):** $[M+H]^+$ calcd. $C_{26}H_{31}N_4OS$ m/z 447.2213, found 447.2209.

6-(6-(Benzylthio)hexyl)-3,4-dimethyl-2-(*p*-tolyl)-2,6-dihydro-7*H*-pyrazolo[3,4-*d*]pyridazin-7-one (**S7d**)

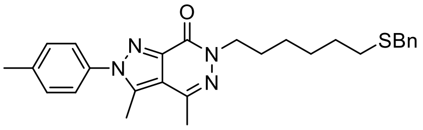

The title product compound **S7d** was prepared using general procedure B from **S6d** (1.1 g, 2.66 mmol) as a white solid (1.2 g, 2.63 mmol, 99%). TLC (EtOAc/petrol 50%): Rf = 0.43. **^{1}H NMR (600 MHz, DMSO-*d*$_6$)** δ 7.49 (d, *J* = 8.3 Hz, 2H), 7.44 (d, *J* = 8.3 Hz, 2H), 7.39 – 7.27 (m, 4H), 7.25 – 7.20 (m, 1H), 4.02 (t, *J* = 7.2 Hz, 2H), 3.71 (s, 2H), 2.59 (s, 3H), 2.52 (s, 3H), 2.43 (s, 3H), 2.37 (t, *J* = 7.3 Hz, 2H), 1.68 (p, *J* = 7.4 Hz, 2H), 1.49 (p, *J* = 7.3 Hz, 2H), 1.34 (dt, *J* = 14.8, 6.9 Hz, 2H), 1.26 (p, *J* = 7.2, 6.8 Hz, 2H). **^{13}C NMR (151 MHz, DMSO-*d*$_6$)** δ 155.1, 141.1, 140.9, 139.3, 138.8, 137.4, 135.9, 129.9, 128.8, 128.3, 126.7, 125.7, 117.1, 48.7, 35.0, 30.5, 28.6, 28.1, 27.9, 25.6, 20.7, 19.4, 11.9. **HRMS(ESI):** $[M+Na]^+$ calcd. $C_{27}H_{32}N_4OSNa$ m/z 483.2189, found 483.2180.

***tert*-Butyl 4-(((3-(3,4-dimethyl-7-oxo-2-(*p*-tolyl)-2,7-dihydro-6*H*-pyrazolo[3,4-*d*]pyridazin-6-yl) propyl)sulphonamido)methyl)piperidine-1-carboxylate** (**S8a**)

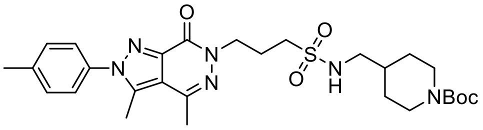

The title product compound **S8a** was prepared using general procedure B from **S7a** (1.0 g, 2.39 mmol) as a yellow solid (0.77 g, 1.34 mmol, 56%). TLC (100% EtOAc): Rf = 0.32. **^{1}H NMR (500 MHz, CDCl$_3$)** δ 7.36 (d, *J* = 8.6 Hz, 2H), 7.33 (d, *J* = 8.5 Hz, 2H), 4.77 (br. s, 1H), 4.31 (t, *J* = 6.7 Hz, 2H), 4.13 – 4.04 (m, 2H), 3.18 – 3.07 (m, 2H), 2.97 (d, *J* = 6.6 Hz, 2H), 2.69 – 2.64 (m, 2H), 2.63 (s, 3H), 2.56 (s, 3H), 2.45 (s, 3H), 2.39 – 2.26 (m, 2H), 1.74 – 1.59 (m, 3H), 1.44 (s, 9H), 1.16 – 1.02 (m, 2H). **^{13}C NMR (176 MHz, CDCl$_3$)** δ 156.6, 154.9, 141.9, 141.8, 140.1, 136.7, 136.0, 130.1, 125.9, 117.9, 79.6, 50.2, 48.8, 48.0, 43.6, 37.2, 29.7, 28.6, 23.7, 21.4, 20.0, 12.5. **HRMS(ESI):** $[M+H]^+$ calcd. $C_{28}H_{41}N_6O_5S$ m/z 573.2854, found 573.2854.

***tert*-Butyl 4-(((4-(3,4-dimethyl-7-oxo-2-(*p*-tolyl)-2,7-dihydro-6*H*-pyrazolo[3,4-*d*]pyridazin-6-yl) butyl)sulphonamido)methyl)piperidine-1-carboxylate** (**S8b**)

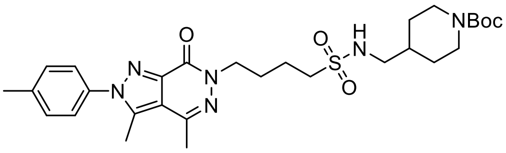

The title product compound **S8b** was prepared using general procedure B from **S7b** (1.0 g, 2.31 mmol) as a yellow solid (1.1 g, 1.83 mmol, 79%). TLC (100% EtOAc): Rf = 0.30. **^{1}H NMR (500 MHz, CDCl$_3$)** δ 7.36 (d, *J* = 8.5 Hz, 2H), 7.33 (d, *J* = 8.5 Hz, 2H), 4.24 (t, *J* = 6.7 Hz, 2H), 4.12 – 4.06 (m, 2H), 3.15 – 3.09 (m, 2H), 2.99 (t, *J* = 6.4 Hz, 2H), 2.71 – 2.64 (m, 2H), 2.63 (s, 3H), 2.56 (s, 3H), 2.45 (s, 3H), 2.02 – 1.95 (m, 2H), 1.88 – 1.81 (m, 2H), 1.76 – 1.60 (m, 3H), 1.45 (s, 9H), 1.10 (qd, *J* = 12.4, 4.4 Hz, 2H). **^{13}C NMR (176 MHz, CDCl$_3$)** δ 156.5, 154.9, 142.0, 141.5, 140.0, 136.6, 136.1, 130.1, 125.9, 117.8, 79.6, 52.1, 48.8, 48.5, 43.6, 37.2, 29.7, 28.6, 27.3, 21.4, 20.9, 20.0, 12.4. **HRMS(ESI):** [M+H]$^+$ calcd. $C_{29}H_{43}N_6O_5S$ m/z 587.3010, found 587.3010.

***tert*-Butyl 4-(((5-(3,4-dimethyl-7-oxo-2-(*p*-tolyl)-2,7-dihydro-6*H*-pyrazolo[3,4-*d*]pyridazin-6-yl) pentyl)sulphonamido)methyl)piperidine-1-carboxylate** (**S8c**)

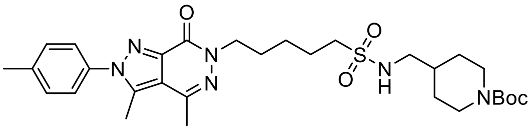

The title product compound **S8c** was prepared using general procedure B from **S7c** (1.0 g, 2.24 mmol) as a yellow solid (0.93 g, 1.54 mmol, 69%). TLC (100% EtOAc): Rf = 0.30. **^{1}H NMR (500 MHz, CDCl$_3$)** δ 7.36 (d, *J* = 8.6 Hz, 2H), 7.33 (d, *J* = 8.5 Hz, 2H), 4.78 (br. s, 1H), 4.21 (t, *J* = 7.0 Hz, 2H), 4.08 (d, *J* = 13.3 Hz, 2H), 3.03 – 2.94 (m, 4H), 2.70 – 2.63 (m, 2H), 2.62 (s, 3H), 2.57 (s, 3H), 2.45 (s, 3H), 1.92 – 1.82 (m, 6H), 1.78 – 1.63 (m, 3H), 1.44 (s, 9H), 1.09 (qd, *J* = 12.5, 4.4 Hz, 2H). **^{13}C NMR (176 MHz, CDCl$_3$)** δ 156.6, 154.9, 142.1, 141.4, 140.0, 136.5, 136.1, 130.1, 125.9, 117.8, 79.5, 52.4, 48.8, 48.6, 43.6, 37.2, 29.7, 28.6, 28.3, 25.0, 23.4, 21.4, 20.0, 12.5. **HRMS(ESI):** [M+H]$^+$ calcd. $C_{30}H_{45}N_6O_5S$ m/z 601.3167, found 601.3167.

***tert*-Butyl 4-(((6-(3,4-dimethyl-7-oxo-2-(*p*-tolyl)-2,7-dihydro-6*H*-pyrazolo[3,4-*d*]pyridazin-6-yl) hexyl)sulphonamido)methyl)piperidine-1-carboxylate** (**S8d**)

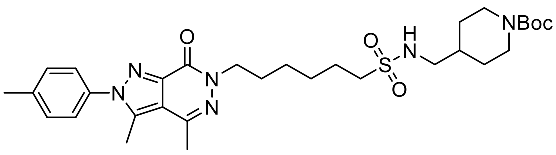

The title product compound **S8d** was prepared using general procedure B from **S7d** (1.0 g, 2.17 mmol) as a yellow solid (1.2 g, 1.98 mmol, 91%). TLC (100% EtOAc): Rf = 0.39. **^{1}H NMR (700 MHz, CDCl$_3$)** δ 7.37 (d, *J* = 8.4 Hz, 2H), 7.33 (d, *J* = 8.1 Hz, 2H), 4.49 (br. s, 1H), 4.19 (t, *J* = 7.2 Hz, 2H), 4.12 – 4.07 (m, 2H), 3.02 – 2.96 (m, 4H), 2.70 – 2.64 (m, 2H), 2.63 (s, 3H), 2.57 (s, 3H), 2.45 (s, 3H), 1.88 – 1.81 (m, 2H), 1.81 – 1.75 (m, 2H), 1.72 (d, *J* = 12.6 Hz, 2H), 1.68 – 1.63 (m, 1H), 1.54 – 1.47 (m, 2H), 1.44 (s, 9H), 1.43 – 1.37 (m, 2H), 1.11 (qd, *J* = 12.6, 4.4 Hz, 2H). **^{13}C NMR (176 MHz, CDCl$_3$)** δ 156.3,

154.9, 142.2, 141.1, 140.0, 136.4, 136.2, 130.0, 126.0, 117.8, 79.6, 52.6, 49.4, 48.8, 43.6, 37.2, 29.7, 28.6, 28.2, 27.8, 26.0, 23.7, 21.4, 20.0, 12.4. **HRMS(ESI):** $[M+H]^+$ calcd. $C_{31}H_{47}N_6O_5S$ m/z 615.3323, found 615.3323.

4-(3,4-Dimethyl-7-oxo-2-(*p*-tolyl)-2,7-dihydro-6*H*-pyrazolo[3,4-*d*]pyridazin-6-yl)-*N*-(2-phenylpropyl)butanamide (Deltazinone 1)

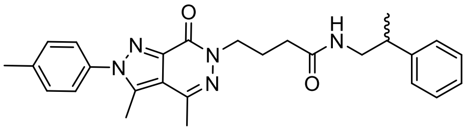

The title product compound **1** was prepared according previously published procedure from **S5** (0.95 g, 2.79 mmol, 1 equiv) and *β*-methylphenethylamine (0.43 ml, 2.93 mmol, 1.1 equiv) as a white solid (1.2 g, 2.62 mmol, 94%). ^{1}H NMR, ^{13}C NMR and HRMS (ESI) are identical with the reported ones. **^{1}H NMR (700 MHz, CDCl$_3$)** δ 7.38 – 7.33 (m, 4H), 7.31 – 7.26 (m, 4H), 7.22 – 7.17 (m, 1H), 3.96 – 3.84 (m, 2H), 3.61 – 3.57 (m, 1H), 3.57 – 3.51 (m, 1H), 3.22 – 3.16 (m, 1H), 2.64 (s, 3H), 2.56 (d, *J* = 1.3 Hz, 3H), 2.46 (s, 3H), 2.44 – 2.33 (m, 2H), 2.11 – 2.03 (m, 2H), 1.32 (d, *J* = 7.0 Hz, 3H). **^{13}C NMR (176 MHz, CDCl$_3$)** δ 174.7, 157.4, 143.9, 141.6, 141.6, 140.3, 136.9, 135.9, 130.1, 128.8, 127.6, 126.9, 125.9, 118.1, 48.5, 47.2, 39.4, 31.6, 25.8, 21.4, 20.0, 19.9, 12.5. **HRMS(ESI):** $[M+H]^+$ calcd. $C_{27}H_{32}N_5O_2$ m/z 458.2551, found 458.2552.

***N*-(3-chloropropyl)-4-(3,4-dimethyl-7-oxo-2-(*p*-tolyl)-2,7-dihydro-6*H*-pyrazolo[3,4-*d*]pyridazin-6-yl)butanamide (2a)**

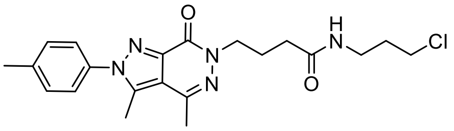

The title product compound **2a** was prepared using general procedure A from **S5** (34.0 mg, 0.10 mmol, 1 equiv) and **S9a** (19.9 mg, 0.11 mmol, 1.1 equiv) as a yellow oil (30.4 mg, 0.07 mmol, 73% over three steps). TLC (100% EtOAc): Rf = 0.58. **^{1}H NMR (700 MHz, DMSO-*d*$_6$)** δ 7.48 (br. s, 1H, NH), 7.48 (d, *J* = 8.3 Hz, 2H), 7.43 (d, *J* = 8.4 Hz, 2H), 4.08 (t, *J* = 6.9 Hz, 2H), 4.02 (t, *J* = 6.4 Hz, 2H), 3.65 (t, *J* = 6.6 Hz, 2H), 2.59 (s, 3H), 2.54 (s, 3H), 2.43 (s, 3H), 2.35 (t, *J* = 7.4 Hz, 2H), 1.99 – 1.93 (m, 2H), 1.87 – 1.77 (m, 2H). **^{13}C NMR (151 MHz, DMSO-*d*$_6$)** δ 172.4, 155.3, 141.1, 141.1, 139.4, 137.5, 135.9, 129.9, 125.8, 117.2, 59.9, 48.2, 37.7, 30.7, 23.7, 20.8, 19.4, 14.1, 11.9. **HRMS(ESI):** $[M+H]^+$ calcd. $C_{21}H_{27}ClN_5O_2$ m/z 416.1848, found 416.1864.

***N*-(5-chloropentyl)-4-(3,4-dimethyl-7-oxo-2-(*p*-tolyl)-2,7-dihydro-6*H*-pyrazolo[3,4-*d*]pyridazin-6-yl)butanamide (2b)**

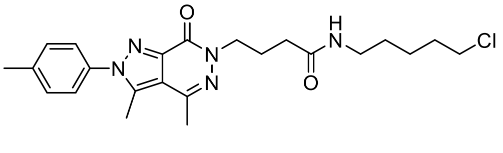

The title product compound **2b** was prepared using general procedure A from **S5** (81.7 mg, 0.24 mmol, 1 equiv) and **S9b** (54.8 mg, 0.25 mmol, 1.1 equiv) as a yellow oil (63.9 mg, 0.14 mmol, 60% over three steps). TLC (100% EtOAc): Rf = 0.66. **^{1}H NMR**

(700 MHz, DMSO-*d*$_6$) δ 7.48 (br. s, 1H, NH), 7.48 (d, *J* = 8.4 Hz, 2H), 7.43 (d, *J* = 8.3 Hz, 2H), 4.07 (t, *J* = 6.9 Hz, 2H), 4.02 (t, *J* = 6.4 Hz, 2H), 3.65 (t, *J* = 6.6 Hz, 2H), 2.59 (s, 3H), 2.54 (s, 3H), 2.43 (s, 3H), 2.35 (t, *J* = 7.3 Hz, 2H), 1.98 – 1.93 (m, 2H), 1.87 – 1.73 (m, 2H), 1.71 – 1.64 (m, 2H), 1.61 – 1.51 (m, 2H). **^{13}C NMR (176 MHz, DMSO-*d*$_6$)** δ 172.4, 155.3, 141.2, 141.1, 139.4, 137.5, 135.9, 129.9, 125.8, 117.2, 48.2, 45.1, 37.7, 34.7, 30.7, 28.7, 25.7, 23.7, 20.8, 19.4, 11.9. **HRMS(ESI):** $[M+H]^+$ calcd. $C_{23}H_{31}ClN_5O_2$ m/z 444.2161, found 444.2143.

***N*-(3-bromopropyl)-4-(3,4-dimethyl-7-oxo-2-(*p*-tolyl)-2,7-dihydro-6*H*-pyrazolo[3,4-*d*]pyridazin-6-yl)butanamide** (**2c**)

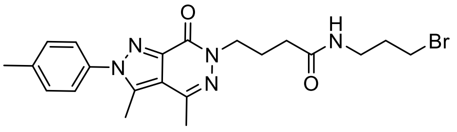

The title product compound **2c** was prepared using general procedure A from **S5** (34.0 mg, 0.10 mmol, 1 equiv) and **S9a** (19.9 mg, 0.11 mmol, 1.1 equiv) as a yellow oil (14.3 mg, 0.03 mmol, 31% over three steps). TLC (100% EtOAc): Rf = 0.61. **^{1}H NMR (700 MHz, DMSO-*d*$_6$)** δ 7.48 (br. s, 1H, NH), 7.48 (d, *J* = 8.4 Hz, 2H), 7.43 (d, *J* = 8.1 Hz, 2H), 4.08 (t, *J* = 6.9 Hz, 2H), 4.02 (t, *J* = 6.4 Hz, 2H), 3.55 (t, *J* = 6.6 Hz, 2H), 2.59 (s, 3H), 2.52 (s, 3H), 2.43 (s, 3H), 2.35 (t, *J* = 7.3 Hz, 2H), 2.00 – 1.92 (m, 2H), 1.89 – 1.81 (m, 2H). **^{13}C NMR (176 MHz, DMSO-*d*$_6$)** δ 172.4, 155.2, 141.1, 139.4, 137.5, 135.9, 129.9, 125.7, 117.1, 48.1, 34.7, 30.6, 28.9, 26.9, 23.6, 20.7, 19.4, 11.9. **HRMS(APCI):** $[M+H]^+$ calcd. $C_{21}H_{27}BrN_5O_2$ m/z 460.1343, found 460.1327.

***N*-(5-bromopentyl)-4-(3,4-dimethyl-7-oxo-2-(*p*-tolyl)-2,7-dihydro-6*H*-pyrazolo[3,4-*d*]pyridazin-6-yl)butanamide** (**2d**)

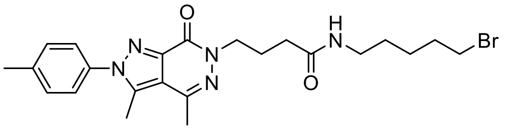

The title product compound **2d** was prepared using general procedure A from **S5** (81.7 mg, 0.24 mmol, 1 equiv) and **S9b** (54.8 mg, 0.25 mmol, 1.1 equiv) as a yellow oil (41.0 mg, 0.08 mmol, 35% over three steps). TLC (100% EtOAc): Rf = 0.65. **^{1}H NMR (700 MHz, DMSO-*d*$_6$)** δ 7.48 (br. s, 1H, NH), 7.49 (d, *J* = 8.3 Hz, 2H), 7.44 (d, *J* = 8.3 Hz, 2H), 4.08 (t, *J* = 6.9 Hz, 2H), 4.02 (t, *J* = 6.5 Hz, 2H), 3.55 (t, *J* = 6.6 Hz, 2H), 2.59 (s, 3H), 2.52 (s, 3H), 2.43 (s, 3H), 2.35 (t, *J* = 7.3 Hz, 2H), 1.96 (p, *J* = 7.2 Hz, 2H), 1.86 – 1.84 (m, 2H), 1.71 – 1.66 (m, 2H), 1.53 – 1.48 (m, 2H). **^{13}C NMR (176 MHz, DMSO-*d*$_6$)** δ 172.4, 155.2, 141.1, 139.4, 137.5, 135.9, 129.9, 125.7, 117.1, 48.1, 34.7, 30.6, 28.9, 26.9, 26.1, 23.6, 22.1, 20.7, 19.4, 11.9. **HRMS(APCI):** $[M+H]^+$ calcd. $C_{23}H_{31}BrN_5O_2$ m/z 488.1656, found 488.1645.

***N*-(3-chloropropyl)-4-(3,4-dimethyl-7-oxo-2-(*p*-tolyl)-2,7-dihydro-6*H*-pyrazolo[3,4-*d*]pyridazin-6-yl)-*N*-(piperidin-4-ylmethyl)butanamide** (**3a**)

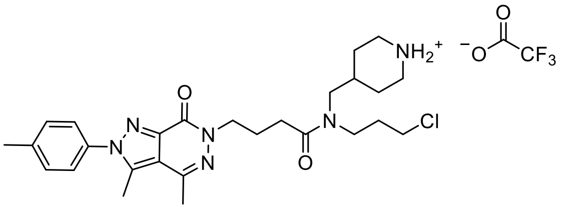

The title product compound **3a** (as a CF_3COO^- salt) was prepared using general procedure A from **S5** (250 mg, 0.74 mmol, 1 equiv) and **S9c** (315.2 mg, 0.81 mmol, 1.1 equiv) as a yellow oil (295.0 mg, 0.57 mmol, 82% over three steps). TLC (MeOH/DCM = 5%): Rf = 0.55. **^{1}H NMR (600 MHz, DMSO-*d*$_6$)** δ 9.03 (br. s, 1H), 8.71 (br. s, 1H), 7.44 (d, *J* = 8.1 Hz, 2H), 7.40 (d, *J* = 8.1 Hz, 2H), 4.02 (t, *J* = 7.1 Hz, 2H), 3.87 (4H hiding in water peak), 3.23 (d, *J* = 13.3 Hz, 2H), 2.94 (t, *J* = 6.3 Hz, 2H), 2.81 (q, *J* = 12.6 Hz, 2H), 2.56 (s, 3H), 2.49 (s, 3H), 2.39 (s, 3H), 2.13 (t, *J* = 7.6 Hz, 2H), 1.97 – 1.87 (m, 2H), 1.79 – 1.69 (m, 2H), 1.71 – 1.61 (m, 1H), 1.33 – 1.23 (m, 2H), 1.23 – 0.98 (m, 2H). **^{13}C NMR (151 MHz, DMSO-*d*$_6$)** δ 172.1, 159.1, 155.5, 141.4, 141.3, 139.6, 137.7, 136.1, 130.1, 125.9, 117.4, 56.3, 48.8, 43.5, 43.1, 33.9, 32.8, 26.4, 24.9, 20.9, 19.6, 12.0. **HRMS(ESI):** $[M+H]^+$ calcd. $C_{27}H_{38}ClN_6O_2$ m/z 513.2739, found 513.2714.

***N*-(5-chloropentyl)-4-(3,4-dimethyl-7-oxo-2-(*p*-tolyl)-2,7-dihydro-6*H*-pyrazolo[3,4-*d*]pyridazin-6-yl)-*N*-(piperidin-4-ylmethyl)butanamide** (**3b**)

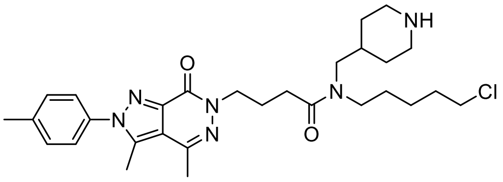

The title product compound **3b** was prepared using general procedure A from **S5** (250 mg, 0.74 mmol, 1 equiv) and **S9d** (336.4 mg, 0.81 mmol, 1.1 equiv) as a yellow oil (280.1 mg, 0.52 mmol, 76% over three steps). TLC (MeOH/DCM = 3%): Rf = 0.57. **^{1}H NMR (600 MHz, DMSO-*d*$_6$)** δ 8.77 (br. s, 1H, NH), 7.45 (d, *J* = 8.4 Hz, 2H), 7.41 (d, *J* = 8.3 Hz, 2H), 4.08 – 4.02 (m, 2H), 3.56 (t, *J* = 6.6 Hz, 2H), 3.26 – 3.10 (m, 6H), 2.83 – 2.75 (m, 2H), 2.58 (s, 3H), 2.49 (s, 3H), 2.41 (s, 3H), 2.36 – 2.28 (m, 2H), 1.98 – 1.90 (m, 2H), 1.90 – 1.83 (m, 1H), 1.73 – 1.61 (m, 4H), 1.51 – 1.39 (m, 2H), 1.35 – 1.24 (m, 4H), 1.21 (br. s, 1H). **^{13}C NMR (151 MHz, DMSO-*d*$_6$)** δ 171.7, 155.3, 141.2, 141.1, 139.5, 137.5, 135.9, 130.0, 125.8, 117.2, 51.7, 49.1, 48.6, 47.4, 45.4, 42.9, 31.8, 29.1, 27.6, 26.2, 24.3, 23.7, 20.8, 19.5, 11.9. **HRMS(ESI):** $[M+H]^+$ calcd. $C_{29}H_{42}ClN_6O_2$ m/z 541.3052, found 541.3041.

***N*-(3-bromopropyl)-4-(3,4-dimethyl-7-oxo-2-(*p*-tolyl)-2,7-dihydro-6*H*-pyrazolo[3,4-*d*]pyridazin-6-yl)-*N*-(piperidin-4-ylmethyl)butanamide** (**3c**)

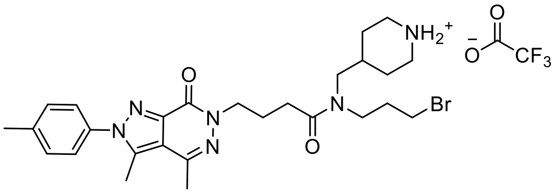

The title product compound **3c** (as a CF_3COO^- salt) was prepared using general procedure A from **S5** (250 mg, 0.74 mmol, 1 equiv) and **S9c** (315.2 mg, 0.81 mmol, 1.1 equiv) as a yellow solid (265.2 mg,

0.48 mmol, 35% over three steps). TLC (MeOH/DCM = 5%): Rf = 0.61. **^{1}H NMR (600 MHz, DMSO-*d*$_6$)** δ 8.83 (br. s, 1H), 8.59 (br. s, 1H), 7.45 (d, *J* = 8.1 Hz, 3H), 7.40 (d, *J* = 8.1 Hz, 2H), 3.99 (t, *J* = 6.6 Hz, 2H), 3.42 – 3.05 (m, 4H), 2.91 – 2.73 (m, 4H), 2.57 (s, 3H), 2.48 (s, 3H), 2.38 (s, 3H), 2.11 (t, *J* = 7.4 Hz, 2H), 1.96 – 1.60 (m, 6H), 1.57 – 1.23 (m, 4H), 1.17 (br. s, 1H). **^{13}C NMR (151 MHz, DMSO-*d*$_6$)** δ 171.9, 155.3, 141.4, 141.2, 139.5, 137.8, 135.8, 130.1, 125.8, 117.2, 60.7, 48.7, 43.3, 42.8, 33.6, 32.7, 29.2, 26.1, 24.7, 20.9, 19.6, 12.2. **HRMS(ESI):** [M+H]$^+$ calcd. $C_{27}H_{38}BrN_6O_2$ m/z 557.2234, found 557.2247.

***N*-(5-bromopentyl)-4-(3,4-dimethyl-7-oxo-2-(*p*-tolyl)-2,7-dihydro-6*H*-pyrazolo[3,4-*d*]pyridazin-6-yl)-*N*-(piperidin-4-ylmethyl)butanamide** (**3d**)

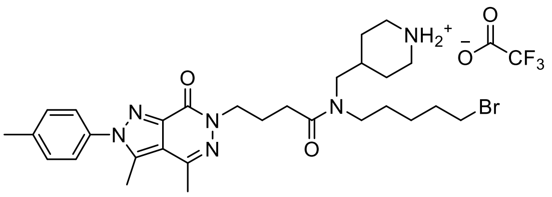

The title product compound **3d** (as a CF_3COO^- salt) was prepared using general procedure A from **S5** (250 mg, 0.71 mmol, 1 equiv) and **S9d** (336.4 mg, 0.81 mmol, 1.1 equiv) as a yellow oil (210.1 mg, 0.36 mmol, 53% over three steps). TLC (MeOH/DCM = 3%): Rf = 0.67. **^{1}H NMR (600 MHz, DMSO-*d*$_6$)** δ 8.78 (br. s, 1H), 8.56 (br. s, 1H), 7.43 (d, *J* = 7.9 Hz, 2H), 7.38 (d, *J* = 8.0 Hz, 2H), 4.05 – 3.92 (m, 2H), 3.57 (m, 2H hiding in water peak), 3.49 – 2.99 (m, 6H), 2.94 – 2.70 (m, 2H), 2.55 (s, 3H), 2.47 (s, 3H), 2.36 (s, 3H), 2.32 – 2.23 (m, 1H), 2.11 (t, *J* = 7.6 Hz, 1H), 1.97 – 1.80 (m, 2H), 1.78 – 1.59 (m, 2H), 1.49 – 1.04 (m, 6H). **^{13}C NMR (151 MHz, DMSO-*d*$_6$)** δ 171.9, 155.5, 141.5, 141.2, 139.5, 137.8, 135.9, 130.1, 125.9, 117.3, 60.6, 48.8, 43.4, 43.0, 33.6, 32.7, 32.1, 29.2, 26.1, 24.8, 22.9, 20.9, 19.7, 12.2. **HRMS(ESI):** [M+H]$^+$ calcd. $C_{29}H_{42}BrN_6O_2$ m/z 585.2547, found 585.2525.

***N*-(5-bromopentyl)-3-(3,4-dimethyl-7-oxo-2-(*p*-tolyl)-2,7-dihydro-6*H*-pyrazolo[3,4-*d*]pyridazin-6-yl)-*N*-(piperidin-4-ylmethyl)propane-1-sulphonamide** (**4a**)

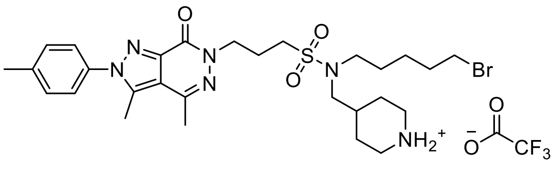

The title product compound **4a** (as a CF_3COO^- salt) was prepared using general procedure C from **S8a** (51.5 mg, 0.09 mmol, 1 equiv) and 1,5-dibromopentane (41.4 mg, 0.18 mmol, 2.0 equiv) as a yellow oil (44.1 mg, 0.06 mmol, 66% over two steps). TLC (MeOH/DCM = 5%): Rf = 0.34. **^{1}H NMR (600 MHz, DMSO-*d*$_6$)** δ 8.45 (br. s, 1H), 8.11 (br. s, 1H), 7.44 (d, *J* = 8.4 Hz, 2H), 7.40 (d, *J* = 8.0 Hz, 2H), 4.11 (t, *J* = 7.1 Hz, 2H), 3.48 (t, *J* = 6.6 Hz, 1H), 3.23 (d, *J* = 12.7 Hz, 1H), 3.17 – 3.10 (m, 1H), 3.10 – 3.05 (m, 1H), 3.01 (d, *J* = 7.3 Hz, 2H), 2.81 (q, *J* = 12.9 Hz, 2H), 2.56 (s, 3H), 2.49 (s, 3H), 2.39 (s, 3H), 2.07 – 1.99 (m, 2H), 1.81 – 1.71 (m, 4H), 1.48 (h, *J* = 7.4 Hz, 2H), 1.37 – 1.26 (m, 2H), 1.25 – 1.15 (m, 3H). **^{13}C NMR (151 MHz, DMSO-*d*$_6$)** δ 158.0, 155.3, 141.6, 141.1, 139.5, 137.7, 135.9, 130.0,

125.8, 117.3, 60.6, 52.6, 48.2, 47.7, 47.5, 42.9, 35.2, 32.1, 31.8, 27.6, 26.2, 24.9, 22.6, 20.8, 19.5, 11.9. **HRMS(ESI):** $[M+H]^+$ calcd. $C_{28}H_{42}BrN_6O_3S$ m/z 621.2217, found 621.2196.

***N*-(5-bromopentyl)-4-(3,4-dimethyl-7-oxo-2-(*p*-tolyl)-2,7-dihydro-6*H*-pyrazolo[3,4-*d*]pyridazin-6-yl)-*N*-(piperidin-4-ylmethyl)butane-1-sulphonamide** (**4b**)

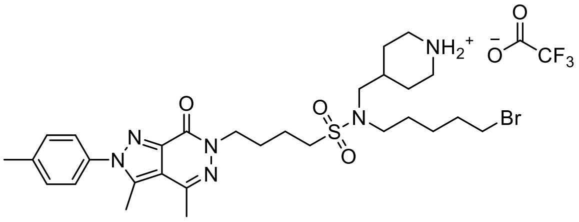

The title product compound **4b** (as a CF_3COO^- salt) was prepared using general procedure C from **S8b** (52.8 mg, 0.09 mmol, 1 equiv) and 1,5-dibromopentane (41.4 mg, 0.18 mmol, 2.0 equiv) as a yellow oil (67.5 mg, 0.09 mmol, 99% over two steps). TLC (MeOH/DCM = 5%): Rf = 0.40. **^{1}H NMR (700 MHz, DMSO-*d*$_6$)** δ 8.57 (br. s, 1H), 8.20 (br. s, 1H), 7.48 (d, *J* = 8.3 Hz, 2H), 7.43 (d, *J* = 8.1 Hz, 2H), 4.08 (t, *J* = 6.7 Hz, 2H), 3.52 (t, *J* = 6.6 Hz, 2H), 3.26 (d, *J* = 13.0 Hz, 2H), 3.16 – 3.11 (m, 2H), 3.11 – 3.07 (m, 2H), 3.00 (d, *J* = 7.4 Hz, 2H), 2.85 (q, *J* = 12.3 Hz, 2H), 2.60 (s, 3H), 2.54 (s, 3H), 2.43 (s, 3H), 1.87 – 1.76 (m, 6H), 1.69 – 1.61 (m, 2H), 1.57 – 1.48 (m, 2H), 1.44 – 1.32 (m, 2H), 1.31 – 1.18 (m, 3H). **^{13}C NMR (176 MHz, DMSO-*d*$_6$)** δ 158.1, 155.3, 141.2, 141.1, 139.5, 137.6, 135.9, 129.9, 125.8, 117.1, 60.6, 52.8, 49.1, 48.4, 48.1, 42.9, 35.2, 32.2, 31.8, 27.8, 27.0, 26.2, 24.9, 20.8, 20.2, 19.5, 11.9. **HRMS(ESI):** $[M+H]^+$ calcd. $C_{29}H_{44}BrN_6O_3S$ m/z 635.2373, found 635.2345.

***N*-(5-bromopentyl)-5-(3,4-dimethyl-7-oxo-2-(*p*-tolyl)-2,7-dihydro-6*H*-pyrazolo[3,4-*d*]pyridazin-6-yl)-*N*-(piperidin-4-ylmethyl)pentane-1-sulphonamide** (**4c**)

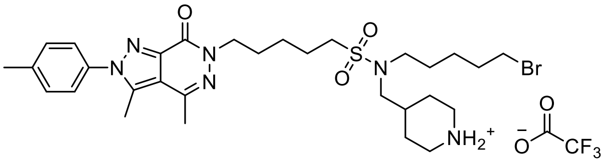

The title product compound **4c** (as a CF_3COO^- salt) was prepared using general procedure C from **S8c** (40.0 mg, 0.07 mmol, 1 equiv) and 1,5-dibromopentane (30.6 mg, 0.13 mmol, 2.0 equiv) as a yellow oil (30.0 mg, 0.04 mmol, 57% over two steps). TLC (MeOH/DCM = 5%): Rf = 0.43. **^{1}H NMR (700 MHz, DMSO-*d*$_6$)** δ 8.54 (br. s, 1H), 8.17 (br. s, 1H), 7.48 (d, *J* = 8.3 Hz, 2H), 7.44 (d, *J* = 8.2 Hz, 2H), 4.05 (t, *J* = 7.2 Hz, 2H), 3.53 (t, *J* = 6.6 Hz, 2H), 3.27 (d, *J* = 12.4 Hz, 2H), 3.13 – 3.08 (m, 2H), 3.08 – 3.03 (m, 2H), 3.02 (d, *J* = 7.2 Hz, 2H), 2.86 (q, *J* = 11.9 Hz, 2H), 2.60 (s, 3H), 2.52 (s, 3H), 2.43 (s, 3H), 1.88 – 1.78 (m, 4H), 1.77 – 1.65 (m, 4H), 1.58 – 1.51 (m, 2H), 1.43 – 1.32 (m, 4H), 1.32 – 1.19 (m, 4H). **^{13}C NMR (176 MHz, DMSO-*d*$_6$)** δ 158.1, 155.1, 141.1, 141.0, 139.4, 137.5, 135.9, 129.9, 125.7, 117.1, 115.5, 60.5, 52.7, 49.4, 48.5, 48.3, 42.9, 35.1, 32.2, 31.8, 27.8, 27.8, 26.2, 24.9, 24.8, 22.6, 20.7, 19.4, 11.9. **HRMS(ESI):** $[M+H]^+$ calcd. $C_{30}H_{46}BrN_6O_3S$ m/z 649.2530, found 649.2522.

N-(5-bromopentyl)-6-(3,4-dimethyl-7-oxo-2-(p-tolyl)-2,7-dihydro-6H-pyrazolo[3,4-d]pyridazin-6-yl)-N-(piperidin-4-ylmethyl)hexane-1-sulphonamide (**4d**)

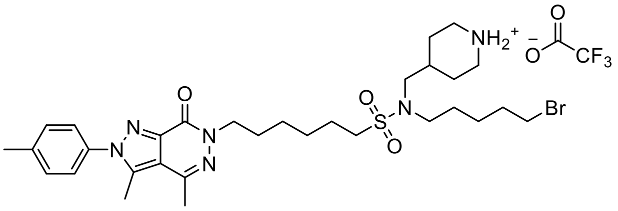

The title product compound **4d** (as a CF_3COO^- salt) was prepared using general procedure C from **S8d** (20.0 mg, 0.03 mmol, 1 equiv) and 1,5-dibromopentane (15.0 mg, 0.07 mmol, 2.0 equiv) as a yellow oil (23.3 mg, 0.03 mmol, 79% over two steps). TLC (MeOH/DCM = 5%): Rf = 0.59. **^{1}H NMR (700 MHz, DMSO-d_6)** δ 8.52 (br. s, 1H), 8.14 (br. s, 1H), 7.48 (d, J = 8.3 Hz, 2H), 7.43 (d, J = 8.0 Hz, 2H), 4.04 (t, J = 7.2 Hz, 2H), 3.53 (t, J = 6.6 Hz, 2H), 3.38 (d, J = 6.5 Hz, 1H), 3.27 (d, J = 12.4 Hz, 2H), 3.13 – 3.08 (m, 2H), 3.07 – 3.00 (m, 4H), 2.86 (q, J = 12.2 Hz, 2H), 2.59 (s, 3H), 2.52 (s, 3H), 2.43 (s, 3H), 1.88 – 1.78 (m, 5H), 1.74 – 1.66 (m, 2H), 1.66 – 1.58 (m, 2H), 1.59 – 1.51 (m, 2H), 1.45 – 1.38 (m, 3H), 1.39 – 1.33 (m, 2H), 1.34 – 1.28 (m, 3H), 1.28 – 1.19 (m, 4H). **^{13}C NMR (176 MHz, DMSO-d_6)** δ 158.0, 155.1, 141.1, 141.0, 139.4, 137.5, 135.9, 129.9, 125.7, 117.1, 60.5, 52.7, 49.5, 48.7, 48.3, 42.9, 35.1, 32.2, 31.8, 28.0, 27.7, 27.3, 26.2, 25.6, 24.9, 22.8, 20.7, 19.4, 11.9. **HRMS(ESI):** $[M+H]^+$ calcd. $C_{31}H_{48}BrN_6O_3S$ m/z 663.2687, found 663.2665.

N-(2-bromoethyl)-4-(3,4-dimethyl-7-oxo-2-(p-tolyl)-2,7-dihydro-6H-pyrazolo[3,4-d]pyridazin-6-yl)-N-(piperidin-4-ylmethyl)butane-1-sulphonamide (**5a**)

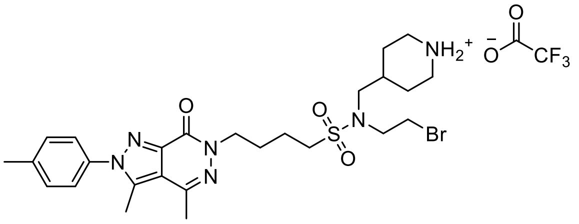

The title product compound **5a** (as a CF_3COO^- salt) was prepared using general procedure C from **S8b** (20.0 mg, 0.03 mmol, 1 equiv) and 1,2-dibromoethane (12.8 mg, 0.07 mmol, 2.0 equiv) as a white solid (9.5 mg, 0.02 mmol, 47% over two steps). TLC (MeOH/DCM = 5%): Rf = 0.32. **^{1}H NMR (700 MHz, DMSO-d_6)** δ 8.47 (br. s, 1H), 8.13 (br. s, 1H), 7.49 (d, J = 8.3 Hz, 2H), 7.44 (d, J = 8.1 Hz, 2H), 4.08 (t, J = 6.8 Hz, 2H), 3.57 (t, J = 7.6 Hz, 2H), 3.51 – 3.48 (m, 2H), 3.26 (d, J = 12.6 Hz, 2H), 3.24 – 3.20 (m, 2H), 3.06 (d, J = 7.4 Hz, 2H), 2.86 (t, J = 11.3 Hz, 2H), 2.60 (s, 3H), 2.53 (s, 3H), 2.43 (s, 3H), 1.90 – 1.78 (m, 5H), 1.70 – 1.62 (m, 2H), 1.24 – 1.17 (m, 3H). **^{13}C NMR (176 MHz, DMSO-d_6)** δ 157.8, 155.3, 141.2, 141.1, 139.5, 137.6, 135.9, 129.9, 125.8, 117.1, 116.5, 53.2, 49.8, 49.3, 48.2, 42.9, 32.1, 30.8, 27.0, 26.1, 20.8, 20.2, 19.5, 11.9. **HRMS(ESI):** $[M+H]^+$ calcd. $C_{26}H_{38}BrN_6O_3S$ m/z 593.1904, found 593.1888.

***N*-(3-bromopropyl)-4-(3,4-dimethyl-7-oxo-2-(*p*-tolyl)-2,7-dihydro-6*H*-pyrazolo[3,4-*d*]pyridazin-6-yl)-*N*-(piperidin-4-ylmethyl)butane-1-sulphonamide** (**5b**)

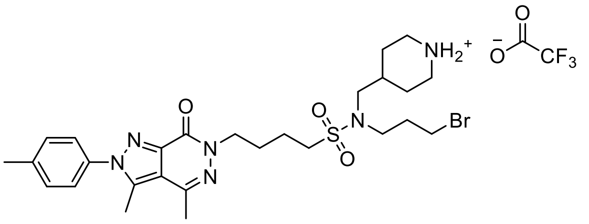

The title product compound **5b** (as a CF_3COO^- salt) was prepared using general procedure C from **S8b** (20.0 mg, 0.03 mmol, 1 equiv) and 1,3-dibromopropane (13.8 mg, 0.07 mmol, 2.0 equiv) as a yellow oil (20.1 mg, 0.03 mmol, 97% over two steps). TLC (MeOH/DCM = 5%): Rf = 0.35. **^{1}H NMR (700 MHz, DMSO-*d*$_6$)** δ 8.48 (br. s, 1H), 8.13 (br. s, 1H), 7.48 (d, *J* = 8.3 Hz, 2H), 7.44 (d, *J* = 8.3 Hz, 2H), 4.08 (t, *J* = 6.8 Hz, 2H), 3.50 (t, *J* = 6.5 Hz, 2H), 3.27 (d, *J* = 12.3 Hz, 2H), 3.25 – 3.21 (m, 2H), 3.19 – 3.13 (m, 2H), 3.02 (d, *J* = 7.4 Hz, 2H), 2.85 (q, *J* = 11.5 Hz, 2H), 2.60 (s, 3H), 2.53 (s, 4H), 2.43 (s, 3H), 2.12 – 1.94 (m, 2H), 1.89 – 1.75 (m, 5H), 1.71 – 1.60 (m, 2H), 1.31 – 1.18 (m, 4H). **^{13}C NMR (176 MHz, DMSO-*d*$_6$)** δ 157.9, 155.3, 141.2, 141.1, 139.5, 137.6, 135.9, 129.9, 125.8, 117.1, 58.2, 52.8, 49.2, 48.1, 47.1, 42.9, 32.1, 31.9, 31.8, 27.0, 26.1, 20.8, 20.2, 19.5, 11.9. **HRMS(ESI):** $[M+H]^+$ calcd. $C_{27}H_{40}BrN_6O_3S$ m/z 607.2060, found 607.2045.

***N*-(4-bromobutyl)-4-(3,4-dimethyl-7-oxo-2-(*p*-tolyl)-2,7-dihydro-6*H*-pyrazolo[3,4-*d*]pyridazin-6-yl)-*N*-(piperidin-4-ylmethyl)butane-1-sulphonamide** (**5c**)

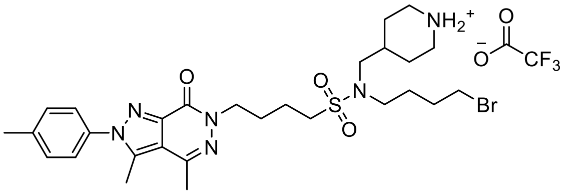

The title product compound **5c** (as a CF_3COO^- salt) was prepared using general procedure C from **S8b** (20.0 mg, 0.03 mmol, 1 equiv) and 1,4-dibromobutane (14.7 mg, 0.07 mmol, 2.0 equiv) as a yellow oil (22.1 mg, 0.03 mmol, 92% over two steps). TLC (MeOH/DCM = 5%): Rf = 0.37. **^{1}H NMR (700 MHz, DMSO-*d*$_6$)** δ 8.50 (br. s, 1H), 8.16 (br. s, 1H), 7.48 (d, *J* = 8.5 Hz, 2H), 7.44 (d, *J* = 8.4 Hz, 2H), 4.08 (t, *J* = 6.8 Hz, 2H), 3.54 (t, *J* = 6.7 Hz, 2H), 3.27 (d, *J* = 12.2 Hz, 2H), 3.16 – 3.11 (m, 4H), 3.01 (d, *J* = 7.3 Hz, 2H), 2.85 (q, *J* = 12.5 Hz, 2H), 2.60 (s, 3H), 2.53 (s, 8H), 2.43 (s, 3H), 1.89 – 1.75 (m, 7H), 1.64 (dtd, *J* = 16.9, 8.0, 4.5 Hz, 4H), 1.27 – 1.18 (m, 3H). **^{13}C NMR (176 MHz, DMSO-*d*$_6$)** δ 157.8, 155.3, 141.2, 141.1, 139.5, 137.6, 135.9, 129.9, 125.8, 117.1, 60.3, 52.9, 49.0, 48.1, 47.7, 42.9, 34.7, 32.2, 29.6, 27.3, 27.0, 26.2, 20.8, 20.2, 19.5, 11.9. **HRMS(ESI):** $[M+H]^+$ calcd. $C_{28}H_{42}BrN_6O_3S$ m/z 621.2217, found 621.2202.

N-(6-bromohexyl)-4-(3,4-dimethyl-7-oxo-2-(p-tolyl)-2,7-dihydro-6H-pyrazolo[3,4-d]pyridazin-6-yl)-N-(piperidin-4-ylmethyl)butane-1-sulphonamide (**5d**)

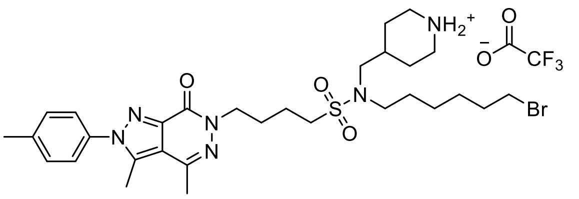

The title product compound **5d** (as a CF_3COO^- salt) was prepared using general procedure C from **S8b** (20.0 mg, 0.03 mmol, 1 equiv) and 1,6-dibromohexane (16.6 mg, 0.07 mmol, 2.0 equiv) as a yellow oil (22.9 mg, 0.03 mmol, 99% over two steps). TLC (MeOH/DCM = 5%): Rf = 0.45. **^{1}H NMR (700 MHz, DMSO-d_6)** δ 8.51 (br. s, 1H), 8.15 (br. s, 1H), 7.48 (d, J = 8.4 Hz, 2H), 7.44 (d, J = 8.4 Hz, 2H), 4.08 (t, J = 6.8 Hz, 2H), 3.51 (t, J = 6.7 Hz, 2H), 3.26 (d, J = 12.3 Hz, 2H), 3.15 – 3.10 (m, 2H), 3.08 (t, J = 7.9 Hz, 2H), 3.00 (d, J = 7.3 Hz, 2H), 2.85 (q, J = 12.6 Hz, 2H), 2.60 (s, 3H), 2.53 (s, 3H), 2.43 (s, 3H), 1.88 – 1.75 (m, 6H), 1.68 – 1.61 (m, 2H), 1.54 – 1.48 (m, 2H), 1.42 – 1.35 (m, 2H), 1.28 – 1.19 (m, 5H). **^{13}C NMR (176 MHz, DMSO-d_6)** δ 157.9, 155.3, 141.2, 141.1, 139.5, 137.6, 135.9, 129.9, 125.8, 117.1, 60.6, 52.6, 49.2, 48.3, 48.1, 42.9, 35.2, 32.2, 32.2, 28.4, 27.2, 27.0, 26.2, 25.4, 20.8, 20.2, 19.5, 11.9. **HRMS(ESI):** $[M+H]^+$ calcd. $C_{30}H_{46}BrN_6O_3S$ m/z 649.2530, found 649.2524.

N-(7-bromoheptyl)-4-(3,4-dimethyl-7-oxo-2-(p-tolyl)-2,7-dihydro-6H-pyrazolo[3,4-d]pyridazin-6-yl)-N-(piperidin-4-ylmethyl)butane-1-sulphonamide (**5e**)

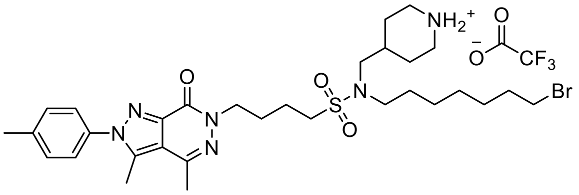

The title product compound **5e** (as a CF_3COO^- salt) was prepared using general procedure C from **S8b** (58.7 mg, 0.10 mmol, 1 equiv) and 1,7-dibromoheptane (52.0 mg, 0.20 mmol, 2.0 equiv) as a white solid (70.0 mg, 0.09 mmol, 93% over two steps). TLC (MeOH/DCM = 5%): Rf = 0.57. **^{1}H NMR (700 MHz, DMSO-d_6)** δ 8.61 (br. s, 1H), 8.25 (br. s, 1H), 7.48 (d, J = 8.3 Hz, 2H), 7.43 (d, J = 8.1 Hz, 2H), 4.08 (t, J = 6.8 Hz, 2H), 3.51 (t, J = 6.7 Hz, 2H), 3.26 (d, J = 12.2 Hz, 2H), 3.14 – 3.10 (m, 2H), 3.08 (t, J = 7.9 Hz, 2H), 2.99 (d, J = 7.3 Hz, 2H), 2.85 (q, J = 12.8 Hz, 2H), 2.60 (s, 3H), 2.52 (s, 4H), 2.43 (s, 3H), 1.86 – 1.74 (m, 7H), 1.69 – 1.61 (m, 2H), 1.50 (p, J = 7.7 Hz, 2H), 1.41 – 1.32 (m, 2H), 1.33 – 1.17 (m, 7H). **^{13}C NMR (176 MHz, DMSO-d_6)** δ 158.2, 155.3, 141.2, 141.1, 139.4, 137.6, 135.9, 129.9, 125.8, 117.1, 60.7, 52.6, 49.2, 48.4, 48.1, 42.9, 35.2, 32.2, 32.2, 28.5, 27.7, 27.5, 27.0, 26.2, 26.1, 20.8, 20.2, 19.5, 11.9. **HRMS(ESI):** $[M+H]^+$ calcd. $C_{31}H_{48}BrN_6O_3S$ m/z 663.2687, found 663.2683.

***N*-(5-chloropentyl)-4-(3,4-dimethyl-7-oxo-2-(*p*-tolyl)-2,7-dihydro-6*H*-pyrazolo[3,4-*d*]pyridazin-6-yl)-*N*-(piperidin-4-ylmethyl)butane-1-sulphonamide** (**5f**)

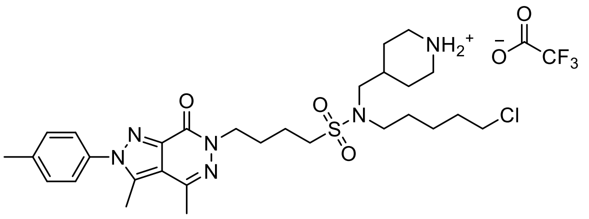

The title product compound **5f** (as a CF_3COO^- salt) was prepared by incubating **4b** (30.0 mg, 0.05 mmol, 1 equiv) with NaCl (13.8 mg, 0.24 mmol, 5 equiv) in DMSO at room temperature overnight, followed by purification by pre-HPLC as a white solid (28.2 mg, 0.04 mmol, 95%). TLC (MeOH/DCM = 5%): Rf = 0.37. **^{1}H NMR (700 MHz, DMSO-*d*$_6$)** δ 8.63 (br. s, 1H), 8.28 (br. s, 1H), 7.45 (d, *J* = 8.4 Hz, 2H), 7.40 (d, *J* = 8.1 Hz, 2H), 4.04 (t, *J* = 6.8 Hz, 2H), 3.59 (t, *J* = 6.5 Hz, 2H), 3.21 (d, *J* = 12.6 Hz, 2H), 3.11 – 3.07 (m, 2H), 3.07 – 3.03 (m, 2H), 2.96 (d, *J* = 7.3 Hz, 2H), 2.80 (q, *J* = 12.4 Hz, 2H), 2.56 (s, 3H), 2.49 (s, 3H), 2.39 (s, 3H), 1.83 – 1.72 (m, 5H), 1.68 (p, *J* = 6.7 Hz, 2H), 1.64 – 1.53 (m, 3H), 1.49 (p, *J* = 7.7 Hz, 2H), 1.34 – 1.27 (m, 2H), 1.24 – 1.15 (m, 5H). **^{13}C NMR (176 MHz, DMSO-*d*$_6$)** δ 157.8, 155.2, 141.1, 141.1, 139.4, 137.6, 135.9, 129.9, 125.7, 117.1, 116.5, 52.7, 49.1, 48.3, 48.1, 45.4, 42.8, 32.2, 31.6, 27.9, 27.0, 26.1, 23.6, 20.8, 20.2, 19.4, 11.9. **HRMS(ESI):** $[M+H]^+$ calcd. $C_{29}H_{44}ClN_6O_3S$ m/z 591.2879, found 591.2876.

***N*-(6-chlorohexyl)-4-(3,4-dimethyl-7-oxo-2-(*p*-tolyl)-2,7-dihydro-6*H*-pyrazolo[3,4-*d*]pyridazin-6-yl)-*N*-(piperidin-4-ylmethyl)butane-1-sulphonamide** (**5g**)

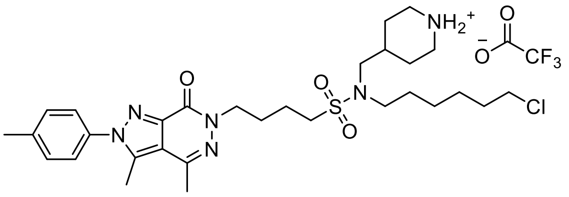

The title product compound **5g** (as a CF_3COO^- salt) was prepared by incubating **5d** (32.5 mg, 0.05 mmol, 1 equiv) with NaCl (14.6 mg, 0.25 mmol, 5 equiv) in DMSO at room temperature overnight, followed by purification by pre-HPLC as a white solid (28.8 mg, 0.04 mmol, 89%). TLC (MeOH/DCM = 5%): Rf = 0.46. **^{1}H NMR (500 MHz, DMSO-*d*$_6$)** δ 9.11 (br. s, 1H), 8.84 (br. s, 1H), 7.44 (d, *J* = 8.4 Hz, 2H), 7.39 (d, *J* = 8.2 Hz, 2H), 4.03 (t, *J* = 6.7 Hz, 2H), 3.56 (t, *J* = 6.6 Hz, 2H), 3.17 (d, *J* = 12.4 Hz, 2H), 3.12 – 3.00 (m, 4H), 2.94 (d, *J* = 7.3 Hz, 2H), 2.76 (t, *J* = 11.7 Hz, 2H), 2.55 (s, 3H), 2.48 (s, 3H), 2.38 (s, 3H), 1.83 – 1.69 (m, 5H), 1.69 – 1.55 (m, 4H), 1.46 (p, *J* = 7.7 Hz, 2H), 1.38 – 1.29 (m, 2H), 1.28 – 1.15 (m, 6H). **^{13}C NMR (126 MHz, DMSO-*d*$_6$)** δ 158.2, 155.3, 141.3, 141.1, 139.5, 137.7, 135.9, 130.0, 125.8, 117.2, 116.2, 52.7, 49.2, 48.4, 48.1, 45.4, 42.6, 32.3, 32.0, 28.5, 27.0, 26.1, 25.9, 25.6, 20.8, 20.2, 19.5, 12.0. **HRMS(ESI):** $[M+H]^+$ calcd. $C_{30}H_{46}ClN_6O_3S$ m/z 605.3035, found 605.3020.

***N*-(7-chloroheptyl)-4-(3,4-dimethyl-7-oxo-2-(*p*-tolyl)-2,7-dihydro-6*H*-pyrazolo[3,4-*d*]pyridazin-6-yl)-*N*-(piperidin-4-ylmethyl)butane-1-sulphonamide** (**5h**)

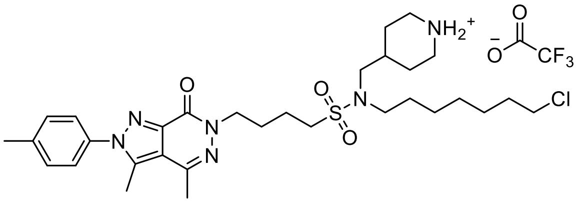

The title product compound **5h** (as a CF_3COO^- salt) was prepared by incubating **5e** (33.2 mg, 0.05 mmol, 1 equiv) with NaCl (14.6 mg, 0.25 mmol, 5 equiv) in DMSO at room temperature overnight, followed by purification by pre-HPLC as a white solid (28.7 mg, 0.05 mmol, 93%). TLC (MeOH/DCM = 5%): Rf = 0.58. **^{1}H NMR (500 MHz, DMSO-*d*$_6$)** δ 8.55 (br. s, 1H), 8.19 (br. s, 1H), 7.44 (d, *J* = 8.4 Hz, 2H), 7.39 (d, *J* = 8.4 Hz, 2H), 4.04 (t, *J* = 6.7 Hz, 2H), 3.57 (t, *J* = 6.6 Hz, 2H), 3.22 (d, *J* = 13.4 Hz, 2H), 3.11 – 3.01 (m, 4H), 2.96 (d, *J* = 7.1 Hz, 2H), 2.81 (q, *J* = 12.2 Hz, 2H), 2.56 (s, 3H), 2.48 (s, 3H), 2.39 (s, 3H), 1.85 – 1.71 (m, 5H), 1.69 – 1.55 (m, 4H), 1.46 (p, *J* = 7.8 Hz, 2H), 1.36 – 1.21 (m, 5H), 1.21 – 1.12 (m, 4H). **^{13}C NMR (126 MHz, DMSO-*d*$_6$)** δ 158.1, 155.3, 141.2, 141.1, 139.4, 137.6, 135.9, 129.9, 125.8, 117.1, 52.6, 49.2, 48.4, 48.1, 45.4, 42.9, 32.2, 32.0, 28.5, 27.8, 27.0, 26.2, 26.2, 26.2, 20.8, 20.2, 19.5, 11.9. **HRMS(ESI):** $[M+H]^+$ calcd. $C_{31}H_{48}ClN_6O_3S$ m/z 619.3192, found 619.3178.

***N*-(2-(2-bromoethoxy)ethyl)-4-(3,4-dimethyl-7-oxo-2-(*p*-tolyl)-2,7-dihydro-6*H*-pyrazolo[3,4-*d*]pyridazin-6-yl)-*N*-(piperidin-4-ylmethyl)butane-1-sulphonamide** (**5i**)

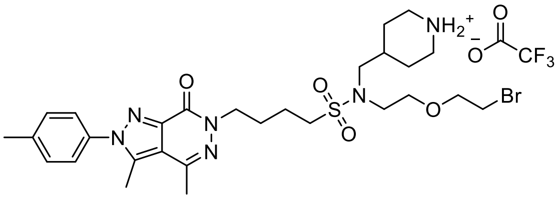

The title product compound **5i** (as a CF_3COO^- salt) was prepared using general procedure C from **S8b** (58.7 mg, 0.10 mmol, 1 equiv) and 2-bromoethyl ether (46.4 mg, 0.20 mmol, 2.0 equiv) as a white solid (30.1 mg, 0.04 mmol, 43% over two steps). TLC (MeOH/DCM = 5%): Rf = 0.16. **^{1}H NMR (700 MHz, DMSO-*d*$_6$)** δ 8.58 (br. s, 1H), 8.23 (br. s, 1H), 7.44 (d, *J* = 8.4 Hz, 2H), 7.39 (d, *J* = 8.0 Hz, 2H), 4.04 (t, *J* = 6.8 Hz, 2H), 3.70 (t, *J* = 5.5 Hz, 2H), 3.58 – 3.55 (m, 2H), 3.53 (t, *J* = 5.6 Hz, 2H), 3.30 (t, *J* = 5.5 Hz, 2H), 3.23 (d, *J* = 12.4 Hz, 2H), 3.17 – 3.12 (m, 2H), 3.02 (d, *J* = 7.4 Hz, 2H), 2.80 (q, *J* = 12.3 Hz, 2H), 2.55 (s, 3H), 2.48 (s, 3H), 2.38 (s, 3H), 1.89 – 1.83 (m, 1H), 1.83 – 1.73 (m, 4H), 1.62 (p, *J* = 7.8 Hz, 2H), 1.24 – 1.14 (m, 2H). **^{13}C NMR (176 MHz, DMSO-*d*$_6$)** δ 158.1, 155.3, 141.2, 141.1, 139.5, 137.6, 135.9, 130.0, 125.8, 117.2, 70.3, 68.3, 52.2, 49.6, 48.2, 47.1, 43.0, 32.4, 31.5, 27.1, 26.1, 20.8, 20.2, 19.5, 11.9. **HRMS(ESI):** $[M+H]^+$ calcd. $C_{28}H_{42}BrN_6O_4S$ m/z 637.2166, found 637.2166.

***N*-(2-(2-chloroethoxy)ethyl)-4-(3,4-dimethyl-7-oxo-2-(*p*-tolyl)-2,7-dihydro-6*H*-pyrazolo[3,4-*d*]pyridazin-6-yl)-*N*-(piperidin-4-ylmethyl)butane-1-sulphonamide** (**5j**)

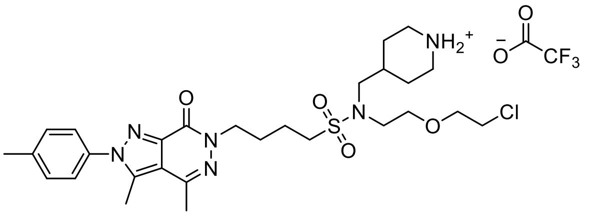

The title product compound **5j** (as a CF_3COO^- salt) was prepared by incubating **5i** (15.1 mg, 0.02 mmol, 1 equiv) with NaCl (5.8 mg, 0.10 mmol, 5 equiv) in DMSO at room temperature overnight, followed by purification by pre-HPLC as a white solid (10.7 mg, 0.015 mmol, 76%). TLC (MeOH/DCM = 5%): Rf = 0.18. **^{1}H NMR (500 MHz, DMSO-*d*$_6$)** δ 8.57 (br. s, 1H), 8.23 (br. s, 1H), 7.44 (d, *J* = 8.3 Hz, 2H), 7.39 (d, *J* = 8.2 Hz, 2H), 4.04 (t, *J* = 6.7 Hz, 2H), 3.72 – 3.66 (m, 2H), 3.66 – 3.60 (m, 1H), 3.59 – 3.50 (m, 3H), 3.30 (t, *J* = 5.5 Hz, 2H), 3.23 (d, *J* = 12.5 Hz, 2H), 3.18 – 3.11 (m, 2H), 3.04 – 2.97 (m, 2H), 2.80 (q, *J* = 10.7 Hz, 2H), 2.55 (s, 3H), 2.48 (s, 3H), 2.39 (s, 3H), 1.90 – 1.72 (m, 5H), 1.62 (p, *J* = 6.4, 4.7 Hz, 2H), 1.24 – 1.13 (m, 2H). **^{13}C NMR (126 MHz, DMSO-*d*$_6$)** δ 158.3, 155.3, 141.2, 141.1, 139.5, 137.6, 135.9, 130.0, 125.8, 117.2, 70.3, 68.3, 52.2, 49.6, 48.2, 47.1, 43.8, 43.0, 32.4, 27.1, 26.1, 20.8, 20.2, 19.5, 11.9. **HRMS(ESI):** $[M+H]^+$ calcd. $C_{28}H_{42}ClN_6O_4S$ m/z 593.2671, found 593.2662.

***N*-(2-(2-(2-bromoethoxy)ethoxy)ethyl)-4-(3,4-dimethyl-7-oxo-2-(*p*-tolyl)-2,7-dihydro-6*H*-pyrazolo[3,4-*d*]pyridazin-6-yl)-*N*-(piperidin-4-ylmethyl)butane-1- sulphonamide** (**5k**)

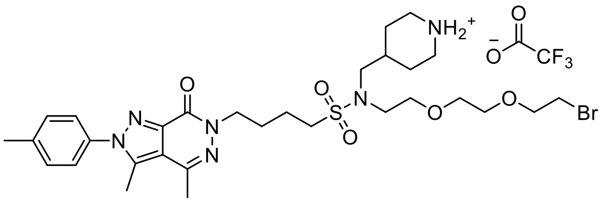

The title product compound **5k** (as a CF_3COO^- salt) was prepared using general procedure C from **S8b** (21.5 mg, 0.04 mmol, 1 equiv) and 1,2-bis(2-bromoethoxy)ethane (19.3 mg, 0.07 mmol, 2.0 equiv) as a yellow oil (23.9 mg, 0.03 mmol, 95% over two steps). TLC (MeOH/DCM = 5%): Rf = 0.32. **^{1}H NMR (500 MHz, DMSO-*d*$_6$)** δ 8.59 (br. s, 1H), 8.25 (br. s, 1H), 7.48 (d, *J* = 8.4 Hz, 2H), 7.43 (d, *J* = 8.3 Hz, 2H), 4.08 (t, *J* = 6.8 Hz, 2H), 3.72 (t, *J* = 5.7 Hz, 2H), 3.58 – 3.52 (m, 8H), 3.32 (t, *J* = 5.6 Hz, 2H), 3.27 (d, *J* = 12.1 Hz, 2H), 3.21 – 3.14 (m, 2H), 3.05 (d, *J* = 7.4 Hz, 2H), 2.83 (q, *J* = 12.1 Hz, 2H), 2.59 (s, 3H), 2.52 (s, 3H), 2.43 (s, 3H), 1.93 – 1.74 (m, 5H), 1.70 – 1.61 (m, 2H), 1.28 – 1.16 (m, 2H). **^{13}C NMR (126 MHz, DMSO-*d*$_6$)** δ 158.2, 155.3, 141.2, 141.1, 139.5, 137.6, 135.9, 130.0, 125.8, 117.2, 70.4, 69.6, 69.6, 68.5, 52.1, 49.6, 48.2, 47.1, 43.0, 32.3, 31.5, 27.1, 26.0, 20.8, 20.2, 19.5, 11.9. **HRMS(ESI):** $[M+H]^+$ calcd. $C_{30}H_{46}BrN_6O_5S$ m/z 681.2428, found 681.2411.

***N*-(2-(2-(2-chloroethoxy)ethoxy)ethyl)-4-(3,4-dimethyl-7-oxo-2-(*p*-tolyl)-2,7-dihydro-6*H*-pyrazolo[3,4-*d*]pyridazin-6-yl)-*N*-(piperidin-4-ylmethyl)butane-1-sulphonamide** (**5l**)

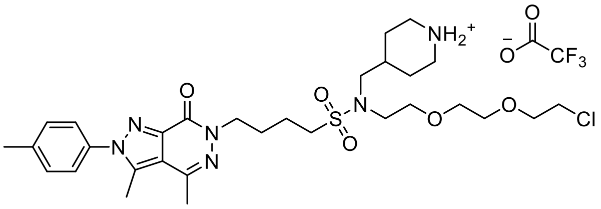

The title product compound **5l** was prepared using general procedure C from **S8b** (29.3 mg, 0.05 mmol, 1 equiv) and 1,2-bis(2-chloroethoxy) -ethane (18.7 mg, 0.10 mmol, 2.0 equiv) as a white solid (17.6 mg, 0.03 mmol, 55% over two steps). TLC (MeOH/DCM = 5%): Rf = 0.35. **^{1}H NMR (500 MHz, DMSO-*d*$_6$)** δ 8.43 (br. s, 1H), 8.09 (br. s, 1H), 7.45 (d, *J* = 8.4 Hz, 2H), 7.40 (d, *J* = 8.4 Hz, 2H), 4.04 (t, *J* = 6.8 Hz, 2H), 3.68 – 3.64 (m, 2H), 3.64 – 3.59 (m, 2H), 3.54 – 3.47 (m, 7H), 3.27 (d, *J* = 5.8 Hz, 3H), 3.23 (d, *J* = 12.0 Hz, 3H), 3.17 – 3.10 (m, 2H), 3.01 (d, *J* = 7.4 Hz, 2H), 2.85 – 2.73 (m, 2H), 2.56 (s, 3H), 2.49 (s, 3H), 2.39 (s, 3H), 1.87 – 1.71 (m, 5H), 1.62 (dq, *J* = 10.1, 7.5 Hz, 2H), 1.24 – 1.11 (m, 3H). **^{13}C NMR (126 MHz, DMSO-*d*$_6$)** δ 155.2, 141.2, 141.1, 139.4, 137.6, 135.9, 129.9, 125.8, 117.1, 70.5, 69.7, 69.6, 68.5, 52.1, 49.6, 48.2, 47.1, 43.6, 43.0, 31.5, 27.1, 26.0, 20.8, 20.2, 19.5, 11.9. **HRMS(ESI):** [M+H]$^+$ calcd. $C_{30}H_{46}ClN_6O_5S$ m/z 637.2933, found 637.2933.

***N*-(3-(2-bromoethyl)phenethyl)-4-(3,4-dimethyl-7-oxo-2-(*p*-tolyl)-2,7-dihydro-6*H*-pyrazolo[3,4-*d*]pyridazin-6-yl)-*N*-(piperidin-4-ylmethyl)butane-1-sulphonamide** (**6a**)

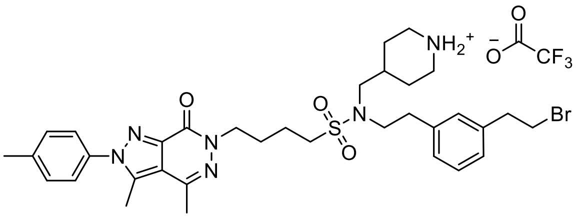

To 1,3-phenylenediacetic acid (1 g, 5.15 mmol, 1 equiv) in dry THF was added $LiAlH_4$ (2 M in THF, 5.15 ml, 10.3 mmol, 2 equiv) and the reaction mixture was stirred at room temperature overnight. The crude residue was redissolved in ethyl acetate and washed sequentially with water and brine, then dried with $MgSO_4$, filtered and concentrated *in vacuo.* To the obtained diol was redissolved in HBr in water and the reaction was refluxed overnight, and then extracted with ethyl acetate, washed sequentially with water and brine and dried with $MgSO_4$, filtered and concentrated *in vacuo* to obtain the desired dibromoalkane, where the crude was used without further purification. The title product compound **6a** (as a CF_3COO^- salt) was prepared using general procedure C from **S8b** (36.4 mg, 0.06 mmol, 1 equiv) and the desired dibromoalkane (36.2 mg, 0.12 mmol, 2.0 equiv) as a yellow oil (25.7 mg, 0.04 mmol, 59% over two steps). TLC (MeOH/DCM = 5%): Rf = 0.22. **^{1}H NMR (700 MHz, DMSO-*d*$_6$)** δ 8.50 (br. s, 1H), 8.14 (br. s, 1H), 7.48 (d, *J* = 8.3 Hz, 2H), 7.43 (d, *J* = 8.3 Hz, 2H), 7.37 – 7.10 (m, 4H), 4.06 (t, *J* = 6.8 Hz, 2H), 3.71 (t, *J* = 7.3 Hz, 2H), 3.38 – 3.34 (m, 2H), 3.26 (d, *J* = 11.8 Hz, 2H), 3.12 – 3.01 (m, 5H), 2.90 – 2.76 (m, 5H), 2.58 (s, 3H), 2.54 (s, 3H), 2.43 (s, 3H), 1.82 – 1.74 (m, 4H), 1.63 – 1.60 (m, 2H), 1.30 – 1.23 (m, 4H). **^{13}C NMR (176 MHz, DMSO-*d*$_6$)** δ 158.0, 155.2, 141.1, 141.0, 139.4, 139.4, 139.3, 137.5, 135.9, 129.9, 129.3,

128.9, 126.7, 125.7, 124.2, 117.1, 115.1, 52.3, 49.3, 48.1, 48.1, 42.9, 40.4, 38.3, 34.7, 34.4, 31.7, 26.9, 26.1, 21.3, 20.7, 20.1, 19.4, 11.9. **HRMS(ESI):** $[M+H]^+$ calcd. $C_{34}H_{46}BrN_6O_3S$ m/z 697.2530, found 697.2533.

***N*-(3-(2-chloroethyl)phenethyl)-4-(3,4-dimethyl-7-oxo-2-(*p*-tolyl)-2,7-dihydro-6*H*-pyrazolo[3,4-*d*]pyridazin-6-yl)-*N*-(piperidin-4-ylmethyl)butane-1-sulphonamide** (**6b**)

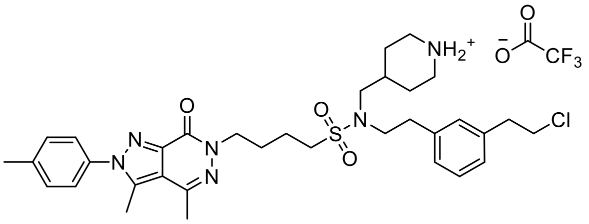

The title product compound **6b** (as a CF_3COO^- salt) was prepared by incubating **6a** (10 mg, 0.01 mmol, 1 equiv) with NaCl (4.2 mg, 0.07 mmol, 5 equiv) in DMSO at room temperature overnight, followed by purification by pre-HPLC as a white solid (7.7 mg, 0.01 mmol, 83%). TLC (MeOH/DCM = 5%): Rf = 0.15. **^{1}H NMR (700 MHz, DMSO-*d*$_6$)** δ 8.54 (br. s, 1H), 8.18 (br. s, 1H), 7.48 (d, *J* = 8.3 Hz, 2H), 7.43 (d, *J* = 8.3 Hz, 2H), 7.30 – 7.13 (m, 4H), 4.06 (t, *J* = 6.7 Hz, 2H), 3.83 (t, *J* = 7.1 Hz, 2H), 3.39 – 3.34 (m, 2H), 3.26 (d, *J* = 11.7 Hz, 2H), 3.06 – 2.98 (m, 5H), 2.92 – 2.76 (m, 5H), 2.58 (s, 3H), 2.54 (s, 3H), 2.43 (s, 3H), 1.85 – 1.73 (m, 4H), 1.63 – 1.59 (m, 2H), 1.26 – 1.21 (m, 4H). **^{13}C NMR (176 MHz, DMSO-*d*$_6$)** δ 158.0, 155.2, 141.1, 141.0, 139.4, 138.3, 137.6, 137.0, 135.9, 129.9, 129.4, 128.6, 128.4, 127.3, 125.7, 117.1, 116.1, 52.3, 49.3, 48.1, 45.7, 45.3, 42.9, 40.4, 38.1, 33.5, 31.8, 26.9, 26.1, 21.3, 20.7, 20.2, 19.4, 11.9. **HRMS(ESI):** $[M+H]^+$ calcd. $C_{34}H_{46}ClN_6O_3S$ m/z 653.3035, found 653.3039.

Ethyl 4-((2-((3-bromopropyl)amino)-3,4-dioxocyclobut-1-en-1-yl)amino)piperidine-1-carboxylate (7)

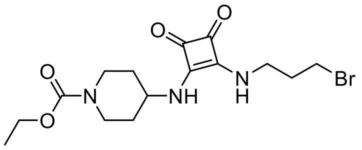

The title product compound **7** was prepared from the reaction between **S9** (50 mg, 0.17 mmol) and 3-bromopropylamine hydrobromide (41 mg, 0.19 mmol), with triethylamine (10 eqv.) in EtOH (5 ml) at room temperature overnight, followed by purification by column chromatography as an off-white solid (50.4 mg, 0.13 mmol, 77%). TLC (EtOAc 100%): Rf = 0.23. **^{1}H NMR (700 MHz, DMSO-*d*$_6$)** δ 7.78 (d, *J* = 7.6 Hz, 1H), 6.97 (t, *J* = 5.1 Hz, 1H), 4.43 – 4.40 (m, 2H), 4.02 – 3.96 (m, 6H), 3.93 – 3.90 (m, 2H), 3.13 – 3.10 (m, 1H), 2.12 – 2.06 (m, 2H), 2.05 – 1.92 (m, 2H), 1.68 – 1.64 (m, 2H), 1.09 (t, *J* = 7.1 Hz, 3H). **^{13}C NMR (176 MHz, DMSO-*d*$_6$)** δ 188.6, 182.1, 177.5, 173.5, 154.7, 60.9, 50.4, 46.5, 41.9, 35.9, 32.7, 30.4, 14.7. **HRMS(APCI):** $[M+H]^+$ calcd. $C_{15}H_{23}BrN_3O_4$ m/z 388.0866, found 388.0872.

4. Supplementary Figures for Chemical Synthesis: Compound NMR Spectra

^{1}H NMR Spectrum of **S1** (700 MHz, $CDCl_3$)

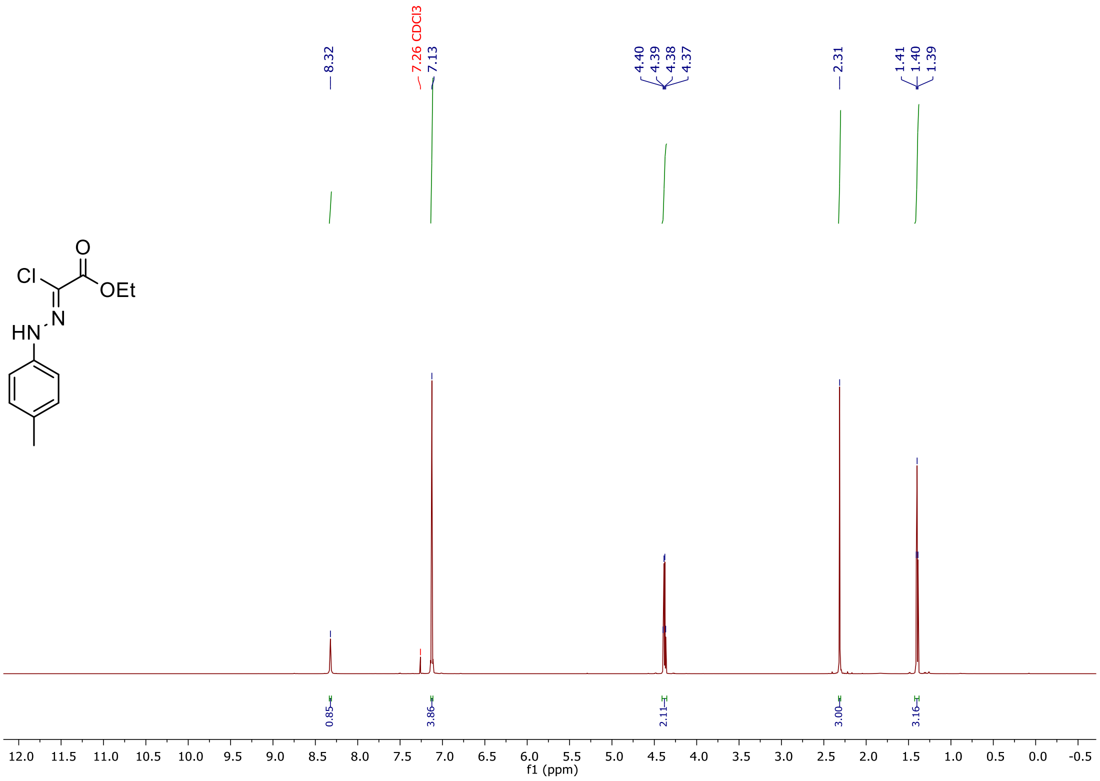

^{13}C NMR Spectrum of **S1** (176 MHz, $CDCl_3$)

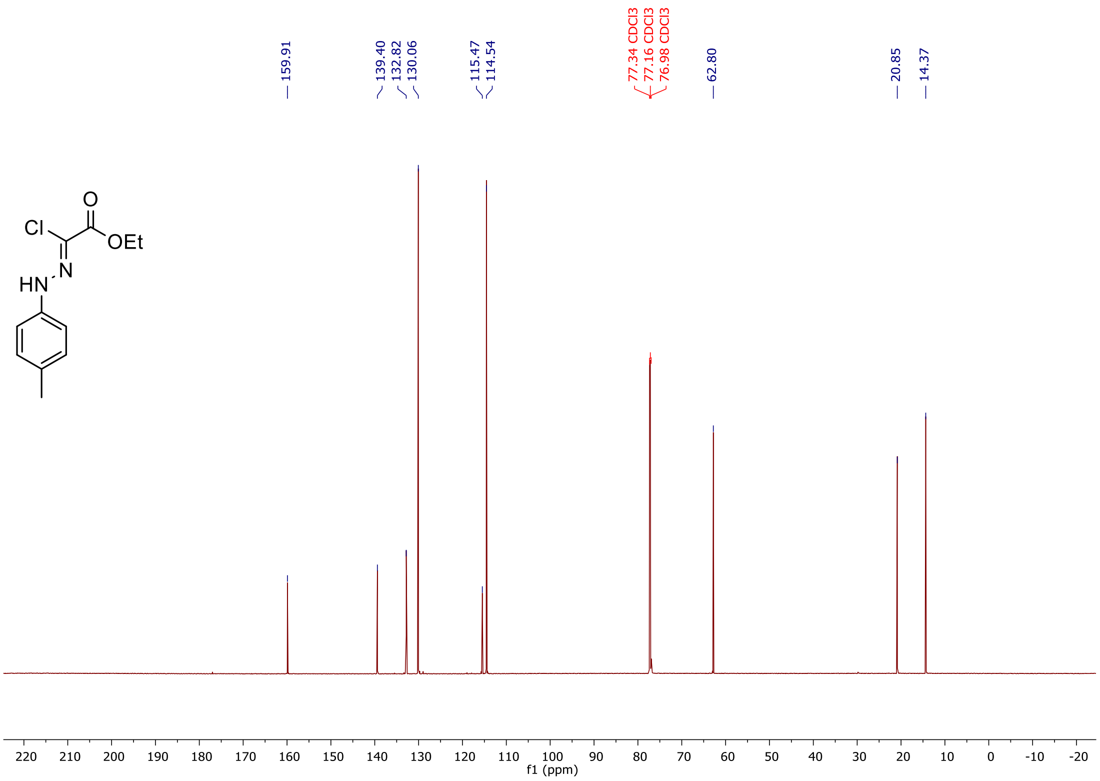

¹H NMR Spectrum of **S2** (700 MHz, DMSO-d_6)

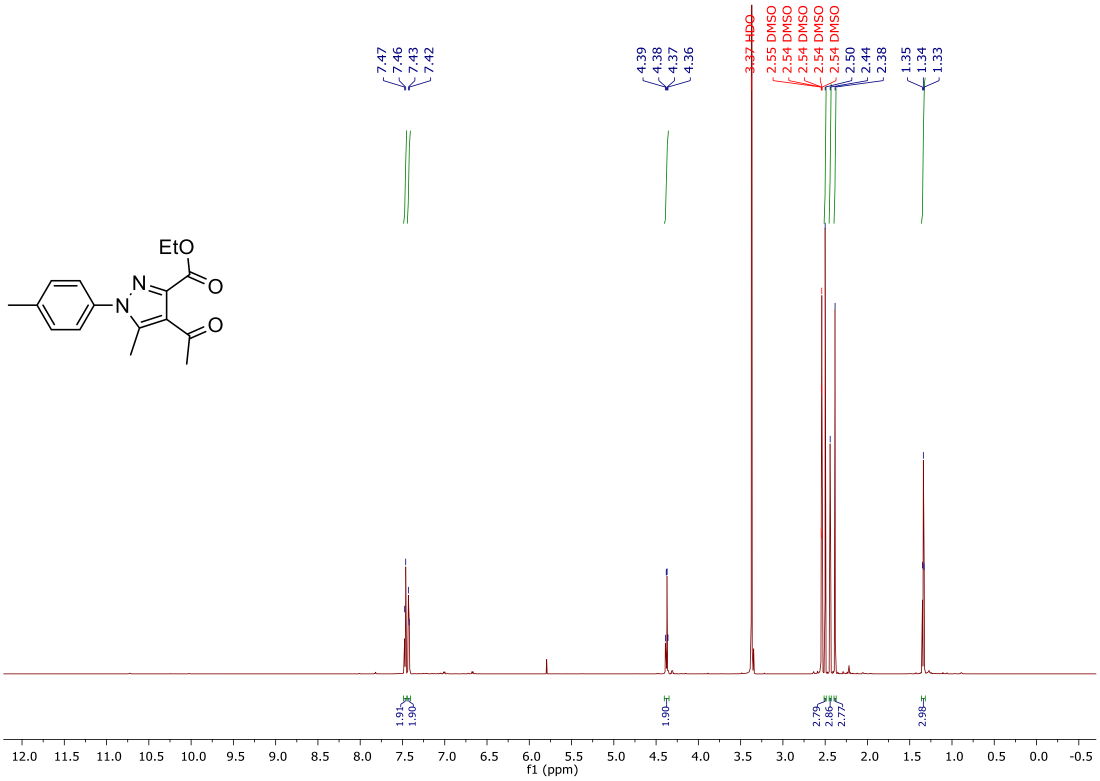

¹³C NMR Spectrum of **S2** (176 MHz, DMSO-d_6)

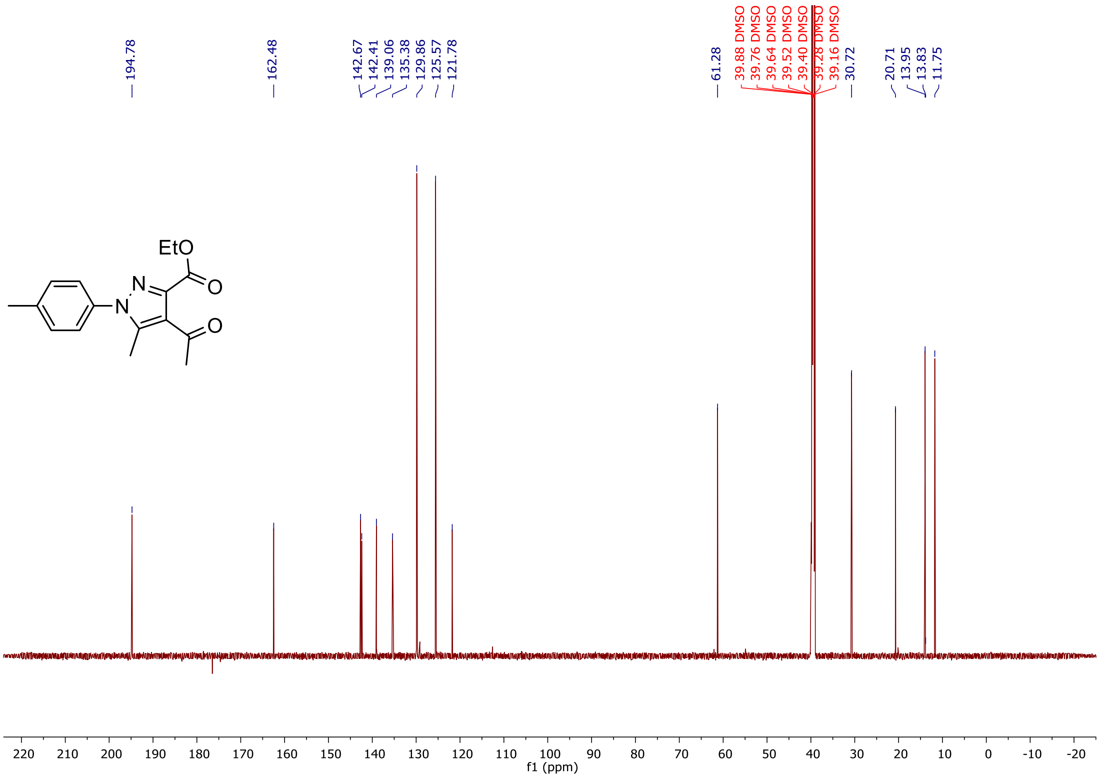

^{1}H NMR Spectrum of **S3** (700 MHz, DMSO-d_6)

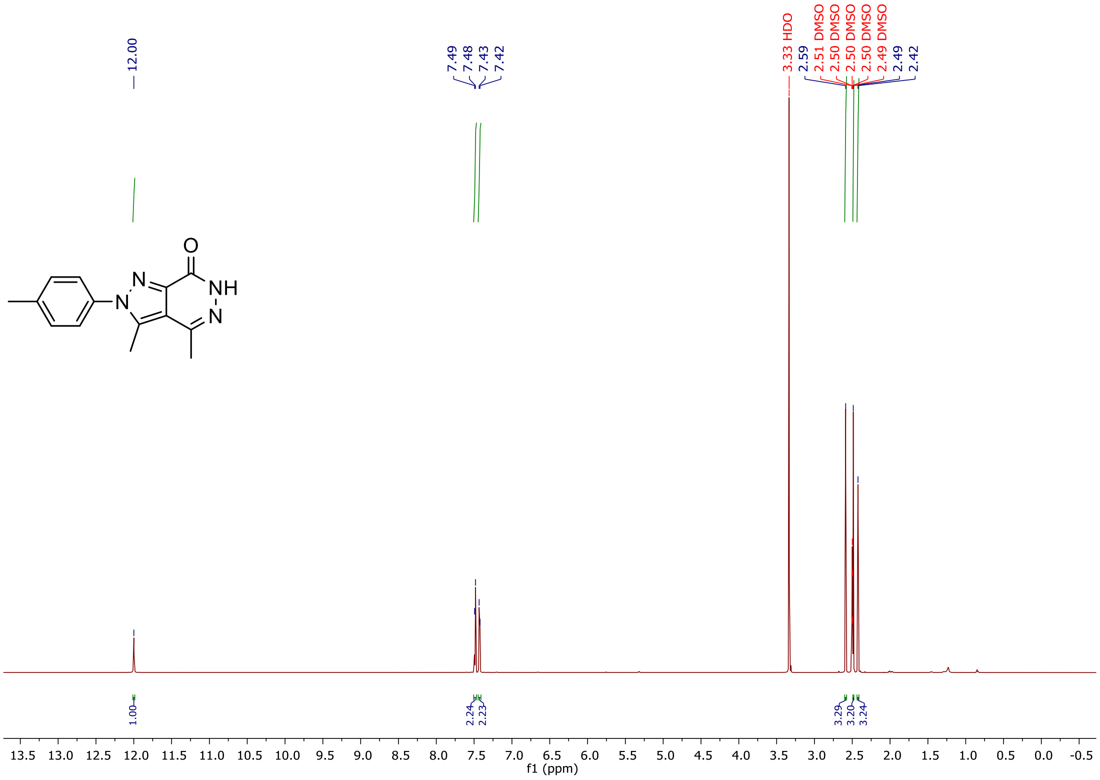

^{13}C NMR Spectrum of **S3** (176 MHz, DMSO-d_6)

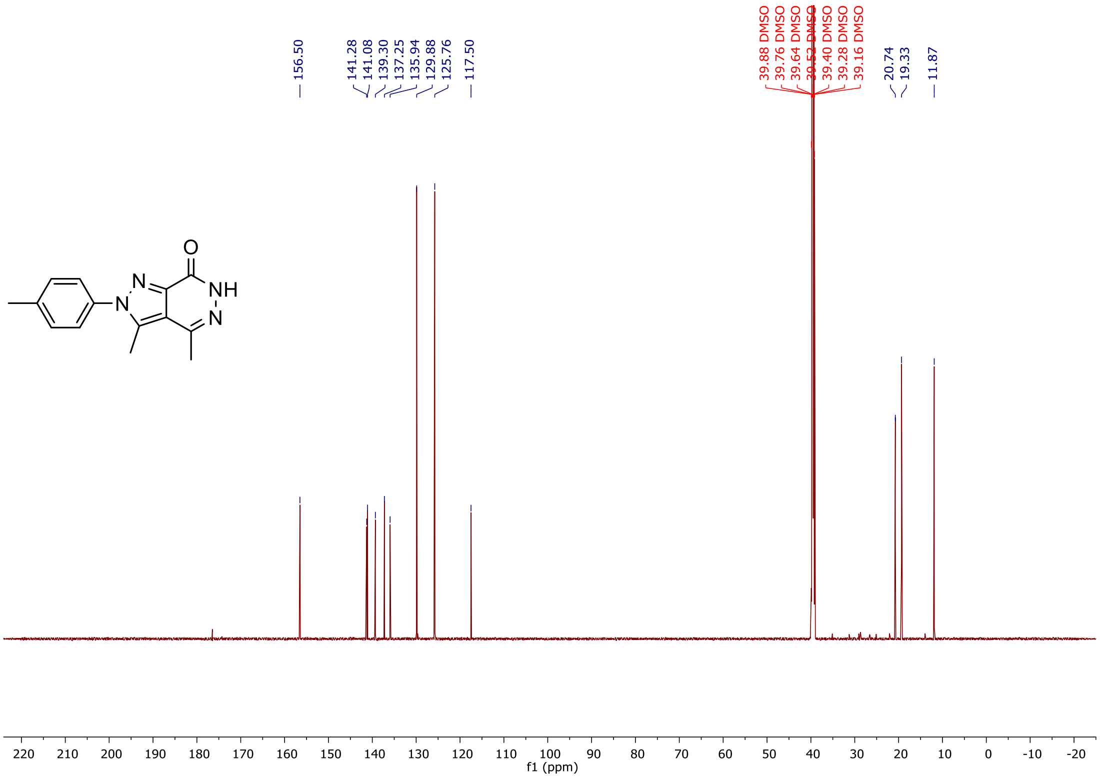

^{1}H NMR Spectrum of **S4** (700 MHz, $CDCl_3$)

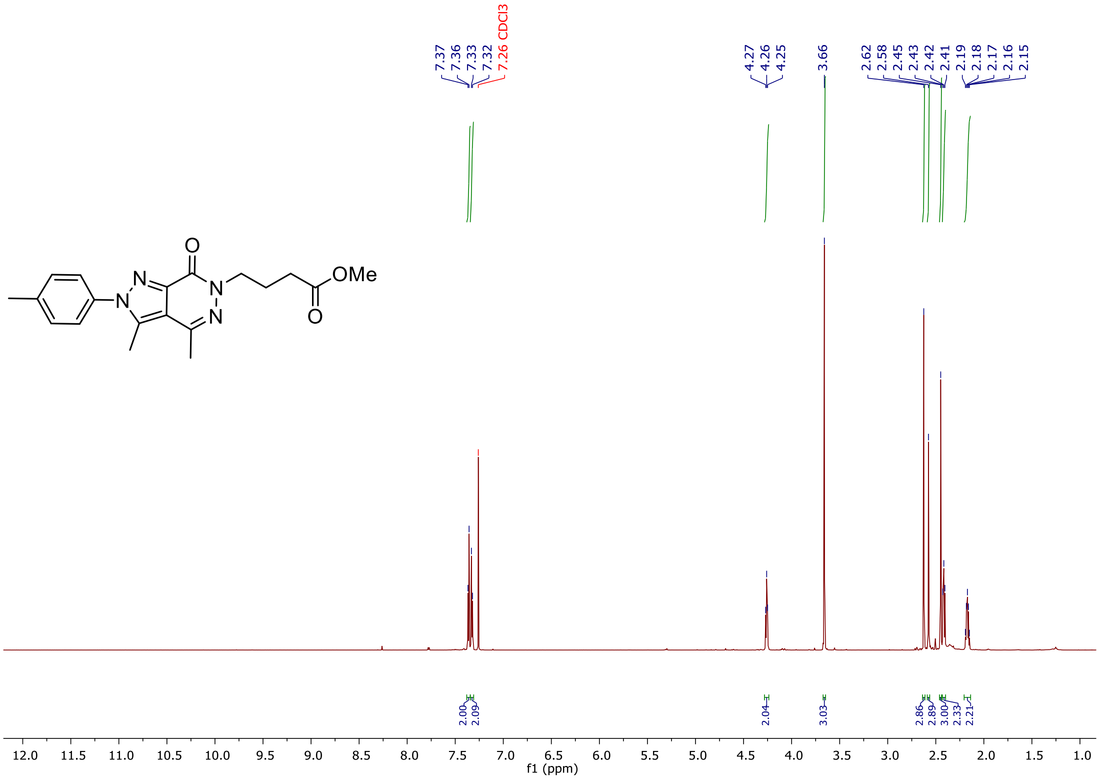

^{13}C NMR Spectrum of **S4** (176 MHz, $CDCl_3$)

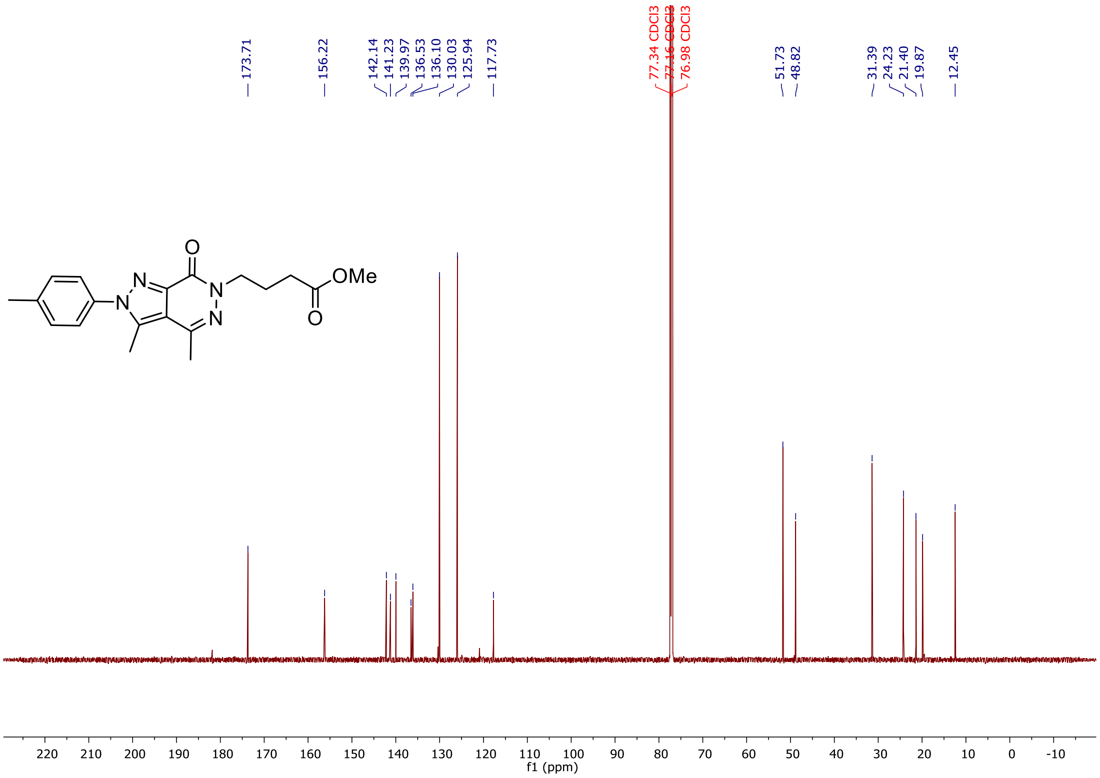

^{1}H NMR Spectrum of **S5** (700 MHz, DMSO-d_6)

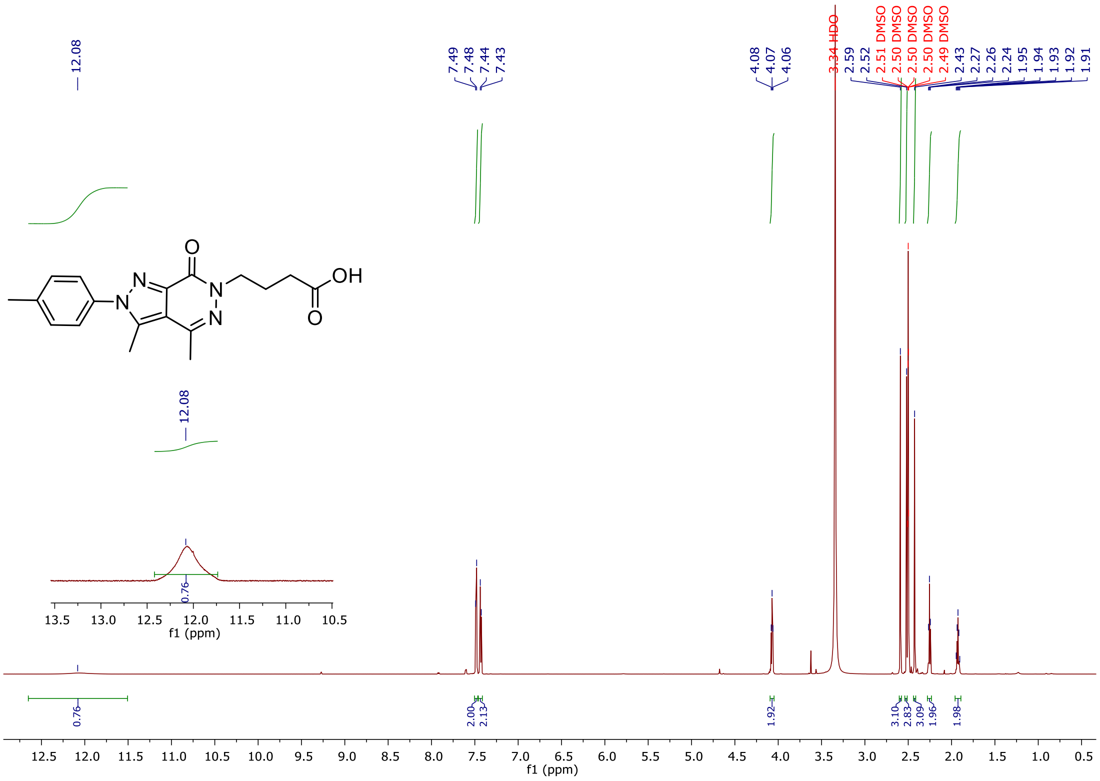

^{13}C NMR Spectrum of **S5** (176 MHz, DMSO-d_6)

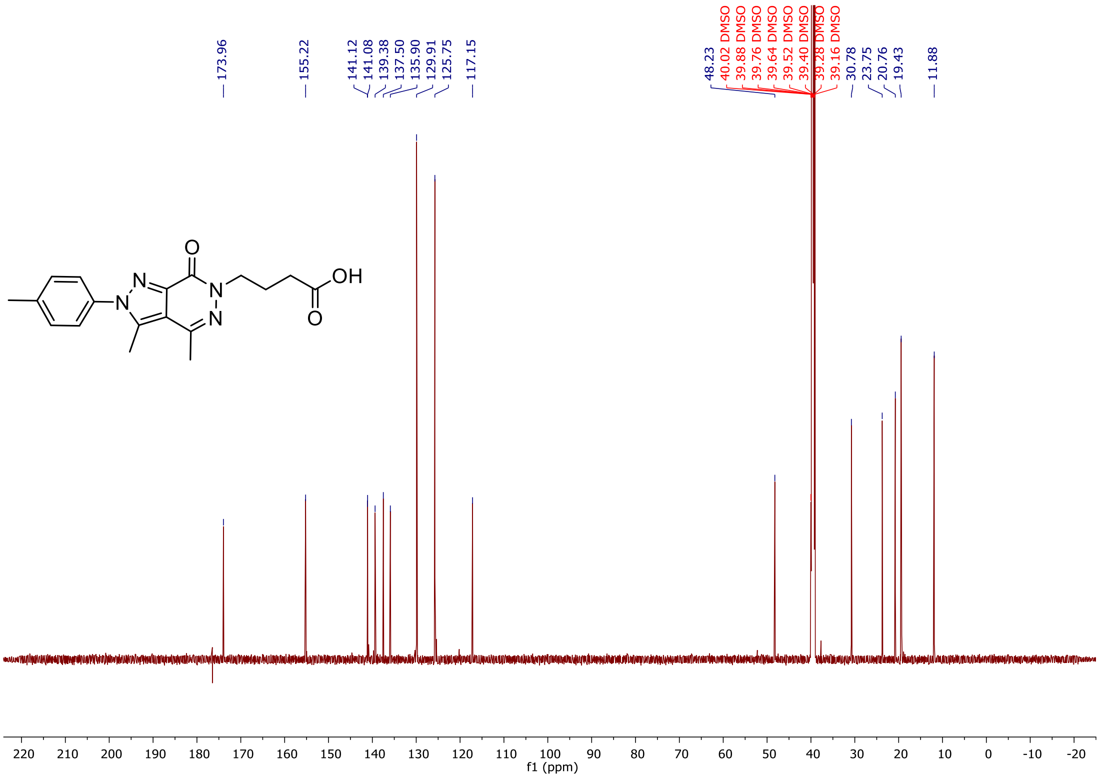

^{1}**H NMR** Spectrum of **S6a** (700 MHz, DMSO-d_6)

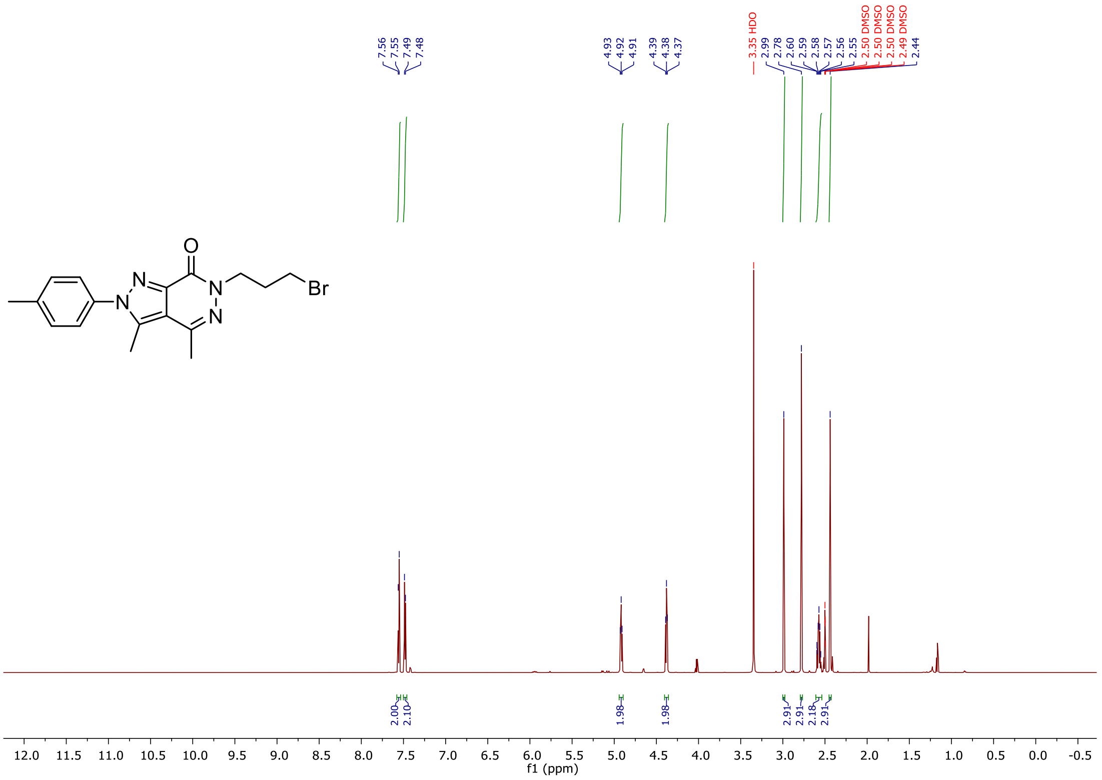

^{13}**C NMR** Spectrum of **S6a** (176 MHz, DMSO-d_6)

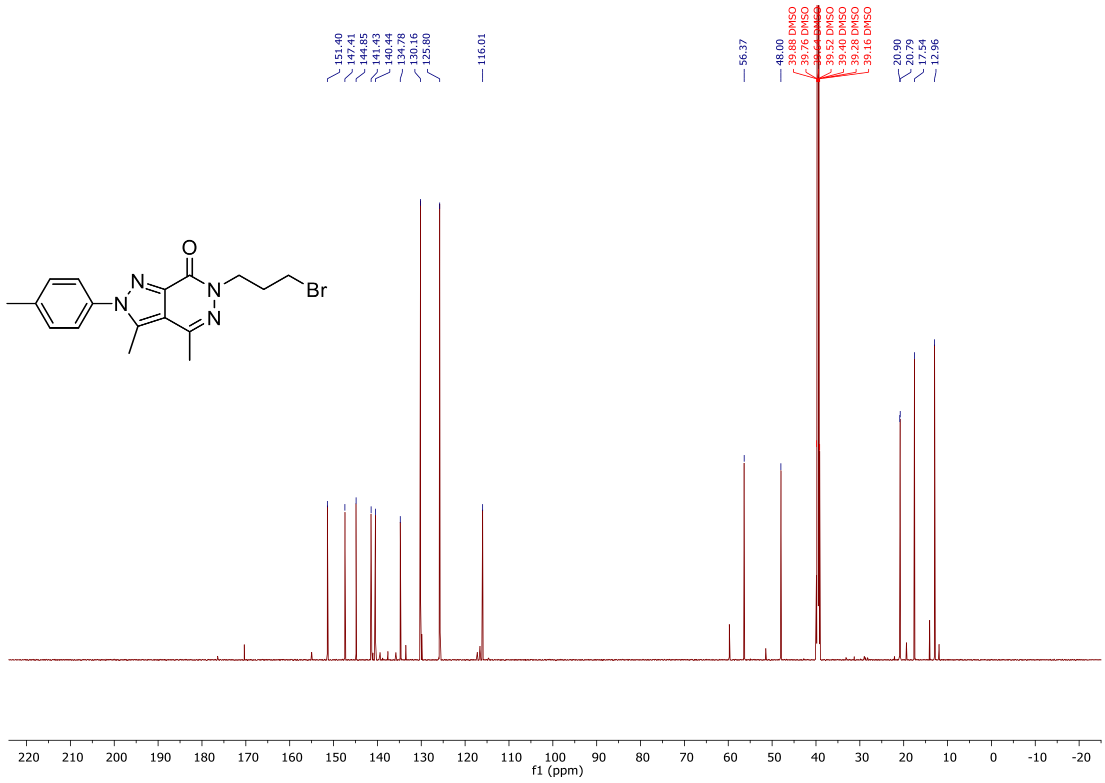

^{1}H NMR Spectrum of **S6b** (500 MHz, DMSO-d_6)

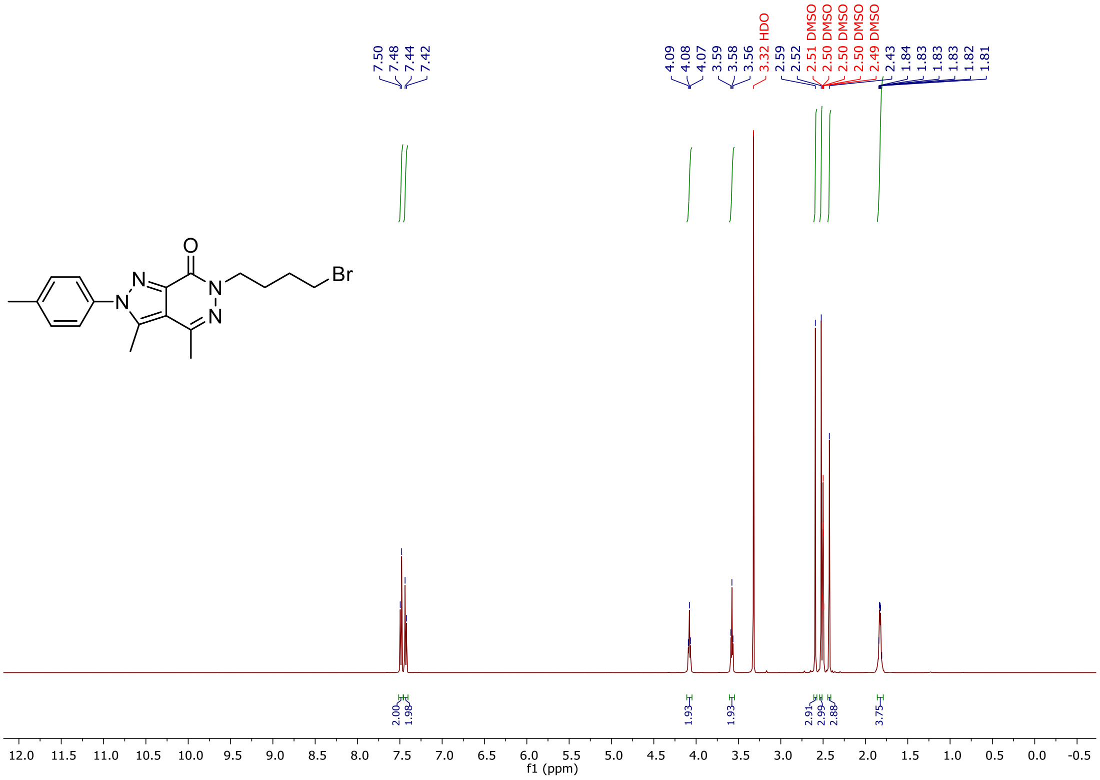

^{13}C NMR Spectrum of **S6b** (126 MHz, DMSO-d_6)

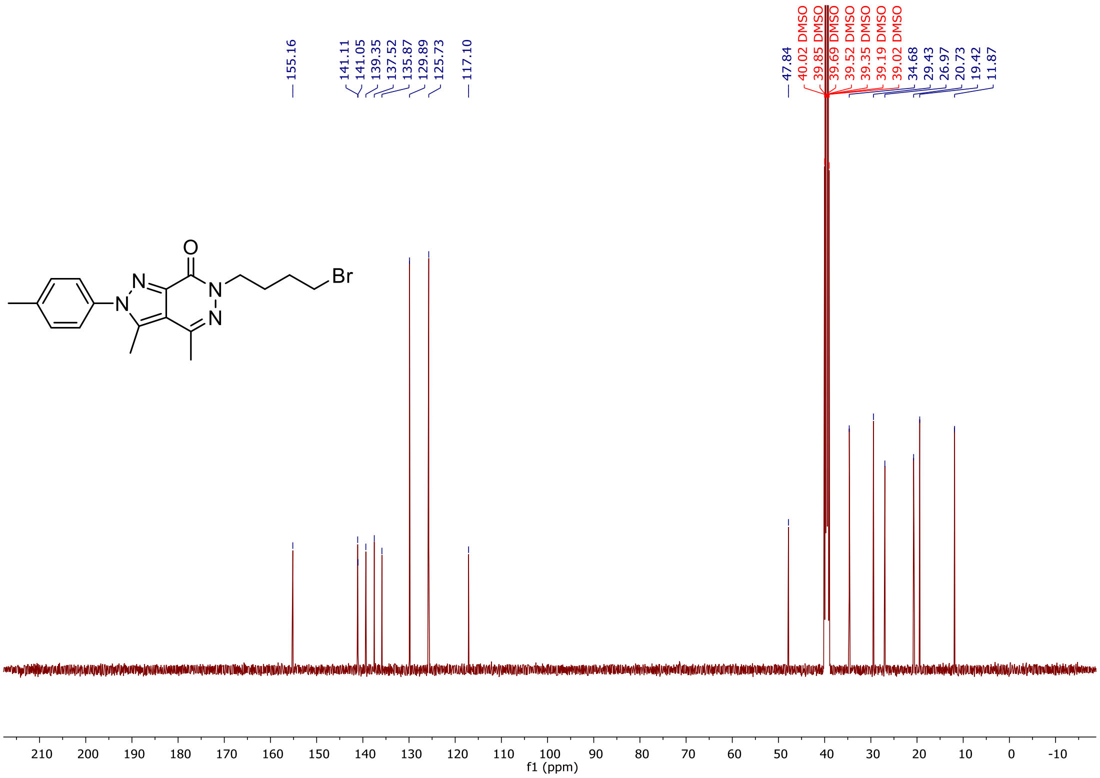

¹H NMR Spectrum of **S6c** (500 MHz, DMSO-d_6)

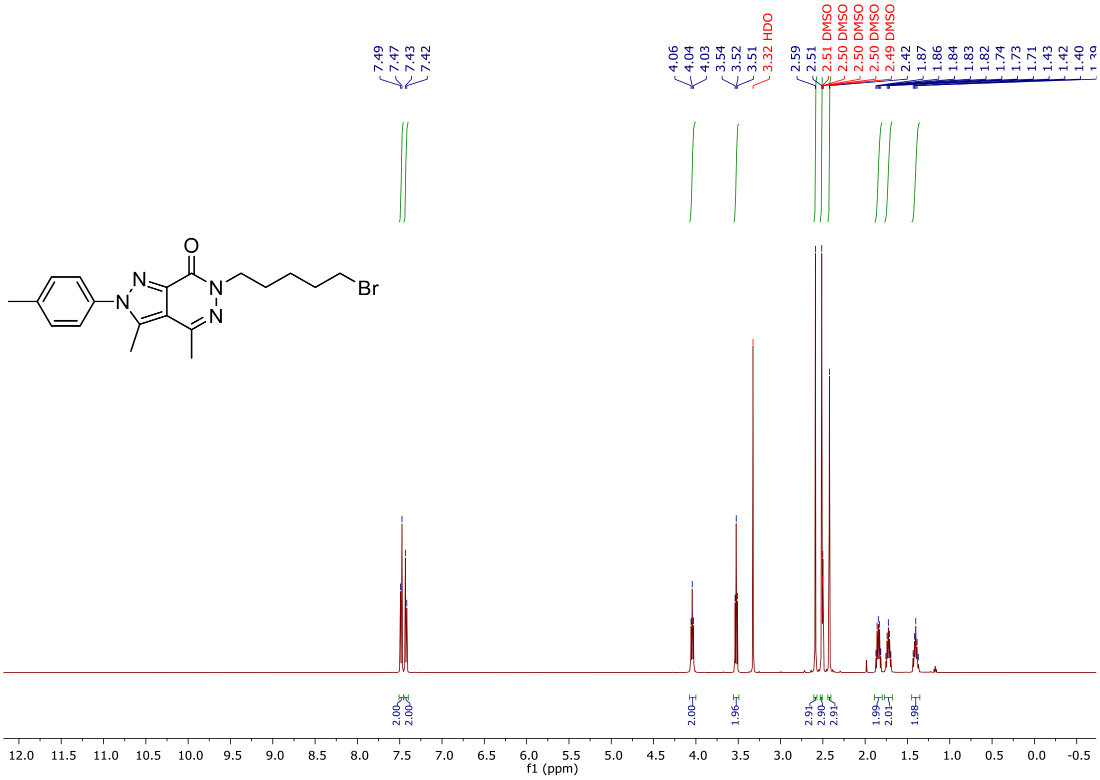

¹³C NMR Spectrum of **S6c** (126 MHz, DMSO-d_6)

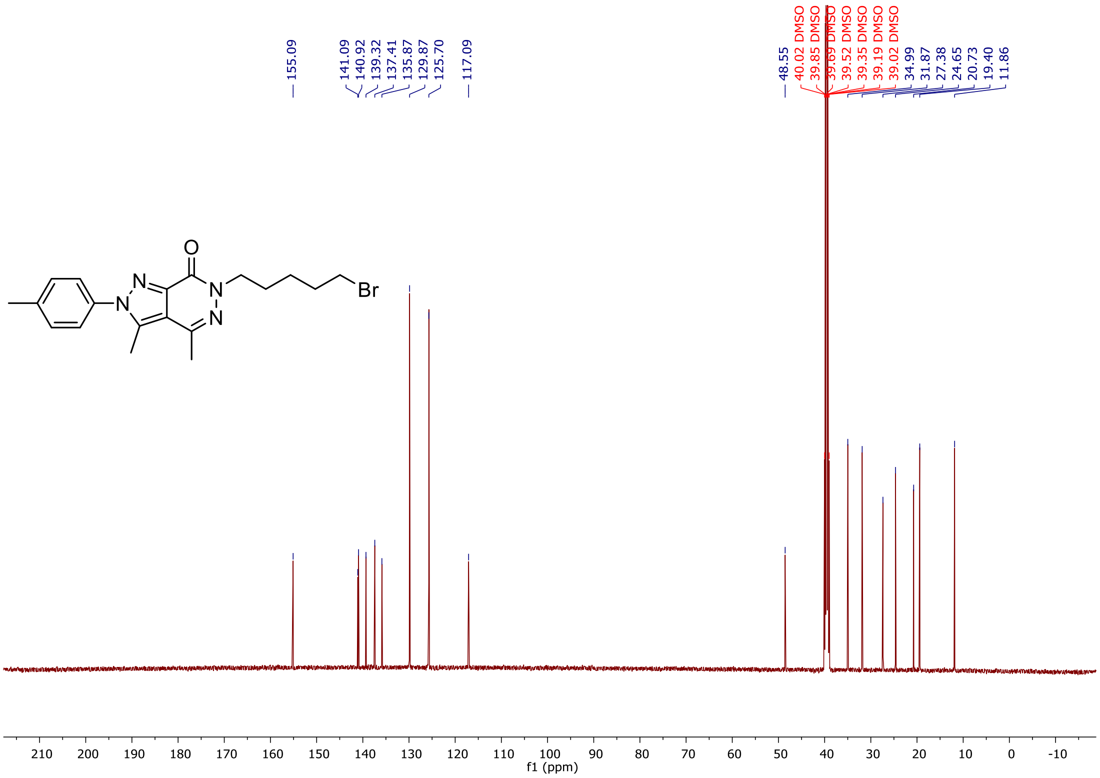

^{1}H NMR Spectrum of **S6d** (500 MHz, DMSO-d_6)

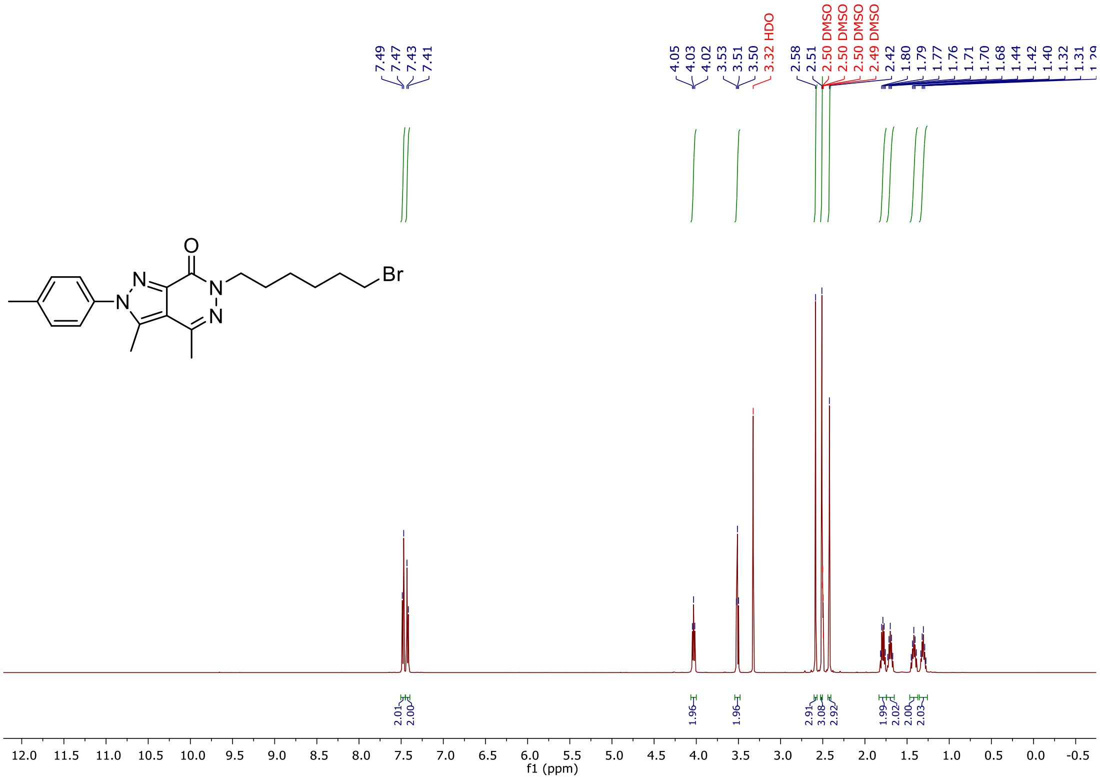

^{13}C NMR Spectrum of **S6d** (126 MHz, DMSO-d_6)

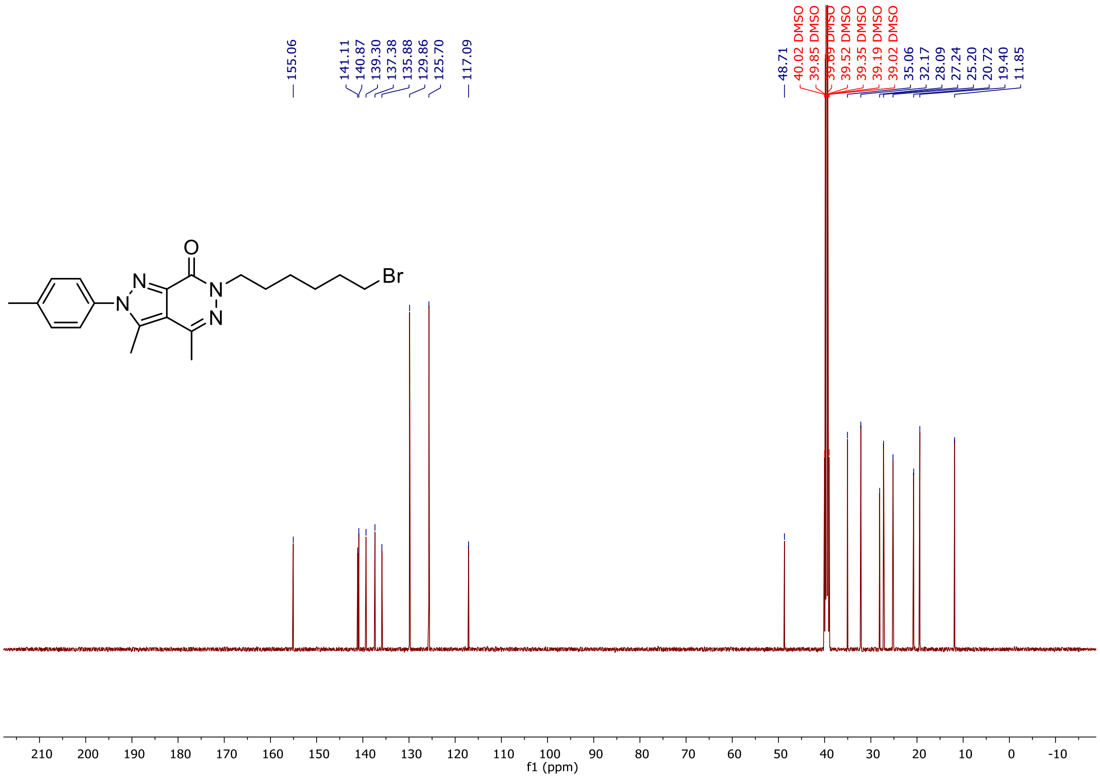

^{1}H NMR Spectrum of **S7a** (500 MHz, $CDCl_3$)

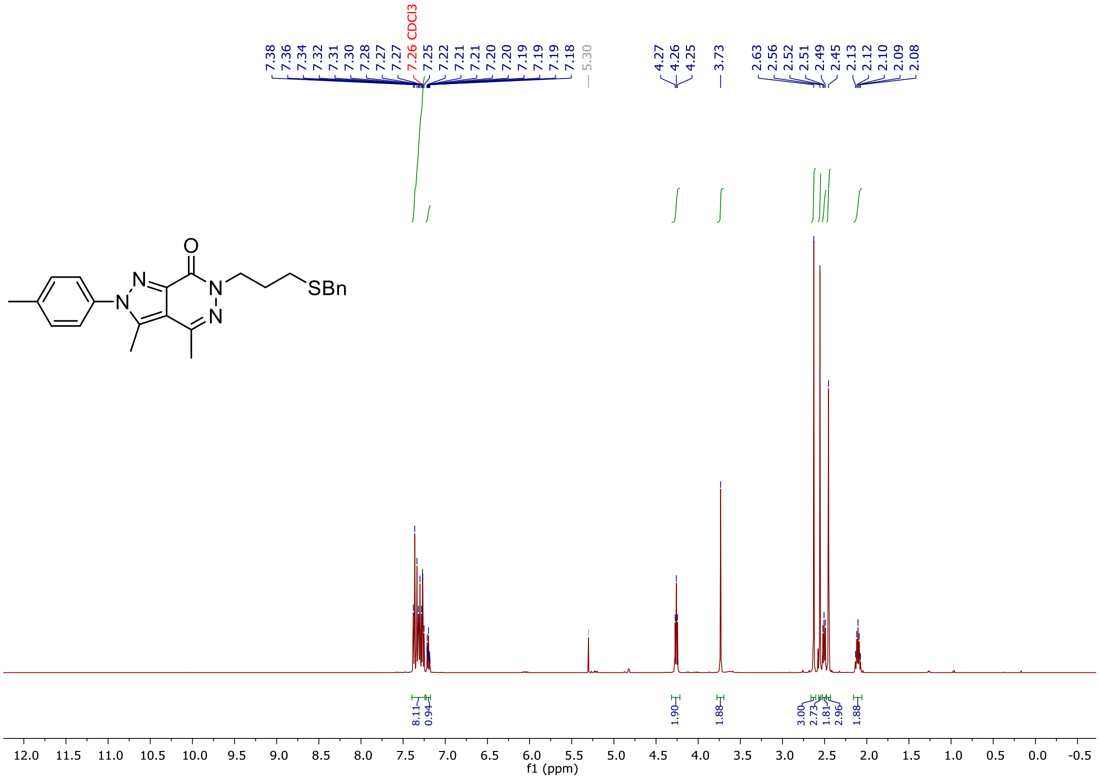

^{13}C NMR Spectrum of **S7a** (126 MHz, $CDCl_3$)

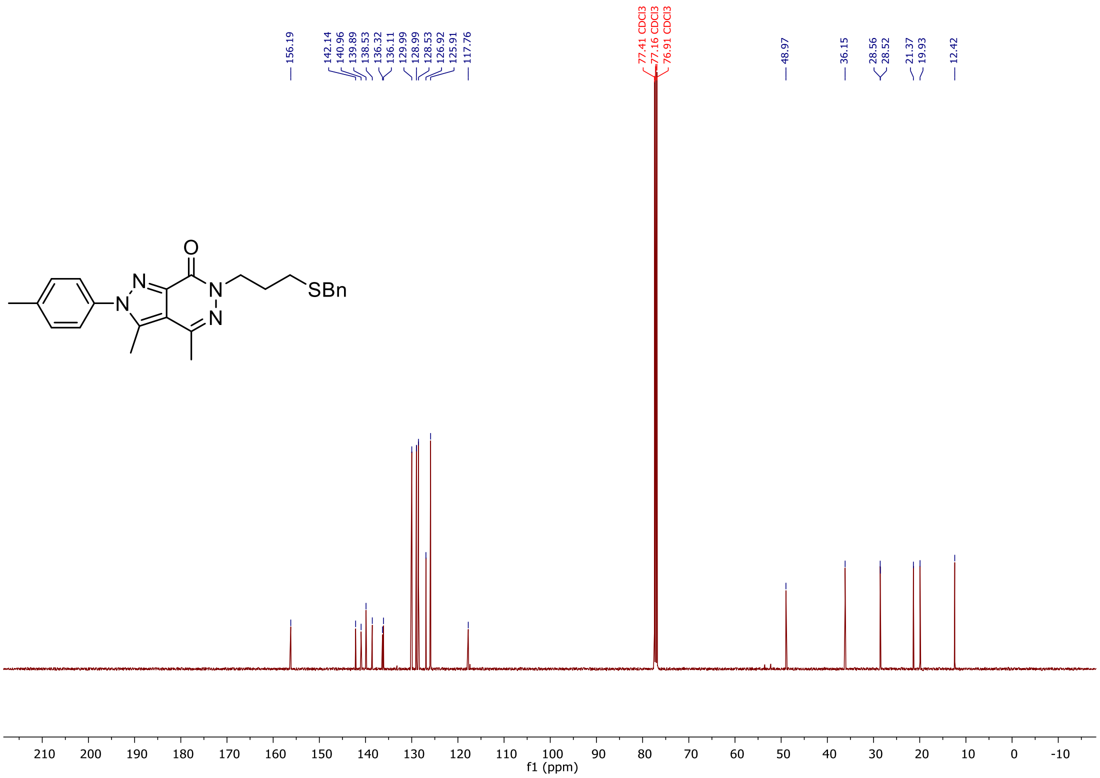

^{1}H NMR Spectrum of **S7b** (600 MHz, DMSO-d_6)

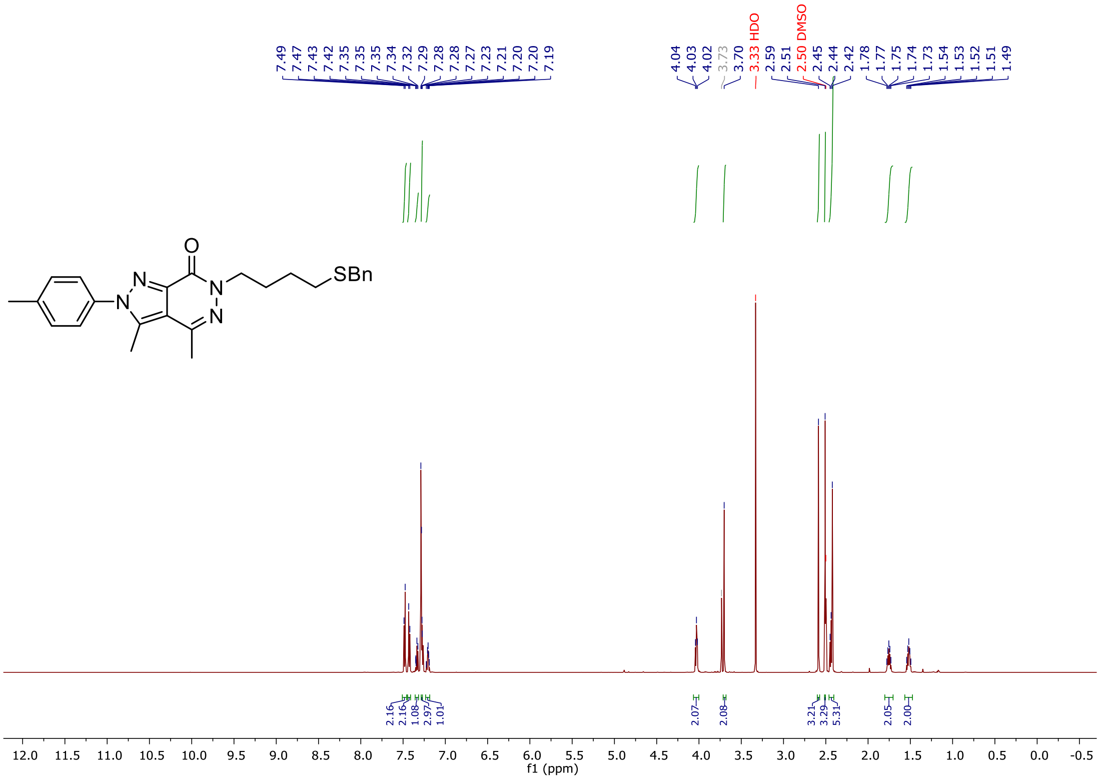

^{13}C NMR Spectrum of **S7b** (151 MHz, DMSO-d_6)

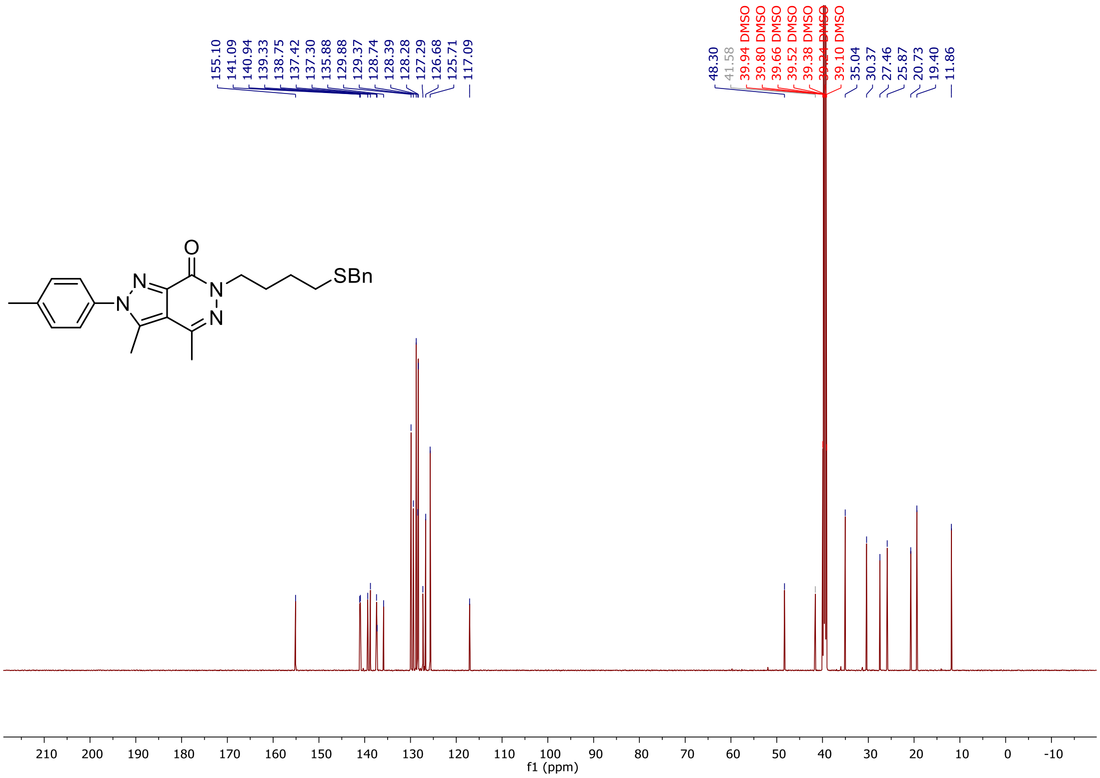

^{1}H NMR Spectrum of **S7c** (600 MHz, DMSO-d_6)

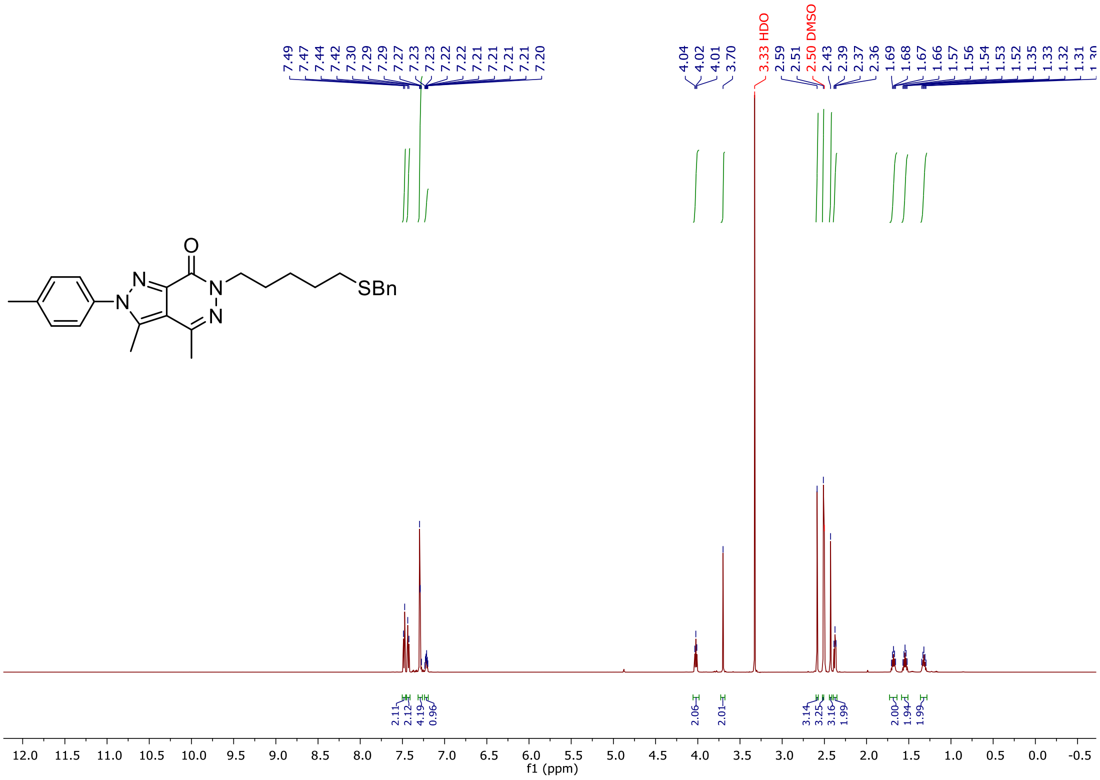

^{13}C NMR Spectrum of **S7c** (151 MHz, DMSO-d_6)

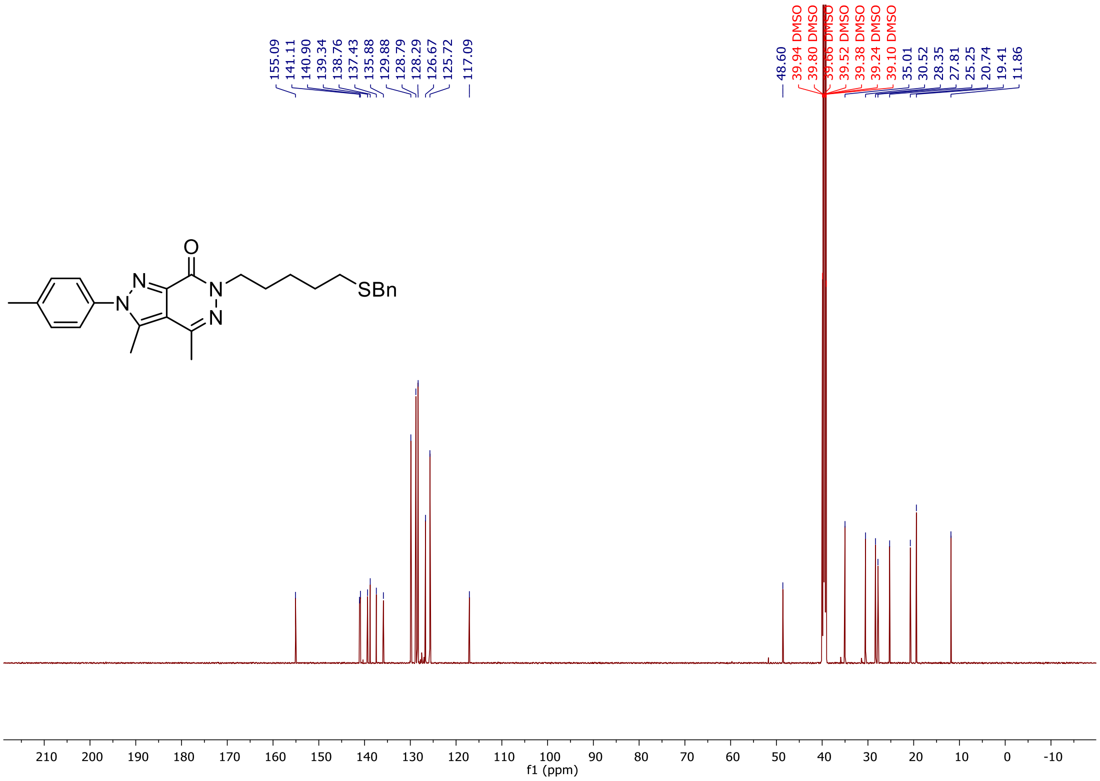

^{1}H NMR Spectrum of **S7d** (600 MHz, DMSO-d_6)

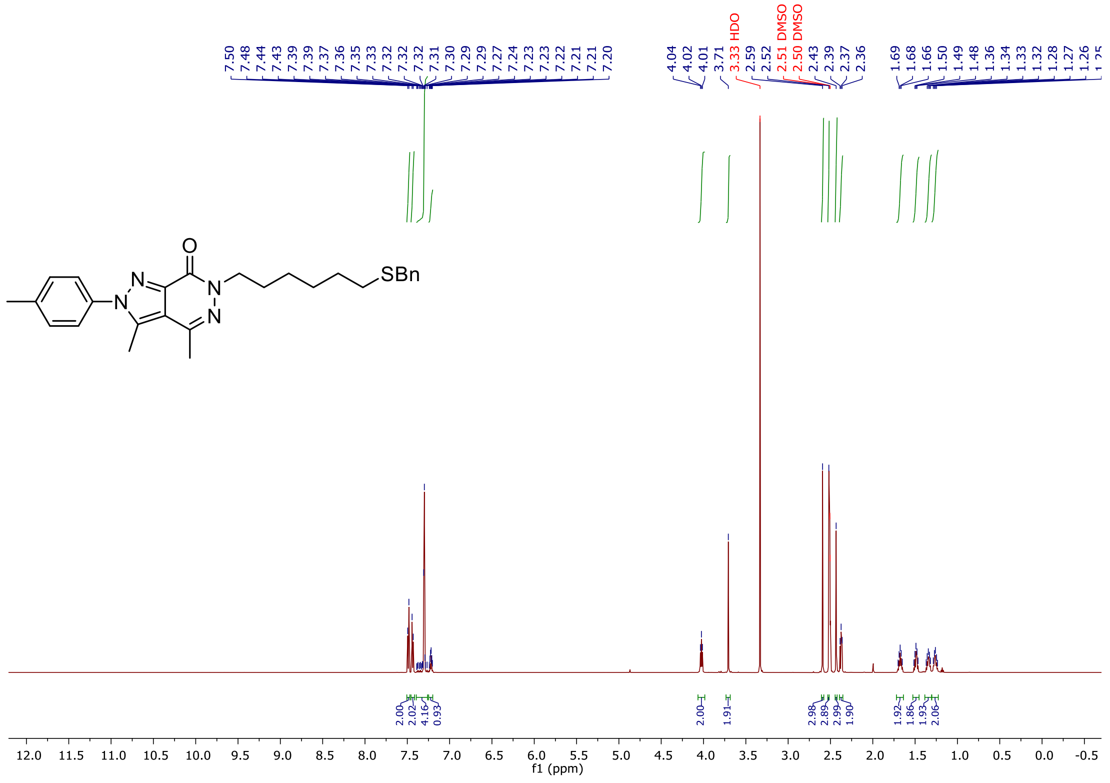

^{13}C NMR Spectrum of **S7d** (151 MHz, DMSO-d_6)

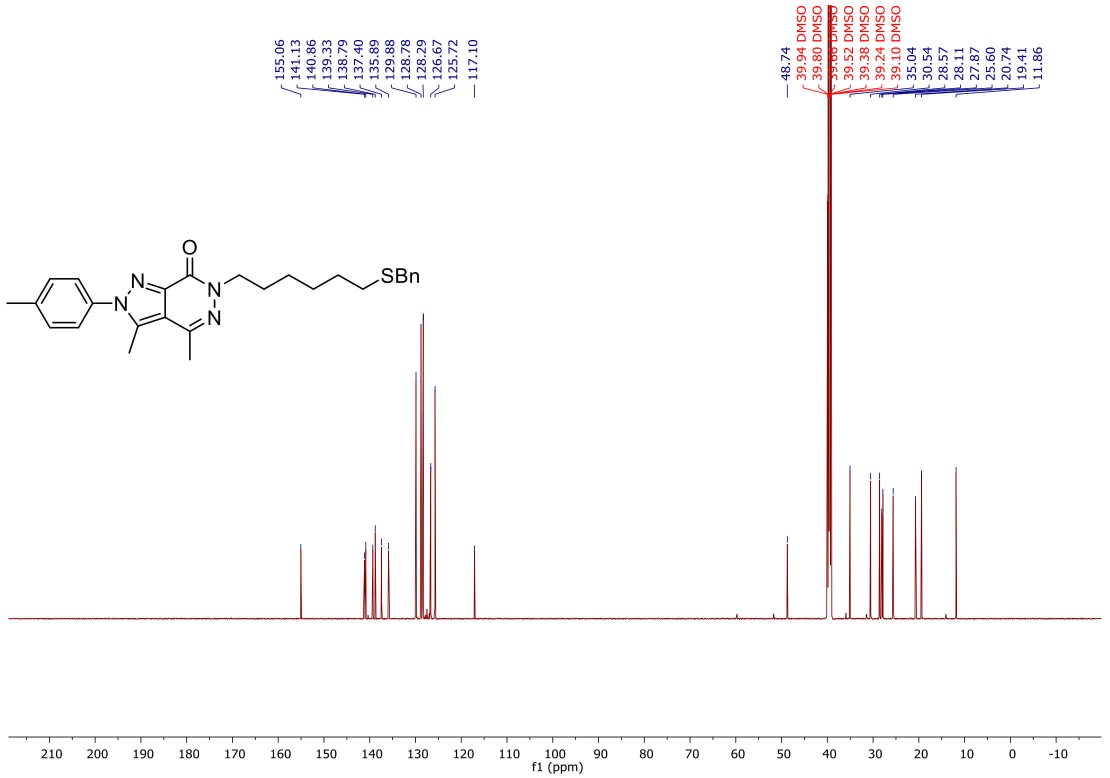

^{1}H NMR Spectrum of **S8a** (500 MHz, $CDCl_3$)

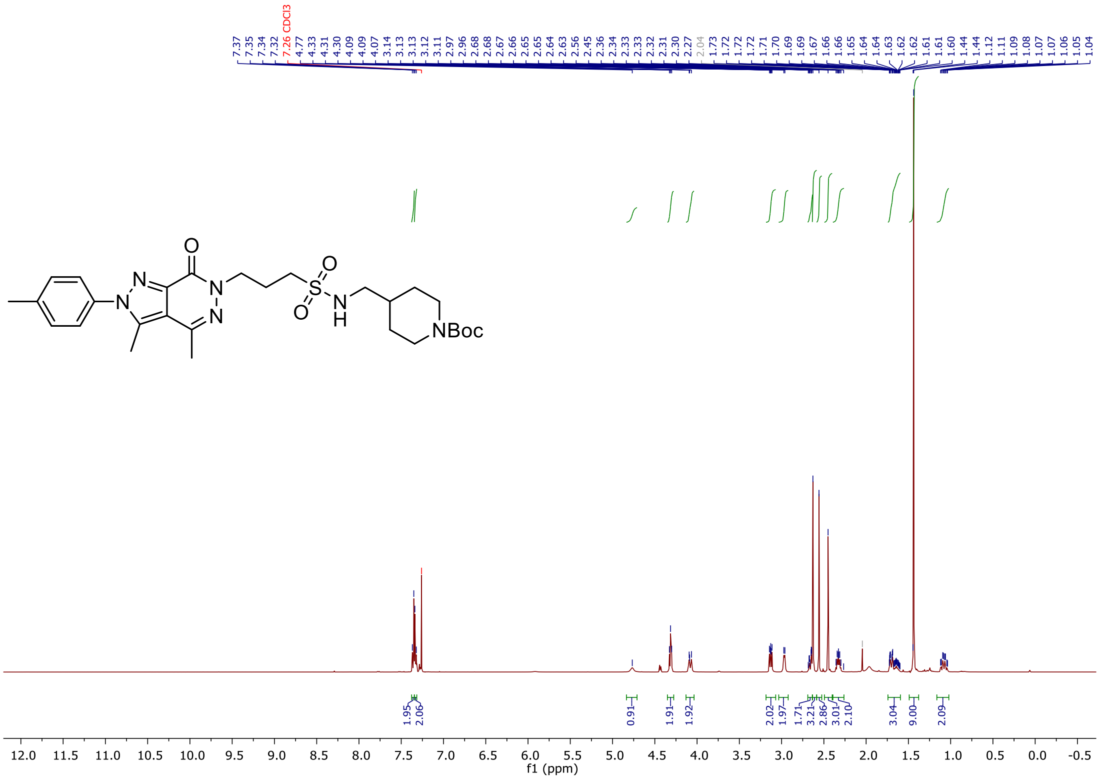

^{13}C NMR Spectrum of **S8a** (176 MHz, $CDCl_3$)

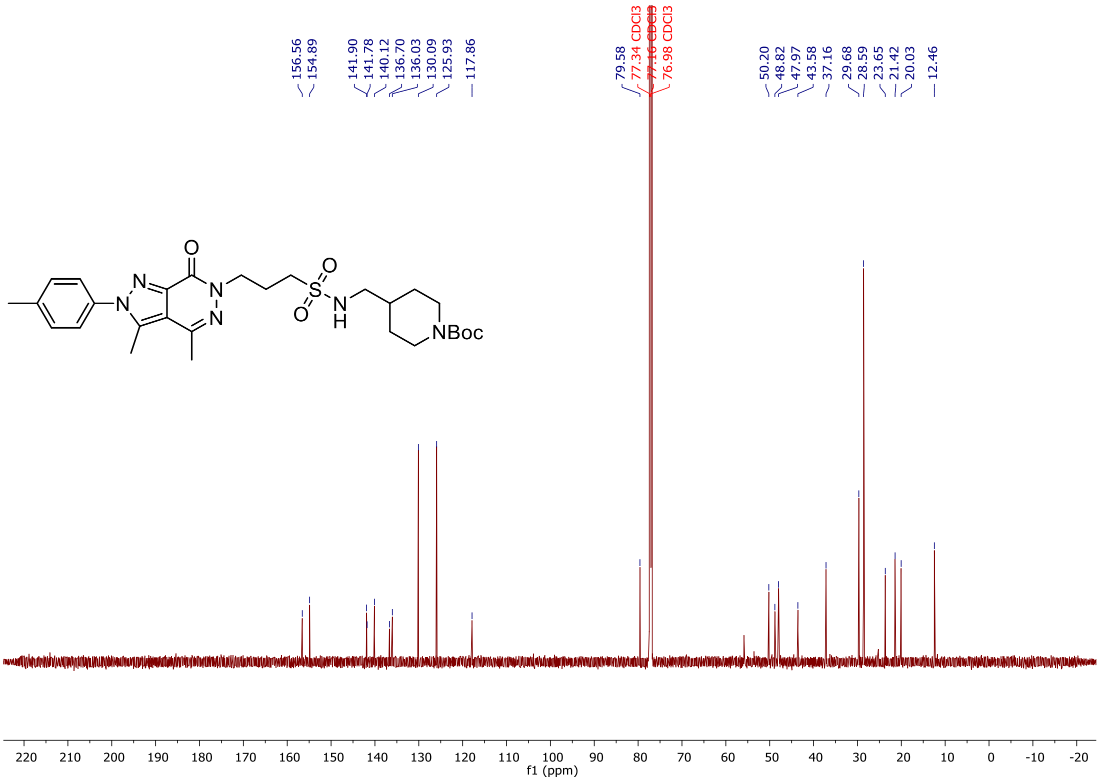

^{1}H NMR Spectrum of **S8b** (500 MHz, $CDCl_3$)

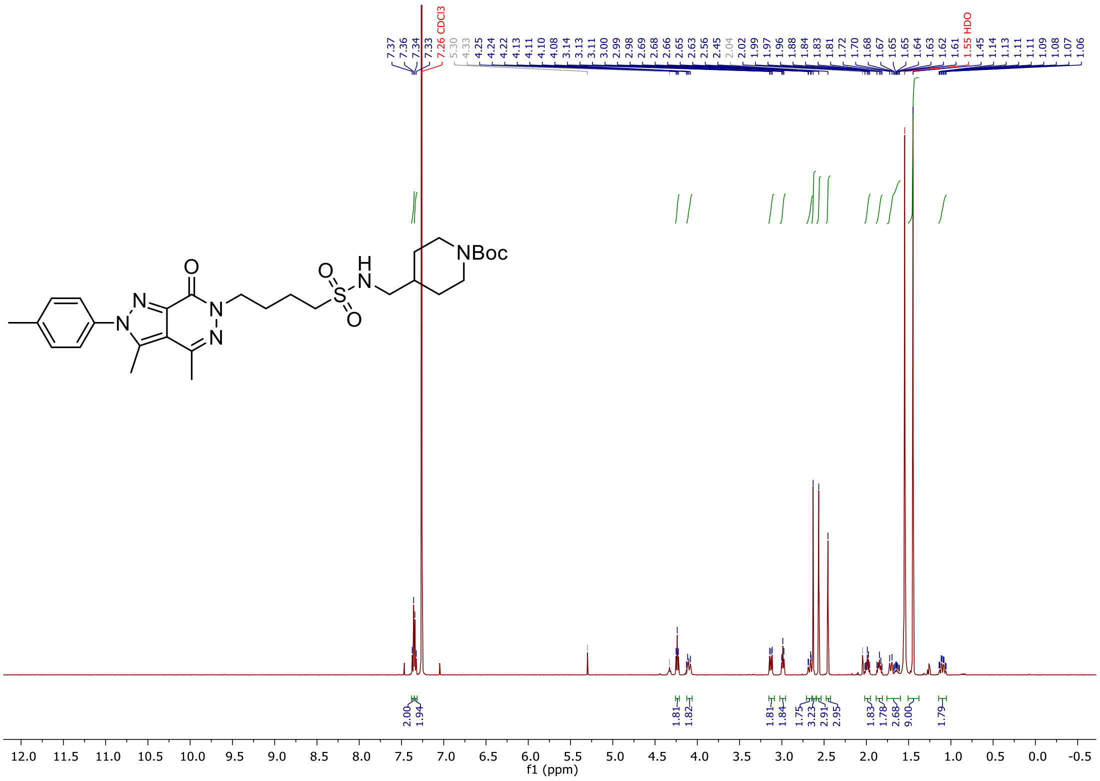

^{13}C NMR Spectrum of **S8b** (176 MHz, $CDCl_3$)

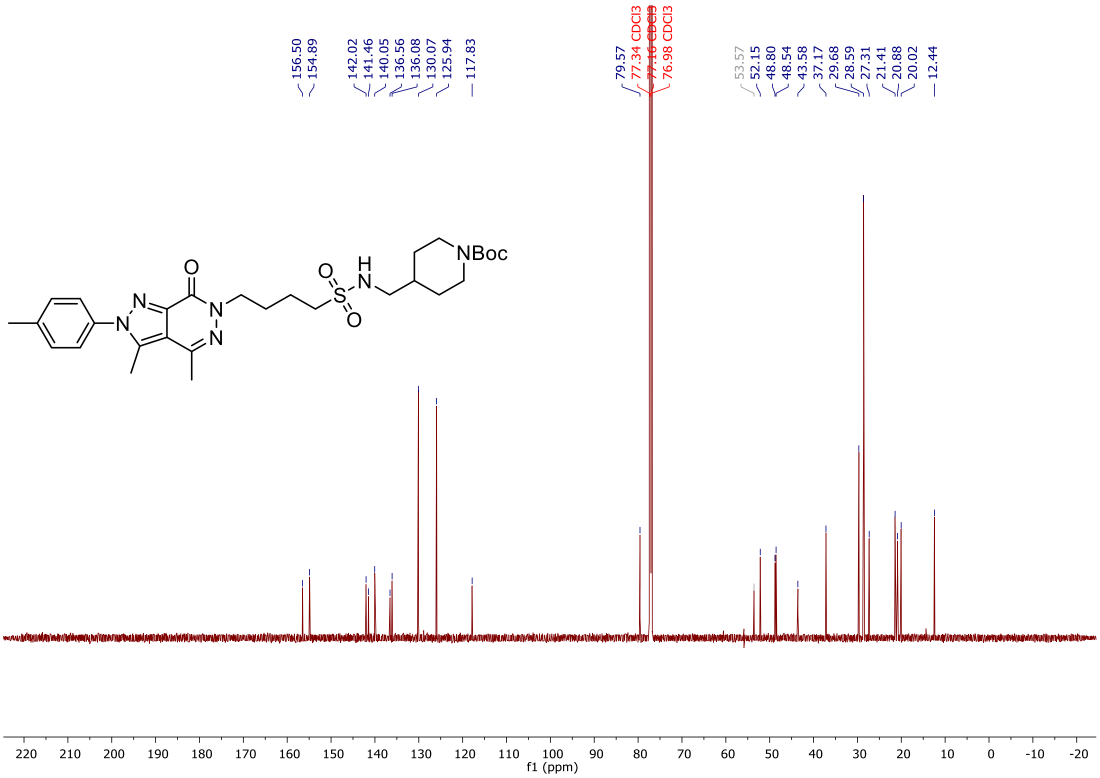

^{1}**H NMR** Spectrum of **S8c** (500 MHz, $CDCl_3$)

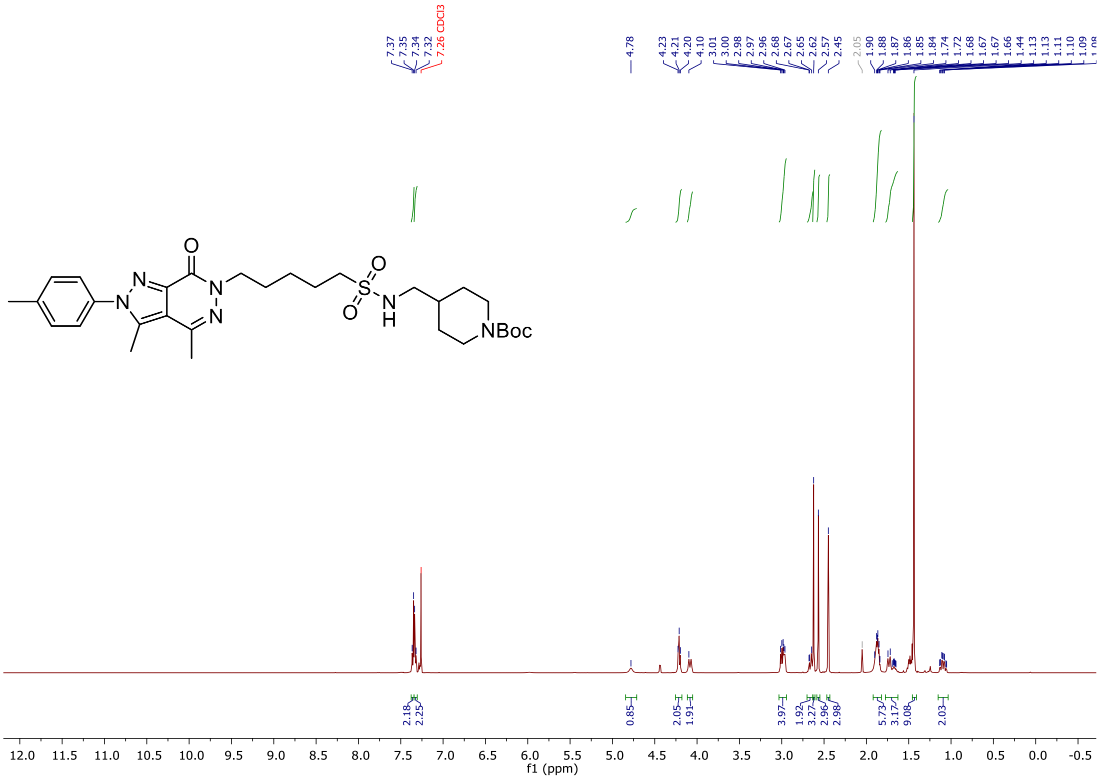

^{13}**C NMR** Spectrum of **S8c** (176 MHz, $CDCl_3$)

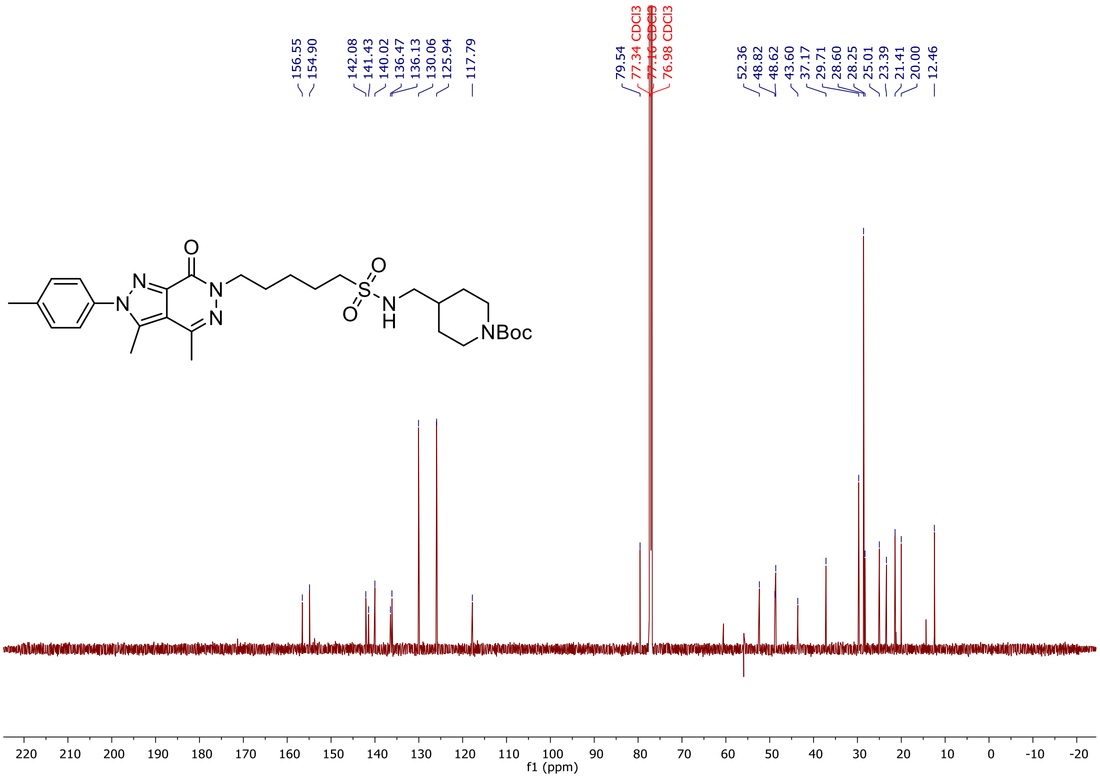

^{1}H NMR Spectrum of **S8d** (700 MHz, $CDCl_3$)

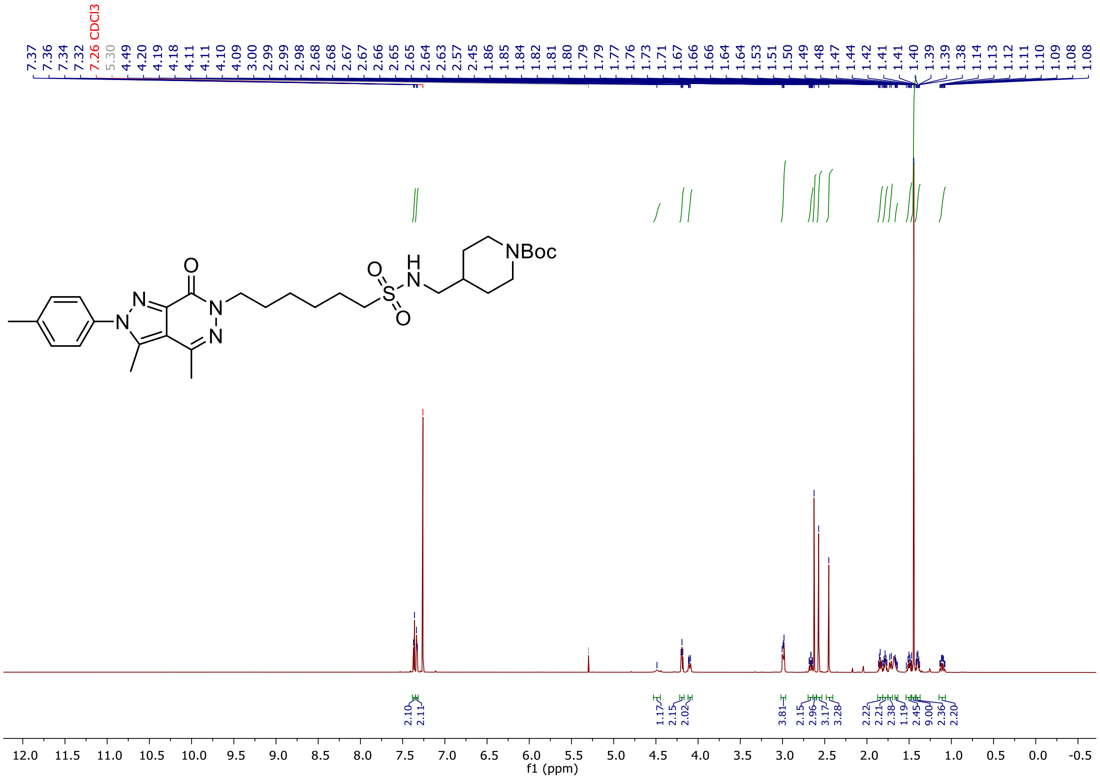

^{13}C NMR Spectrum of **S8d** (176 MHz, $CDCl_3$)

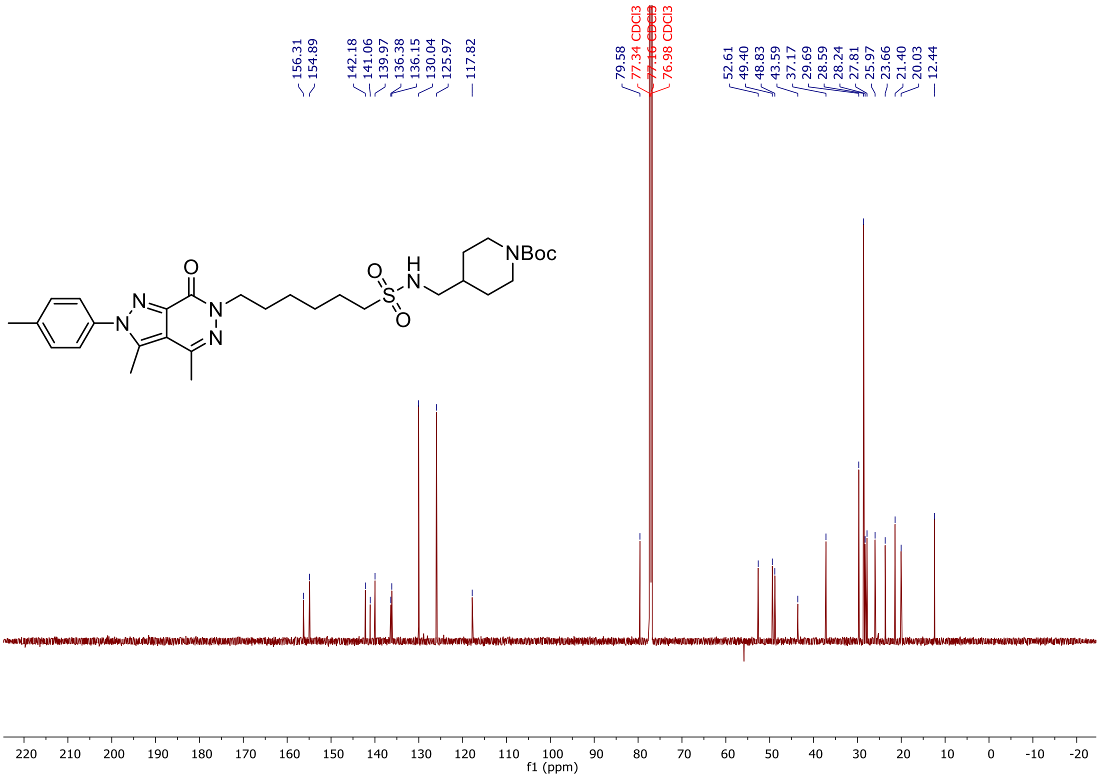

^{1}H NMR Spectrum of **Deltazinone 1** (700 MHz, $CDCl_3$)

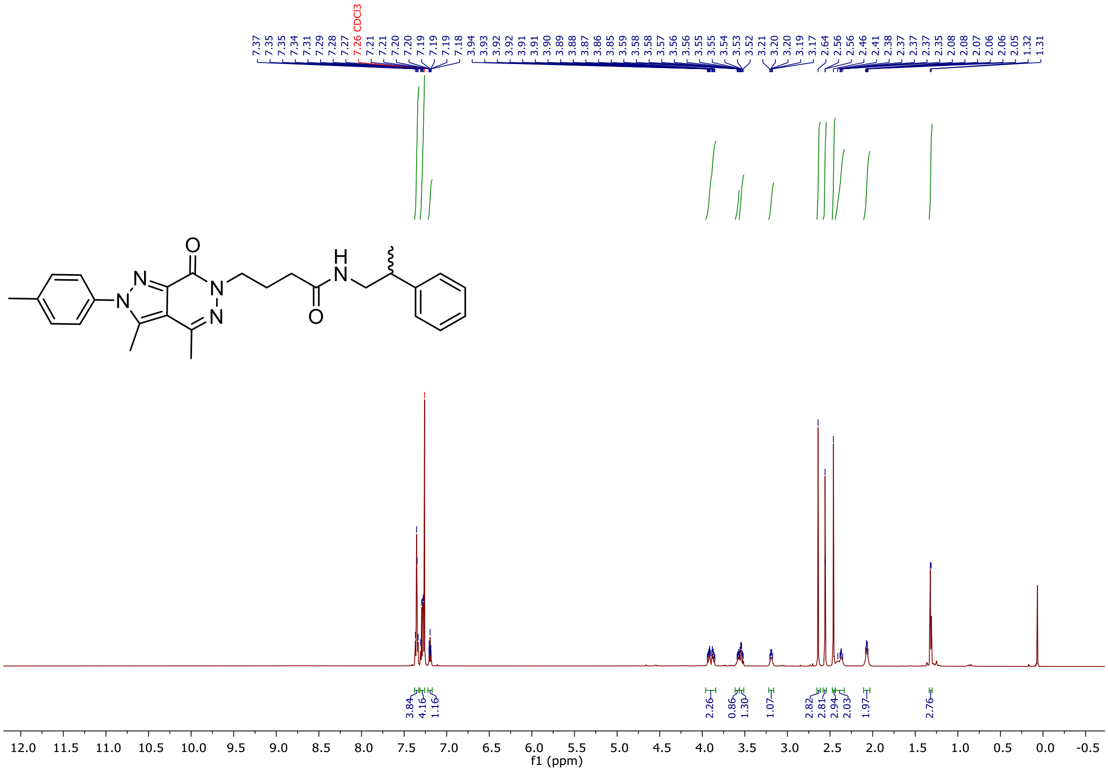

^{13}C NMR Spectrum of **Deltazinone 1** (176 MHz, $CDCl_3$)

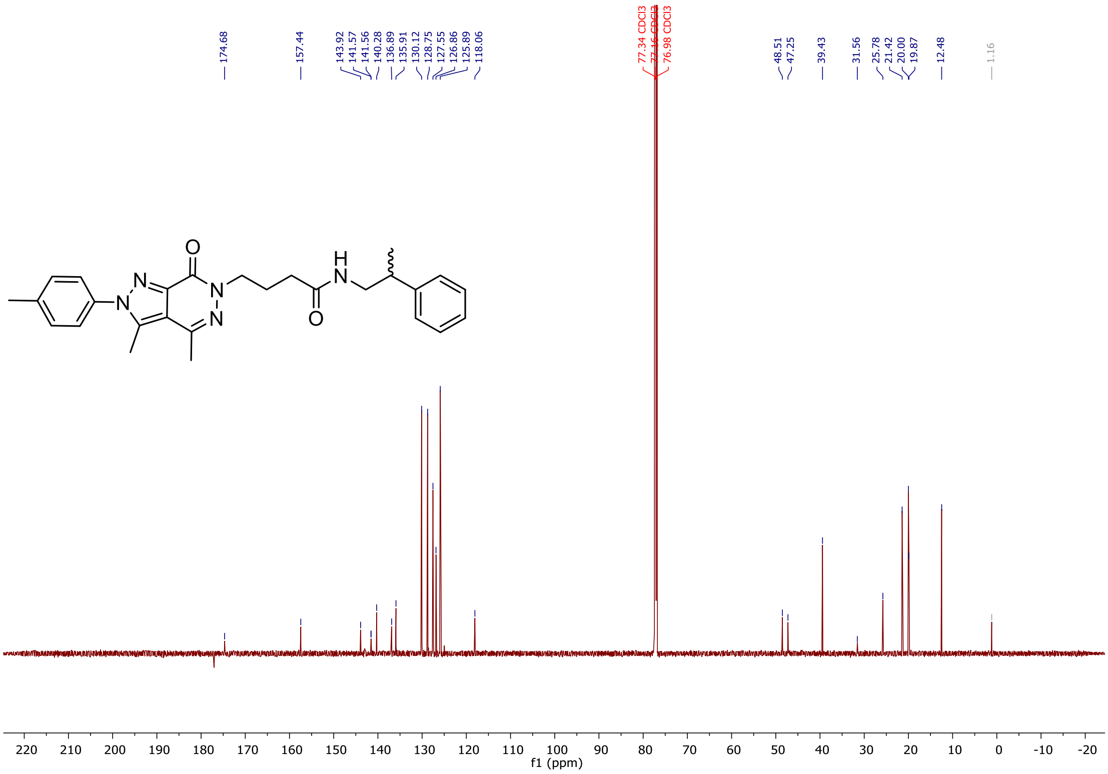

^{1}H NMR Spectrum of **2a** (700 MHz, DMSO-d_6)

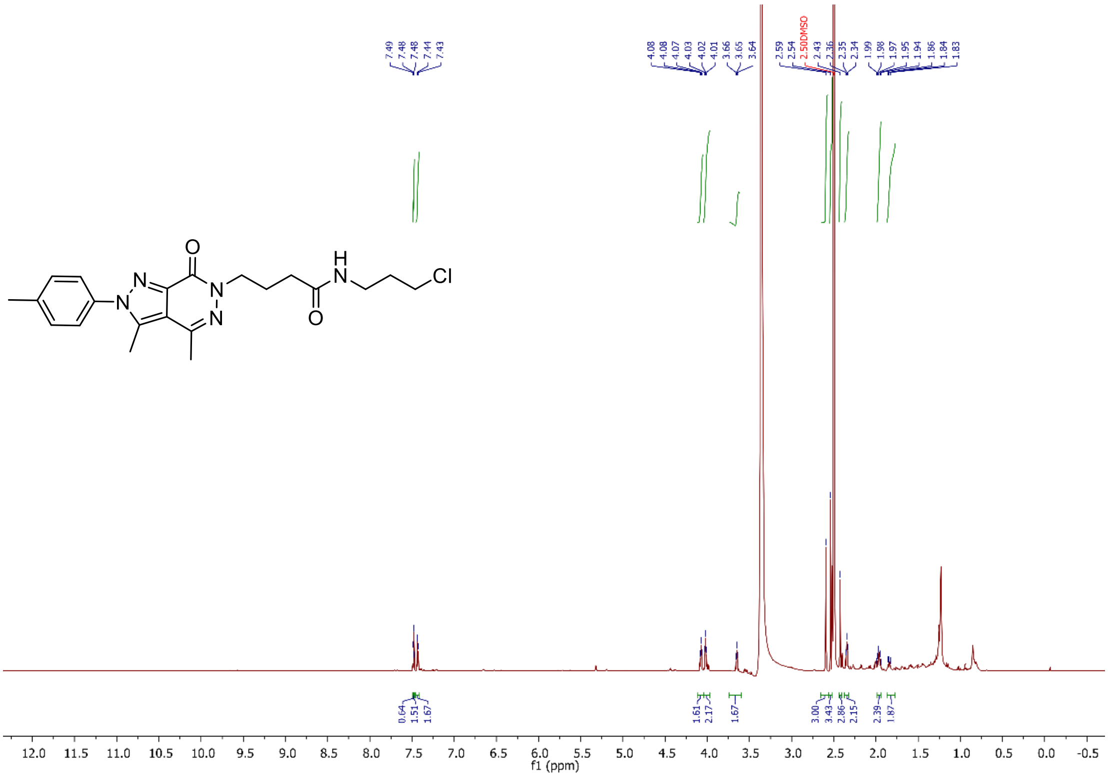

^{13}C NMR Spectrum of **2a** (151 MHz, DMSO-d_6)

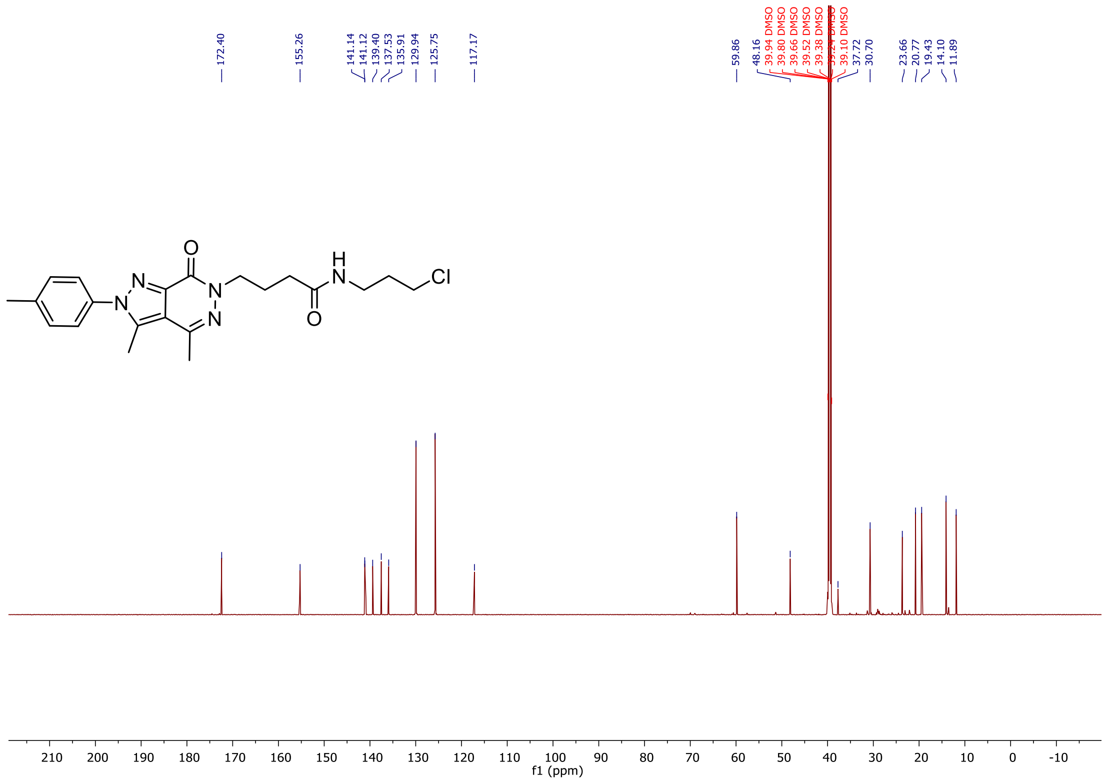

^{1}H NMR Spectrum of 2b (700 MHz, DMSO-d_6)

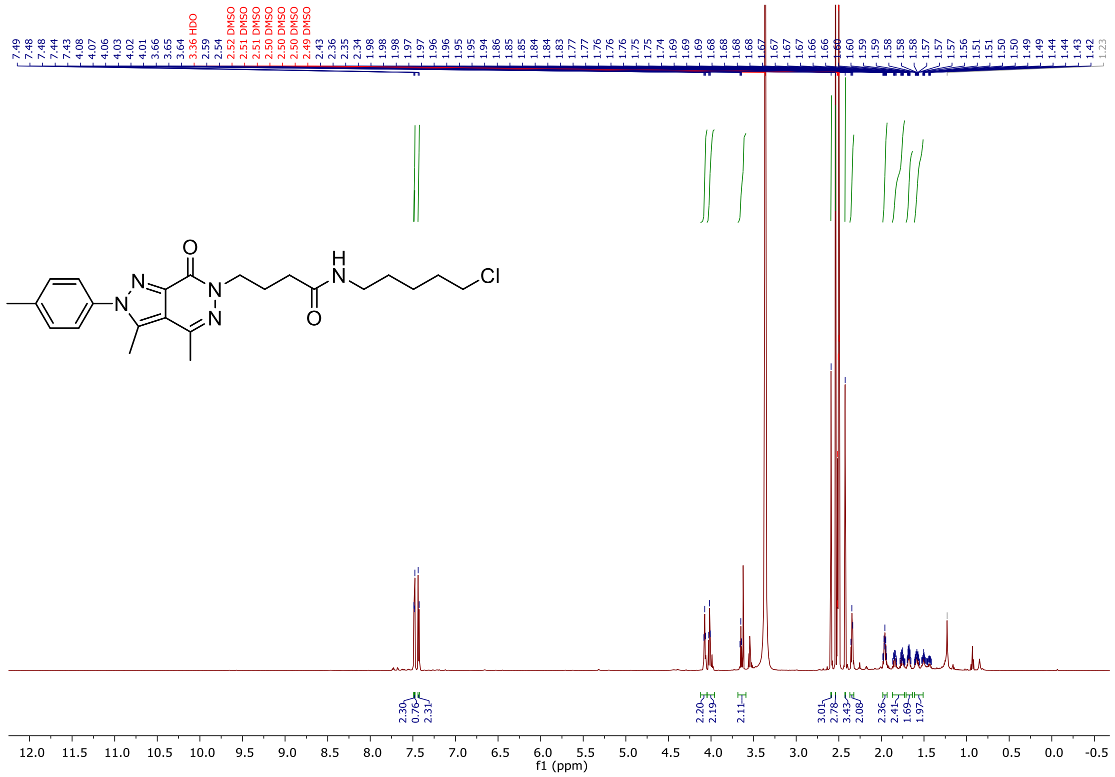

^{13}C NMR Spectrum of 2b (176 MHz, DMSO-d_6)

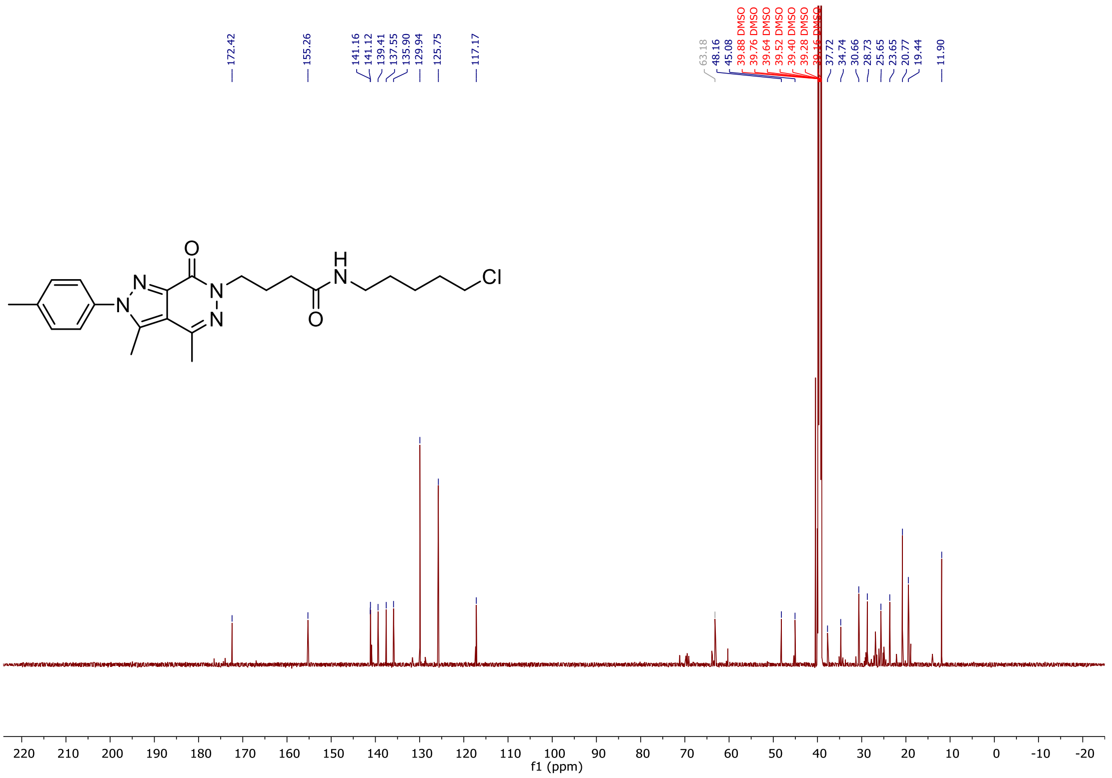

^{1}H NMR Spectrum of **2c** (700 MHz, DMSO-d_6)

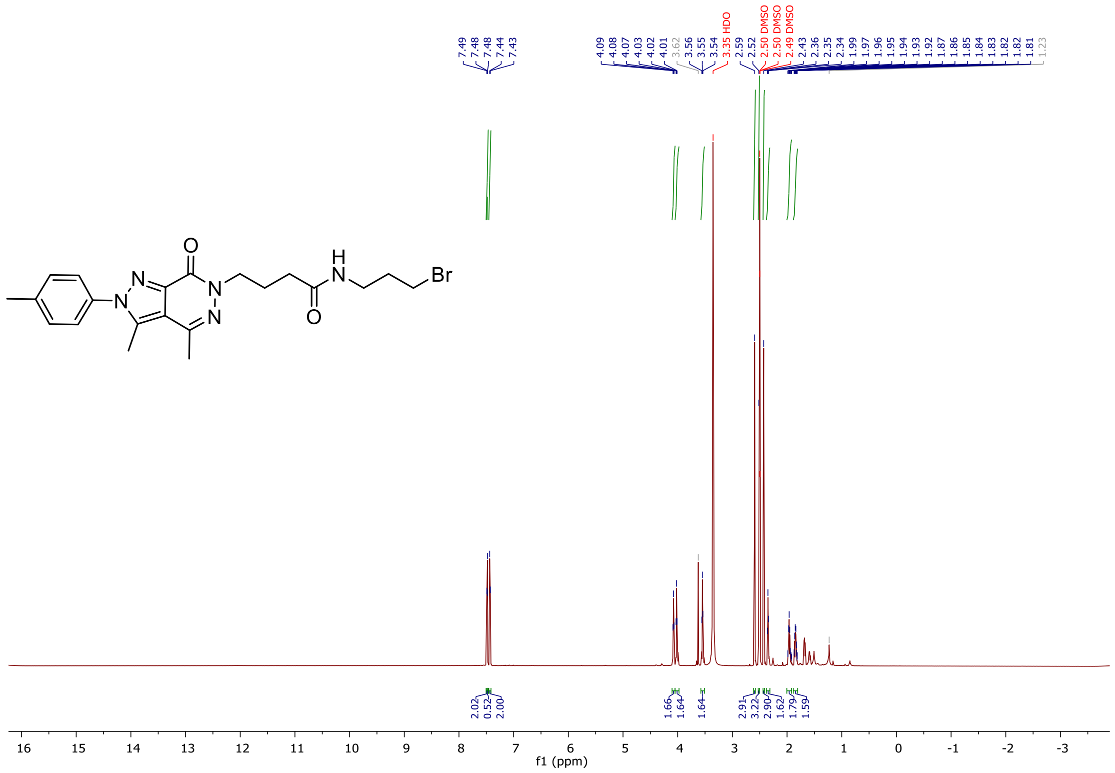

^{13}C NMR Spectrum of **2c** (176 MHz, DMSO-d_6)

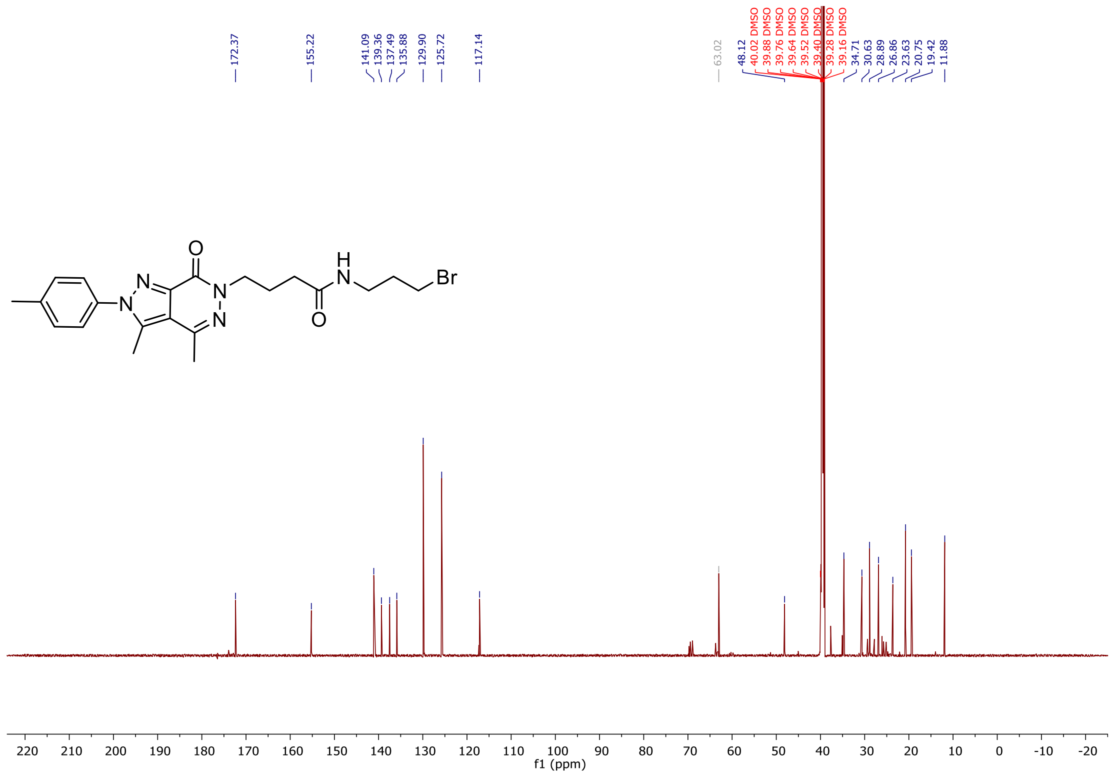

^{1}H NMR Spectrum of **2d** (700 MHz, DMSO-d_6)

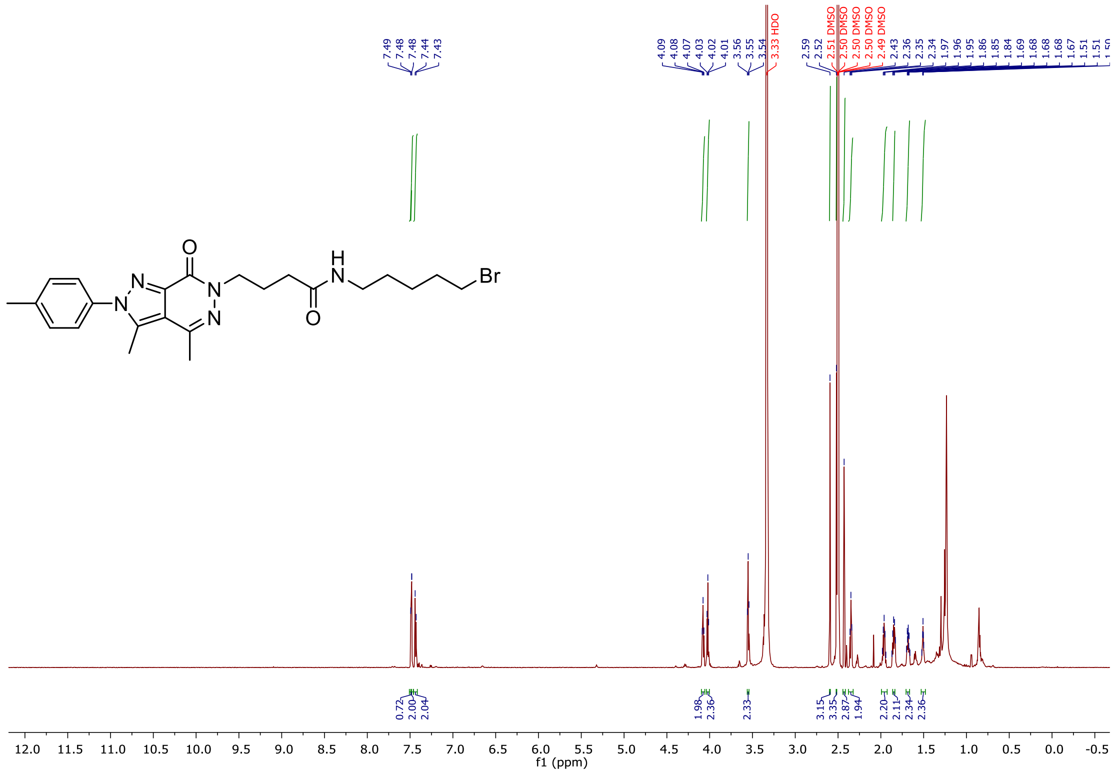

^{13}C NMR Spectrum of **2d** (176 MHz, DMSO-d_6)

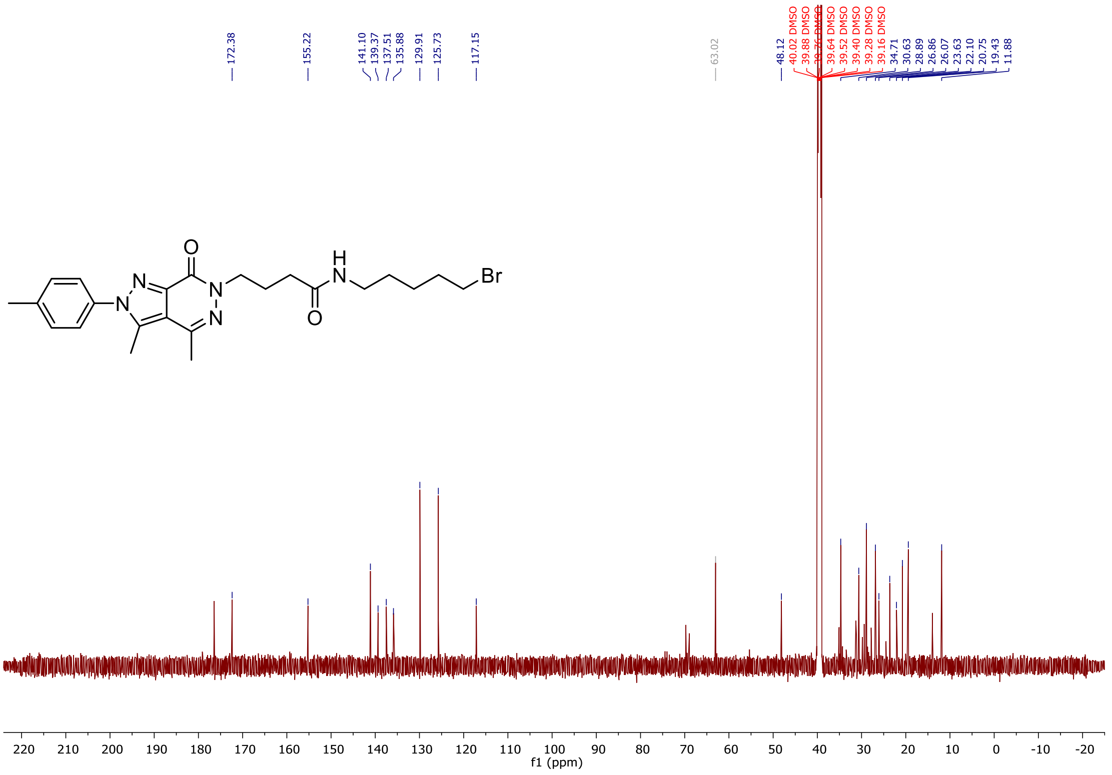

^{1}H NMR Spectrum of **3a** (600 MHz, DMSO-d_6)

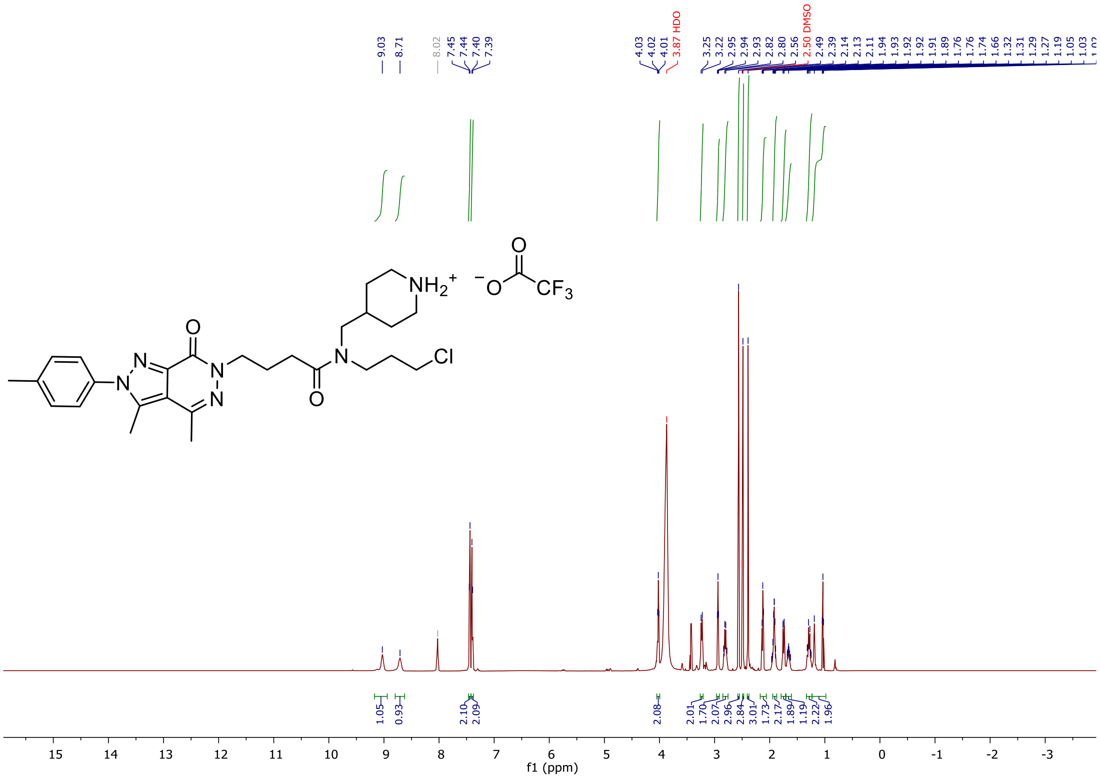

^{13}C NMR Spectrum of **3a** (151 MHz, DMSO-d_6)

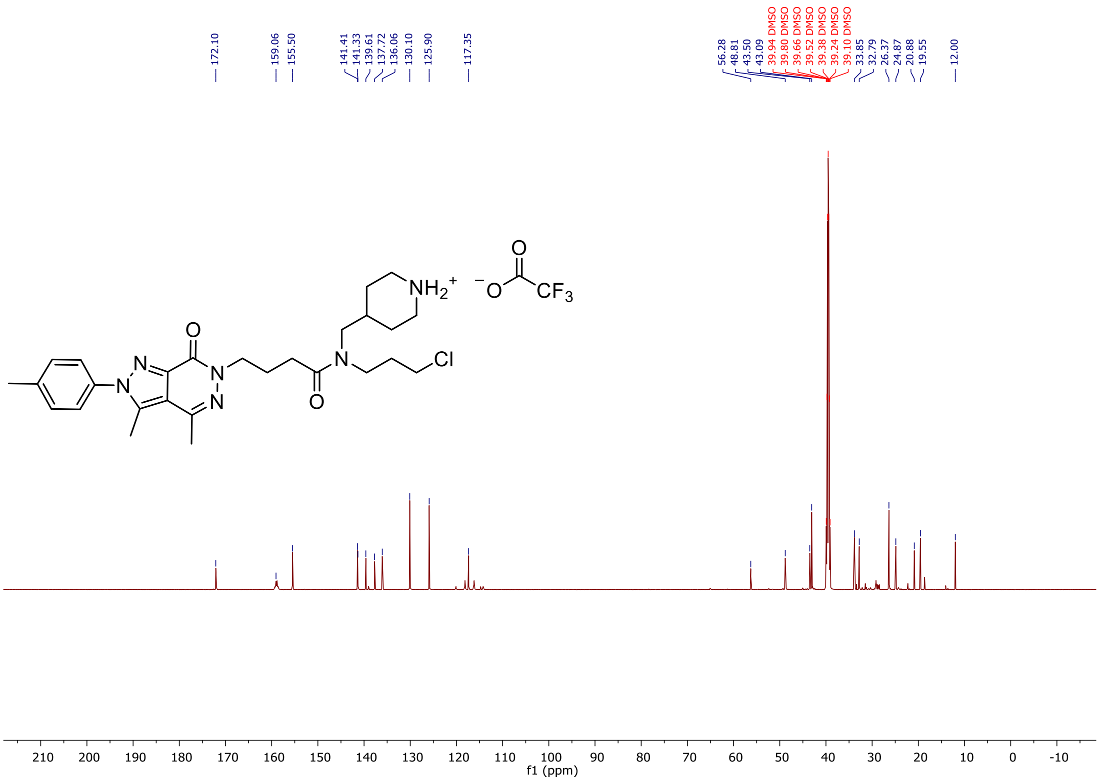

^{1}H NMR Spectrum of **3b** (600 MHz, DMSO-d_6)

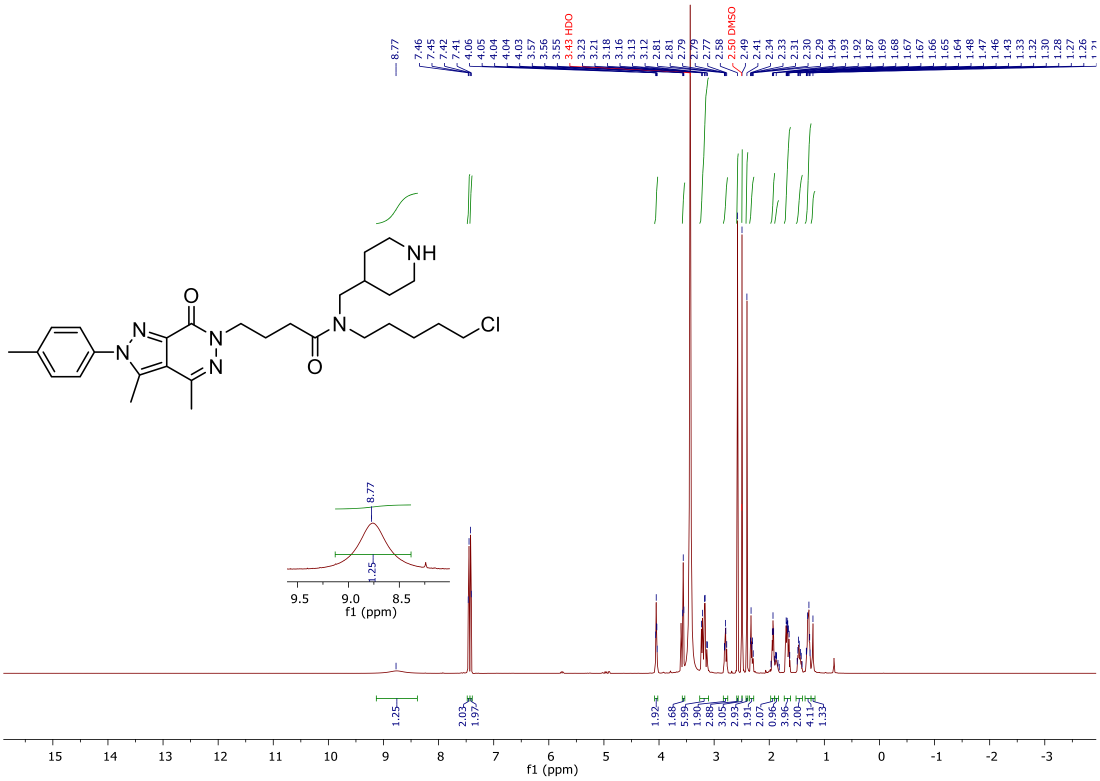

^{13}C NMR Spectrum of **3b** (151 MHz, DMSO-d_6)

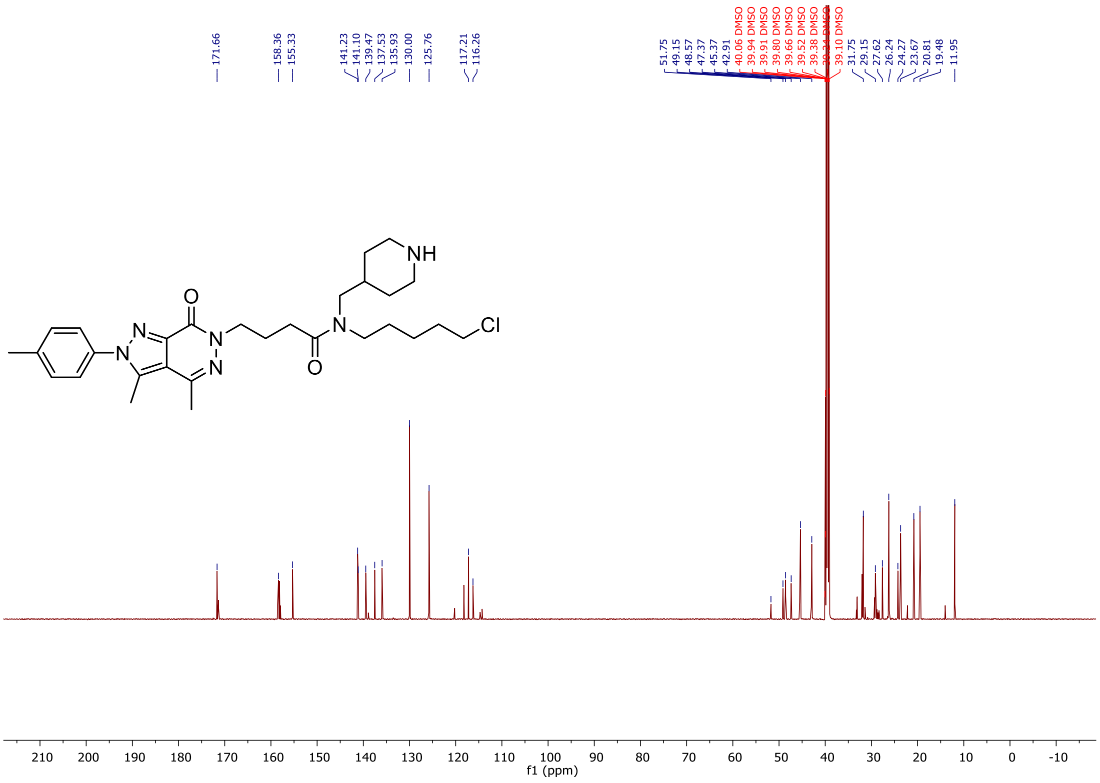

^{1}H NMR Spectrum of 3c (600 MHz, DMSO-d_6)

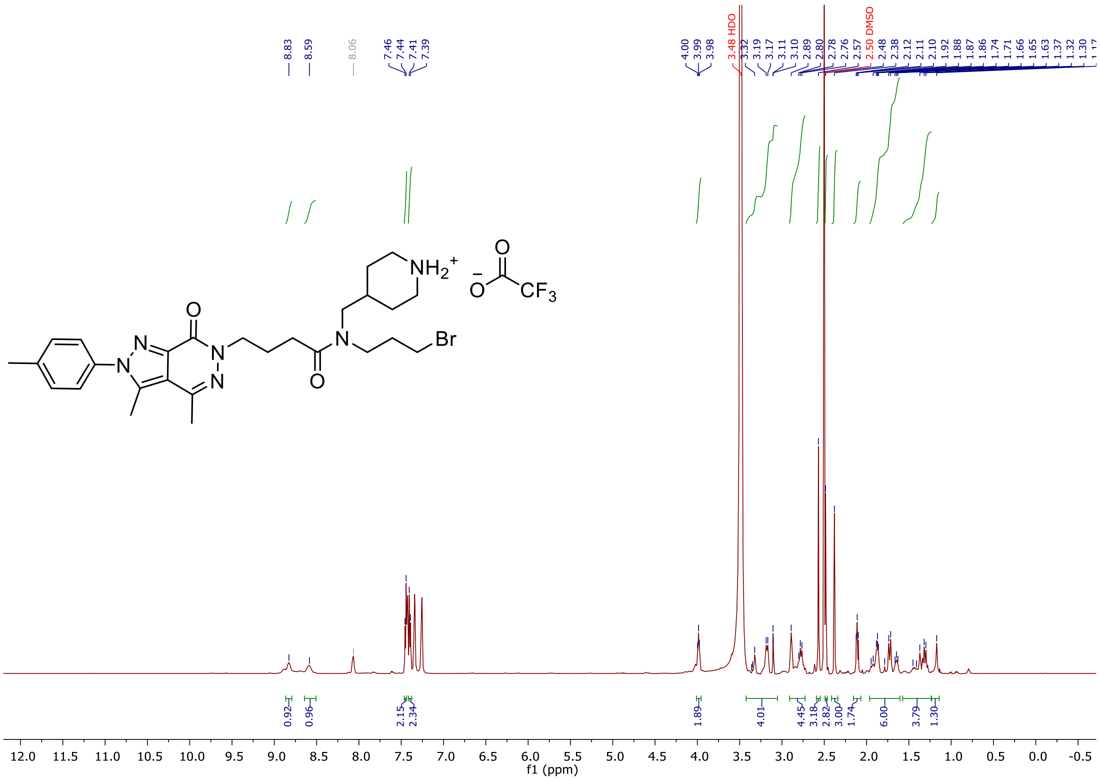

^{13}C NMR Spectrum of 3c (151 MHz, DMSO-d_6)

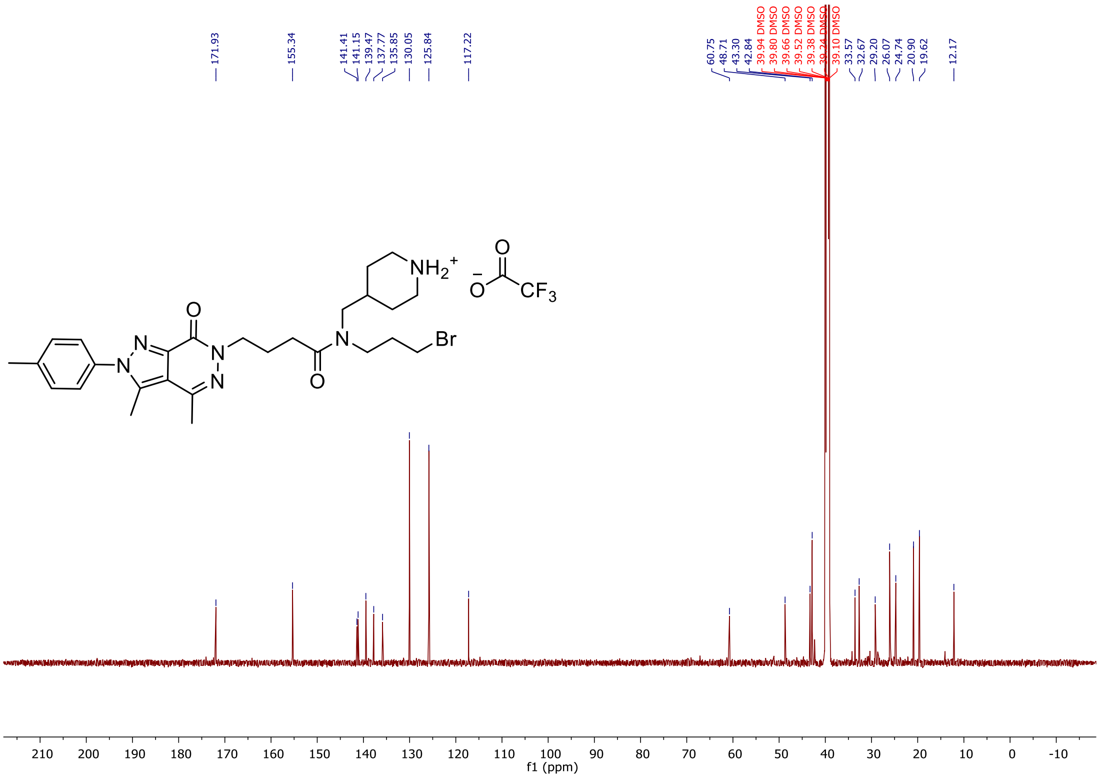

¹H NMR Spectrum of **3d** (600 MHz, DMSO-d_6)

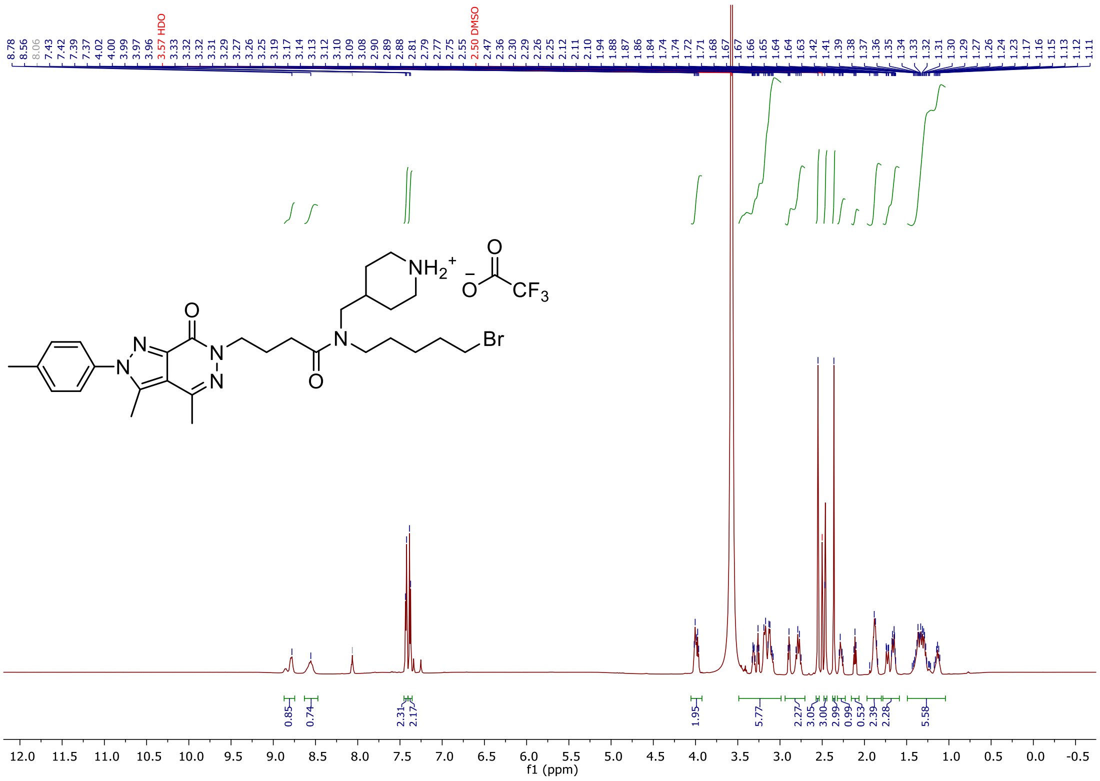

¹³C NMR Spectrum of **3d** (151 MHz, DMSO-d_6)

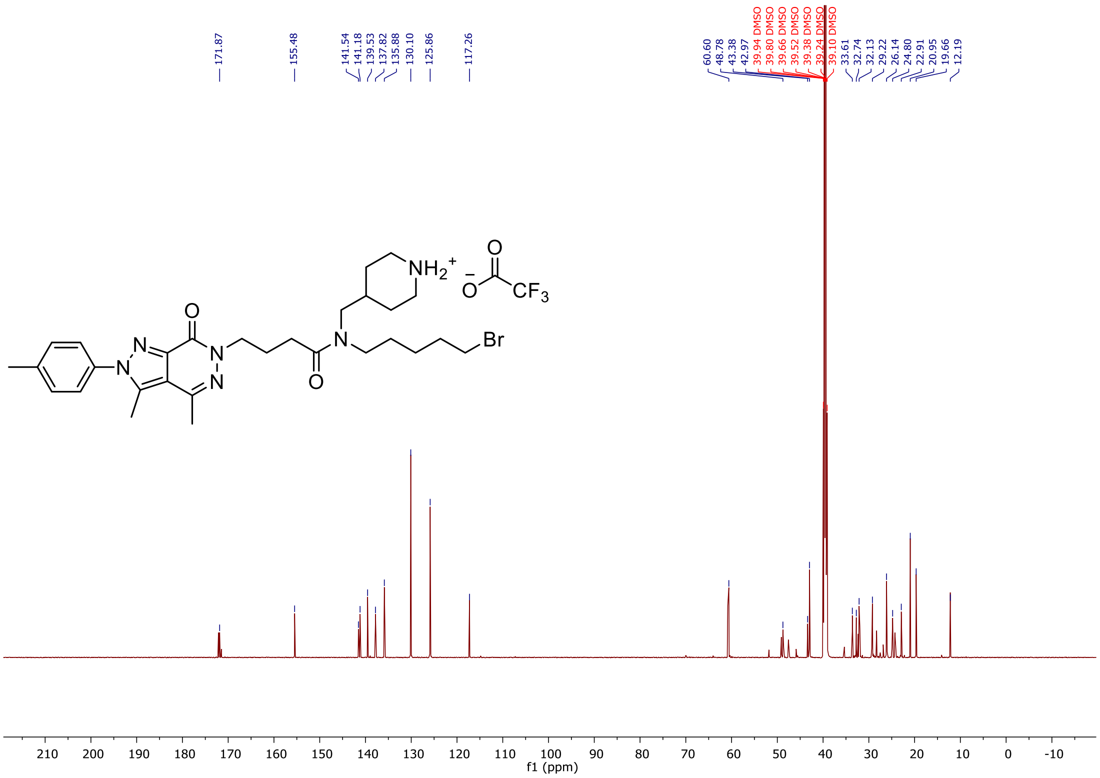

^{1}**H NMR** Spectrum of **4a** (600 MHz, DMSO-d_6)

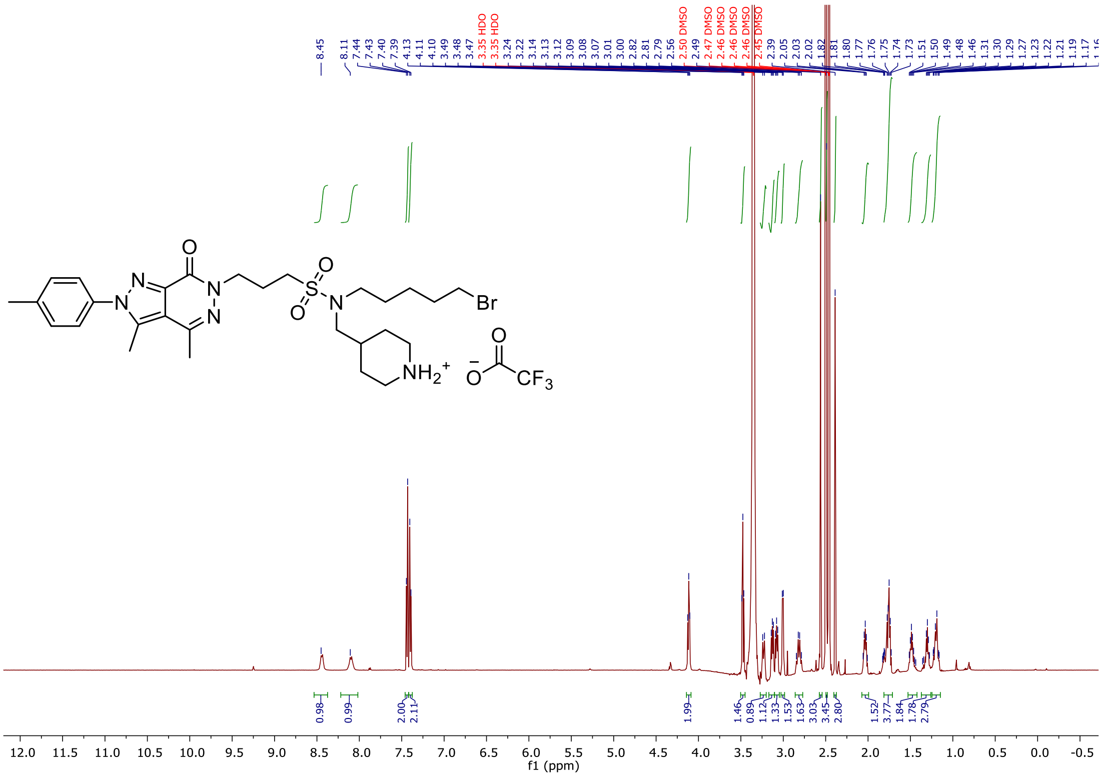

^{13}**C NMR** Spectrum of **4a** (151 MHz, DMSO-d_6)

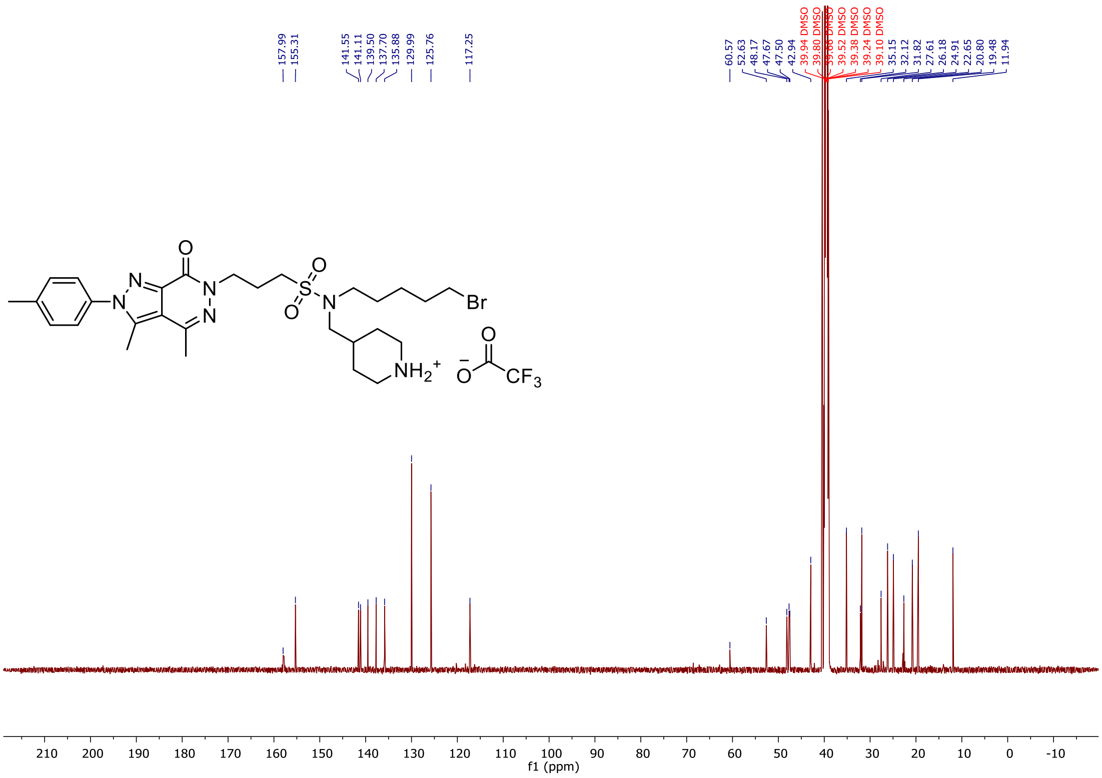

[1]**H NMR** Spectrum of **4b** (700 MHz, DMSO-d_6)

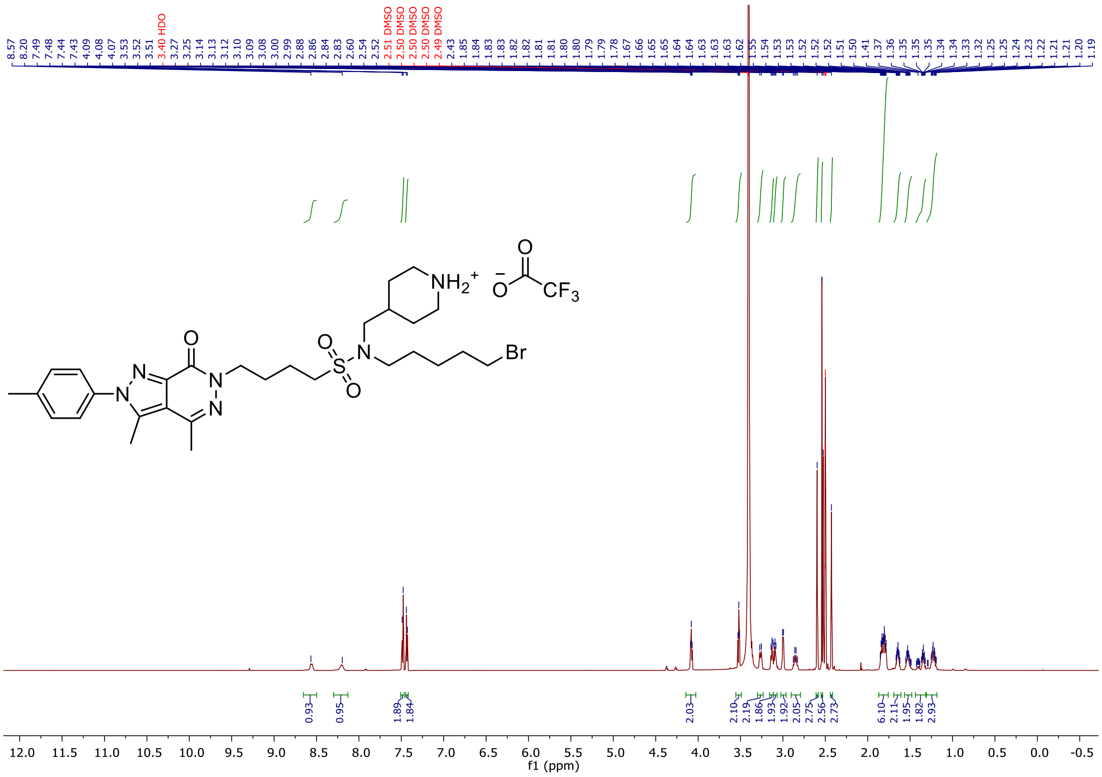

[13]**C NMR** Spectrum of **4b** (176 MHz, DMSO-d_6)

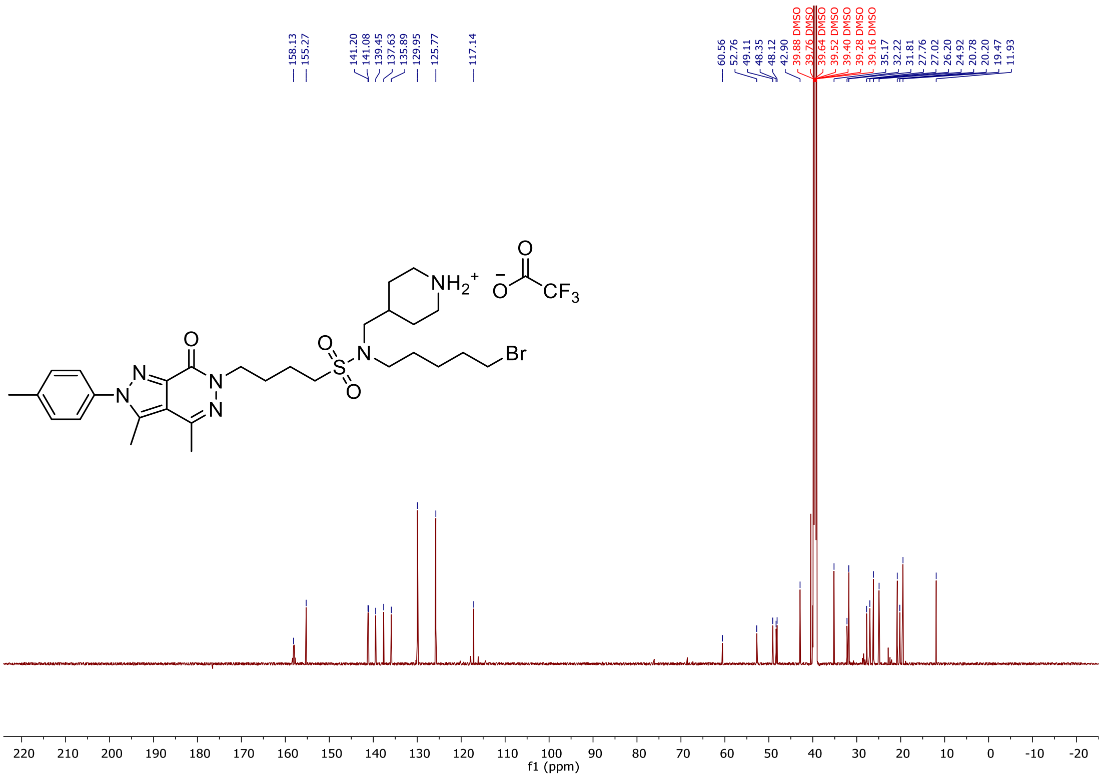

^{1}H NMR Spectrum of **4c** (700 MHz, DMSO-d_6)

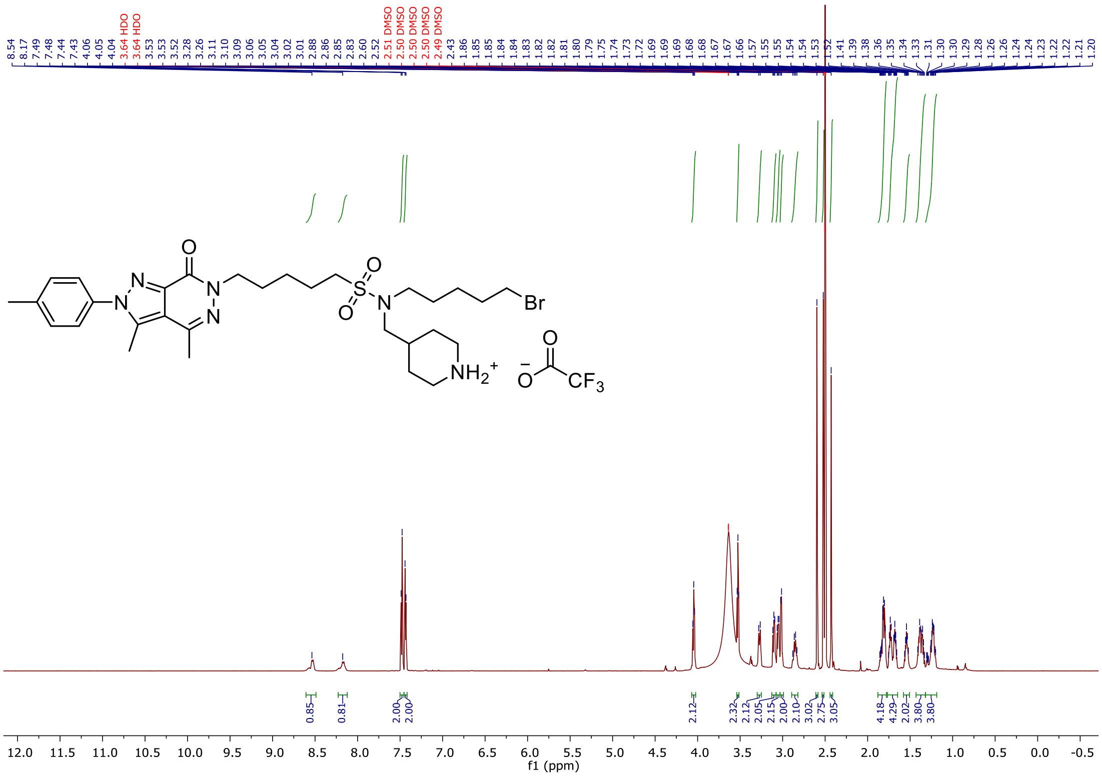

^{13}C NMR Spectrum of **4c** (176 MHz, DMSO-d_6)

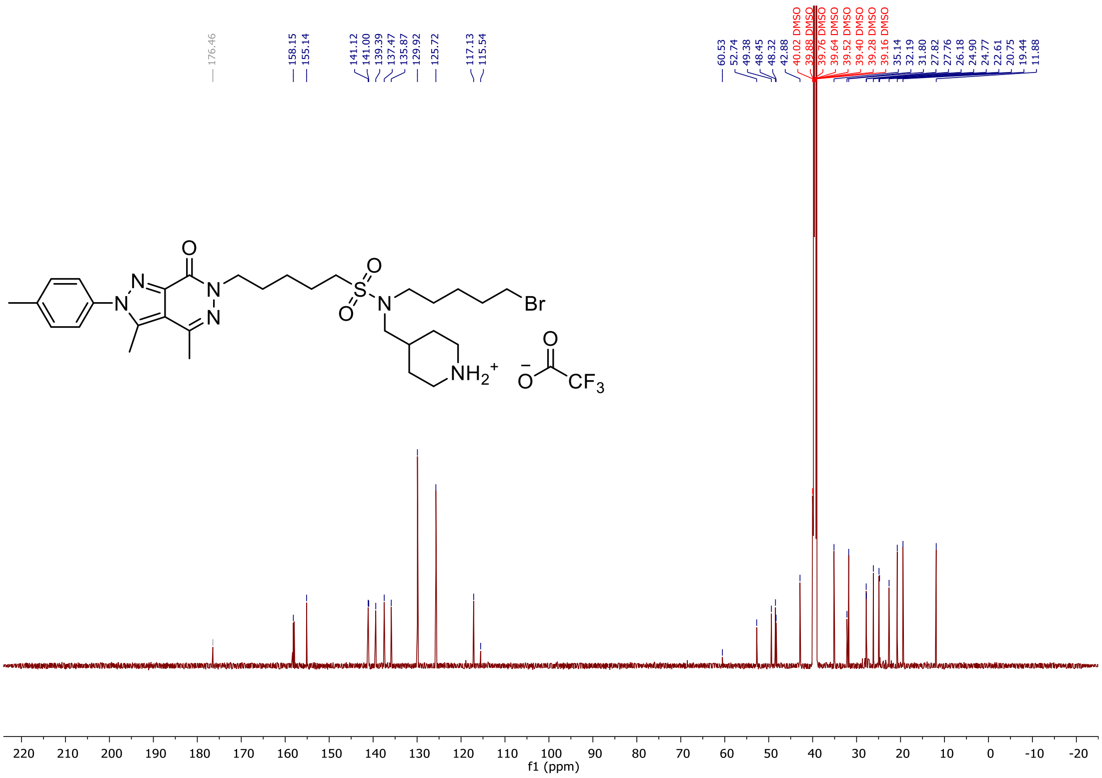

^{1}H NMR Spectrum of **4d** (700 MHz, DMSO-d_6)

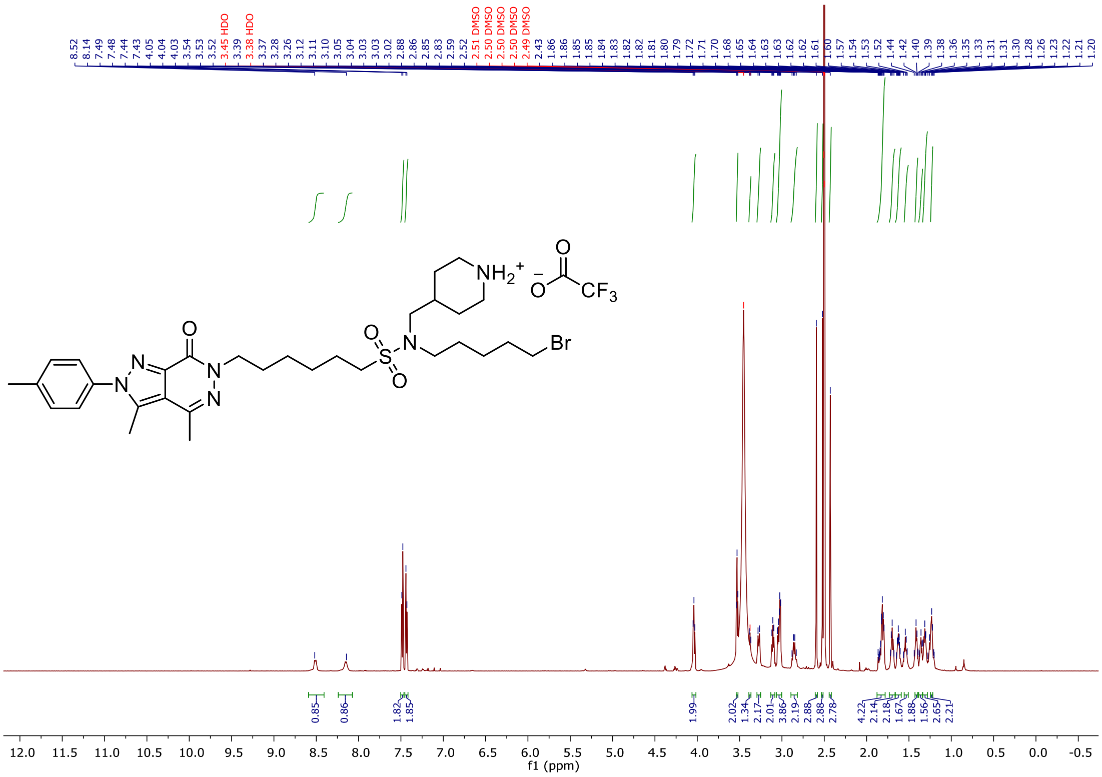

^{13}C NMR Spectrum of **4d** (176 MHz, DMSO-d_6)

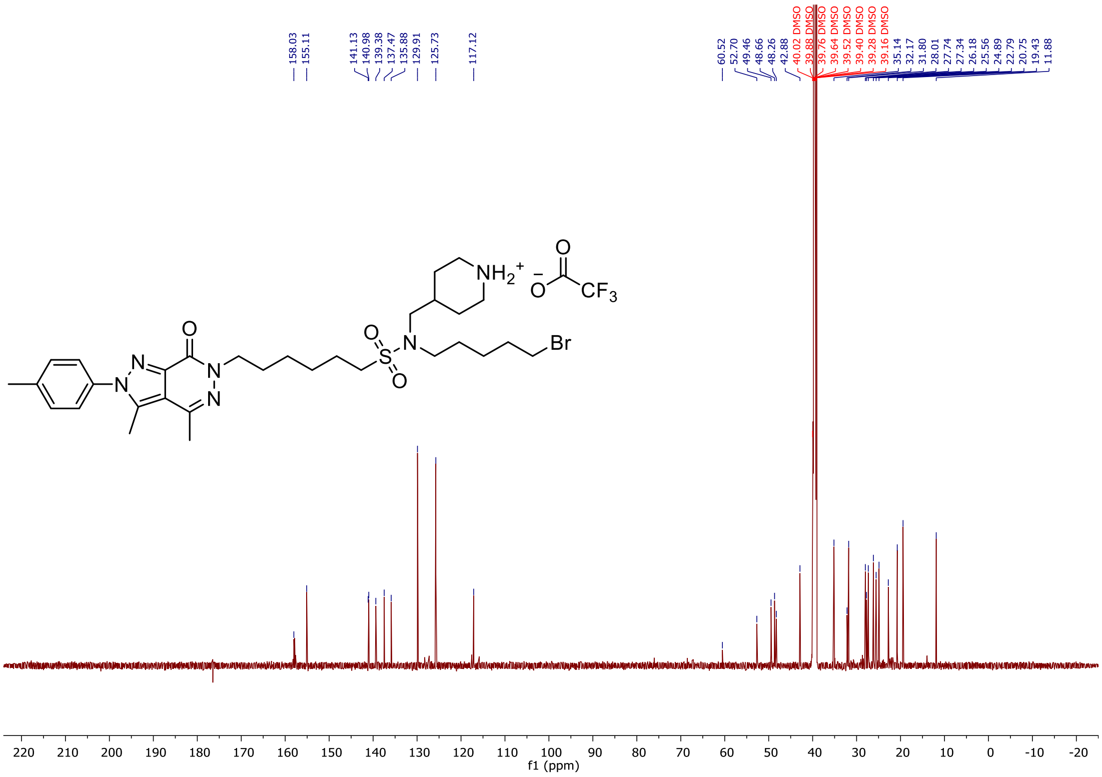

^{1}H NMR Spectrum of **5a** (700 MHz, DMSO-d_6)

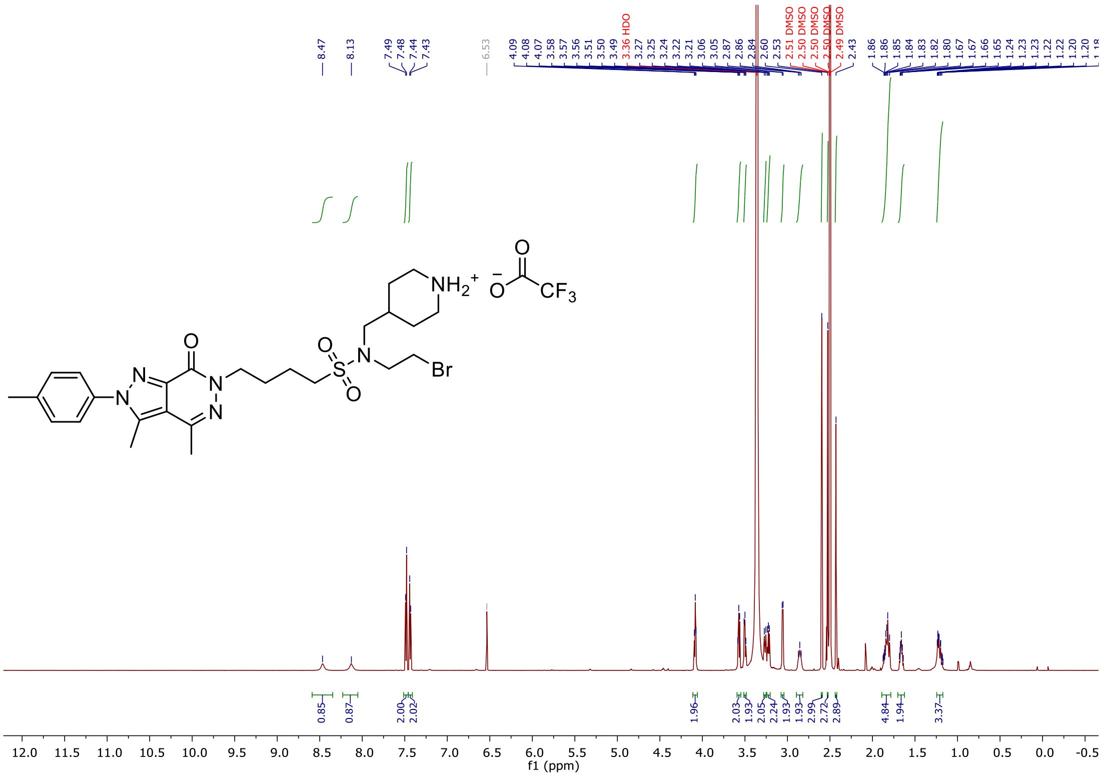

^{13}C NMR Spectrum of **5a** (176 MHz, DMSO-d_6)

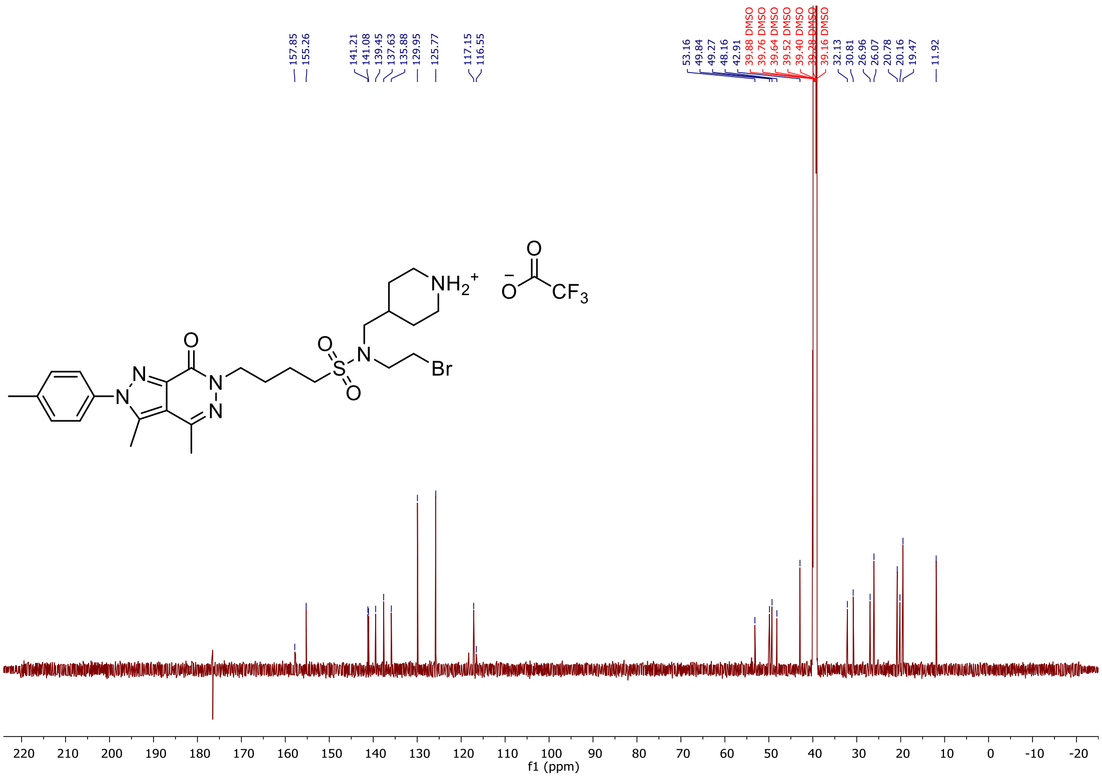

^{1}**H NMR** Spectrum of **5b** (700 MHz, DMSO-d_6)

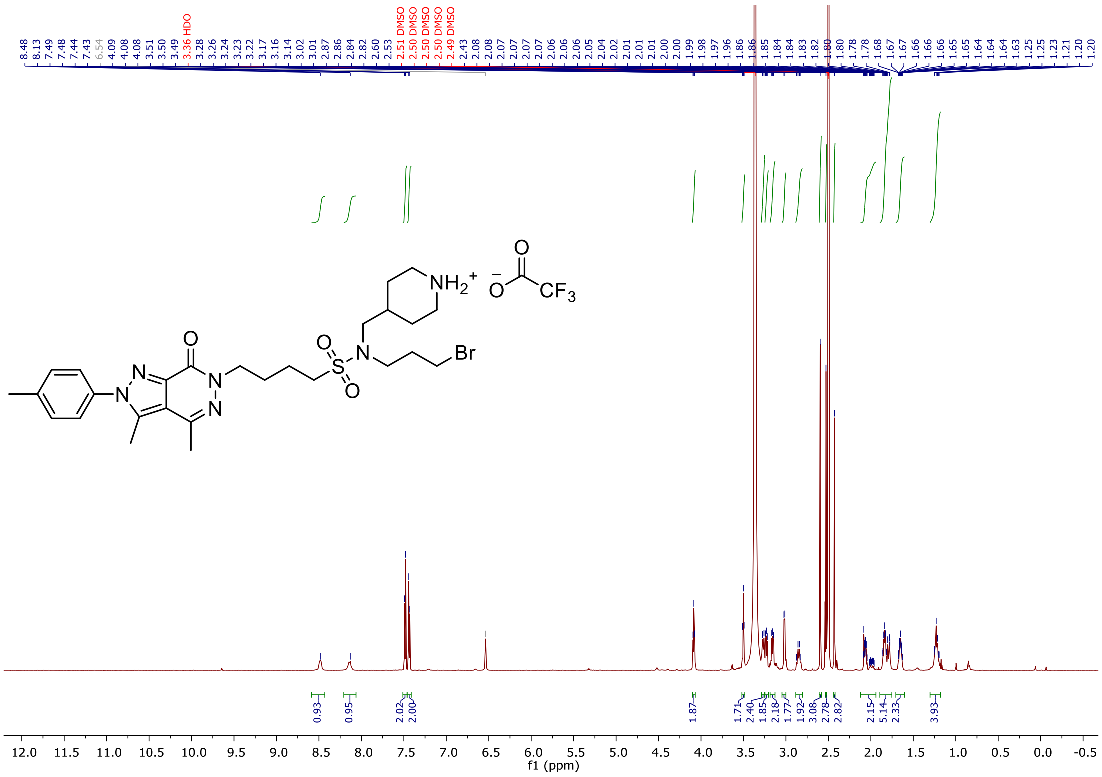

^{13}**C NMR** Spectrum of **5b** (176 MHz, DMSO-d_6)

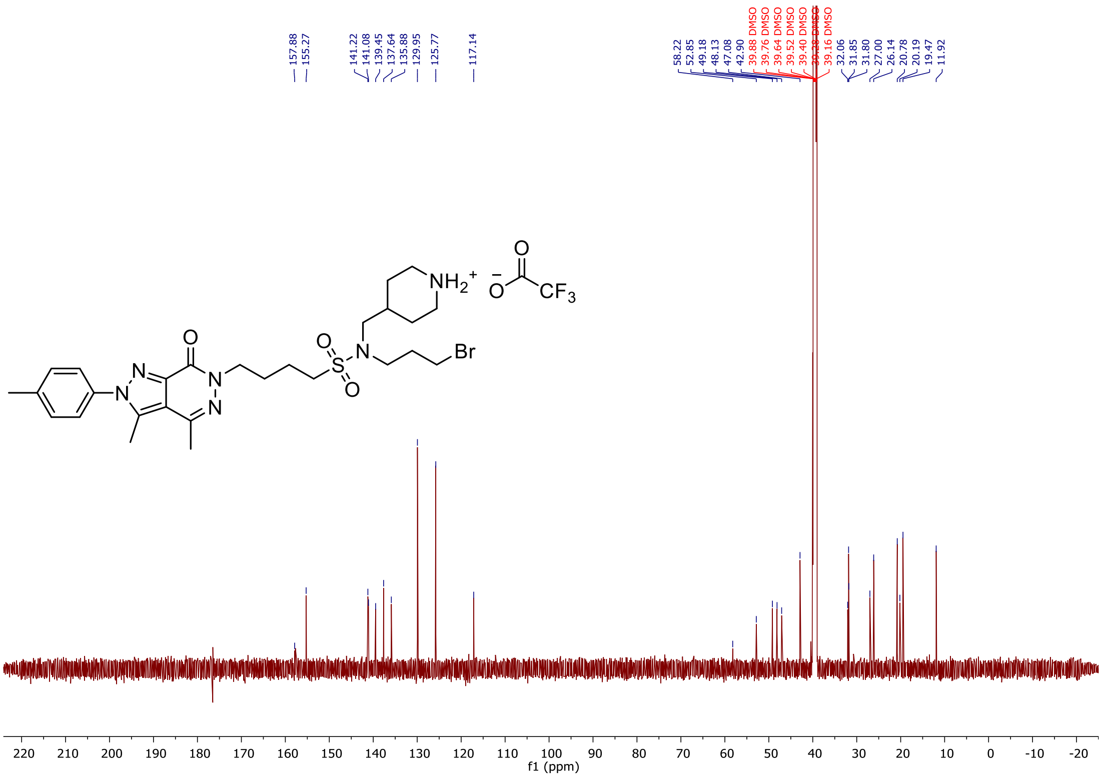

^{1}H NMR Spectrum of **5c** (700 MHz, DMSO-d_6)

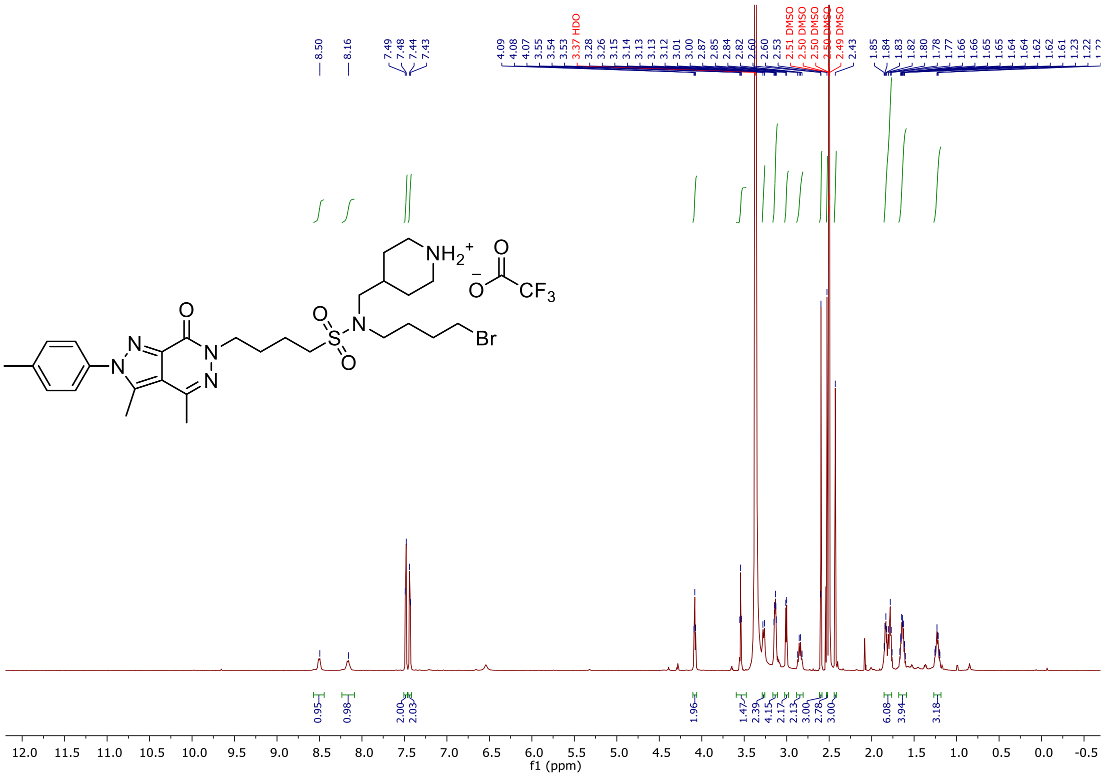

^{13}C NMR Spectrum of **5c** (176 MHz, DMSO-d_6)

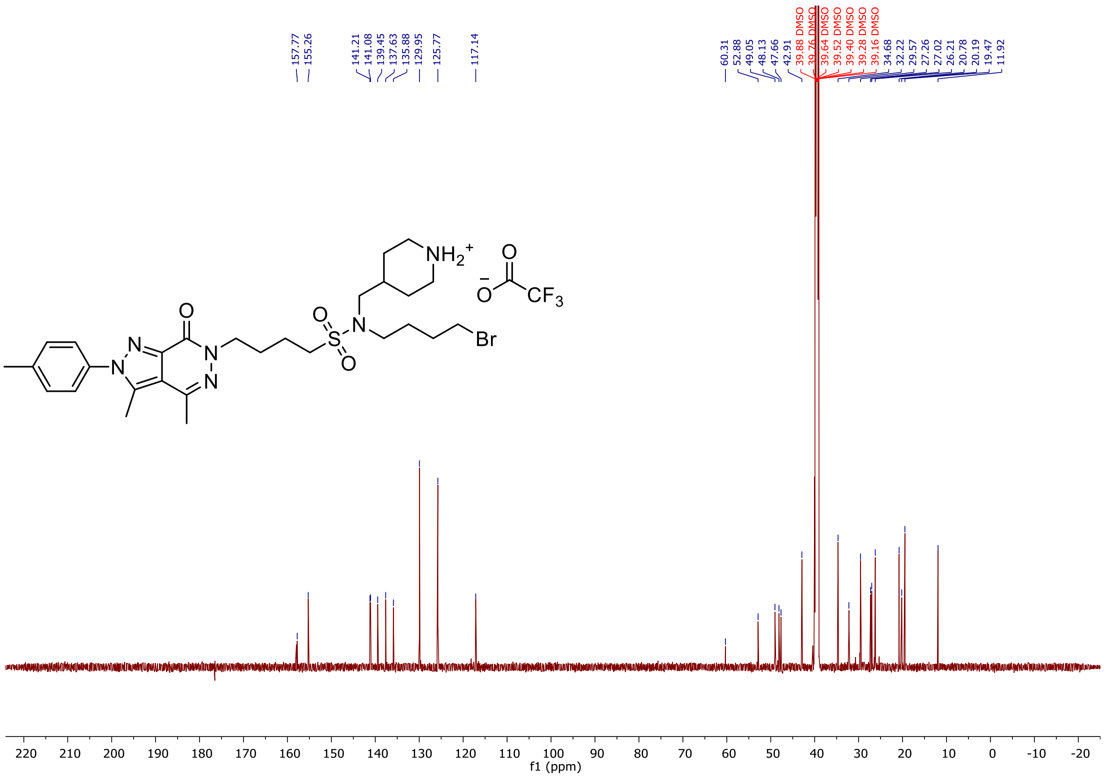

^{1}**H NMR** Spectrum of **5d** (700 MHz, DMSO-d_6)

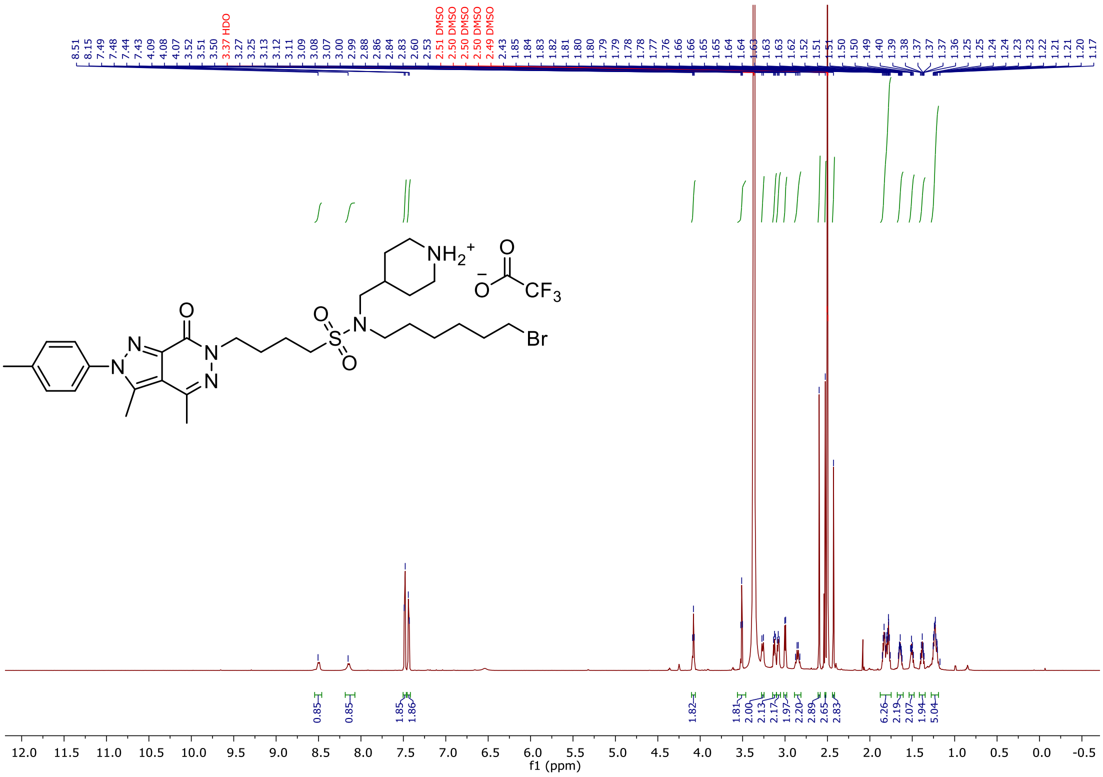

^{13}**C NMR** Spectrum of **5d** (176 MHz, DMSO-d_6)

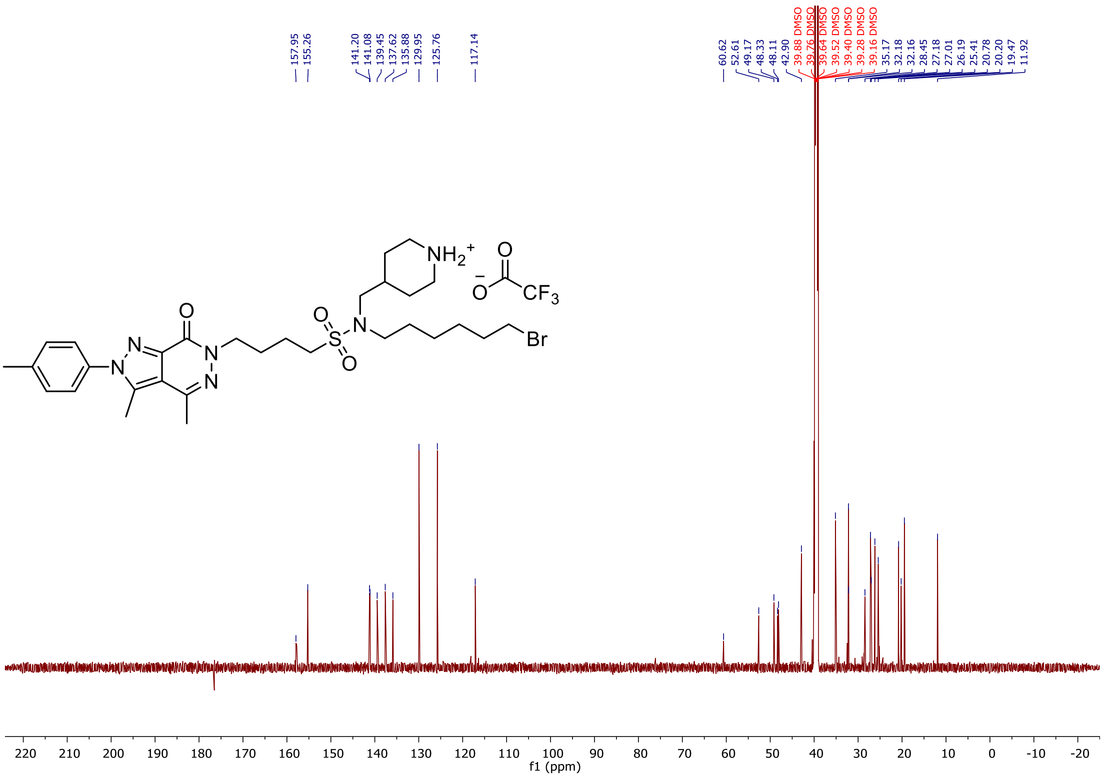

^{1}H NMR Spectrum of **5e** (700 MHz, DMSO-d_6)

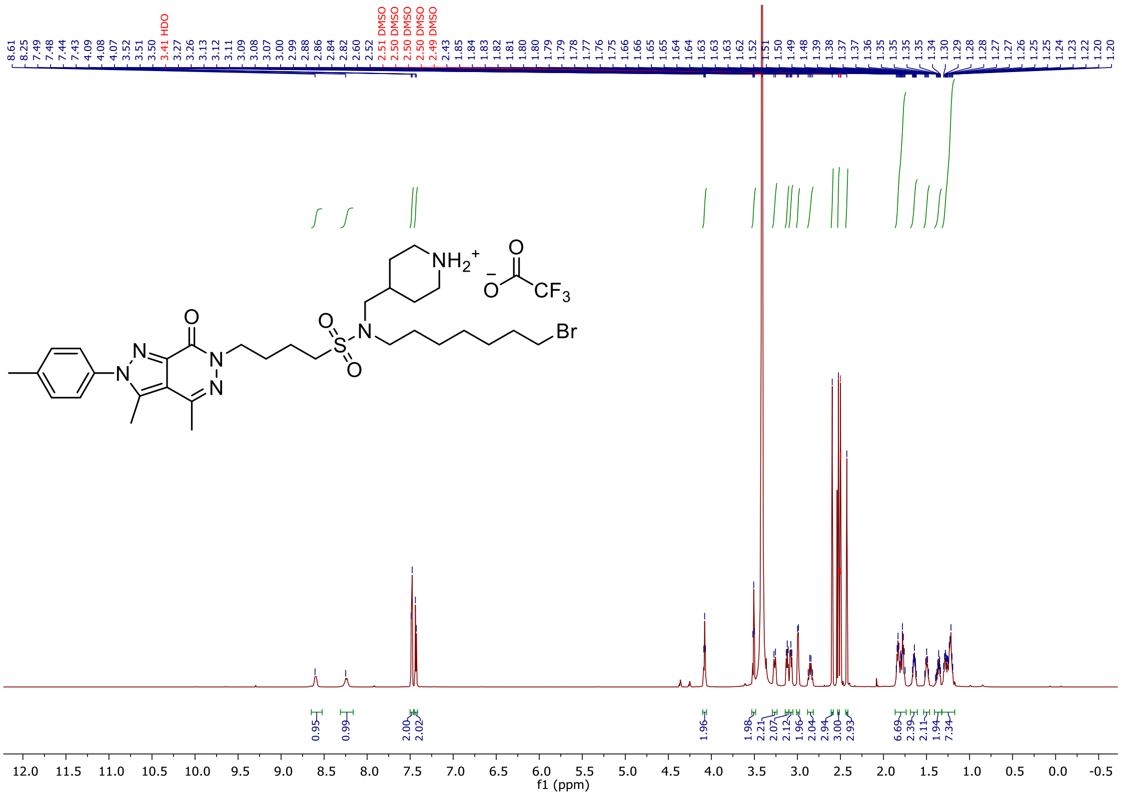

^{13}C NMR Spectrum of **5e** (176 MHz, DMSO-d_6)

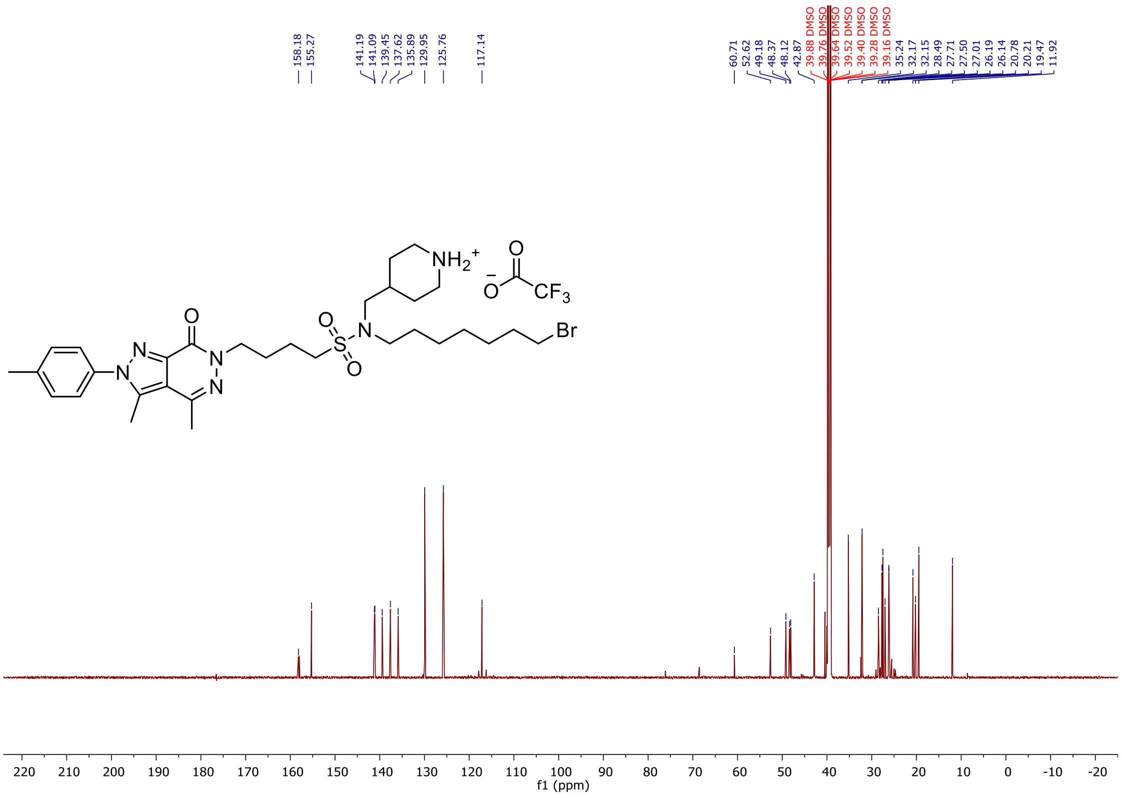

^{1}H NMR Spectrum of **5f** (700 MHz, DMSO-d_6)

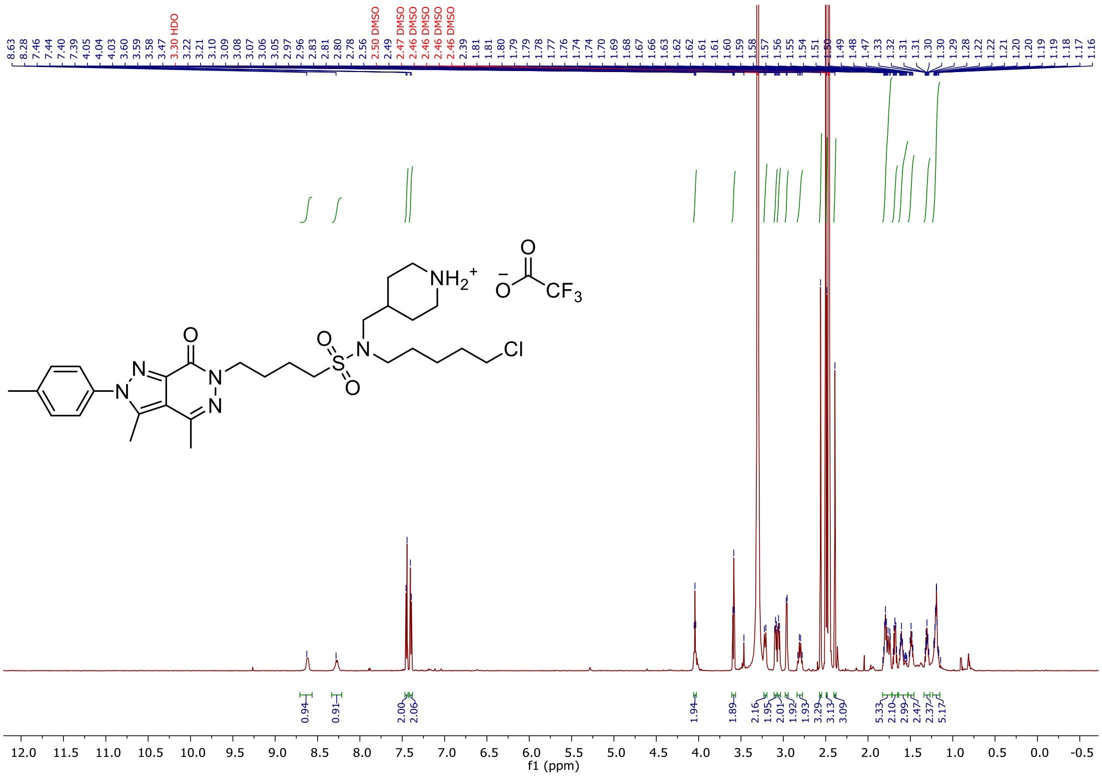

^{13}C NMR Spectrum of **5f** (176 MHz, DMSO-d_6)

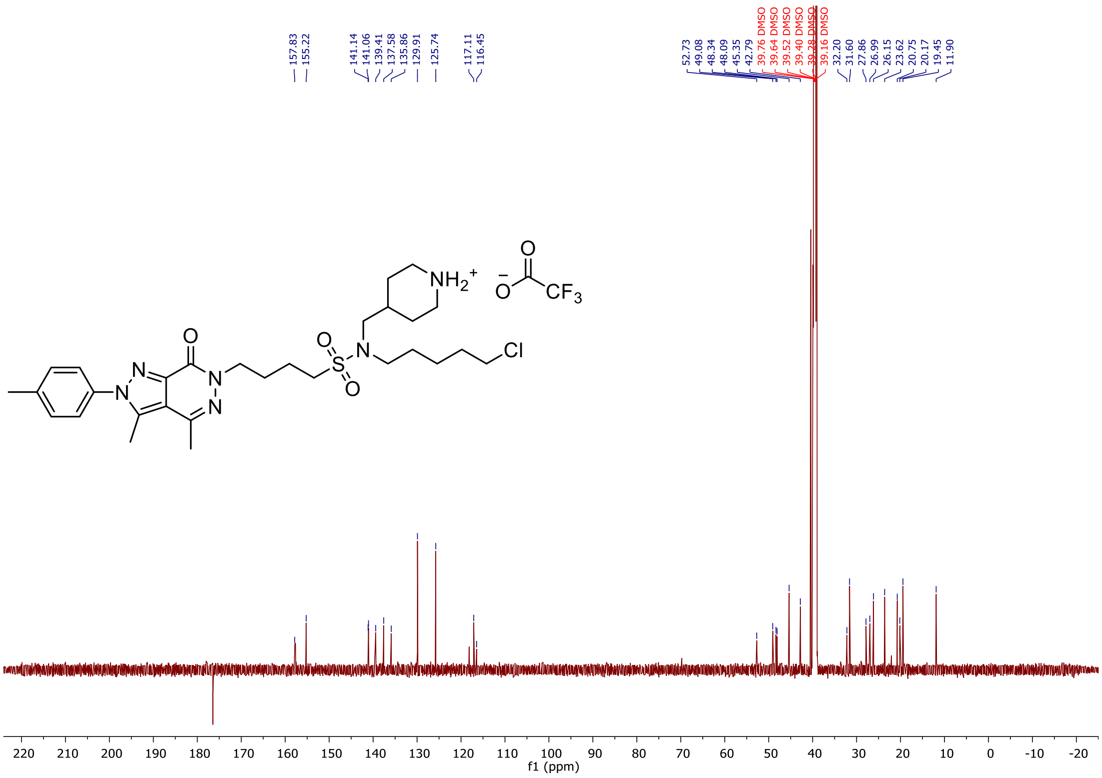

^{1}H NMR Spectrum of **5g** (500 MHz, DMSO-d_6)

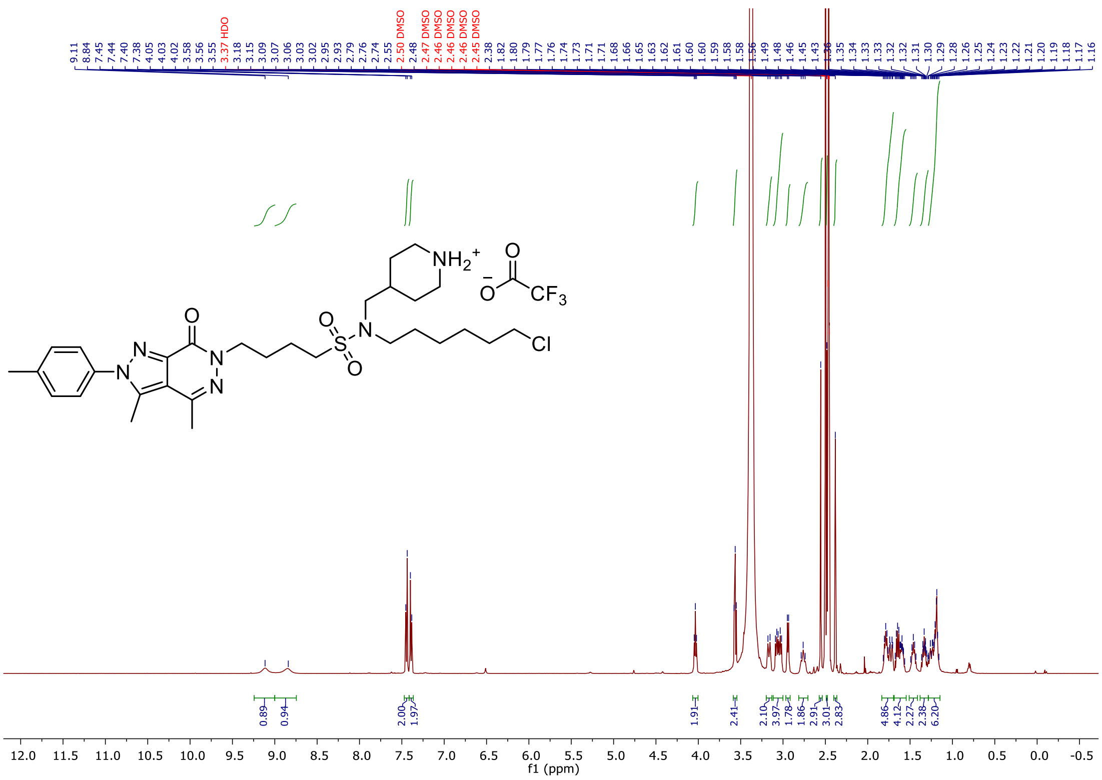

^{13}C NMR Spectrum of **5g** (126 MHz, DMSO-d_6)

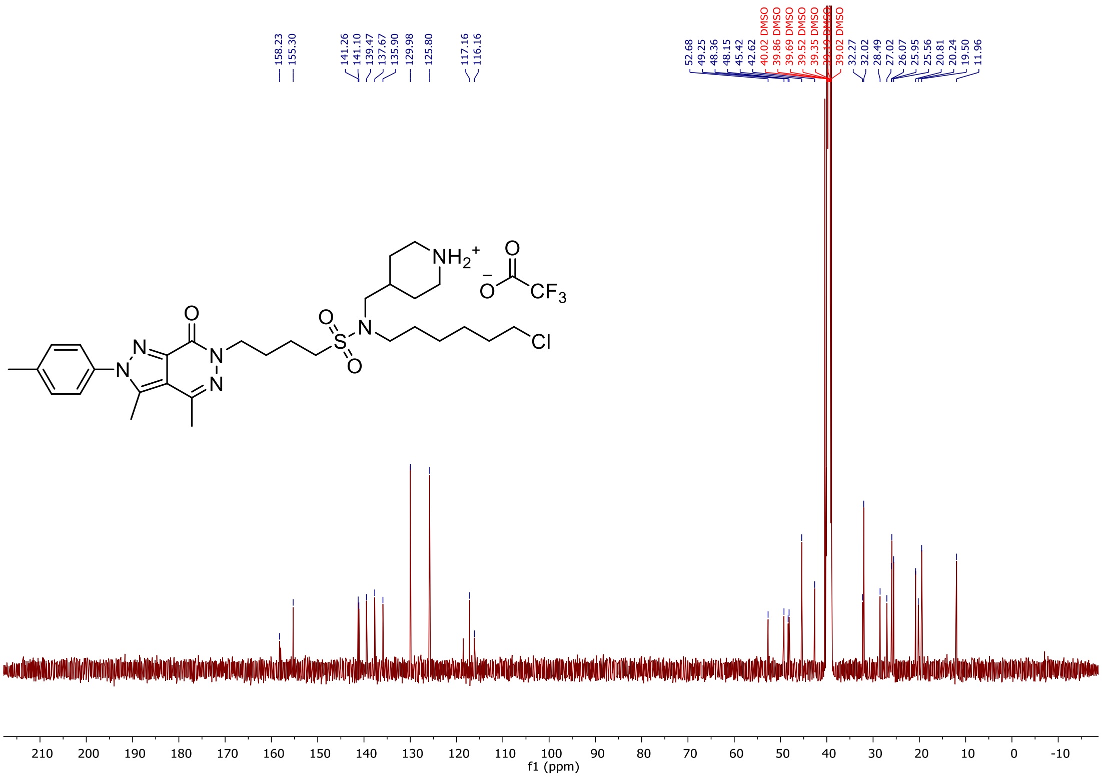

^{1}H NMR Spectrum of **5h** (500 MHz, DMSO-d_6)

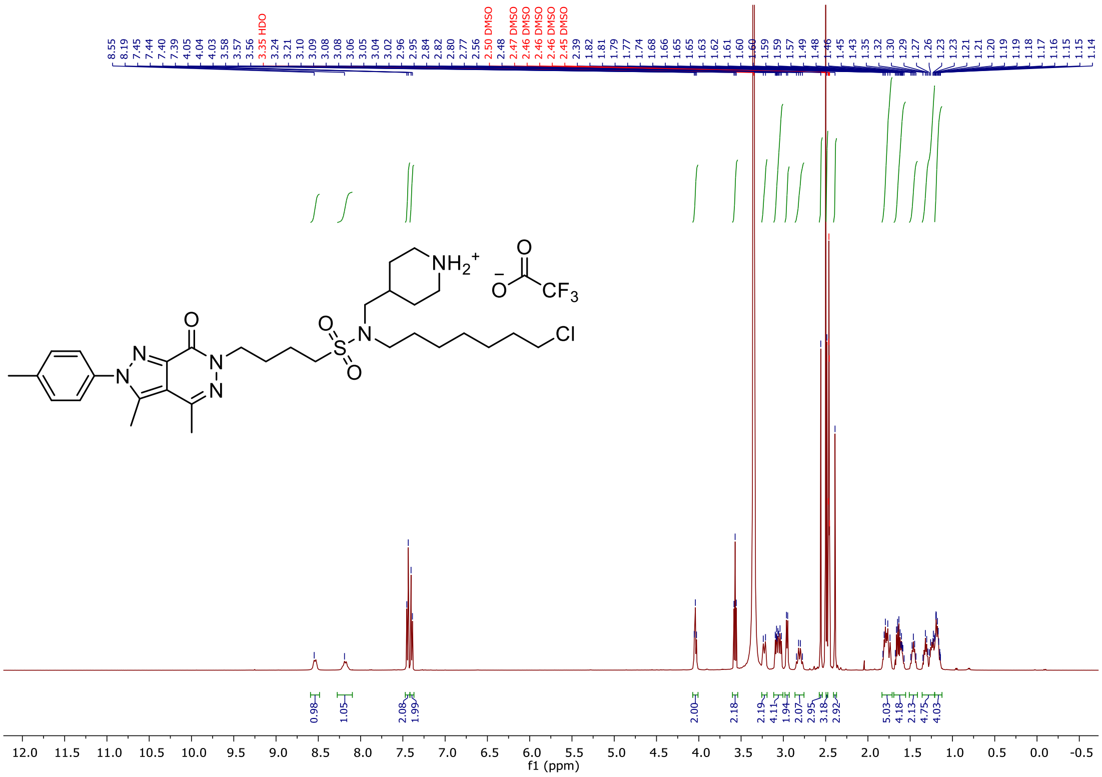

^{13}C NMR Spectrum of **5h** (126 MHz, DMSO-d_6)

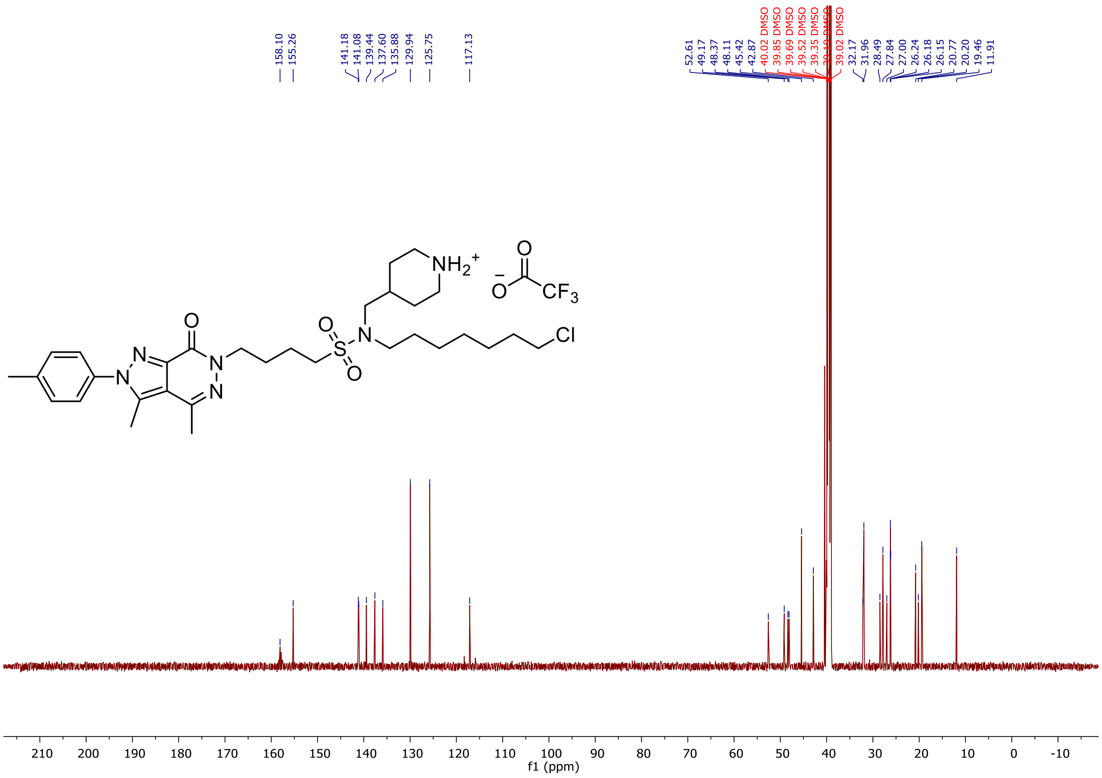

^{1}H NMR Spectrum of **5i** (700 MHz, DMSO-d_6)

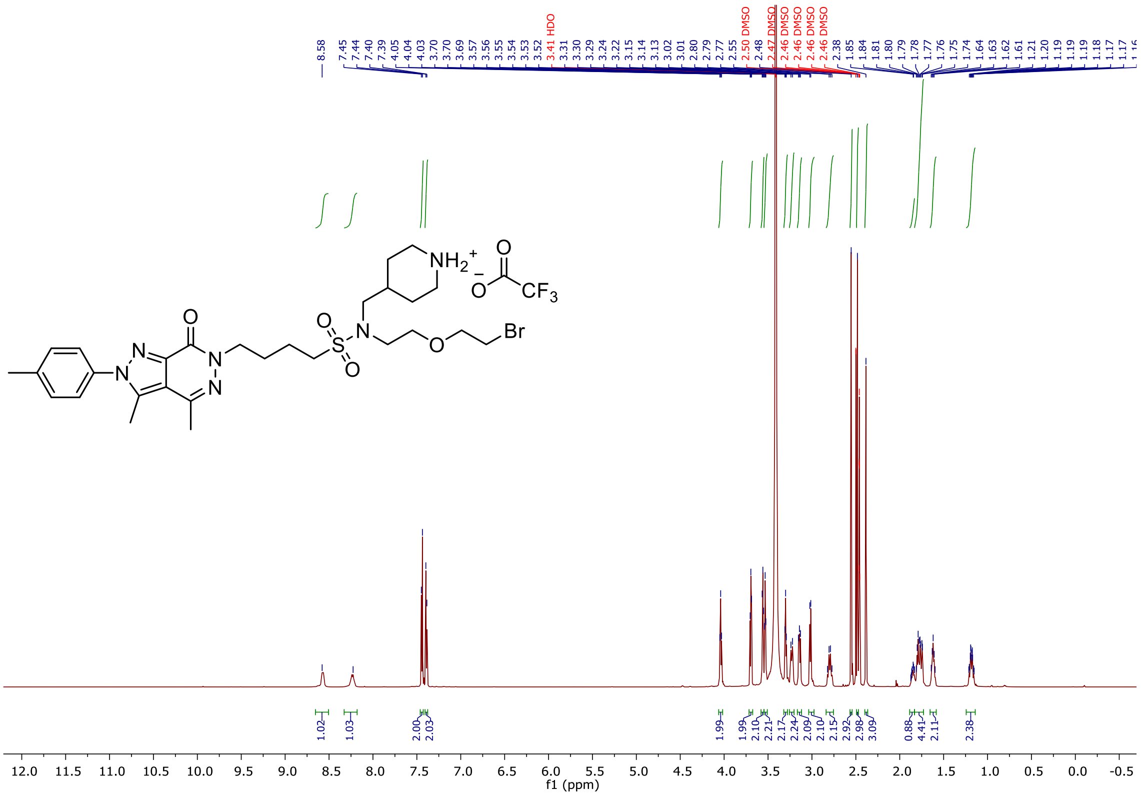

^{13}C NMR Spectrum of **5i** (176 MHz, DMSO-d_6)

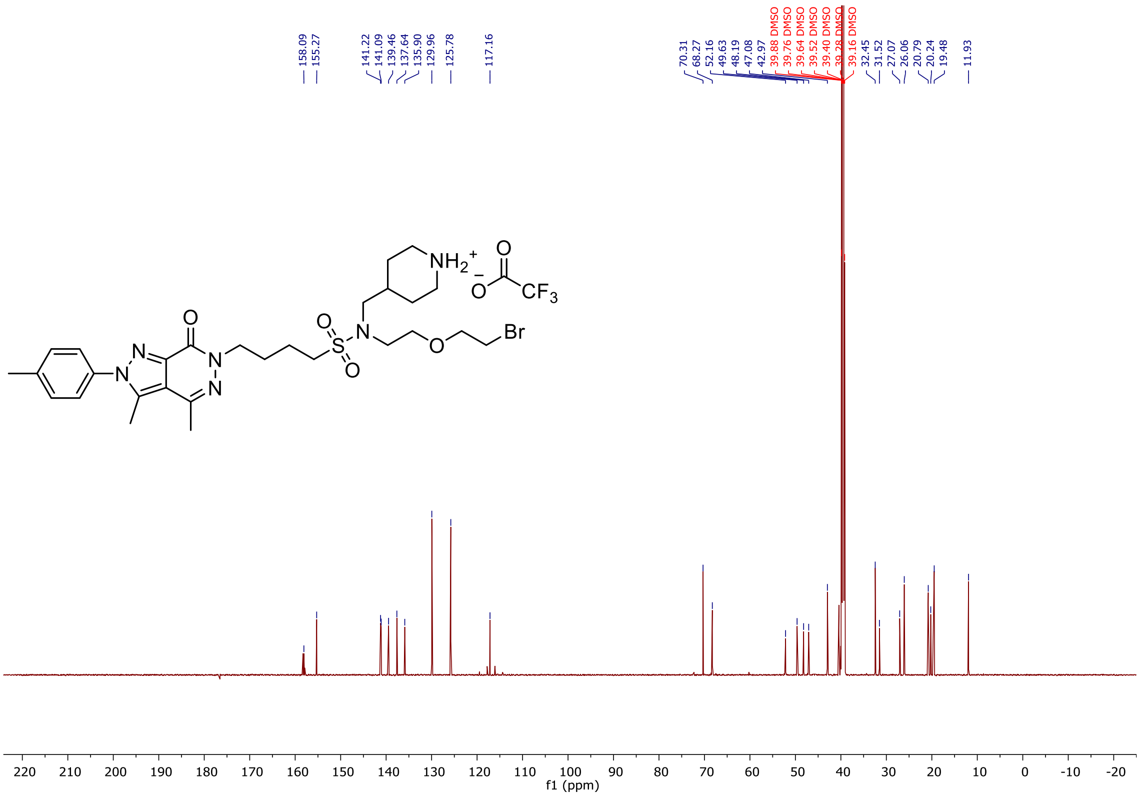

^{1}H NMR Spectrum of **5j** (500 MHz, DMSO-d_6)

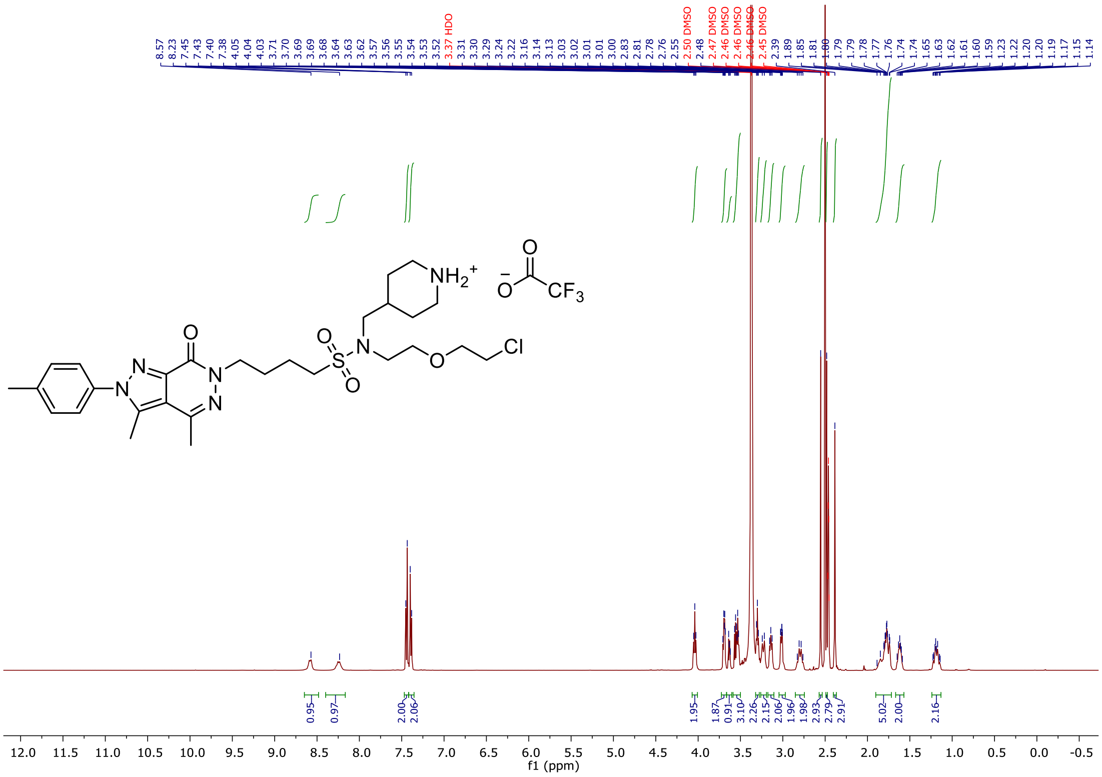

^{13}C NMR Spectrum of **5j** (126 MHz, DMSO-d_6)

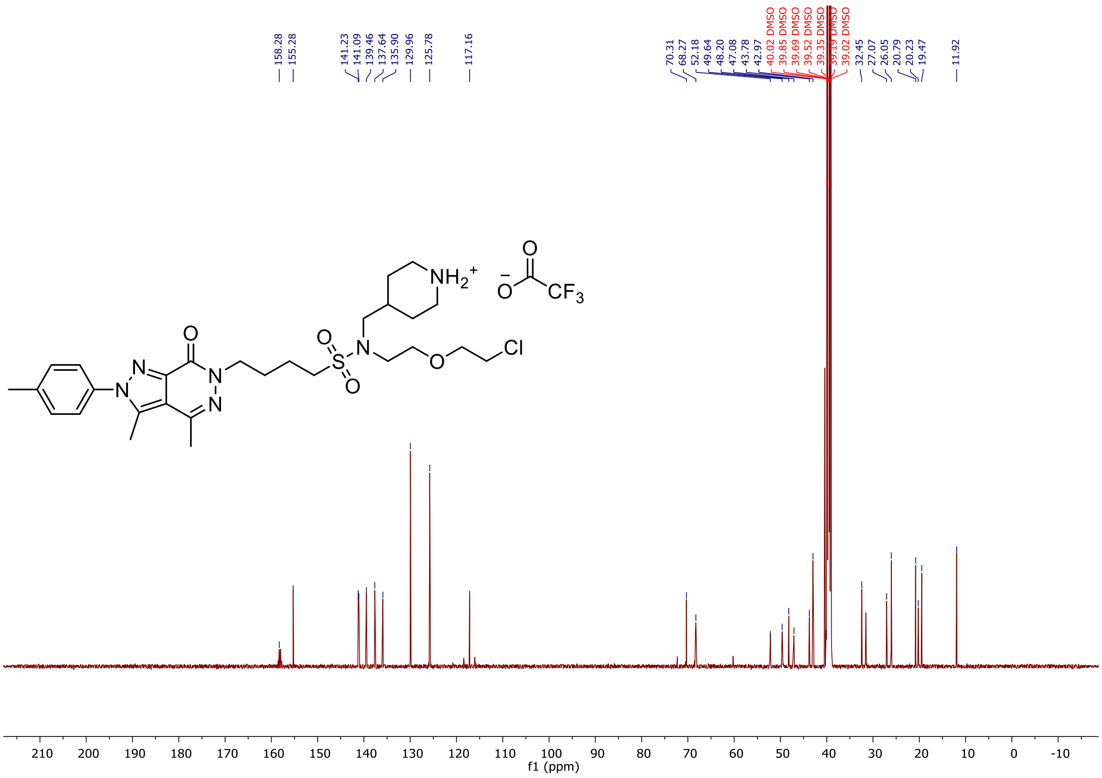

^{1}H NMR Spectrum of **5k** (500 MHz, DMSO-d_6)

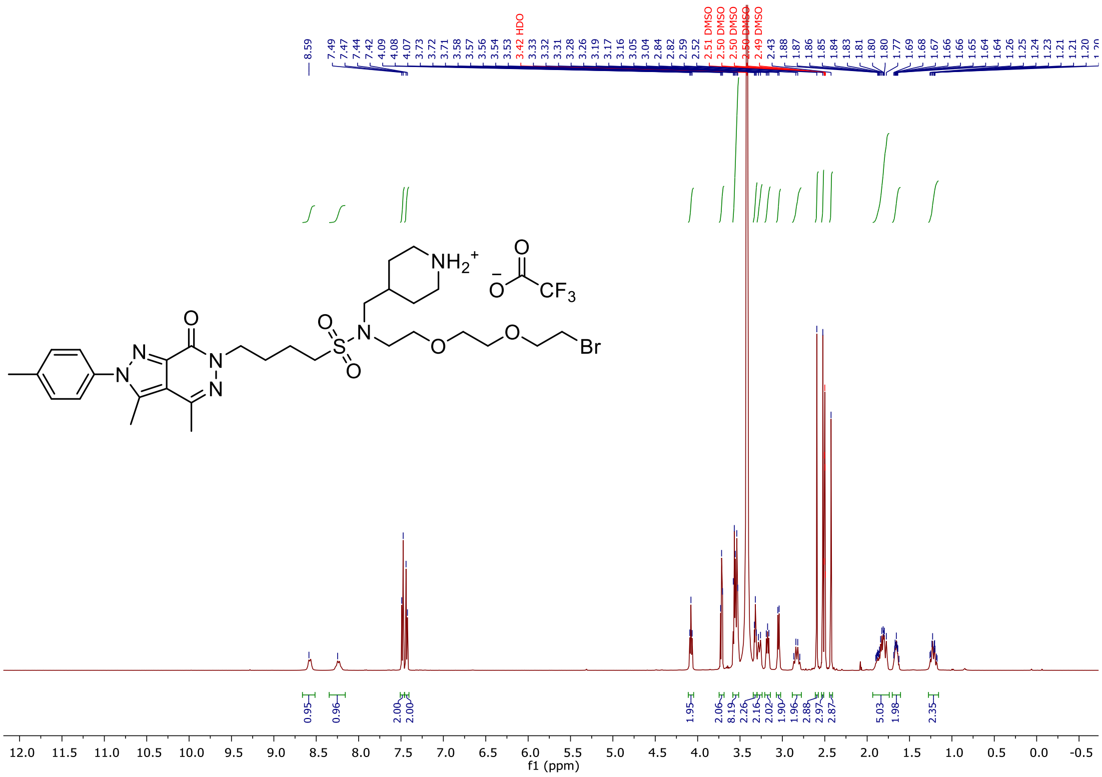

^{13}C NMR Spectrum of **5k** (126 MHz, DMSO-d_6)

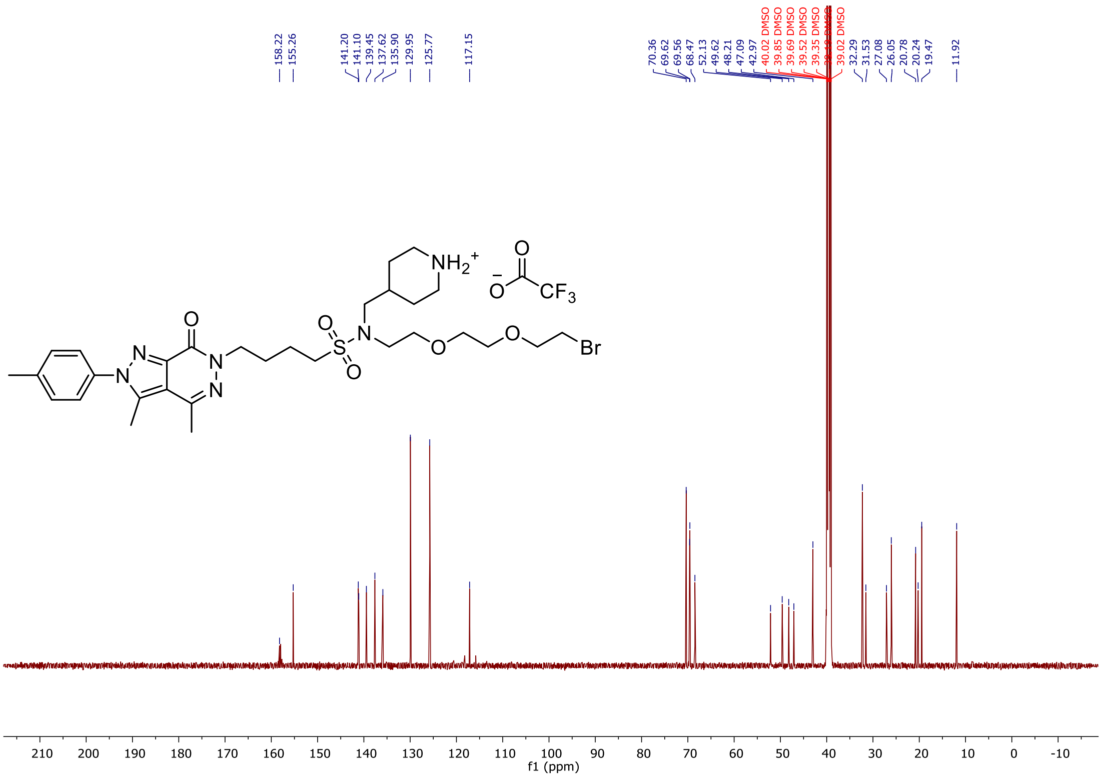

^{1}H NMR Spectrum of 5l (500 MHz, DMSO-d_6)

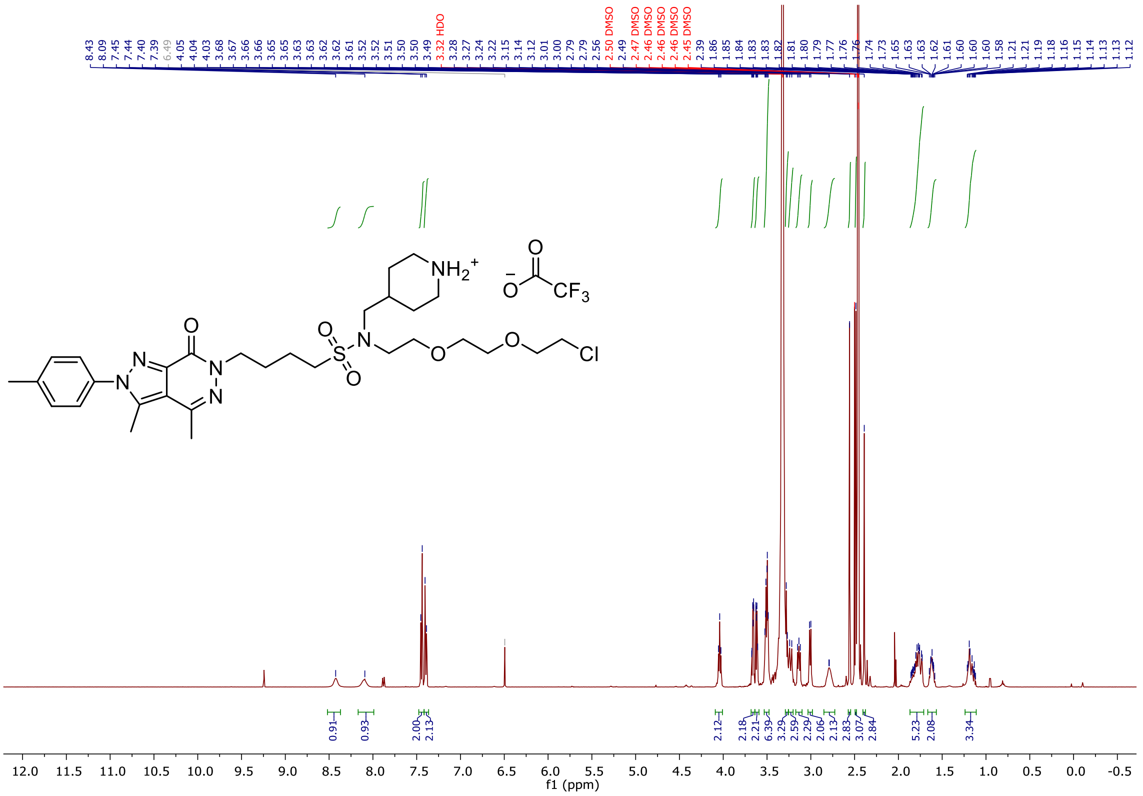

^{13}C NMR Spectrum of 5l (126 MHz, DMSO-d_6)

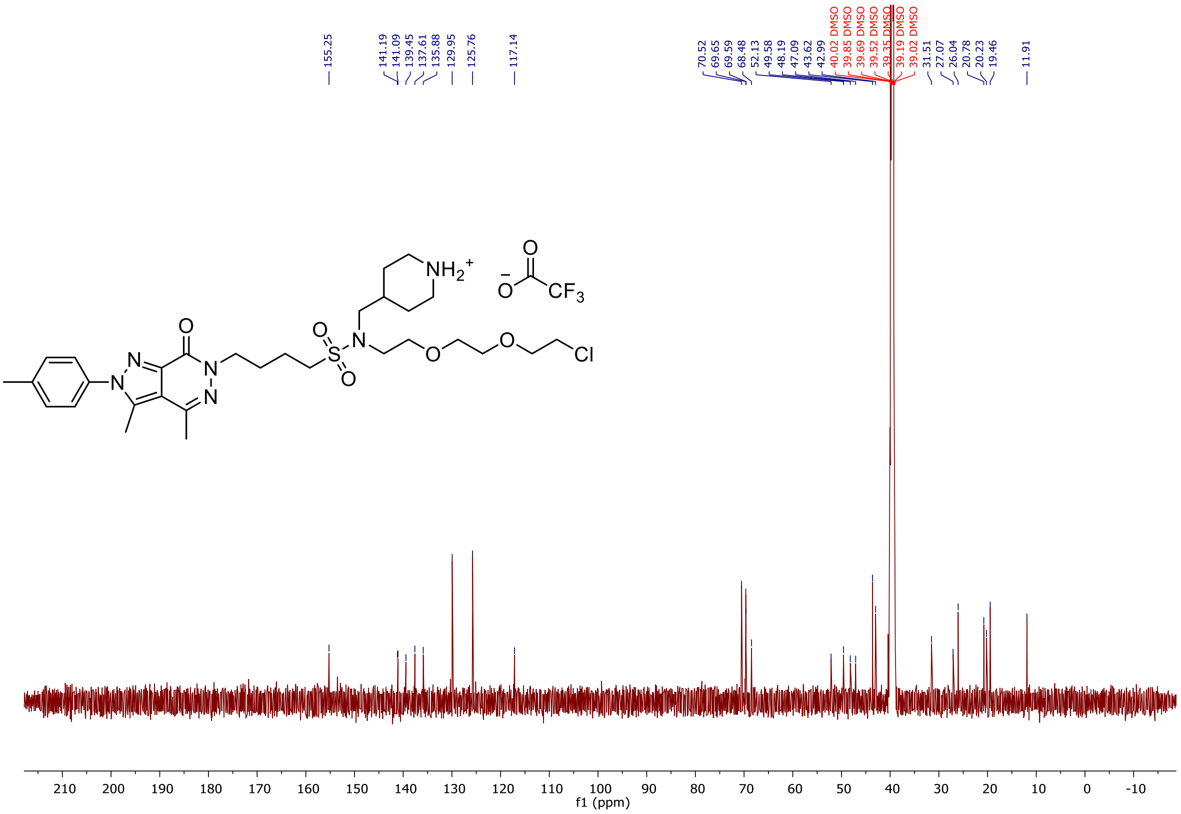

^{1}**H NMR** Spectrum of **6a** (700 MHz, DMSO-d_6)

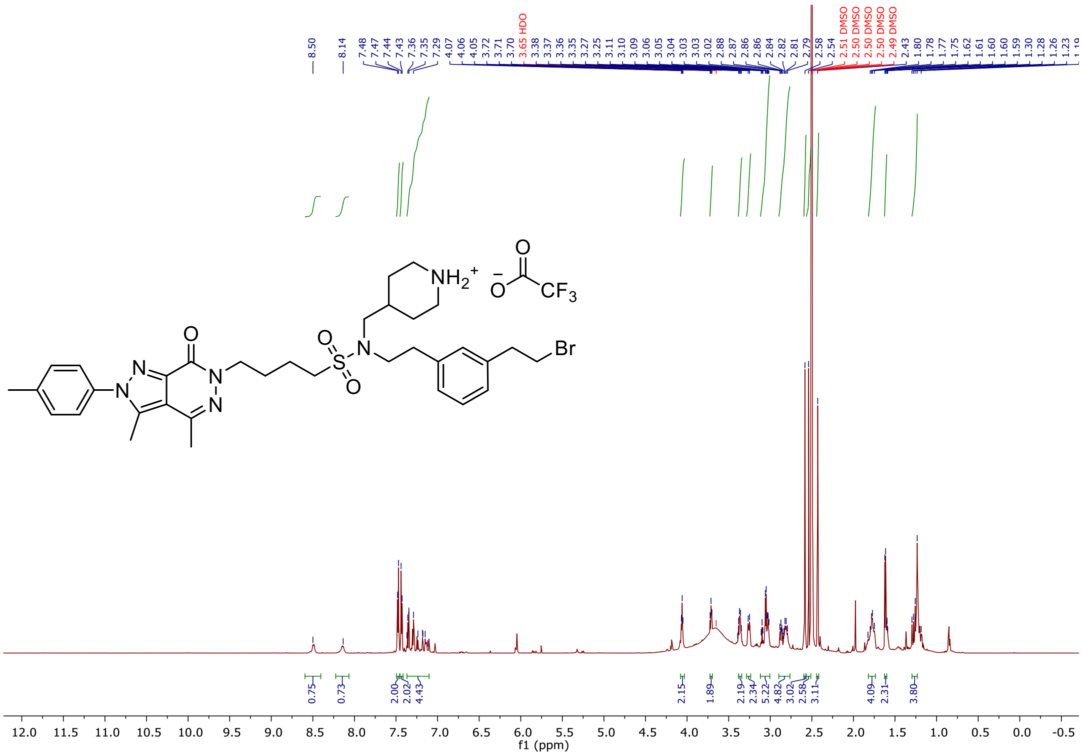

^{13}**C NMR** Spectrum of **6a** (176 MHz, DMSO-d_6)

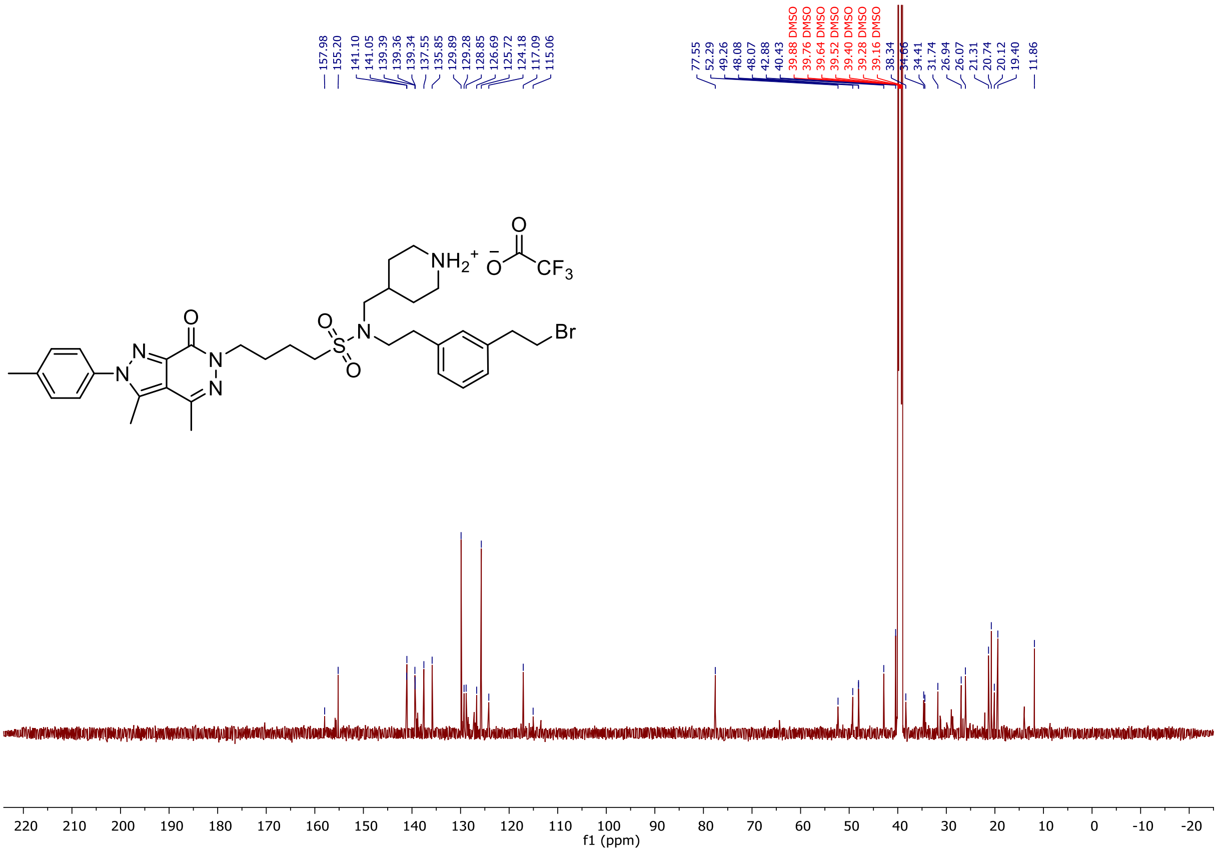

^{1}H NMR Spectrum of **6b** (700 MHz, DMSO-d_6)

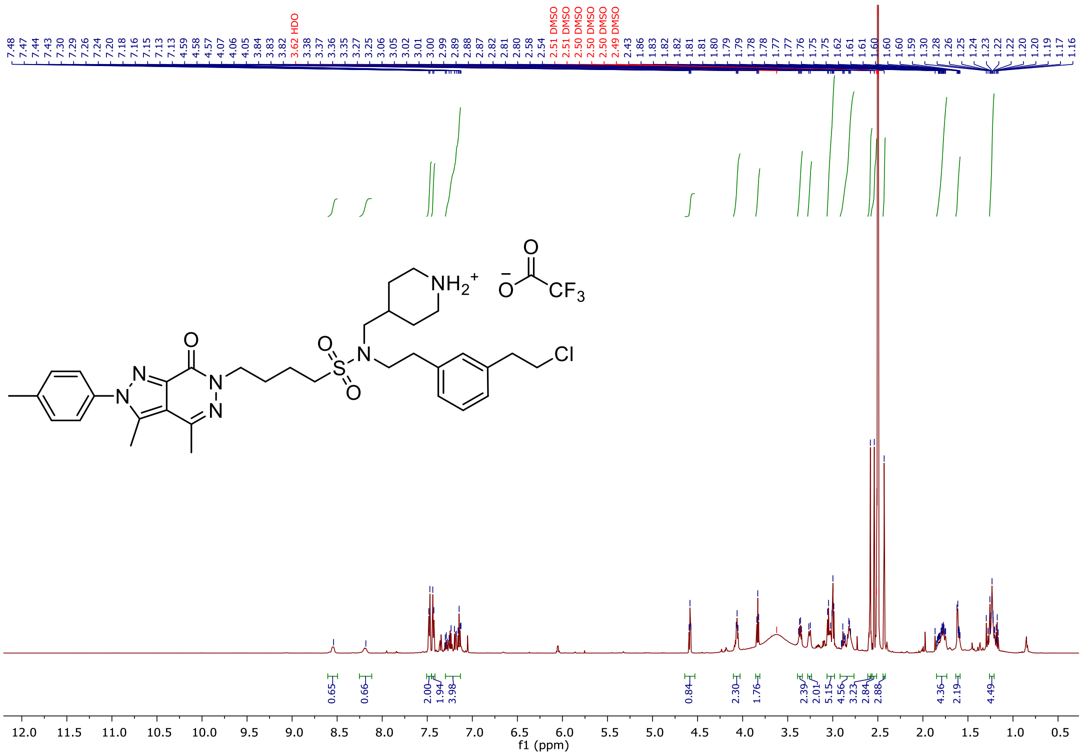

^{13}C NMR Spectrum of **6b** (176 MHz, DMSO-d_6)

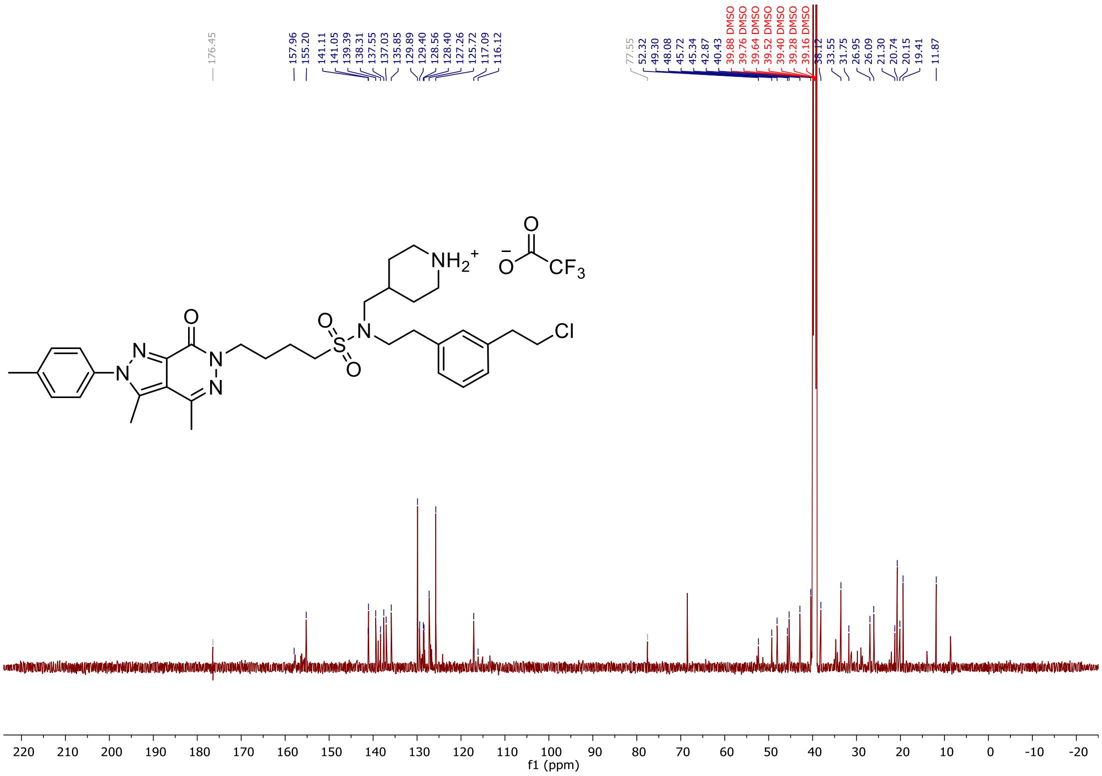

5. Supplementary References

1 Zhang, H. *et al.* Phospho-Analyst: An Interactive, Easy-to-Use Web Platform To Analyze Quantitative Phosphoproteomics Data. *J. Proteome Res.* **22**, 2890-2899, doi:10.1021/acs.jproteome.3c00186 (2023).

2 Kanehisa, M., Furumichi, M., Sato, Y., Ishiguro-Watanabe, M. & Tanabe, M. KEGG: integrating viruses and cellular organisms. *Nucleic Acids Res.* **49**, D545-D551, doi:10.1093/nar/gkaa970 (2021).

3 Luo, W. & Brouwer, C. Pathview: an R/Bioconductor package for pathway-based data integration and visualization. *Bioinformatics* **29**, 1830-1831, doi:10.1093/bioinformatics/btt285 (2013).

4 Ge, S. X., Jung, D. & Yao, R. ShinyGO: a graphical gene-set enrichment tool for animals and plants. *Bioinformatics* **36**, 2628-2629, doi:10.1093/bioinformatics/btz931 (2020).

5 Olive, P. L. & Banáth, J. P. The comet assay: a method to measure DNA damage in individual cells. *Nat. Protoc.* **1**, 23-29, doi:10.1038/nprot.2006.5 (2006).

6 Ziegler, S., Sievers, S. & Waldmann, H. Morphological profiling of small molecules. *Cell Chem. Biol.* **28**, 300-319, doi:10.1016/j.chembiol.2021.02.012 (2021).

7 Pahl, A. *et al.* Morphological subprofile analysis for bioactivity annotation of small molecules. *Cell Chem. Biol.* **30**, 839-853.e837, doi:https://doi.org/10.1016/j.chembiol.2023.06.003 (2023).

8 Grizot, S. *et al.* Crystal Structure of the Rac1−RhoGDI Complex Involved in NADPH Oxidase Activation. *Biochemistry* **40**, 10007-10013, doi:10.1021/bi010288k (2001).

9 Bon, R. S. *et al.* Structure-Guided Development of Selective RabGGTase Inhibitors. *Angew. Chem., Int. Ed. Engl.* **50**, 4957-4961, doi:https://doi.org/10.1002/anie.201101210 (2011).

10 Abrami, L. *et al.* Palmitoylated acyl protein thioesterase APT2 deforms membranes to extract substrate acyl chains. *Nat. Chem. Biol.* **17**, 438-447, doi:10.1038/s41589-021-00753-2 (2021).

11 Jaiswal, M. *et al.* Novel Biochemical and Structural Insights into the Interaction of Myristoylated Cargo with Unc119 Protein and Their Release by Arl2/3*. *J. Biol. Chem.* **291**, 20766-20778, doi:https://doi.org/10.1074/jbc.M116.741827 (2016).

12 Mejuch, T. *et al.* Small-Molecule Inhibition of the UNC119–Cargo Interaction. *Angew. Chem., Int. Ed. Engl.* **56**, 6181-6186, doi:https://doi.org/10.1002/anie.201701905 (2017).

13 Yelland, T., Garcia, E., Samarakoon, Y. & Ismail, S. The Structural and Biochemical Characterization of UNC119B Cargo Binding and Release Mechanisms. *Biochemistry* **60**, 1952-1963, doi:10.1021/acs.biochem.1c00251 (2021).

14 Daina, A., Michielin, O. & Zoete, V. SwissADME: a free web tool to evaluate pharmacokinetics, drug-likeness and medicinal chemistry friendliness of small molecules. *Sci. Rep.* **7**, 42717, doi:10.1038/srep42717 (2017).

15 Kong, A. T., Leprevost, F. V., Avtonomov, D. M., Mellacheruvu, D. & Nesvizhskii, A. I. MSFragger: ultrafast and comprehensive peptide identification in mass spectrometry–based proteomics. *Nat. Methods* **14**, 513-520, doi:10.1038/nmeth.4256 (2017).

16 Yu, F. *et al.* Analysis of DIA proteomics data using MSFragger-DIA and FragPipe computational platform. *Nat. Commun.* **14**, 4154, doi:10.1038/s41467-023-39869-5 (2023).

17 Yang, K. L. *et al.* MSBooster: improving peptide identification rates using deep learning-based features. *Nat. Commun.* **14**, 4539, doi:10.1038/s41467-023-40129-9 (2023).

18 Demichev, V., Messner, C. B., Vernardis, S. I., Lilley, K. S. & Ralser, M. DIA-NN: neural networks and interference correction enable deep proteome coverage in high throughput. *Nat. Methods* **17**, 41-44, doi:10.1038/s41592-019-0638-x (2020).

19 da Veiga Leprevost, F. *et al.* Philosopher: a versatile toolkit for shotgun proteomics data analysis. *Nat. Methods* **17**, 869-870, doi:10.1038/s41592-020-0912-y (2020).

20 Tyanova, S. *et al.* The Perseus computational platform for comprehensive analysis of (prote)omics data. *Nat. Methods* **13**, 731-740, doi:10.1038/nmeth.3901 (2016).
21 Cox, J. & Mann, M. MaxQuant enables high peptide identification rates, individualized p.p.b.-range mass accuracies and proteome-wide protein quantification. *Nat. Biotechnol.* **26**, 1367-1372, doi:10.1038/nbt.1511 (2008).
22 Reckzeh, E. S., Brockmeyer, A., Metz, M., Waldmann, H. & Janning, P. Target Engagement of Small Molecules: Thermal Profiling Approaches on Different Levels. *Methods Mol. Biol.* **1888**, 73-98, doi:10.1007/978-1-4939-8891-4_4 (2019).
23 Humphrey, S. J., Karayel, O., James, D. E. & Mann, M. High-throughput and high-sensitivity phosphoproteomics with the EasyPhos platform. *Nat. Protoc.* **13**, 1897-1916, doi:10.1038/s41596-018-0014-9 (2018).
24 Casado, P. *et al.* Kinase-Substrate Enrichment Analysis Provides Insights into the Heterogeneity of Signaling Pathway Activation in Leukemia Cells. *Sci. Signaling* **6**, rs6-rs6, doi:10.1126/scisignal.2003573 (2013).
25 Horn, H. *et al.* KinomeXplorer: an integrated platform for kinome biology studies. *Nat. Methods* **11**, 603-604, doi:10.1038/nmeth.2968 (2014).
26 Hornbeck, P. V. *et al.* PhosphoSitePlus, 2014: mutations, PTMs and recalibrations. *Nucleic Acids Res.* **43**, D512-D520, doi:10.1093/nar/gku1267 (2015).
27 Papke, B. *et al.* Identification of pyrazolopyridazinones as PDEδ inhibitors. *Nat. Commun.* **7**, 11360, doi:10.1038/ncomms11360 (2016).
28 Murarka, S. *et al.* Development of Pyridazinone Chemotypes Targeting the PDEδ Prenyl Binding Site. *Chem. - Eur. J* **23**, 6083-6093, doi:https://doi.org/10.1002/chem.201603222 (2017).
