# Supplementary material for: Covalent modification of a glutamic acid inspired by HaloTag technology
Source: Nat Commun. 2026 Jan 30;17:1257. doi: 10.1038/s41467-026-68999-9 (PMC12864978; doi:10.1038/s41467-026-68999-9)
Supplement: Supplementary file 2 — Reporting Summary [file 41467_2026_68999_MOESM2_ESM.pdf]

nature portfolio

Corresponding author(s): Herbert Waldmann

Last updated by author(s): Jan 13, 2026

Reporting Summary

Nature Portfolio wishes to improve the reproducibility of the work that we publish. This form provides structure for consistency and transparency in reporting. For further information on Nature Portfolio policies, see our Editorial Policies and the Editorial Policy Checklist.

Statistics

For all statistical analyses, confirm that the following items are present in the figure legend, table legend, main text, or Methods section.

n/a	Confirmed	
☐	☒	The exact sample size (*n*) for each experimental group/condition, given as a discrete number and unit of measurement
☐	☒	A statement on whether measurements were taken from distinct samples or whether the same sample was measured repeatedly
☐	☒	The statistical test(s) used AND whether they are one- or two-sided<br>*Only common tests should be described solely by name; describe more complex techniques in the Methods section.*
☒	☐	A description of all covariates tested
☒	☐	A description of any assumptions or corrections, such as tests of normality and adjustment for multiple comparisons
☐	☒	A full description of the statistical parameters including central tendency (e.g. means) or other basic estimates (e.g. regression coefficient) AND variation (e.g. standard deviation) or associated estimates of uncertainty (e.g. confidence intervals)
☐	☒	For null hypothesis testing, the test statistic (e.g. *F*, *t*, *r*) with confidence intervals, effect sizes, degrees of freedom and *P* value noted<br>*Give P values as exact values whenever suitable.*
☒	☐	For Bayesian analysis, information on the choice of priors and Markov chain Monte Carlo settings
☒	☐	For hierarchical and complex designs, identification of the appropriate level for tests and full reporting of outcomes
☒	☐	Estimates of effect sizes (e.g. Cohen's *d*, Pearson's *r*), indicating how they were calculated

Our web collection on statistics for biologists contains articles on many of the points above.

Software and code

Policy information about availability of computer code

Data collection	Immunoblotting: ChemiDocTM MP system (Bio-Rad) operated on Image Lab (v2.4.0.03).<br>Mass spectrometry: Bruker UltrafleXtreme XIAL DI-TOF/TOF mass spectrometer, Agilent Technologies 1290 Infinity, 6150 Quadrupole LC/MS, UltiMateTM 3000 RSLCnano system (Thermo Fisher Scientific, Germany) online coupled to a Q Exactive™ HF Hybrid Quadrupole-Orbitrap Mass Spectrometer equipped with a nanospray source (Nanospray Flex Ion Source, Thermo Fisher Scientific), LTQ Orbitrap mass spectrometer coupled to an Accela HPLC-System (HPLC column: Hypersyl GOLD, 50 mm x 1 mm, particle size 1.9 µm, ionization method: electron spray ionization), Orbitrap Fusion LUMOS instrument (Thermo) coupled to an Vanquish Neo ultra-performance liquid chromatography (UPLC) system (Thermo) controlled by the Orbitrap Fusion Lumos Tune Application (version 4.1.4244) and operated using the Xcalibur software (version 4.7.69.37).<br>NanoDSF: Prometheus Panta device (NanoTemper® Technologies).<br>Fluorescence polarisation: Spark® multimode microplate plate reader (Tecan).<br>X-ray crystallography data collection: Bruker D8 Discover home source with a Bruker Photon III detector and Diffrac software.<br>Microscopy: Leica SP8 (Leica Microsystems) with Leica Application Suite X (LAS X), Observer Z1 (Carl Zeiss, Germany).<br>Live-cell analysis: IncuCyte S3 system (Sartorius).<br>NMR: Bruker DRX400 (400 MHz), Bruker DRX500 (500 MHz), INOVA500 (500 MHz) or Bruker DRX700.
Data analysis	Immunoblot quantification: Image Lab (v6.0).<br>Data compiling and processing: Microsoft Excel (version 2108), GraphPad Prism (9.5.1).<br>Mass Spectrometry: MaxQuant (v.2.2.0.0), MSFragger (v 4.1.), Fragpipe (v 22).<br>X-ray analysis: XDS, XSCALE, Phaser(Phenix suite), phenix.refine, COOT, PyMOL Molecular Graphics System (2.5.4, Schrödinger, LLC), AceDRG within the CCP4 suite, LigPlot Plus.

NMR: MestReNova x64.
Microscopy: Leica Application Suite X (LAS X), Zen application (v2.3, Carl Zeiss, Germany), Image J with OpenComet plugin (v1.3).

For manuscripts utilizing custom algorithms or software that are central to the research but not yet described in published literature, software must be made available to editors and reviewers. We strongly encourage code deposition in a community repository (e.g. GitHub). See the Nature Portfolio guidelines for submitting code & software for further information.

Data

Policy information about availability of data

All manuscripts must include a data availability statement. This statement should provide the following information, where applicable:

- Accession codes, unique identifiers, or web links for publicly available datasets
- A description of any restrictions on data availability
- For clinical datasets or third party data, please ensure that the statement adheres to our policy

All data generated or analysed during this study are available within the article and its Supplementary Information files, and from the corresponding author upon request. The proteomics data related to DeltaTag have been deposited in MassIVE with the following accession codes: MSV000096863, PXD059864 (https://doi.org/doi:10.25345/C5GH9BN3J, artificial modification of PDEδ by 5e) and MSV000096865, PXD059867 (https://doi.org/doi:10.25345/C5736MD8P, artificial modification of PDEδ by 6a), MSV000096867, PXD059870 (https://doi.org/doi:10.25345/C5ZK55Z8F, phosphoproteomic profiling of 6a), and MSV000096869, PXD059872 (https://doi.org/doi:10.25345/C5Q23RC0W, thermal proteome profiling of 6a). The mass spectrometry proteomics data for the UNC119 experiment have been deposited to the ProteomeXchange Consortium via the PRIDE partner repository with the dataset identifier PXD067823 (https://www.ebi.ac.uk/pride/archive/projects/PXD067823). The crystal structures of PDEδ modified by compounds 5e and 6a (DeltaTag) were deposited in the Protein Data Bank (PDB) with the accession numbers 9HMC (https://doi.org/10.2210/pdb9HMC/pdb) and 9HMD respectively (https://doi.org/10.2210/pdb9HMD/pdb). Other crystal structures analysed in this study are available in the PDB database with the following accession numbers: 4JVF (https://doi.org/10.2210/pdb4JVF/pdb), 5E80 (https://doi.org/10.2210/pdb5E80/pdb), 5ML3 (https://doi.org/10.2210/pdb5ML3/pdb), 5NAL (https://doi.org/10.2210/pdb5NAL/pdb), 6ZVY (https://doi.org/10.2210/pdb6ZVY/pdb), 1HH4 (https://doi.org/10.2210/pdb1HH4/pdb), 3PZ2 (https://doi.org/10.2210/pdb3PZ2/pdb), 6QGS (https://doi.org/10.2210/pdb6QGS/pdb), 5L7K (https://doi.org/10.2210/pdb5L7K/pdb) and 7OK7 (https://doi.org/10.2210/pdb7OK7/pdb). Source data are provided with this paper.

Research involving human participants, their data, or biological material

Policy information about studies with human participants or human data. See also policy information about sex, gender (identity/presentation), and sexual orientation and race, ethnicity and racism.

Reporting on sex and gender	N/A
Reporting on race, ethnicity, or other socially relevant groupings	N/A
Population characteristics	N/A
Recruitment	N/A
Ethics oversight	N/A

Note that full information on the approval of the study protocol must also be provided in the manuscript.

Field-specific reporting

Please select the one below that is the best fit for your research. If you are not sure, read the appropriate sections before making your selection.

☒ Life sciences ☐ Behavioural & social sciences ☐ Ecological, evolutionary & environmental sciences

For a reference copy of the document with all sections, see nature.com/documents/nr-reporting-summary-flat.pdf

Life sciences study design

All studies must disclose on these points even when the disclosure is negative.

Sample size	Sample size was not predetermined. Sample size was selected based on previous experience and is indicated in each figure caption.
Data exclusions	No data were excluded except one outlier in Figure S5a (see Source Data).
Replication	All experiments were performed in three biological replicates unless otherwise stated. The number of biological replicates is specified in the respective figure legends.
Randomization	No randomisation was performed as is this is not standard for biochemical and in vitro studies. Internal controls were included for comparison.
Blinding	No blinding was performed as no subjective measurements were done.

Reporting for specific materials, systems and methods

We require information from authors about some types of materials, experimental systems and methods used in many studies. Here, indicate whether each material, system or method listed is relevant to your study. If you are not sure if a list item applies to your research, read the appropriate section before selecting a response.

Materials & experimental systems

n/a	Involved in the study
☐	☒ Antibodies
☐	☒ Eukaryotic cell lines
☒	☐ Palaeontology and archaeology
☒	☐ Animals and other organisms
☒	☐ Clinical data
☒	☐ Dual use research of concern
☒	☐ Plants

Methods

n/a	Involved in the study
☒	☐ ChIP-seq
☒	☐ Flow cytometry
☒	☐ MRI-based neuroimaging

Antibodies

Antibodies used

Anti-vinculin: Sigma-Aldrich, V9131, RRID: AB_477629, 1:5000;
anti-β-actin: Abcam, ab8227, RRID:AB_2305186, 1: 5000;
Anti-PDEδ: Invitrogen, PA5-22008, RRID: AB_11154288, 1:500;
Anti-Rheb: Santa Cruz Biotechnology Cat# sc-271509, RRID: AB_10659102, 1:500;
Anti-pS6P: Cell Signalling Technology, #4856, RRID: AB_2181037, 1:1000;
Anti-tS6P: Cell Signalling Technology, #2317, RRID: AB_2238583, 1:500;
IRDye 800CW-conjugated anti-mouse secondary antibody: LI-COR Biosciences, #926-32210, RRID: AB_621842, 1:5000;
Anti-mouse HRP: Invitrogen, #62-6520, RRID: AB_88369 for anti-mouse, 1:5000;
Anti-rabbit HRP: Invitrogen, #31460, RRID: AB_228341 for anti-rabbit, 1:5000;
Donkey anti-Mouse IgG (H+L) Highly Cross-Adsorbed Secondary Antibody, Alexa Fluor™ 555: Invitrogen # A-31570, RRID:AB_2536180, 1: 1000.

Validation

Anti-PDEδ antibody was validated by analysis in HAP1 wild type versus HAP1 PDEδ knockout cell lines (Supplementary Figure S5a, Source Data). Antibodies were validated by their manufacturers and their use is validated in numerous publications as exemplified by citeab.com.

Eukaryotic cell lines

Policy information about cell lines and Sex and Gender in Research

Cell line source(s)

Jurkat (ACC282, RRID: CVCL_0065), PA-TU-8902 (ACC179, RRID: CVCL_1845), BxPC3 cells (ACC760, RRID: CVCL_0186), HEP-G2 (ACC180, RRID: CVCL_0027) were purchased from DSMZ GmbH (Germany). MIA PaCa-2 (CRM-CRL-1420, RRID: CVCL_0428), SW480 (CCL-228, RRID: CVCL_0546), HEK293T (ATCC-CRL-3216, RRID: CVCL_0063) and CaCo-2 (ATCC-HTB-37, RRID: CVCL_0025) cells were obtained from ATCC (USA), and NCI-H358 (ATCC-CRL-5807, RRID: CVCL_1559) from LGC Standards (Germany). U2OS (CLS-300364, RRID: CVCL_0042) cells were obtained from CLS Cell Lines Service GmbH (Germany). HAP1 wild type (Horizon #C631, RRID: CVCL_Y019) and HAP1 PDE6D knockout (Horizon #HZGHC006484c003, RRID: CVCL_XR47) cells were purchased from Horizon (Horizon Discovery, UK).

Authentication

All used cell lines were authenticated by vendors and routinely authenticated via cell morphology.

Mycoplasma contamination

All used cell lines were routinely tested and confirmed negative for mycoplasma contamination.

Commonly misidentified lines
(See ICLAC register)

No commonly misidentified cell lines were used.

Plants

Seed stocks	N/A
Novel plant genotypes	N/A
Authentication	N/A
